# Phenotypic Differences in Inflammatory, Metabolic, and Biochemical Biomarkers in Dogs with Osteoarthritis According to Body Condition and Sex

**DOI:** 10.3390/ani16040692

**Published:** 2026-02-23

**Authors:** Liceth Agudelo-Giraldo, Catalina López, Jorge U. Carmona

**Affiliations:** 1 Grupo de Investigación Terapia Regenerativa, Departamento de Salud Animal, Universidad de Caldas, Calle 65 No 26-10, Manizales 170004, Colombia; liceth.agudelo@ucaldas.edu.co; 2 Grupo de Investigación Patología Clínica Veterinaria, Departamento de Salud Animal, Universidad de Caldas, Calle 65 No 26-10, Manizales 170004, Colombia; catalina.lopez@ucaldas.edu.co

**Keywords:** canine osteoarthritis, systemic inflammation, metabolic alterations, inflammatory biomarkers, adipokines, interleukin-1 beta, gamma-glutamyl transferase, cholesterol metabolism, body condition score, phenotypic heterogeneity

## Abstract

Osteoarthritis (OA) in dogs is increasingly recognized as a condition with systemic inflammatory and metabolic components, potentially influenced by body condition and sex. This study aimed to characterize phenotypic differences in circulating inflammatory, metabolic, and biochemical biomarkers in dogs with OA according to body condition and sex. In this cross-sectional study, client-owned dogs were classified as healthy controls, thin dogs with OA (TOA), or obese dogs with OA (OOA). Circulating cytokines, adipokines, cartilage degradation markers, and routine biochemical parameters were measured in blood samples, including interleukin-1 beta, interleukin-4, interleukin-10, adiponectin, C-terminal telopeptide of type II collagen, and standard metabolic and hepatic markers. Data were analyzed using linear models fitted on log-transformed values, with group and sex as fixed effects, complemented by adjusted and sensitivity analyses. TOA dogs showed significantly higher interleukin-1 beta concentrations compared with controls (multiplicative effect 1.39, 95% confidence interval 1.05–1.82), indicating increased systemic inflammatory activity. In contrast, OOA dogs exhibited predominantly metabolic-associated alterations, including higher gamma-glutamyl transferase activity (multiplicative effect 1.22, 95% confidence interval 1.03–1.46) and higher cholesterol concentrations (multiplicative effect 1.22, 95% confidence interval 1.03–1.46). Several other biomarkers showed no clear group-related differences. Overall, these findings demonstrate that systemic biomarker profiles in canine OA vary primarily according to body condition, with secondary sex-related patterns, supporting the existence of biologically distinct OA phenotypes relevant for future diagnostic and therapeutic strategies.

## Simple Summary

Osteoarthritis (OA) is a common joint disease in dogs that causes pain and reduced mobility. Although it primarily affects the joints, it can also influence the whole body through changes in inflammation and metabolism. Body condition, such as being overweight or underweight, as well as sex, may shape these systemic effects. The aim of this study was to evaluate blood markers related to inflammation, metabolism, organ function, and cartilage breakdown in dogs with OA, comparing thin and obese animals with healthy dogs. Blood samples from client-owned dogs were analyzed using statistical models that allowed fair comparisons between groups. Dogs with OA showed distinct biological patterns depending on body condition. Thin dogs tended to show higher inflammatory activity, whereas obese dogs mainly exhibited changes related to metabolic processes. Several commonly studied markers showed little or no difference between groups. These findings indicate that osteoarthritis in dogs is not a single uniform condition but includes different biological profiles, which may help improve understanding and clinical management of the disease.

## 1. Introduction

Osteoarthritis (OA) is one of the most prevalent chronic degenerative joint diseases in dogs and represents a major cause of pain, reduced mobility, and diminished quality of life. The disease is characterized by progressive cartilage degradation, synovial inflammation, and structural alterations in periarticular tissues, ultimately leading to functional impairment [1,2,3,4,5]. Although OA has traditionally been considered a localized musculoskeletal disorder, growing evidence supports its recognition as a condition with systemic inflammatory [6] and metabolic components [7].

Body condition, particularly obesity, is a well-established risk factor for canine OA. Excess body weight increases mechanical loading on joints, accelerating cartilage wear and joint degeneration. Beyond biomechanical stress, adipose tissue functions as an active endocrine organ, secreting adipokines and cytokines that contribute to chronic low-grade systemic inflammation [8,9,10,11]. This inflammatory and metabolic milieu may influence not only joint tissues but also liver function, energy metabolism, and overall biochemical homeostasis [12,13,14,15]. Importantly, OA is also frequently observed in dogs with normal or low body condition, suggesting that non-weight-related mechanisms also contribute to disease-associated systemic alterations [2].

Cytokines play a central role in the regulation of inflammatory and anti-inflammatory pathways involved in OA [16,17,18]. Pro-inflammatory mediators such as interleukin-1 beta (IL-1β) are directly implicated in cartilage matrix degradation, synovial inflammation, and the amplification of catabolic signaling within the joint [12,18,19]. Conversely, anti-inflammatory cytokines, including interleukin-4 (IL-4) and interleukin-10 (IL-10), contribute to immune regulation and may counterbalance pro-inflammatory activity [20,21]. While these mediators have been extensively studied at the local joint level, their systemic behavior in dogs with OA remains incompletely characterized.

In addition to cytokine signaling, OA (particularly when associated with altered body condition) may be accompanied by changes in metabolic and biochemical markers reflecting broader physiological adaptations or dysregulation [13,14,15]. Alterations in liver-associated enzymes (alanine aminotransferase (AST), aspartate aminotransferase (ALT), gamma-glutamyl transferase (GGT), and alkaline phosphatase (ALP)), renal and muscle-related markers (creatinine and urea), and metabolic parameters such as glucose and cholesterol have been reported in dogs with chronic inflammatory or metabolic conditions [22,23,24]. Adipokines such as adiponectin (ADP) may further modulate inflammatory responses and energy metabolism [25,26], while biomarkers of cartilage turnover, including the C-terminal telopeptide of type II collagen (CTX-II), provide insight into ongoing joint matrix degradation [27,28,29]. However, the extent to which these diverse biomarkers collectively reflect distinct osteoarthritic phenotypes remains poorly defined.

Systemic biomarker profiles in dogs with OA may vary according to body condition and sex, given the known influence of adipose tissue distribution, hormonal regulation, and metabolic status on inflammatory and biochemical pathways [2,8,9]. Despite this biological plausibility, available evidence in dogs remains fragmented and often limited to the evaluation of isolated biomarkers, such as C-reactive protein, selected interleukins, or cartilage degradation markers [30,31,32], without simultaneous assessment of metabolic mediators or consistent stratification according to body condition score and sex. As a result, reported findings are frequently heterogeneous and sometimes contradictory, hindering a comprehensive understanding of potential OA phenotypes.

Therefore, the aim of this study was to characterize phenotypic differences in inflammatory, metabolic, and biochemical biomarkers in dogs with OA according to body condition (weight) and sex. Specifically, circulating cytokines, adipokines, markers of cartilage degradation, and routine biochemical parameters were compared between healthy control dogs, thin dogs with OA (TOA), and obese dogs with OA (OOA) using a model-based analytical approach. By integrating inflammatory and metabolic perspectives, this study seeks to contribute to a more comprehensive understanding of OA as a multifactorial and systemic disease in dogs.

## 2. Materials and Methods

### 2.1. Study Design and Animals

This study was conducted as an observational, cross-sectional investigation involving client-owned dogs evaluated at the Veterinary Teaching Hospital of Universidad de Caldas, Manizales, Colombia. Dogs were recruited consecutively according to predefined inclusion and exclusion criteria and were classified into study groups based on OA status and body condition.

The animal study protocol was reviewed and approved by the Institutional Ethics Committee of Universidad de Caldas (Protocol code PRY-298; approved on 9 February 2025). All procedures were performed in accordance with institutional guidelines for animal research. Written informed consent was obtained from all owners prior to enrollment.

Dogs were allocated into three groups: healthy control dogs (CG), TOA, and OOA dogs. Healthy control dogs had no history or clinical signs of joint disease and showed no radiographic evidence of OA. Osteoarthritis diagnosis was established based on orthopedic examination and imaging findings consistent with degenerative joint disease, including osteophyte formation and joint remodeling [33]. Body condition was assessed by trained veterinarians using a standardized 9-point body condition scoring system, with thin dogs defined as a BCS ≤ 4 and obese dogs as a BCS ≥ 7 [34]. Sex was recorded for all animals and considered a fixed biological factor in subsequent analyses.

For the purposes of this study, the term “phenotype” is used in an operational and descriptive sense to denote clinically defined subgroups of dogs with osteoarthritis based on body condition and associated systemic biomarker profiles. Accordingly, phenotypic differences refer to patterns of circulating inflammatory, metabolic, and biochemical markers, rather than to genetically defined or causally inferred disease subtypes. Body condition was used to classify osteoarthritis phenotypes, whereas body weight was included as a continuous covariate in adjusted models to account for differences in overall body size [35,36].

### 2.2. Clinical Assessment, Eligibility Criteria, and Sample Collection

Dogs diagnosed with OA were identified at least two weeks prior to blood sample collection. Inclusion criteria for OA dogs consisted of a clinical diagnosis based on orthopedic examination and radiographic evidence of degenerative joint disease. Both monoarticular and polyarticular presentations were eligible for inclusion.

To minimize potential pharmacological interference with circulating inflammatory and metabolic mediators, dogs that had received non-steroidal anti-inflammatory drugs (NSAIDs), corticosteroids, or nutraceuticals were required to complete a washout period of at least two weeks before enrollment and sampling.

Exclusion criteria included the presence of concurrent systemic inflammatory, endocrine, or metabolic diseases, as well as ongoing pharmacological treatment that did not comply with the established washout period.

All enrolled dogs underwent a complete physical examination to assess general health status. Dogs presenting evidence of systemic illness unrelated to OA or with laboratory abnormalities suggestive of concurrent inflammatory, endocrine, or metabolic disorders were excluded.

Fasting blood samples were obtained by venipuncture under standardized conditions. Whole blood and plasma samples were processed according to routine clinical pathology protocols. Aliquots intended for biomarker analyses were stored under appropriate conditions until analysis.

### 2.3. Laboratory Analyses

Circulating inflammatory and regulatory mediators were quantified in plasma samples to characterize systemic inflammatory and metabolic profiles associated with osteoarthritis phenotypes.

Pro-inflammatory and anti-inflammatory cytokines, including IL-1β (Canine IL-1 Beta DuoSet, R&D Systems, Minneapolis, MN, USA), IL-4 (Canine IL-4 DuoSet, R&D Systems, Minneapolis, MN, USA), and IL-10 (Canine IL-10 DuoSet, R&D Systems, Minneapolis, MN, USA), were measured using immunoassays validated for use in canine samples. All cytokine determinations were performed following the manufacturers’ instructions, and samples were analyzed in duplicate when applicable to ensure analytical reliability.

Routine biochemical analyses were conducted to assess liver function, renal status, energy metabolism, and lipid profile. Serum concentrations of ALT, AST, GGT, ALP, creatinine, urea, glucose, and total cholesterol were measured using standardized automated clinical chemistry methods routinely employed in veterinary diagnostic laboratories. Quality control procedures were applied according to laboratory standards to ensure analytical accuracy and precision.

To further explore metabolic regulation and joint tissue turnover, circulating adiponectin (ADP) (Dog ADP (Adiponectin), ELISA kit (Cat: ELK10713), ELK Biotechnology, Denver, CO, USA) concentrations were measured as a marker of adipose tissue-derived metabolic signaling, and collagen type II degradation was assessed by quantification of the C-terminal telopeptide of type II collagen (CTX-II) (Dog CTX-II (Cross Linked C-Telopeptide Of Type II Collagen), ELISA kit (Cat: ELK10021), ELK Biotechnology, Denver, CO, USA), a biomarker of cartilage breakdown. These analytes were determined using immunoassay-based methods validated for canine biological matrices.

All laboratory analyses were performed in accordance with established clinical pathology protocols or manufacturers’ recommendations. Samples from different study groups were processed under identical conditions to minimize analytical variability.

### 2.4. Statistical Analysis

Data analysis was performed using R software (version 4.5.2; R Foundation for Statistical Computing, Vienna, Austria). Linear modeling was conducted using the base stats package, estimated marginal means were obtained using the emmeans package, and graphical outputs were generated with ggplot2.

Continuous variables were initially explored using descriptive statistics and graphical inspection (histograms and boxplots) to evaluate their distributional properties. Most circulating mediator concentrations exhibited right-skewed or non-normal distributions and heteroscedasticity across groups. Therefore, all biomarker concentrations were natural log-transformed prior to inferential analyses to improve normality and variance homogeneity [37,38].

Group differences in biomarker concentrations were evaluated using linear models fitted to log-transformed outcomes. In the primary analysis, dogs were classified into three phenotypic groups: healthy controls (CG), TOA, and OOA. Phenotypic group and sex were included as fixed categorical predictors. Model coefficients were exponentiated and are reported as multiplicative effects (geometric mean ratios) with corresponding 95% confidence intervals, allowing direct interpretation of proportional differences between groups.

For biomarkers presenting zero or near-zero values, a log(Y + 1) transformation was applied to avoid undefined values. This transformation rule was applied consistently across all individual biomarkers and ratio-based outcomes to ensure comparability of effect estimates. Ratio variables were constructed as differences between log-transformed biomarkers, which is mathematically equivalent to modeling the logarithm of their ratios on the original scale. Zero or near-zero values were infrequent across biomarkers, affecting fewer than 5% of observations for most analytes, and were primarily limited to specific cytokines.

To assess the robustness of the primary findings, additional models were fitted including age and body weight as continuous covariates. A secondary sensitivity analysis was performed by collapsing dogs with osteoarthritis into a single group regardless of body condition and comparing them with healthy controls. Interaction terms were explored in exploratory analyses but were not retained in the final models due to limited sample size within strata and the resulting instability of interaction estimates.

In addition to individual biomarker analyses, integrative inflammatory–metabolic ratios were evaluated to explore the balance between systemic inflammation and metabolic alterations. Specifically, the ratios IL-1β:GGT, IL-1β:cholesterol, and IL-1β:ADP were analyzed using a harmonized log-ratio transformation defined as the difference between the natural logarithms of the individual biomarkers [log(IL-1β + 1) − log(GGT + 1), log(IL-1β + 1) − log(cholesterol + 1), and log(IL-1β + 1) − log(ADP + 1), respectively]. This approach preserves the interpretability of ratios while maintaining consistency with the transformation applied to individual biomarkers and reducing the influence of extreme values. Exponentiated coefficients from these models were interpreted as multiplicative effects on the corresponding inflammatory–metabolic ratios.

Model assumptions were evaluated through systematic inspection of residual diagnostics, including residuals versus fitted values, Q–Q plots, scale–location plots, and leverage diagnostics. No major violations of linear model assumptions or influential observations were identified. Representative diagnostic plots are provided in the Supplementary Material (Figures S1–S36). Adjusted R^2^ values were used to assess overall model fit.

Estimated marginal means and corresponding 95% confidence intervals on the original mediator scale (geometric means) were obtained from the fitted models and used for graphical representation [38]. Statistical significance was set at *p* < 0.05.

## 3. Results

### 3.1. Study Population Characteristics

A total of 60 client-owned dogs were included in the study and classified into three groups according to osteoarthritis status and body condition: CG (n = 19), TOA (n = 21), and OOA (n = 20). Both female and male dogs were represented in all groups, with an overall predominance of females (70%).

The mean age of the study population was 5.3 ± 2.7 years, with dogs diagnosed with OA tending to be older than healthy controls. Body weight differed according to body condition phenotype, with obese dogs with OA exhibiting higher body weight compared with thin dogs with OA and control dogs, as expected by study design. Sex distribution was comparable across groups, and no marked imbalance between females and males was observed.

Most dogs were mixed-breed animals, with a smaller representation of Labrador Retrievers, Golden Retrievers, and other breeds. Breed distribution was similar across study groups and was not considered a primary stratification factor in subsequent analyses. A total of 41/60 dogs (68.3%) were diagnosed with osteoarthritis. All OA cases (100%) exhibited hip joint involvement. In the TOA group (n = 21), hip-only involvement was observed in 66.7% of dogs, while 9.5% had concurrent hip and elbow involvement, 9.5% hip and stifle involvement, and 9.5% involvement of hip, stifle, and elbow joints. In the OOA group (n = 20), 40.0% showed isolated hip involvement, 25.0% had hip and stifle involvement, 20.0% had hip and elbow involvement, and 15.0% presented combined hip, stifle, and elbow disease.

Overall, elbow involvement was present in 17.1% and stifle involvement in 22.0% of OA dogs.

All dogs underwent clinical and laboratory screening prior to inclusion. Animals presenting evidence of systemic, endocrine, or metabolic diseases unrelated to osteoarthritis were excluded. Baseline hematological and biochemical parameters were within clinically acceptable ranges, supporting the inclusion of a systemically stable population aside from osteoarthritis-related changes.

Given the observed variability in age and body weight across groups, these variables were considered potential confounders and were included as covariates in adjusted statistical models. Summary characteristics of the study population are presented in Table 1.

### 3.2. Plasma Inflammatory and Regulatory Cytokines

Unless otherwise stated, model-based estimates are presented as exponentiated coefficients (geometric mean ratios) with 95% confidence intervals, derived from linear models fitted on log-transformed data. A ratio of 1 represents the null effect.

#### 3.2.1. IL-1β

In the primary linear model (m1) including group and sex as fixed effects, TOA dogs exhibited significantly higher IL-1β concentrations compared with healthy controls, corresponding to an approximately 39% increase in geometric mean IL-1β concentrations (Table 2, m1).

In contrast, no significant differences were observed between OOA dogs and controls. Sex was independently associated with IL-1β concentrations, with male dogs showing approximately 40% higher IL-1β concentrations than females (Table 2).

**Figure 1:**
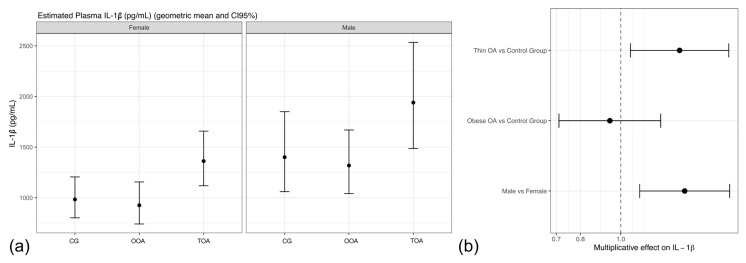
(**a**) Model-based geometric mean plasma IL-1β concentrations by group and sex with 95% confidence intervals. (**b**) Forest plot of multiplicative effects (expβ) for IL-1β concentrations comparing osteoarthritis phenotypes and sex relative to the control group. The vertical line denotes the null effect (ratio = 1). Acronyms as defined in Table 1.

Estimated marginal means derived from the fitted model and back-transformed to the original scale further illustrated these findings (Figure 1a). Elevated IL-1β concentrations in TOA dogs were evident in both sexes, whereas IL-1 β concentrations in OOA dogs remained comparable to controls. Sex-related differences were consistent across all groups.

Adjustment for age and body weight did not materially alter the direction or magnitude of the observed effects (Table 2, Model 2 (m2)). In sensitivity analyses where dogs with osteoarthritis were analyzed as a single group regardless of body condition, no significant differences in IL-1β concentrations were detected between osteoarthritis dogs and healthy controls, while the effect of sex remained significant (Table 2, Model 3 (m3)).

Forest plot visualization of model estimates confirmed that increased IL-1β concentrations were specifically associated with the TOA phenotype and male sex, whereas the confidence interval for OOA overlapped the null value, indicating the absence of a significant effect (Figure 1b).

#### 3.2.2. IL-4

Circulating IL-4 concentrations showed no clear group- or sex-related differences across the study population (Table 3, m1). Effect estimates for both thin and obese dogs with osteoarthritis were close to unity relative to healthy controls, with confidence intervals broadly overlapping, indicating the absence of statistically robust phenotypic contrasts. Sex was not associated with IL-4 concentrations, and male–female differences were small and imprecise.

**Figure 2:**
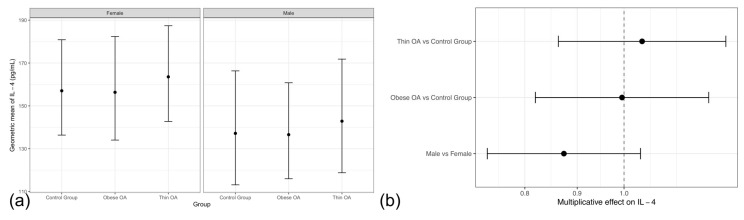
(**a**) Model-based geometric mean plasma IL-4 concentrations by study group and sex with 95% confidence intervals. (**b**) Forest plot of multiplicative effects (expβ) of osteoarthritis phenotype and sex on plasma IL-4 concentrations. The vertical line denotes the null effect (ratio = 1). Acronyms as defined in Figure 1.

These findings remained consistent after adjustment for age and body weight and in sensitivity analyses comparing dogs with osteoarthritis as a single group with healthy controls (Table 3, m2–m3). Model-based geometric mean estimates and forest plot visualization further supported the stability of IL-4 concentrations across osteoarthritis phenotypes and sexes, with only minor, non-significant elevations observed in thin dogs with osteoarthritis (Figure 2a,b).

#### 3.2.3. IL-10

**Figure 3:**
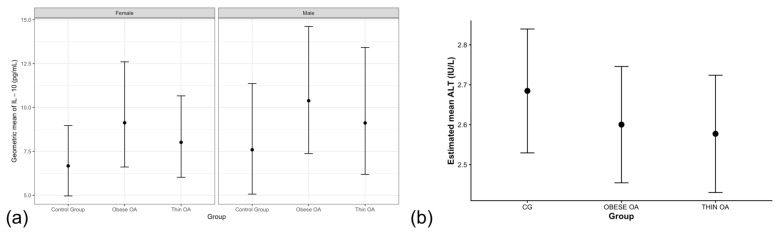
(**a**) Estimated marginal means (EMMs) of circulating IL-10 concentrations by osteoarthritis phenotype and sex. (**b**) EMMs of circulating ALT concentrations by osteoarthritis phenotype. Values represent model-based back-transformed geometric means with 95% confidence intervals. Acronyms as defined in Table 1.

Circulating IL-10 concentrations showed modest, non-significant elevations in dogs with osteoarthritis, with point estimates tending to be higher in obese individuals compared with healthy controls, while thin dogs exhibited smaller increases (Table 4; Figure 3a). Male dogs also showed numerically higher IL-10 concentrations than females; however, confidence intervals overlapped the null value. After adjustment, these patterns did not reach clear statistical significance, suggesting that systemic IL-10 variability may be influenced by factors beyond osteoarthritis status and gender.

### 3.3. Metabolic and Biochemical Parameters

#### 3.3.1. Liver-Associated Enzymes (ALT, AST, GGT, ALP)

- Serum ALT Concentrations

Serum ALT concentrations did not differ meaningfully between dogs with osteoarthritis and healthy controls.

**Figure 4:**
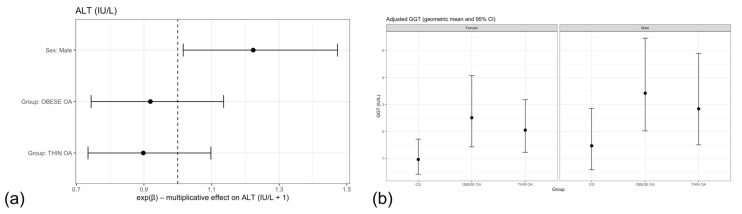
(**a**) Forest plot of multiplicative effects (expβ) for serum ALT concentrations. The vertical dashed line denotes the null effect (ratio = 1). (**b**) Adjusted geometric mean serum GGT concentrations by study group and sex with 95% confidence intervals. ALT, alanine aminotransferase; GGT, gamma-glutamyl transferase. Other acronyms as defined in Table 1.

Both thin and obese dogs with OA showed slightly lower geometric mean ALT values relative to controls, but confidence intervals broadly overlapped unity, indicating the absence of a robust group effect (Table 5, m1; Figure 3b). Male dogs exhibited higher ALT concentrations than females across models; however, effect sizes were modest and of limited biological relevance. Forest plot visualization confirmed that neither osteoarthritis phenotype nor sex exerted a strong independent effect on systemic ALT concentrations in this cohort (Figure 4a).

Adjustment for age and body weight did not materially alter these findings, and no significant associations with ALT concentrations were observed for age, body weight, or osteoarthritis status in adjusted or sensitivity analyses (Table 5, m2–m3).

- Serum GGT Concentrations

In the primary linear model including group and sex as fixed effects, dogs with osteoarthritis exhibited higher GGT concentrations compared with healthy controls. Specifically, dogs with obese osteoarthritis (OOA) showed a clear increase in geometric mean GGT concentrations relative to controls, while dogs with thin osteoarthritis (TOA) also demonstrated higher GGT levels, although with greater variability and a more modest effect size (Table 6, m1).

Estimated marginal means derived from the fitted model and back-transformed to the original scale illustrated these patterns (Figure 4b). Elevated GGT concentrations were observed across osteoarthritis phenotypes in both sexes, with adjusted geometric means tending to be higher in OOA dogs. Although male dogs showed numerically higher GGT concentrations, sex-related differences were small and confidence intervals largely overlapped across groups, indicating limited evidence for a strong independent sex effect.

Adjustment for age and body weight did not materially alter the direction of the association between osteoarthritis phenotype and GGT concentrations, although effect estimates were attenuated and no longer statistically significant in the fully adjusted model (Table 6, m2).

In sensitivity analyses in which dogs with osteoarthritis were analyzed as a single group regardless of body condition, GGT concentrations remained higher in osteoarthritis dogs compared with healthy controls, supporting a robust association between osteoarthritis status and systemic GGT levels (Table 6, m3).

**Figure 5:**
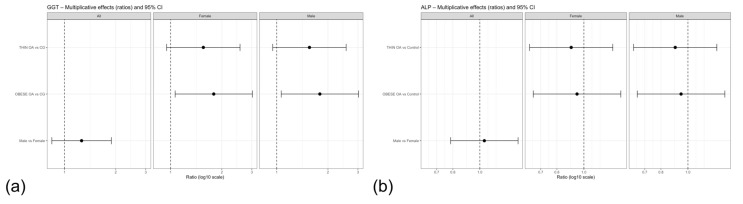
Forest plots of multiplicative effects (expβ) for serum GGT (**a**) and ALP (**b**) concentrations. Estimates compare thin and obese osteoarthritis phenotypes with the control group, and males with females, for the overall population and stratified by sex. The vertical dashed line denotes the null effect (ratio = 1). GGT, gamma-glutamyl transferase; ALP, alkaline phosphatase. Other acronyms as defined in Table 1.

Forest plot visualization of model estimates supported an overall trend toward increased GGT concentrations in dogs with osteoarthritis, particularly in the OOA phenotype (Figure 5a). In contrast, the effect of sex was comparatively weak, with confidence intervals for the male–female contrast overlapping the null value, suggesting that osteoarthritis phenotype rather than sex was the primary contributor to variation in GGT concentrations.

- Serum ALP Concentrations

Serum ALP concentrations were analyzed using the primary linear model including group and sex as fixed effects. No statistically significant differences in ALP concentrations were observed between obese or thin dogs with osteoarthritis and the control group. Similarly, sex was not associated with ALP concentrations (Table 7).

Forest plot visualization of exponentiated model estimates confirmed that all group- and sex-related effects overlapped the null value, indicating the absence of a meaningful association between osteoarthritis phenotype, sex, and systemic ALP levels (Figure 5b).

#### 3.3.2. Renal and Nitrogen Metabolism Markers (Creatinine, Urea)

- Serum Creatinine

**Figure 6:**
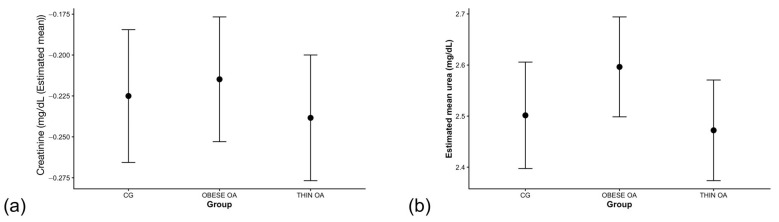
(**a**) Estimated marginal means (EMMs) of serum creatinine concentrations by study group. (**b**) EMMs of serum urea concentrations by study group. Values represent model-based back-transformed geometric means with 95% confidence intervals. Acronyms as defined in Table 1.

Serum creatinine concentrations did not differ between dogs with osteoarthritis and healthy controls. Effect estimates for both thin and obese osteoarthritis phenotypes were close to unity, with broadly overlapping confidence intervals, indicating the absence of a meaningful group effect (Table 8; Figure 6a). Sex was not associated with creatinine concentrations, with male–female contrasts remaining close to the null value.

- Serum urea

Serum urea concentrations did not differ meaningfully between obese dogs with osteoarthritis and healthy controls. Thin dogs with osteoarthritis showed a modest, non-significant decrease in urea concentrations relative to controls, with confidence intervals overlapping unity (Table 9; Figure 6b). Sex was not associated with urea concentrations, with male–female contrasts remaining close to the null value.

#### 3.3.3. Energy and Lipid Metabolism (Glucose, Cholesterol)

- Serum Glucose

Serum glucose concentrations were analyzed using the primary linear model including study group and sex as fixed effects. No statistically significant differences in serum glucose concentrations were observed between obese or thin dogs with osteoarthritis and the control group (Table 10). Sex was not associated with serum glucose levels.

**Figure 7:**
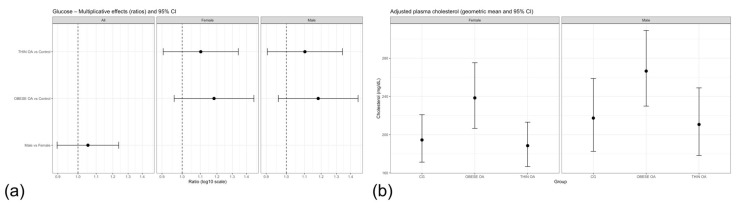
(**a**) Forest plot of multiplicative effects (expβ) for serum glucose concentrations comparing osteoarthritis phenotypes with the control group and males with females. The vertical dashed line denotes the null effect (ratio = 1). (**b**) Adjusted geometric mean serum cholesterol concentrations by study group and sex with 95% confidence intervals. Acronyms as defined in Table 1.

Although point estimates suggested slightly higher glucose levels in obese dogs with osteoarthritis compared with controls, the corresponding confidence intervals overlapped the null value. Sex was not associated with serum glucose concentrations. Forest plot visualization further confirmed the absence of a robust group- or sex-related effect on systemic glucose levels (Figure 7a).

- Serum Cholesterol

**Figure 8:**
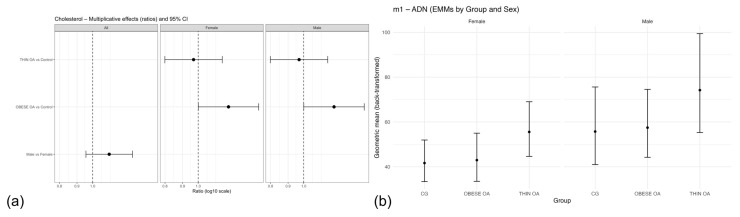
(**a**) Forest plot of multiplicative effects (expβ) for serum cholesterol concentrations comparing osteoarthritis phenotypes with the control group and males with females, shown for the overall population and stratified by sex. The vertical dashed line denotes the null effect (ratio = 1). (**b**) Estimated marginal means (EMMs) of plasma adiponectin (ADP) concentrations by study group and sex with 95% confidence intervals. Acronyms as defined in Table 1.

Serum cholesterol concentrations were modestly higher in obese dogs with osteoarthritis compared with healthy controls in the primary analysis, whereas thin dogs with osteoarthritis showed values comparable to controls (Table 11, m1; Figure 7b). Sex was not independently associated with cholesterol concentrations. After adjustment for age and body weight, the association between obesity-associated osteoarthritis and cholesterol levels was attenuated and no longer statistically significant (Table 11, m2). Consistently, when dogs with osteoarthritis were analyzed as a single group regardless of body condition, cholesterol concentrations did not differ from those of healthy controls (Table 11, m3). Forest plot visualization confirmed that the observed effect was driven by the obese osteoarthritis phenotype and diminished after adjustment or group reclassification (Figure 8a).

### 3.4. Adipokines and Cartilage Turnover Markers

#### 3.4.1. Plasma ADP

In the primary linear model including group and sex as fixed effects (Table 12, m1), circulating ADP concentrations differed according to osteoarthritis phenotype and sex.

TOA dogs showed higher adiponectin concentrations than healthy controls, although this difference did not reach conventional statistical significance (geometric mean ratio = 1.33, 95% CI: 0.99–1.77; *p* = 0.058). In contrast, OOA dogs did not differ from controls (ratio = 1.03, 95% CI: 0.76–1.40; *p* = 0.84). Male dogs exhibited significantly higher ADP concentrations than females (ratio = 1.33, 95% CI: 1.01–1.74; *p* = 0.039) (Figure 8b).

**Figure 9:**
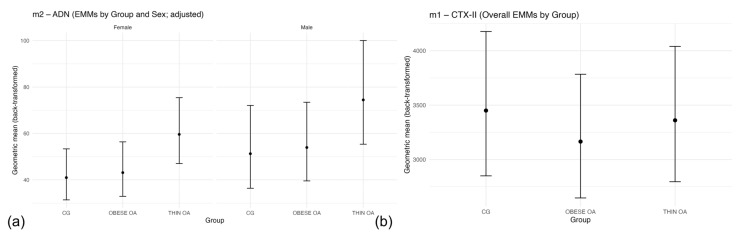
(**a**) Estimated marginal means (EMMs) of circulating adiponectin (ADP) concentrations by study group and sex after adjustment for age and body weight (model m2). (**b**) Overall EMMs of circulating CTX-II concentrations by study group (model m1), averaged over sex. Values represent model-based back-transformed geometric means with 95% confidence intervals. Acronyms as defined in Table 1.

After adjustment for age and body weight (Table 12, m2), ADP concentrations were significantly higher in TOA dogs compared with healthy controls (ratio = 1.44, 95% CI: 1.02–2.04; *p* = 0.037), whereas no differences were observed between OOA dogs and controls (ratio = 1.05, 95% CI: 0.71–1.55; *p* = 0.80). In this adjusted model, sex, age, and body weight were not independently associated with adiponectin concentrations (Figure 9a).

When dogs with OA were analyzed as a single group irrespective of body condition (Table 12, m3), adiponectin concentrations did not differ from those of healthy controls (ratio = 1.18, 95% CI: 0.91–1.53; *p* = 0.21). A non-significant tendency toward higher adiponectin concentrations in male dogs was observed in this model (ratio = 1.27, 95% CI: 0.97–1.66; *p* = 0.079).

#### 3.4.2. Plasma CTX-II

Circulating CTX-II concentrations showed no meaningful differences between dogs with osteoarthritis and healthy controls. Effect estimates for both thin and obese osteoarthritis phenotypes were close to unity, and male dogs exhibited only a non-significant tendency toward higher CTX-II concentrations than females (Table 13, m1; Figure 9b). These patterns remained unchanged after adjustment for age and body weight and in sensitivity analyses comparing dogs with osteoarthritis as a single group with controls (Table 13, m2–m3), indicating that systemic CTX-II concentrations were not associated with osteoarthritis status or phenotype in this cohort.

### 3.5. Ratio-Based Analysis of Inflammatory and Metabolic Biomarkers

#### 3.5.1. IL-1β:GGT Ratio

**Figure 10:**
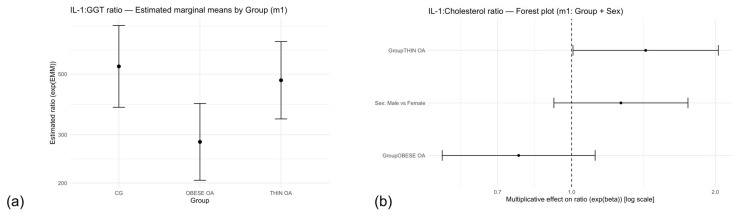
(**a**) Estimated marginal means (EMMs) of the IL-1β:GGT ratio by study group (model m1), adjusted for sex. The model was fitted to the log-transformed ratio [log(IL-1β + 1) − log(GGT + 1)]. Values represent model-based back-transformed geometric means with 95% confidence intervals. (**b**) Forest plot of multiplicative effects (expβ) for the IL-1β:GGT ratio comparing study groups with the control group and males with females. The vertical dashed line denotes the null effect (ratio = 1). Acronyms as defined in Table 1.

In the primary linear model including phenotypic group and sex as fixed effects (m1), significant group-related differences were observed for the IL-1β:GGT ratio (Table 14; Figure 10a). Dogs in the obese osteoarthritis (OOA) group exhibited a significantly lower IL-1β:GGT ratio compared with healthy controls (multiplicative effect = 0.53, 95% confidence interval: 0.33–0.85; *p* = 0.009). In contrast, no significant difference was detected between thin dogs with osteoarthritis (TOA) and controls. Sex was not associated with the IL-1β:GGT ratio in the primary model.

After additional adjustment for age and body weight (m2), the direction of the group-related effects remained consistent, although statistical significance was attenuated. In the sensitivity analysis comparing osteoarthritis dogs as a single group with controls (m3), osteoarthritis was associated with a lower IL-1β:GGT ratio (multiplicative effect = 0.51, 95% confidence interval: 0.12–0.89; *p* = 0.01), whereas sex again showed no significant effect.

Overall, these findings indicate that the IL-1β:GGT ratio identifies obese dogs with osteoarthritis as a subgroup with a lower inflammatory-to-metabolic balance relative to healthy controls, a pattern that is not evident when inflammatory markers are evaluated in isolation.

#### 3.5.2. IL-1β:Cholesterol Ratio

**Figure 11:**
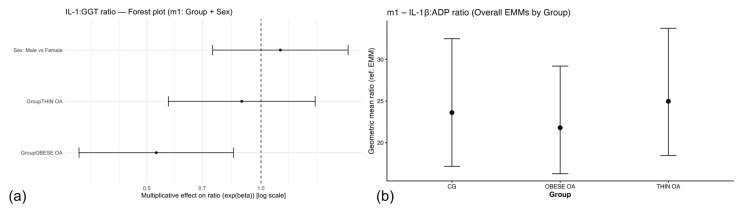
(**a**) Forest plot of multiplicative effects (expβ) for the IL-1β:cholesterol ratio from model m1, including phenotypic group (control group as reference) and sex (female as reference). The vertical dashed line denotes the null effect (ratio = 1). (**b**) Overall estimated marginal means (EMMs) of the IL-1β:adiponectin ratio by group (model m1: log(IL-1β + 1) − log(adiponectin + 1) ~ Group + Sex), averaged over sex. Values represent model-based back-transformed geometric mean ratios with 95% confidence intervals. Acronyms as defined in Table 1.

The IL-1β:cholesterol ratio showed clear phenotypic differentiation across study groups (Table 15, m1). Obese dogs with osteoarthritis exhibited a significantly lower ratio compared with healthy controls (multiplicative effect = 0.54, 95% CI: 0.33–0.85; *p* = 0.009), whereas no significant difference was observed for thin dogs with osteoarthritis. Sex did not contribute to variability in the IL-1β:cholesterol ratio. Forest plot visualization summarizes the magnitude and direction of these effects relative to the reference categories (Figure 11a), while group-level estimated marginal means further illustrate lower average ratios in obese osteoarthritis dogs, albeit with overlapping confidence intervals (Figure 11b). Taken together, these findings indicate differential inflammatory–metabolic profiles across osteoarthritis phenotypes, driven primarily by the obese osteoarthritis group.

#### 3.5.3. IL-1β:ADP Ratio

The IL-1β:ADP ratio showed no evidence of phenotypic differentiation across the study population. Effect estimates from the primary analysis indicated no association with osteoarthritis status or sex (Table 16, m1), and these null findings were consistently supported by adjusted and sensitivity models. Visualization of model-based estimates further confirmed the absence of group- or phenotype-related differences in the IL-1β:adiponectin ratio (Figure 11b).

## 4. Discussion

The present study was designed to explore whether naturally occurring OA in dogs is associated with uniform or heterogeneous systemic biomarker profiles when body condition is explicitly considered. By integrating inflammatory, metabolic, and biochemical mediators using a conservative modeling framework, our results provide insight into the extent to which systemic alterations in canine OA reflect distinct biological patterns rather than a single disease signature. The following sections interpret these findings in the context of emerging concepts of OA as a multifactorial and phenotypically diverse condition.

### 4.1. Osteoarthritis as a Phenotypically Heterogeneous Systemic Condition

The present study demonstrates that naturally occurring OA in dogs is not associated with a uniform systemic inflammatory or metabolic profile. Instead, circulating biomarker patterns varied substantially according to body condition, supporting the presence of phenotypic heterogeneity within canine OA. When dogs with OA were analyzed as a single group, most biomarkers showed limited discriminatory value, which contrasts with studies specifically designed to assess diagnostic discrimination between healthy and diseased dogs [32]. However, stratification into thin and obese OA groups revealed distinct and biologically plausible differences [39].

In the context of this study, the term phenotype is used to describe a reproducible pattern of systemic biomarker expression associated with body condition in dogs with OA, rather than a discrete clinical or mechanistic subtype. Defined in this way, the observed phenotypes highlight meaningful biological variability that would otherwise remain obscured in aggregate analyses [35].

These findings are consistent with emerging concepts in both human and veterinary OA research, in which OA is increasingly recognized as a heterogeneous syndrome encompassing inflammatory, metabolic, and mechanical dimensions rather than a single disease entity [40]. Together, our results indicate that body condition is a key determinant of systemic biomarker expression in canine OA and should be explicitly considered in future research designs as well as in the clinical interpretation of circulating biomarkers [35,36,41].

### 4.2. Systemic Inflammatory Mediators: Selective Rather than Global Activation

Among the inflammatory cytokines evaluated, IL-1β displayed the clearest phenotype-dependent pattern. Increased IL-1β concentrations were primarily observed in thin dogs with OA, whereas obese OA dogs did not show a comparable systemic elevation. This finding suggests that classical inflammatory signaling may be more evident in lean OA phenotypes, while obesity-associated OA may involve alternative or compartmentalized inflammatory pathways [40,42,43]. In canine OA, IL-1β has been consistently identified as a key pro-inflammatory mediator at the joint level, with increased expression reported in synovial membrane, cruciate ligament, and infrapatellar fat pad tissues, even in the absence of uniform systemic elevation [43,44]. These observations support the concept that IL-1β-mediated inflammatory activity in OA is predominantly localized within the joint environment, where it directly modulates chondrocyte metabolism and catabolic processes [42,45].

The preferential increase in IL-1β in TOA dogs suggests the presence of a distinct inflammatory-dominant systemic profile that is less frequently emphasized in the veterinary OA literature. While obesity-associated OA has been widely studied, comparatively limited attention has been given to lean individuals exhibiting heightened inflammatory signaling. In this context, increased systemic IL-1β concentrations may reflect inflammatory activation occurring independently of excess adiposity.

In contrast, the anti-inflammatory cytokines IL-4 and IL-10 did not differ significantly between groups. Although both mediators play established roles in immune regulation, cartilage homeostasis, and modulation of synovial inflammation, their circulating concentrations may not accurately reflect local immunological processes occurring within the osteoarthritic joint. In dogs, cytokine profiling studies have demonstrated that IL-4 expression is minimal or absent in OA synovial fluid, while IL-10 expression is detectable locally but shows limited discriminatory value when assessed systemically [43,44]. Furthermore, experimental and translational studies indicate that the biological activity of IL-4 and IL-10 in OA is largely dependent on local receptor expression and tissue-specific signaling within cartilage and synovium, rather than on sustained systemic availability [46]. These findings underscore the limitations of single time-point circulating measurements of anti-inflammatory cytokines, particularly in clinically stable animals.

The concurrent increase in adiponectin concentrations observed in TOA dogs further supports the existence of an inflammatory–metabolic dissociation within this phenotype. Although adiponectin is often regarded as metabolically protective, its role in inflammatory conditions is complex and sometimes paradoxical, potentially reflecting compensatory signaling or dysregulated metabolic–inflammatory cross-talk. Elevated adiponectin in lean OA dogs may therefore not indicate metabolic protection but rather an altered systemic regulatory state accompanying inflammatory activation.

Together, these results indicate that systemic cytokine profiling captures only a partial aspect of OA-related inflammation and that inflammatory activation in canine OA is selective rather than generalized. Rather than reflecting a global inflammatory state, circulating cytokine patterns appear to represent phenotype-specific and context-dependent expressions of joint pathology. This interpretation is consistent with emerging concepts of OA as a heterogeneous syndrome, in which inflammatory, metabolic, and mechanical components vary across phenotypes and are differentially expressed at the systemic versus local joint level [35,36,47].

One possible explanation for the TOA phenotype is a chronic catabolic state, potentially associated with reduced muscle mass or altered energy balance. Pain-related reductions in activity or appetite could also contribute to lower body weight in affected animals. Although detailed longitudinal data on disease duration, body composition, or standardized severity scores were not available for stratified analysis, OA involvement was bilateral in all affected dogs, and joint involvement frequently included more than one anatomical site. Future studies incorporating quantitative measures of muscle mass, clinical severity indices, and disease duration will be important to further delineate whether the TOA phenotype represents a stable biological subtype or a transitional stage within OA progression.

Taken together, these findings suggest that canine OA may encompass at least two systemic phenotypic patterns influenced by body condition: a predominantly inflammatory–catabolic profile in lean dogs and a metabolic–biochemical profile in obese dogs.

### 4.3. Hepatic and Metabolic Biomarkers: Systemic Patterns of GGT in Canine Osteoarthritis

Most conventional hepatic enzymes, including ALT, AST, and ALP, showed limited discriminatory value in relation to osteoarthritis. In contrast, GGT emerged as a systemic signal associated with OA across phenotypes. Importantly, circulating GGT concentrations in dogs with OA remained within established reference ranges for clinically healthy animals, indicating that these differences do not reflect overt biochemical abnormalities or clinically apparent hepatocellular disease. Based on the available literature, the association of subtle, within-range variations in GGT with naturally occurring canine OA, in the absence of parallel alterations in other hepatic enzymes, appears to represent a novel observation.

The dissociation between GGT and conventional markers of liver injury suggests that elevated GGT activity in dogs with OA is unlikely to indicate primary hepatic dysfunction. Rather, this finding is interpreted in light of evidence from other organ systems, in which GGT has been proposed as a marker of systemic oxidative stress, low-grade inflammation, and altered metabolic regulation, even among clinically asymptomatic individuals and in the absence of liver disease [48,49]. Supporting this interpretation, metabolic studies have demonstrated that GGT reflects biological pathways distinct from those captured by ALT or AST, underscoring its role as a systemic indicator rather than a liver-specific enzyme [50].

Accordingly, GGT may represent a sensitive systemic biomarker associated with osteoarthritis that captures subtle biological alterations not detected by conventional liver enzyme panels. While the underlying mechanisms linking GGT to osteoarthritis in dogs remain to be elucidated, this interpretation supports its relevance as an osteoarthritis-associated systemic signal rather than as a secondary consequence of obesity or hepatic dysfunction alone.

### 4.4. Glucose and Cholesterol: Metabolic Stability Despite Osteoarthritis

The relationship between conventional systemic metabolic markers and osteoarthritis is complex and appears to be shaped by both body condition and disease context. Although “metabolic osteoarthritis” has been increasingly discussed as a relevant framework in humans and translational models [51,52], routinely measured systemic markers such as glucose and total cholesterol may not consistently capture OA-related metabolic alterations. In fact, evidence in human cohorts suggests that impaired glucose metabolism is not invariably associated with OA outcomes [53], while experimental models indicate that metabolic disturbances (i.e., high-fat diet or diabetes) can modulate joint pathology primarily when metabolic stressors are present [54,55].

Similarly, lipid-related alterations linked to OA may be subtle or compartmentalized. In dogs with experimentally induced OA, conventional lipid profiles may show only limited or inconsistent short-term changes [56], whereas lipidomic studies demonstrate more distinct OA-associated shifts in specific lipid species—often more evident in synovial fluid than in serum [57] and detectable systemically mainly through advanced profiling approaches [58]. Together, these observations support the interpretation that conventional systemic metabolic markers (i.e., glucose, total cholesterol) may primarily reflect overall metabolic status and body condition rather than OA-specific biology, underscoring the value of integrative approaches when exploring metabolic–inflammatory interactions in osteoarthritis [59,60].

### 4.5. Ratio-Based Biomarkers Reveal Inflammatory–Metabolic Dissociation

One of the most informative aspects of this study was the exploratory use of ratio-based biomarkers integrating inflammatory and metabolic mediators. The IL-1β:GGT and IL-1β:cholesterol ratios revealed phenotypic patterns that were not apparent when individual analytes were considered independently, suggesting that composite measures may better capture the relative balance between interacting biological processes in osteoarthritis.

In OOA dogs, the IL-1β:cholesterol ratio was reduced, indicating a relative predominance of metabolic alterations over systemic inflammatory signaling. This interpretation is biologically plausible and consistent with experimental evidence from obesity-associated OA models, in which lipid metabolism and cholesterol-related pathways modulate inflammatory cascades and cartilage degradation, including IL-1β-mediated signaling [61]. Conversely, TOA dogs tended to exhibit higher inflammatory-to-metabolic ratios, consistent with a phenotype in which classical inflammatory signaling may play a more prominent systemic role in the absence of overt metabolic dysregulation.

Importantly, ratio-based biomarkers do not imply independent causality of individual mediators. Rather, they provide an integrated representation of inflammatory–metabolic dominance, offering improved biological interpretability in heterogeneous conditions such as naturally occurring osteoarthritis. While ratio-based approaches have previously been explored in osteoarthritis using hematological indices, such as the neutrophil-to-lymphocyte ratio in human studies [62], the application of simple biochemical inflammatory–metabolic ratios to naturally occurring canine osteoarthritis represents a novel exploratory approach.

### 4.6. Adiponectin and CTX-II: Limited Systemic Sensitivity in Cross-Sectional Analysis

Circulating adiponectin concentrations showed limited capacity to discriminate between OA phenotypes and healthy controls. Although adiponectin plays an important role in metabolic and inflammatory regulation, its systemic behavior in OA is highly context-dependent, with inconsistent associations reported across studies and biological compartments [63,64,65]. Experimental and translational evidence suggests that adiponectin exerts much of its biological activity locally within the joint environment, particularly in synovial fluid and periarticular adipose tissues, where both catabolic and regulatory effects have been described [66,67]. This dual and compartment-specific activity may partially explain why circulating adiponectin did not exhibit a clear phenotypic pattern in the present cohort. Consequently, single time-point circulating measurements may inadequately reflect its joint-specific relevance in naturally occurring disease.

Similarly, circulating CTX-II concentrations did not clearly discriminate between OA phenotypes and controls. While CTX-II is a well-established marker of cartilage degradation and correlates with radiographic severity and disease progression in longitudinal and experimental settings [68,69,70], its systemic sensitivity appears limited in cross-sectional assessments. In addition, serum CTX-II concentrations may be influenced by extra-articular collagen turnover and systemic metabolic factors, further reducing specificity for joint-level pathology. Prior studies indicate that CTX-II is more informative when evaluated longitudinally or in urine or synovial fluid, rather than as a single serum measurement [71,72].

Overall, the absence of strong systemic adiponectin and CTX-II signals in this study highlights the limitations of single time-point circulating biomarkers for capturing dynamic and localized joint tissue turnover. Rather than contradicting their established biological relevance, these findings suggest that their discriminatory value may depend on sampling compartment, disease stage, and temporal assessment. These findings do not negate the biological relevance of either marker but support their preferential use in longitudinal designs or local joint assessments when studying OA phenotypes in dogs.

### 4.7. Strengths, Limitations, and Implications

A major strength of the present study is the evaluation of naturally occurring OA in client-owned dogs, which captures the biological and clinical heterogeneity encountered in real-world veterinary practice. In contrast to experimental or surgically induced models, this approach reflects the complexity of spontaneous disease, in which inflammatory, metabolic, and biomechanical processes coexist and vary across individuals. In addition, the use of a conservative statistical framework and the deliberate avoidance of overinterpretation of isolated statistically significant findings enhance the robustness and transparency of the results.

Several limitations inherent to both the present study and to much of the existing literature on systemic biomarkers in canine osteoarthritis should nevertheless be acknowledged. First, the cross-sectional design precludes causal inference and limits the ability to capture temporal dynamics in inflammatory, metabolic, and cartilage turnover markers. Many biomarkers implicated in OA, including cytokines, adipokines, and collagen degradation products, exhibit time-dependent and activity-dependent fluctuations that may not be adequately reflected by single time-point systemic measurements. Similar limitations of cross-sectional designs have been explicitly or implicitly acknowledged in previous OA studies, particularly for biomarkers such as CTX-II and adiponectin, whose discriminatory value improves substantially in longitudinal settings or when local joint compartments are assessed [63,64,71].

Second, the absence of local joint-level measurements, such as synovial fluid or tissue biomarkers, limits interpretation of the relationship between systemic signals and intra-articular pathology. Accumulating evidence indicates that several mediators with established biological relevance in OA exert predominantly local effects within the joint microenvironment, while their circulating concentrations represent attenuated or buffered signals. This compartmentalization likely contributes to the inconsistent performance of systemic biomarkers across studies and species, particularly in naturally occurring disease.

An additional limitation is the modest sample size within each phenotypic subgroup. Although the statistical models employed were appropriate for the study design, the available sample size constrained statistical power to detect small-to-moderate effects, especially for biomarkers characterized by high biological variability and for potential interaction effects involving sex. Consequently, the analyses were primarily sensitive to moderate-to-large effects, and smaller but potentially meaningful differences may have remained undetected and should be interpreted with caution. This limitation is common in studies of naturally occurring canine OA and reflects the practical challenges of recruiting well-characterized clinical populations.

In this context, findings associated with *p*-values approaching conventional thresholds (e.g., 0.05–0.10), particularly those arising from stratified or subgroup analyses, should be interpreted cautiously. Such results may reflect limited statistical power rather than definitive absence or presence of biological effects and therefore require confirmation in larger cohorts.

Given the number of biomarkers and comparisons performed, the present analyses should be considered exploratory and hypothesis-generating. No formal correction for multiple testing was applied, and *p*-values—particularly those close to conventional significance thresholds—should therefore be interpreted cautiously. Emphasis was placed on consistency of effect direction across related models and on biological plausibility rather than on isolated statistically significant findings. Importantly, statistically significant results were generally coherent across complementary analyses, reducing the likelihood that the observed patterns represent spurious associations.

Although sex was included as a fixed effect in the statistical models, and differences were observed for IL-1β and adiponectin concentrations, the study was not powered to formally evaluate phenotype-by-sex interactions. Sex-related differences in these biomarkers may reflect hormonal modulation of immune and metabolic pathways [73,74]. Sex steroids are known to influence cytokine production and adipokine signaling, potentially contributing to differential systemic inflammatory responses [75]. Behavioral factors, including activity level or pain-related adaptations, may also influence systemic biomarker profiles. However, the present study was not designed to mechanistically evaluate these pathways, and such interpretations remain speculative.

Despite these limitations, the present findings provide evidence that systemic inflammatory and metabolic alterations in canine OA are phenotype-dependent rather than uniform. The use of integrative ratio-based biomarkers highlights inflammatory–metabolic dissociation between phenotypes and illustrates how composite measures may reveal biologically meaningful patterns that are not apparent when individual biomarkers are evaluated in isolation. More broadly, these results support a shift toward phenotype-oriented, longitudinal, and multi-compartment approaches in future OA research, rather than reliance on single systemic biomarkers as universal indicators of disease presence or severity.

## 5. Conclusions

This study demonstrates that naturally occurring canine OA is not characterized by a single systemic inflammatory or metabolic signature, but rather by distinct biomarker profiles that vary according to body condition. Rather than identifying universal circulating biomarkers of OA, our findings emphasize phenotypic heterogeneity and support the use of integrative biomarker approaches to capture biologically meaningful variability within affected dogs. These results suggest that body condition is a key determinant of systemic biomarker expression in canine OA and should be explicitly considered in both research design and biomarker interpretation.

From a clinical perspective, these results highlight that dogs with OA should not be interpreted under a uniform inflammatory paradigm. Lean dogs with OA may exhibit a relatively more inflammation-oriented systemic profile, whereas obese dogs may present a predominantly metabolic-associated pattern. Therefore, body condition should be explicitly considered when interpreting laboratory findings and when designing management strategies, including weight control programs, metabolic monitoring, and individualized anti-inflammatory approaches.

Future studies incorporating longitudinal designs, local joint assessments, and independent cohorts are warranted to further define the clinical and translational relevance of these systemic signatures.

## Figures and Tables

**Table 1:** Study population characteristics according to osteoarthritis status and body condition.

Characteristic	CG (n = 19)	TOA (n = 21)	OOA (n = 20)	Total (n = 60)
Age (years), mean ± SD	2.95 ± 0.97	6.2 ± 2.63	6.6 ± 2.50	5.3 ± 2.7
Body weight (kg), mean ± SD	25.63 ± 3.75	26.74 ± 7.91	34.00 ± 6.04	28.81 ± 7.15
Sex, n (%)				
-Female	15 (78.9)	16 (76.2)	11 (55)	42 (70)
-Male	4 (21.1)	5 (23.8)	9 (45)	18 (30)
Body condition score (9-point scale), median (IQR)	5 (0)	4 (0)	8 (1)	5 (3)
Mixed-breed dogs, n (%)	17 (89.50)	12 (57.10)	11 (55)	40 (66.70)

CG, control group; TOA, thin osteoarthritis group; OOA, obese osteoarthritis group; SD, standard deviation; IQR, interquartile range.

**Table 2:** Linear model results for plasma interleukin-1 beta (IL-1β).

Term	Estimate	SE	Statistic	*p*-Value	95% CI (Lower)	95% CI (Upper)
Model 1 (m1)						
(Intercept)	983.04	0.10	67.62	<0.001	801.53	1205.66
Group OOA	0.94	0.14	−0.43	0.67	0.71	1.25
Group TOA	1.39	0.14	2.40	0.02	1.05	1.82
Sex Male	1.42	0.12	2.85	0.01	1.11	1.83
Model 2 (m2)						
(Intercept)	769.68	0.26	25.11	<0.001	452.72	1308.57
Group OOA	0.83	0.18	−1.05	0.30	0.57	1.19
Group TOA	1.29	0.16	1.57	0.12	0.93	1.78
Sex Male	1.39	0.13	2.50	0.02	1.07	1.82
Age	1.02	0.03	0.76	0.45	0.97	1.08
Weight	1.01	0.01	0.76	0.45	0.99	1.03
Model 3 (m3)						
(Intercept)	997.17	0.11	64.03	<0.001	803.50	1237.51
OA vs. Control	1.16	0.13	1.15	0.26	0.90	1.49
Sex Male	1.33	0.13	2.21	0.03	1.03	1.72

Values were analyzed using linear models fitted on log-transformed data and are reported as exp(β) (geometric mean ratios) with 95% confidence intervals, relative to the reference categories (Control group and Female). N: 60. OA, Multiplicative effects (geometric mean ratios) represent proportional differences relative to the reference category; for example, a ratio of 1.39 corresponds to a 39% higher geometric mean concentration. Acronyms as in Table 1.

**Table 3:** Linear model results for plasma IL-4.

Term	Estimate	SE	Statistic	*p*-Value	95% CI (Lower)	95% CI (Upper)
(Intercept)	157.05	0.07	71.74	<0.001	136.37	180.86
m1						
Group OOA	1.00	0.10	−0.05	0.96	0.82	1.21
Group TOA	1.04	0.09	0.43	0.67	0.86	1.26
Sex Male	0.87	0.09	−1.57	0.12	0.74	1.04
m2						
Group OOA	0.97	0.13	−0.24	0.81	0.75	1.25
Group TOA	1.00	0.11	−0.03	0.97	0.80	1.25
Sex Male	0.89	0.09	−1.22	0.23	0.74	1.07
Age	1.02	0.02	0.81	0.42	0.98	1.05
Body Weight	0.99	0.01	−0.59	0.55	0.98	1.00
m3						
OA vs. CG	1.02	0.08	0.24	0.81	0.86	1.20
Sex Male	0.87	0.08	−1.71	0.09	0.73	1.02

Values were analyzed using linear models fitted on log-transformed data and are reported as exp(β) (geometric mean ratios) with 95% confidence intervals, relative to the reference categories (Control group and Female). N: 60. Acronyms as in Table 1.

**Table 4:** Linear model (m1) results for plasma IL-10.

Term	Estimate	SE	Statistic	*p*-Value	95% CI (Lower)	95% CI (Upper)
(Intercept)	6.67	0.15	12.86	<0.001	4.97	8.97
Group OOA	1.37	0.20	1.54	0.13	0.91	2.06
Group TOA	1.20	0.20	0.93	0.36	0.81	1.78
Sex Male	1.14	0.18	0.71	0.48	0.79	1.63

Values were analyzed using linear models fitted on log-transformed data and are reported as exp(β) (geometric mean ratios) with 95% confidence intervals, relative to the reference categories (Control group and Female). N: 60. Acronyms as in Table 1.

**Table 5:** Linear model results for serum ALT.

Term	Estimate	SE	Statistic	*p*-Value	95% CI (Lower)	95% CI (Upper)
m1						
(Intercept)	36.01	0.08	47.65	<0.001	30.97	41.87
Group OOA	0.92	0.11	−0.80	0.43	0.74	1.14
Group TOA	0.90	0.10	−1.07	0.29	0.73	1.10
Sex Male	1.22	0.09	2.18	0.03	1.02	1.47
m2						
(Intercept)	37.34	0.20	18.38	<0.001	25.16	55.43
Group OOA	0.90	0.14	−0.78	0.44	0.68	1.18
Group TOA	0.87	0.12	−1.18	0.24	0.68	1.10
Sex Male	1.24	0.10	2.20	0.03	1.02	1.52
Age	1.01	0.02	0.58	0.57	0.97	1.05
Weight	1.00	0.01	−0.39	0.70	0.98	1.01
m3						
(Intercept)	35.98	0.07	48.12	<0.001	30.99	41.77
OA vs. Control	0.91	0.09	−1.10	0.28	0.76	1.08
Sex Male	1.23	0.09	2.30	0.03	1.03	1.47

Values were analyzed using linear models fitted on log-transformed data and are reported as exp(β) (geometric mean ratios) with 95% confidence intervals, relative to the reference categories (Control group and Female). N: 60. Acronyms as in Table 1.

**Table 6:** Linear model results for serum GGT.

Term	Estimate	SE	Statistic	*p*-Value	95% CI (Lower)	95% CI (Upper)
m1						
(Intercept)	1.96	0.16	4.12	<0.001	1.41	2.72
Group OOA	1.79	0.23	2.55	0.014	1.13	2.84
Group TOA	1.56	0.22	2.04	0.047	1.01	2.41
Sex Male	1.26	0.20	1.15	0.255	0.84	1.88
m2						
(Intercept)	1.47	0.43	0.91	0.367	0.63	3.46
Group OOA	1.53	0.30	1.44	0.157	0.84	2.79
Group TOA	1.43	0.26	1.37	0.177	0.85	2.40
Sex Male	1.23	0.22	0.96	0.343	0.80	1.89
Age	1.02	0.04	0.56	0.575	0.94	1.12
Weight	1.01	0.02	0.54	0.591	0.98	1.04
m3						
(Intercept)	1.95	0.16	4.11	<0.001	1.41	2.69
OA vs. Control	1.66	0.19	2.63	0.011	1.13	2.44
Sex Male	1.29	0.20	1.32	0.192	0.88	1.91

Values were analyzed using linear models fitted on log-transformed data and are reported as exp(β) (geometric mean ratios) with 95% confidence intervals, relative to the reference categories (Control group and Female). N: 60. Acronyms as in Table 1.

**Table 7:** Linear model (m1) results for serum ALP.

Term	Estimate	SE	Statistic	*p*-Value	95% CI (Lower)	95% CI (Upper)
(Intercept)	100.44	0.11	41.41	<0.001	80.08	125.98
Group OOA	0.94	0.16	−0.35	0.73	0.69	1.30
Group TOA	0.90	0.15	−0.70	0.49	0.67	1.22
Sex Male	1.04	0.14	0.26	0.79	0.79	1.37

Values were analyzed using linear models fitted on log-transformed data and are reported as exp(β) (geometric mean ratios) with 95% confidence intervals, relative to the reference categories (Control group and Female). N: 60. Acronyms as in Table 1.

**Table 8:** Linear model (m1) results for serum creatinine.

Term	Estimate	SE	Statistic	*p*-Value	95% CI (Lower)	95% CI (Upper)
(Intercept)	2.13	0.02	38.54	<0.001	2.05	2.22
Group OOA	1.01	0.03	0.37	0.71	0.96	1.07
Group TOA	0.99	0.03	−0.51	0.61	0.94	1.04
Sex (Male)	1.03	0.02	1.39	0.17	0.99	1.09

Values were analyzed using linear models fitted on log-transformed data and are reported as exp(β) (geometric mean ratios) with 95% confidence intervals, relative to the reference categories (Control group and Female). N: 60. Acronyms as in Table 1.

**Table 9:** Linear model (m1) results for serum urea.

Term	Estimate	SE	Statistic	*p*-Value	95% CI (Lower)	95% CI (Upper)
(Intercept)	33.17	0.05	69.34	<0.001	29.98	36.70
Group OOA	1.10	0.07	1.34	0.19	0.95	1.27
Group TOA	0.97	0.07	−0.44	0.66	0.85	1.11
Sex Male	1.00	0.06	−0.00	0.99	0.88	1.13

Values were analyzed using linear models fitted on log-transformed data and are reported as exp(β) (geometric mean ratios) with 95% confidence intervals, relative to the reference categories (Control group and Female). N: 60. Acronyms as in Table 1.

**Table 10:** Linear model (m1) results for serum glucose.

Term	Estimate	SE	Statistic	*p*-Value	95% CI (Lower)	95% CI (Upper)
(Intercept)	4.33	0.06	67.64	<0.001	4.20	4.45
Group OOA	1.19	0.09	1.83	0.07	0.98	1.42
Group TOA	1.10	0.09	1.13	0.27	0.93	1.31
Sex Male	1.05	0.08	0.64	0.52	0.90	1.24

Values were analyzed using linear models fitted on log-transformed data and are reported as exp(β) (geometric mean ratios) with 95% confidence intervals, relative to the reference categories (Control group and Female). N: 60. Acronyms as in Table 1.

**Table 11:** Linear model results for serum cholesterol.

Term	Estimate	SE	Statistic	*p*-Value	95% CI (Lower)	95% CI (Upper)
m1						
(Intercept)	195.51	0.06	83.54	<0.001	172.26	221.88
Group OOA	1.23	0.09	2.29	0.026	1.03	1.46
Group TOA	0.97	0.08	−0.37	0.714	0.82	1.15
Sex Male	1.12	0.08	1.43	0.160	0.96	1.31
m2						
(Intercept)	158.36	0.16	31.06	<0.001	114.18	219.62
Group OOA	1.14	0.11	1.12	0.267	0.90	1.43
Group TOA	0.95	0.10	−0.53	0.596	0.78	1.16
Sex Male	1.08	0.08	0.96	0.341	0.92	1.28
Age	1.00	0.02	0.26	0.793	0.97	1.04
Weight	1.01	0.01	1.33	0.191	1.00	1.02
m3						
(Intercept)	193.67	0.07	79.13	<0.001	169.49	221.29
OA vs. Control	1.08	0.08	0.94	0.354	0.92	1.26
Sex Male	1.17	0.08	1.94	0.057	1.00	1.37

Values were analyzed using linear models fitted on log-transformed data and are reported as exp(β) (geometric mean ratios) with 95% confidence intervals, relative to the reference categories (Control group and Female). N: 60. Acronyms like in Table 1.

**Table 12:** Linear model results for plasma ADP.

Term	Estimate	SE	Statistic	*p*-Value	95% CI (Lower)	95% CI (Upper)
(Intercept)	42.67	0.11	34.85	<0.001	34.40	52.95
m1						
Group OOA	1.03	0.15	0.20	0.84	0.76	1.40
Group TOA	1.33	0.15	1.93	0.058	0.99	1.77
Sex (Male)	1.33	0.13	2.11	0.039	1.01	1.74
m2						
Group OOA	1.05	0.19	0.26	0.80	0.71	1.55
Group TOA	1.44	0.17	2.14	0.037	1.02	2.04
Sex Male	1.25	0.14	1.54	0.13	0.94	1.66
Age	1.02	0.03	0.81	0.42	0.92	1.03
Body Weight	1.01	0.01	−0.59	0.55	0.99	1.03
m3						
OA vs. CG	1.18	0.13	1.26	0.21	0.91	1.53
Sex (Male)	1.27	0.13	1.79	0.079	0.97	1.66

Values were analyzed using linear models fitted on log-transformed data and are reported as exp(β) (geometric mean ratios) with 95% confidence intervals, relative to the reference categories (Control group and Female). N: 60. Acronyms as in Table 1.

**Table 13:** Linear model results for plasma CTX-II.

Term	Estimate	SE	Statistic	*p*-Value	95% CI (Lower)	95% CI (Upper)
(Intercept)	3118.95	0.09	87.56	<0.001	2594.79	3749.00
m1						
Group OOA	0.92	0.13	−0.67	0.51	0.71	1.19
Group TOA	0.97	0.12	−0.21	0.83	0.76	1.25
Sex (Male)	1.22	0.12	1.76	0.084	0.97	1.54
m2						
Group OOA	0.98	0.17	−0.11	0.91	0.70	1.37
Group TOA	1.06	0.15	0.37	0.71	0.78	1.42
Sex (Male)	1.19	0.12	1.42	0.16	0.93	1.52
Age	1.00	0.01	−0.08	0.94	0.98	1.02
Body Weight	1.00	0.01	0.31	0.76	0.99	1.02
m3						
OA vs. CG	0.95	0.11	−0.50	0.62	0.76	1.18
Sex (Male)	1.21	0.11	1.71	0.093	0.97	1.52

Values were analyzed using linear models fitted on log-transformed data and are reported as exp(β) (geometric mean ratios) with 95% confidence intervals, relative to the reference categories (Control group and Female). N: 60. Acronyms as in Table 1.

**Table 14:** Linear model results for the plasma IL-1β:GGT ratio.

Term	Estimate	SE	Statistic	*p*-Value	95% CI (Lower)	95% CI (Upper)
m1						
(Intercept)	503.14	0.17	NA	<0.001	360.19	702.82
Group OOA	0.53	0.23	−2.64	0.009	0.33	0.85
Group TOA	0.89	0.23	−0.52	0.60	0.57	1.39
Sex Male	1.12	0.21	0.57	0.57	0.75	1.70
m2						
(Intercept)	394.71	0.44	NA	0.37	241.00	646.92
Group OOA	0.43	0.30	−1.44	0.16	0.17	1.03
Group TOA	0.36	0.26	−1.37	0.18	0.17	0.88
Sex Male	1.21	0.22	0.96	0.34	0.77	1.64
Age	1.02	0.04	0.56	0.57	0.94	1.11
Body Weight	1.01	0.02	0.54	0.59	0.98	1.04
m3						
(Intercept)	503.41	0.16	NA	<0.001	359.88	703.44
OA vs. Control	0.51	0.19	−2.63	0.01	0.12	0.89
Sex Male	1.26	0.20	1.32	0.19	0.87	1.65

Outcome was defined as log(IL-1β + 1) − log(GGT + 1). Estimates are reported as exponentiated coefficients, representing multiplicative effects on the IL-1β:GGT ratio. Values < 1 indicate a lower ratio relative to the reference category. The constant +1 was added to allow log-transformation of zero values. NA, Test statistic not applicable for intercept in ratio models. Acronyms as in Table 1.

**Table 15:** Linear model (m1) results for the plasma IL-1β:cholesterol ratio.

Term	Estimate	SE	Statistic	*p*-Value	95% CI (Lower)	95% CI (Upper)
(Intercept)	521.84	0.11	54.21	<0.001	420.36	648.79
Group OOA	0.54	0.14	−2.67	0.009	0.33	0.85
Group TOA	0.89	0.15	−0.53	0.60	0.57	1.39
Sex Male	1.12	0.13	0.56	0.57	0.75	1.70

Outcome was defined as log(IL-1β + 1) − log(cholesterol + 1). Estimates are reported as exponentiated coefficients, representing multiplicative effects on the IL-1β:cholesterol ratio. Values < 1 indicate a lower ratio relative to the reference category. The constant +1 was added to allow log-transformation of zero values. Acronyms as in Table 1.

**Table 16:** Linear model (m1) results for the plasma IL-1β:ADP ratio.

Term	Estimate	SE	Statistic	*p*-Value	95% CI (Lower)	95% CI (Upper)
(Intercept)	22.78	0.15	20.20	<0.001	16.71	31.05
Group OOA	0.92	0.21	−0.37	0.710	0.60	1.42
Group TOA	1.06	0.21	0.27	0.789	0.70	1.60
Sex (Male)	1.08	0.19	0.39	0.701	0.74	1.57

Outcome was defined as log(IL-1β + 1) − log(adiponectin + 1). Estimates are reported as exponentiated coefficients, representing multiplicative effects on the IL-1β:adiponectin ratio. Values < 1 indicate a lower ratio relative to the reference category. The constant +1 was added to allow log-transformation of zero values. Acronyms as in Table 1.

## Data Availability

The raw data supporting the conclusions of this article will be made available by the authors on request.

## Supplementary Materials

The following supporting information can be downloaded at: https://www.mdpi.com/article/10.3390/ani16040692/s1, Informed Consent Statement (ICS). Figures S1–S36. Representative diagnostic plots.

## Abbreviations

The following abbreviations are used in this manuscript:

ADP	Adiponectin
ALP	Alkaline phosphatase
ALT	Alanine aminotransferase
AST	Aspartate aminotransferase
CG	Control group
CI	Confidence interval
CTX-II	C-terminal telopeptide of type II collagen
EMM	Estimated marginal mean
EMMs	Estimated marginal means
GGT	Gamma-glutamyl transferase
IL-1β	Interleukin-1 beta
IL-4	Interleukin-4
IL-10	Interleukin-10
LPS	Lipopolysaccharide
m1	Primary linear model (Model 1)
m2	Adjusted linear model (including age and body weight (Model 2))
m3	Linear model comparing osteoarthritis vs. control (Model 3)
OA	Osteoarthritis
OOA	Obese dogs with osteoarthritis
TOA	Thin dogs with osteoarthritis

## References

[1] Anderson K.L., O’Neill D.G., Brodbelt D.C., Church D.B., Meeson R.L., Sargan D., Summers J.F., Zulch H., Collins L.M. (2018). Prevalence, Duration and Risk Factors for Appendicular Osteoarthritis in a UK Dog Population under Primary Veterinary Care. Sci. Rep..

[2] Anderson K.L., Zulch H., O’Neill D.G., Meeson R.L., Collins L.M. (2020). Risk Factors for Canine Osteoarthritis and Its Predisposing Arthropathies: A Systematic Review. Front. Vet. Sci..

[3] Cimino Brown D. (2017). What Can We Learn from Osteoarthritis Pain in Companion Animals?. Clin. Exp. Rheumatol..

[4] Sandell L.J. (2012). Etiology of Osteoarthritis: Genetics and Synovial Joint Development. Nat. Rev. Rheumatol..

[5] Renberg W.C. (2005). Pathophysiology and Management of Arthritis. Vet. Clin. N. Am. Small Anim. Pract..

[6] Beerts C., Broeckx S.Y., Depuydt E., Tack L., Van Hecke L., Chiers K., Van Brantegem L., Braun G., Hellmann K., de Bouvre N. (2023). Low-Dose Xenogeneic Mesenchymal Stem Cells Target Canine Osteoarthritis through Systemic Immunomodulation and Homing. Arthritis Res. Ther..

[7] Marshall W., Bockstahler B., Hulse D., Carmichael S. (2009). A Review of Osteoarthritis and Obesity: Current Understanding of the Relationship and Benefit of Obesity Treatment and Prevention in the Dog. Vet. Comp. Orthop. Traumatol..

[8] German A.J., Ryan V.H., German A.C., Wood I.S., Trayhurn P. (2010). Obesity, Its Associated Disorders and the Role of Inflammatory Adipokines in Companion Animals. Vet. J..

[9] Sanderson S.L. (2012). The Epidemic of Canine Obesity and Its Role in Osteoarthritis. Isr. J. Vet. Med..

[10] Belshaw Z., Dean R., Asher L. (2020). Could It Be Osteoarthritis? How Dog Owners and Veterinary Surgeons Describe Identifying Canine Osteoarthritis in a General Practice Setting. Prev. Vet. Med..

[11] Binvignat M., Sellam J., Berenbaum F., Felson D.T. (2024). The Role of Obesity and Adipose Tissue Dysfunction in Osteoarthritis Pain. Nat. Rev. Rheumatol..

[12] Chow Y.Y., Chin K.-Y. (2020). The Role of Inflammation in the Pathogenesis of Osteoarthritis. Mediat. Inflamm..

[13] Czerewaty M., Łączna M., Kiełbowski K., Bakinowska E., Dec P., Modrzejewski A., Kotrych D., Burszewski P., Safranow K., Pawlik A. (2024). The Effect of Plasma Cytokines on the Expression of Adiponectin and Its Receptors in the Synovial Membrane of Joints and the Infrapatellar Fat Pad in Patients with Rheumatoid Arthritis and Osteoarthritis. Prostaglandins Other Lipid Mediat..

[14] De Roover A., Escribano-Núñez A., Monteagudo S., Lories R. (2023). Fundamentals of Osteoarthritis: Inflammatory Mediators in Osteoarthritis. Osteoarthr. Cartil..

[15] Ait Eldjoudi D., Cordero Barreal A., Gonzalez-Rodríguez M., Ruiz-Fernández C., Farrag Y., Farrag M., Lago F., Capuozzo M., Gonzalez-Gay M.A., Mera Varela A. (2022). Leptin in Osteoarthritis and Rheumatoid Arthritis: Player or Bystander?. Int. J. Mol. Sci..

[16] Mobasheri A., Batt M. (2016). An Update on the Pathophysiology of Osteoarthritis. Ann. Phys. Rehabil. Med..

[17] Carmona J.U., Prades M. (2009). Pathophysiology of Osteoarthritis. Compend. Equine.

[18] Goldring M.B., Goldring S.R. (2007). Osteoarthritis. J. Cell. Physiol..

[19] Goldring S.R., Goldring M.B. (2004). The Role of Cytokines in Cartilage Matrix Degeneration in Osteoarthritis. Clin. Orthop..

[20] Sokolove J., Lepus C.M. (2013). Role of Inflammation in the Pathogenesis of Osteoarthritis: Latest Findings and Interpretations. Ther. Adv. Musculoskelet Dis..

[21] Malemud C.J. (2015). Biologic Basis of Osteoarthritis: State of the Evidence. Curr. Opin. Rheumatol..

[22] Tripathi N.K., Tarrant J.M. (2018). Principles of Clinical Pathology. Toxicologic Pathology.

[23] Center S.A. (2004). Metabolic, Antioxidant, Nutraceutical, Probiotic, and Herbal Therapies Relating to the Management of Hepatobiliary Disorders. Vet. Clin. Small Anim. Pract..

[24] Tvarijonaviciute A., Barić-Rafaj R., Horvatic A., Muñoz-Prieto A., Guillemin N., Lamy E., Tumpa A., Ceron J.J., Martinez-Subiela S., Mrljak V. (2019). Identification of Changes in Serum Analytes and Possible Metabolic Pathways Associated with Canine Obesity-Related Metabolic Dysfunction. Vet. J..

[25] Muñoz-Prieto A., Cerón J.J., Martínez-Subiela S., Mrljak V., Tvarijonaviciute A. (2020). A Systematic Review and Meta-Analysis of Serum Adiponectin Measurements in the Framework of Dog Obesity. Animals.

[26] Theyse L.F.H., Mazur E.M. (2024). Osteoarthritis, Adipokines and the Translational Research Potential in Small Animal Patients. Front. Vet. Sci..

[27] Ren J., Ma J., Zhang X., Aimaiti A., Saiyiti M., Chen Y., Cao L. (2017). Diagnostic Value of Combined Serum Marker Changes and Quantitative MRI Evaluation of Cartilage Volume of Tibial Plateau in a Surgically-Induced Osteoarthritis Dog Model. J. Int. Med. Res..

[28] Hurlbeck C., Einspanier R., Pfeil I., Bondzio A. (2014). Evaluation of Biomarkers for Osteoarthritis Caused by Fragmented Medial Coronoid Process in Dogs. Res. Vet. Sci..

[29] Kapoor M., Martel-Pelletier J., Lajeunesse D., Pelletier J.P., Fahmi H. (2011). Role of Proinflammatory Cytokines in the Pathophysiology of Osteoarthritis. Nat. Rev. Rheumatol..

[30] Hillström A., Bylin J., Hagman R., Björhall K., Tvedten H., Königsson K., Fall T., Kjelgaard-Hansen M. (2016). Measurement of Serum C-Reactive Protein Concentration for Discriminating between Suppurative Arthritis and Osteoarthritis in Dogs. BMC Vet. Res..

[31] Alves J.C., Santos A., Jorge P., Lavrador C., Carreira L.M. (2022). The Influence of IL-1 and C-Reactive Protein Levels in Synovial Fluid of Companion Dogs with Bilateral Hip Osteoarthritis on Various Clinical Disease Parameters. Am. J. Vet. Res..

[32] Cardona-Ramírez S., López-Villegas C., Silva-Molano R.F. (2019). The Differentiating Ability of Four Plasma Biomarkers in Canine Hip Dysplasia. Vet. Clin. Pathol..

[33] Allan G., Davies S. (2018). Radiographic Signs of Joint Disease in Dogs and Cats. Textbook of Veterinary Diagnostic Radiology.

[34] Laflamme D. (1997). Developmental and Validation of a Body Condition Score System for Dogs. Canine Pract..

[35] Mobasheri A., Saarakkala S., Finnilä M., Karsdal M.A., Bay-Jensen A.-C., van Spil W.E. (2019). Recent Advances in Understanding the Phenotypes of Osteoarthritis. F1000Research.

[36] Bauer D.C., Hunter D.J., Abramson S.B., Attur M., Corr M., Felson D., Heinegård D., Jordan J.M., Kepler T.B., Lane N.E. (2006). Classification of Osteoarthritis Biomarkers: A Proposed Approach. Osteoarthr. Cartil..

[37] Bland J.M., Altman D.G. (1996). The Use of Transformation When Comparing Two Means. BMJ.

[38] Glidden D.V., Shiboski S.C., McCulloch C.E. (2012). Regression Methods in Biostatistics: Linear, Logistic, Survival, and Repeated Measures Models.

[39] Vongsirinavarat M., Nilmart P., Somprasong S., Apinonkul B. (2020). Identification of Knee Osteoarthritis Disability Phenotypes Regarding Activity Limitation: A Cluster Analysis. BMC Musculoskelet Disord..

[40] Schmidli M.R., Fuhrer B., Kurt N., Senn D., Drögemüller M., Rytz U., Spreng D.E., Forterre S. (2018). Inflammatory Pattern of the Infrapatellar Fat Pad in Dogs with Canine Cruciate Ligament Disease. BMC Vet. Res..

[41] Mikkola L., Holopainen S., Pessa-Morikawa T., Lappalainen A.K., Hytönen M.K., Lohi H., Iivanainen A. (2019). Genetic Dissection of Canine Hip Dysplasia Phenotypes and Osteoarthritis Reveals Three Novel Loci. BMC Genom..

[42] Hernvann A., Jaffray P., Hilliquin P., Cazalet C., Menkes C.J., Ekindjian O.G. (1996). Interleukin-1 Beta-Mediated Glucose Uptake by Chondrocytes. Inhibition by Cortisol. Osteoarthr. Cartil..

[43] Hegemann N., Wondimu A., Kohn B., Brunnberg L., Schmidt M.F.G. (2005). Cytokine Profile in Canine Immune-Mediated Polyarthritis and Osteoarthritis. Vet. Comp. Orthop. Traumatol. VCOT.

[44] Maccoux L.J., Salway F., Day P.J.R., Clements D.N. (2007). Expression Profiling of Select Cytokines in Canine Osteoarthritis Tissues. Vet. Immunol. Immunopathol..

[45] Nedunchezhiyan U., Varughese I., Sun A.R., Wu X., Crawford R., Prasadam I. (2022). Obesity, Inflammation, and Immune System in Osteoarthritis. Front. Immunol..

[46] Steen-Louws C., Popov-Celeketic J., Mastbergen S.C., Coeleveld K., Hack C.E., Eijkelkamp N., Tryfonidou M., Spruijt S., van Roon J.A.G., Lafeber F.P.J.G. (2018). IL4-10 Fusion Protein Has Chondroprotective, Anti-Inflammatory and Potentially Analgesic Effects in the Treatment of Osteoarthritis. Osteoarthr. Cartil..

[47] Acharjee A., Wijesinghe S.N., Russ D., Gkoutos G., Jones S.W. (2024). Cross-Species Transcriptomics Identifies Obesity Associated Genes between Human and Mouse Studies. J. Transl. Med..

[48] Mason J.E., Starke R.D., Van Kirk J.E. (2010). Gamma-Glutamyl Transferase: A Novel Cardiovascular Risk Biomarker. Prev. Cardiol..

[49] Ali S.S., Oni E.T., Blaha M.J., Veledar E., Feiz H.R., Feldman T., Agatston A.S., Blumenthal R.S., Conceicao R.D., Carvalho J.A.M. (2016). Elevated Gamma-Glutamyl Transferase Is Associated with Subclinical Inflammation Independent of Cardiometabolic Risk Factors in an Asymptomatic Population: A Cross-Sectional Study. Nutr. Metab..

[50] Minato-Inokawa S., Tsuboi-Kaji A., Honda M., Takeuchi M., Kitaoka K., Kurata M., Wu B., Kazumi T., Fukuo K. (2024). The Different Associations of Serum Gamma-Glutamyl Transferase and Alanine Aminotransferase with Insulin Secretion, β-Cell Function, and Insulin Resistance in Non-Obese Japanese. Sci. Rep..

[51] Sellam J., Berenbaum F. (2013). Is Osteoarthritis a Metabolic Disease?. Jt. Bone Spine.

[52] Courties A., Sellam J. (2016). Obesity and Osteoarthritis: Pathophysiological Data. Rev. Rhum. Monogr..

[53] Garessus E.D.G., de Mutsert R., Visser A.W., Rosendaal F.R., Kloppenburg M. (2016). No Association between Impaired Glucose Metabolism and Osteoarthritis. Osteoarthr. Cartil..

[54] Hahn A.K., Batushansky A., Rawle R.A., Prado Lopes E.B., June R.K., Griffin T.M. (2021). Effects of Long-Term Exercise and a High-Fat Diet on Synovial Fluid Metabolomics and Joint Structural Phenotypes in Mice: An Integrated Network Analysis. Osteoarthr. Cartil..

[55] Adeyemi W.J., Olayaki L.A. (2017). Effects of Single or Combined Induction of Diabetes Mellitus and Knee Osteoarthritis on Some Biochemical and Haematological Parameters in Rats. Exp. Mol. Pathol..

[56] Ajadi R.A., Otesile E.B., Kasali O.B. (2012). Short-Term Changes in Lipid Profile Following Experimental Osteoarthritis in Dogs. Bulg. J. Vet. Med..

[57] Kosinska M.K., Mastbergen S.C., Liebisch G., Wilhelm J., Dettmeyer R.B., Ishaque B., Rickert M., Schmitz G., Lafeber F.P., Steinmeyer J. (2016). Comparative Lipidomic Analysis of Synovial Fluid in Human and Canine Osteoarthritis. Osteoarthr. Cartil..

[58] Eichner G., Liebisch G., Hild C., Rickert M., Steinmeyer J. (2025). Serum Phospholipids and Sphingolipids Are Linked to Early-Stage Osteoarthritis by Lipidomic Profiling. Arthritis Res. Ther..

[59] Ashmeik W., Baal J.D., Foreman S.C., Joseph G.B., Bahroos E., Han M., Krug R., Link T.M. (2021). Investigating the Association of Metabolic Biomarkers with Knee Cartilage Composition and Structural Abnormalities Using MRI: A Pilot Study. Cartilage.

[60] Li K., Xiao X., Li Y., Lu S., Zi J., Sun X., Xu J., Liu H.Y., Li X., Song T. (2024). Insights into the Interplay between Gut Microbiota and Lipid Metabolism in the Obesity Management of Canines and Felines. J. Anim. Sci. Biotechnol..

[61] Sudirman S., Chang H.-W., Chen C.-K., Kong Z.-L. (2019). A Dietary Polysaccharide from Eucheuma Cottonii Downregulates Proinflammatory Cytokines and Ameliorates Osteoarthritis-Associated Cartilage Degradation in Obese Rats. Food Funct..

[62] Taşoğlu Ö., Bölük H., Şahin Onat Ş., Taşoğlu İ., Özgirgin N. (2016). Is Blood Neutrophil-Lymphocyte Ratio an Independent Predictor of Knee Osteoarthritis Severity?. Clin. Rheumatol..

[63] Honsawek S., Chayanupatkul M. (2010). Correlation of Plasma and Synovial Fluid Adiponectin with Knee Osteoarthritis Severity. Arch. Med. Res..

[64] Francin P.-J., Abot A., Guillaume C., Moulin D., Bianchi A., Gegout-Pottie P., Jouzeau J.-Y., Mainard D., Presle N. (2014). Association between Adiponectin and Cartilage Degradation in Human Osteoarthritis. Osteoarthr. Cartil..

[65] Cuzdan Coskun N., Ay S., Evcik F.D., Oztuna D. (2017). Adiponectin: Is It a Biomarker for Assessing the Disease Severity in Knee Osteoarthritis Patients?. Int. J. Rheum. Dis..

[66] Koskinen A., Juslin S., Nieminen R., Moilanen T., Vuolteenaho K., Moilanen E. (2011). Adiponectin Associates with Markers of Cartilage Degradation in Osteoarthritis and Induces Production of Proinflammatory and Catabolic Factors through Mitogen-Activated Protein Kinase Pathways. Arthritis Res. Ther..

[67] Gross J.-B., Guillaume C., Gégout-Pottie P., Mainard D., Presle N. (2014). Synovial Fluid Levels of Adipokines in Osteoarthritis: Association with Local Factors of Inflammation and Cartilage Maintenance. Biomed. Mater. Eng..

[68] Dam E.B., Byrjalsen I., Karsdal M.A., Qvist P., Christiansen C. (2009). Increased Urinary Excretion of C-Telopeptides of Type II Collagen (CTX-II) Predicts Cartilage Loss over 21 Months by MRI. Osteoarthr. Cartil..

[69] Duclos M.E., Roualdes O., Cararo R., Rousseau J.C., Roger T., Hartmann D.J. (2010). Significance of the Serum CTX-II Level in an Osteoarthritis Animal Model: A 5-Month Longitudinal Study. Osteoarthr. Cartil..

[70] Cheng H., Hao B., Sun J., Yin M. (2020). C-Terminal Cross-Linked Telopeptides of Type II Collagen as Biomarker for Radiological Knee Osteoarthritis: A Meta-Analysis. Cartilage.

[71] Bruyere O., Collette J., Kothari M., Zaim S., White D., Genant H., Peterfy C., Burlet N., Ethgen D., Montague T. (2006). Osteoarthritis, Magnetic Resonance Imaging, and Biochemical Markers: A One Year Prospective Study. Ann. Rheum. Dis..

[72] Luo J., Liang S., Jin F. (2024). Gut Microbiota and Healthy Longevity. Sci. China Life Sci..

[73] Klein S.L., Flanagan K.L. (2016). Sex Differences in Immune Responses. Nat. Rev. Immunol..

[74] Bouman A., Schipper M., Heineman M.J., Faas M.M. (2004). Gender Difference in the Non-specific and Specific Immune Response in Humans. Am. J. Reprod. Immunol..

[75] Ahonen T., Vanhala M., Kautiainen H., Kumpusalo E., Saltevo J. (2012). Sex Differences in the Association of Adiponectin and Low-Grade Inflammation with Changes in the Body Mass Index from Youth to Middle Age. Gend. Med..

