# Supplementary figures and images for: Phenotypic Differences in Inflammatory, Metabolic, and Biochemical Biomarkers in Dogs with Osteoarthritis According to Body Condition and Sex
Source: Animals (Basel). 2026 Feb 23;16(4):692. doi: 10.3390/ani16040692 (PMC12937412; doi:10.3390/ani16040692)

lm(make\_response ~ DIP[putting name] + Group + Sex)

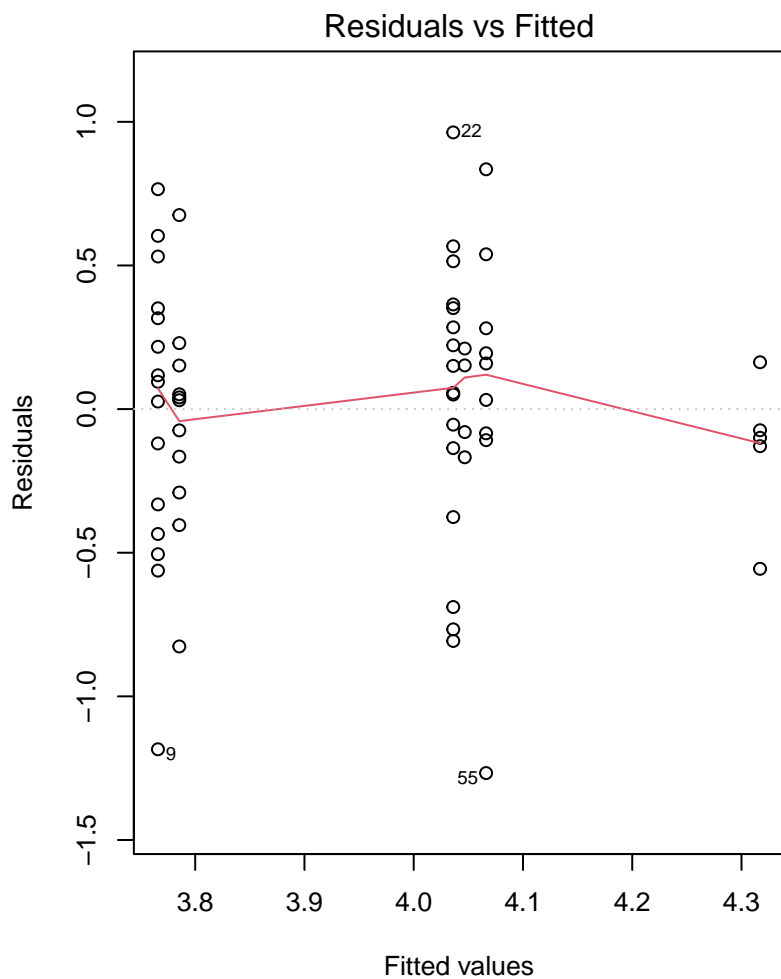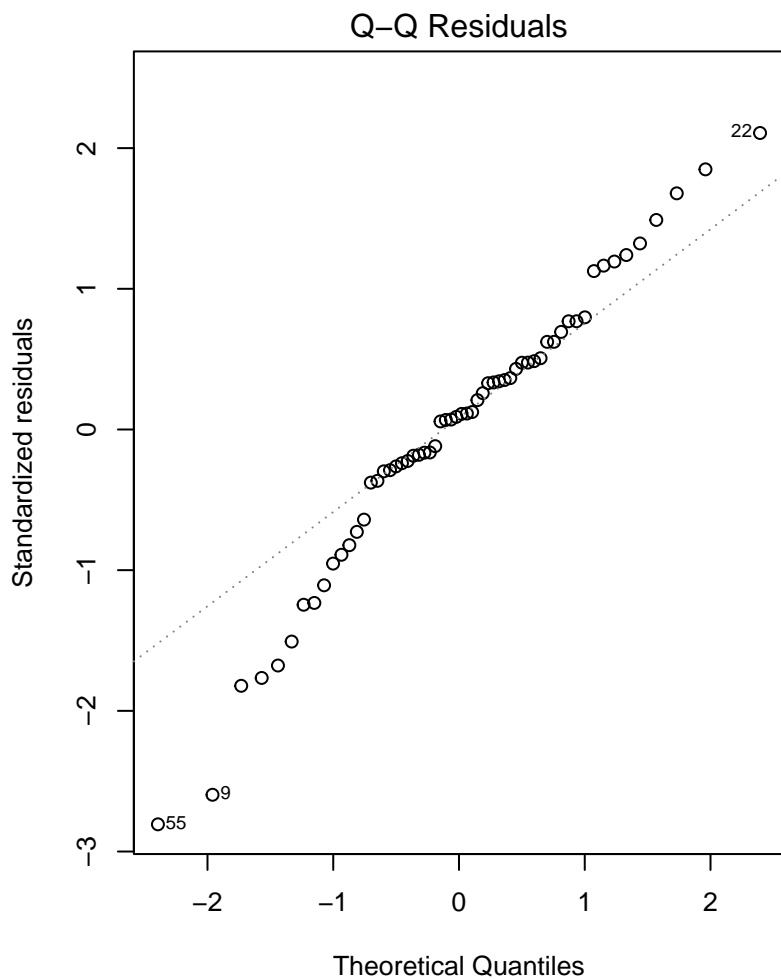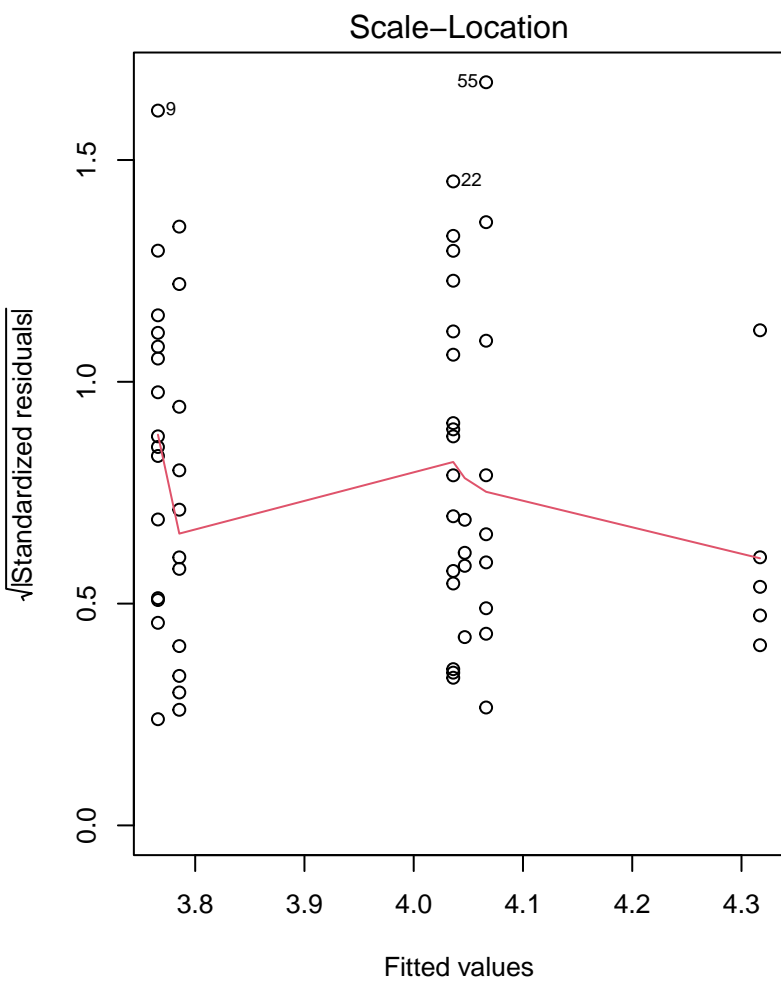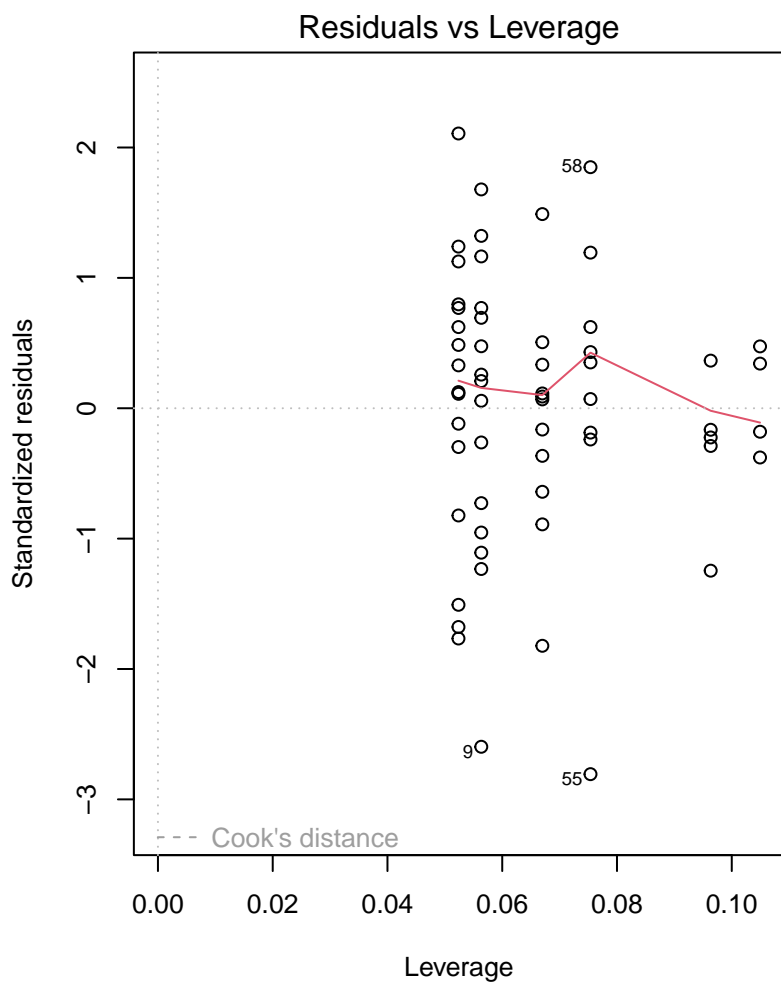

Supplement: Supplementary file 1 [file animals-16-00692-s001.zip › S1_ADP_m1_Group_Sex.pdf]

lm(aes(response, log(putzone), group = Sex + Age + Weight))

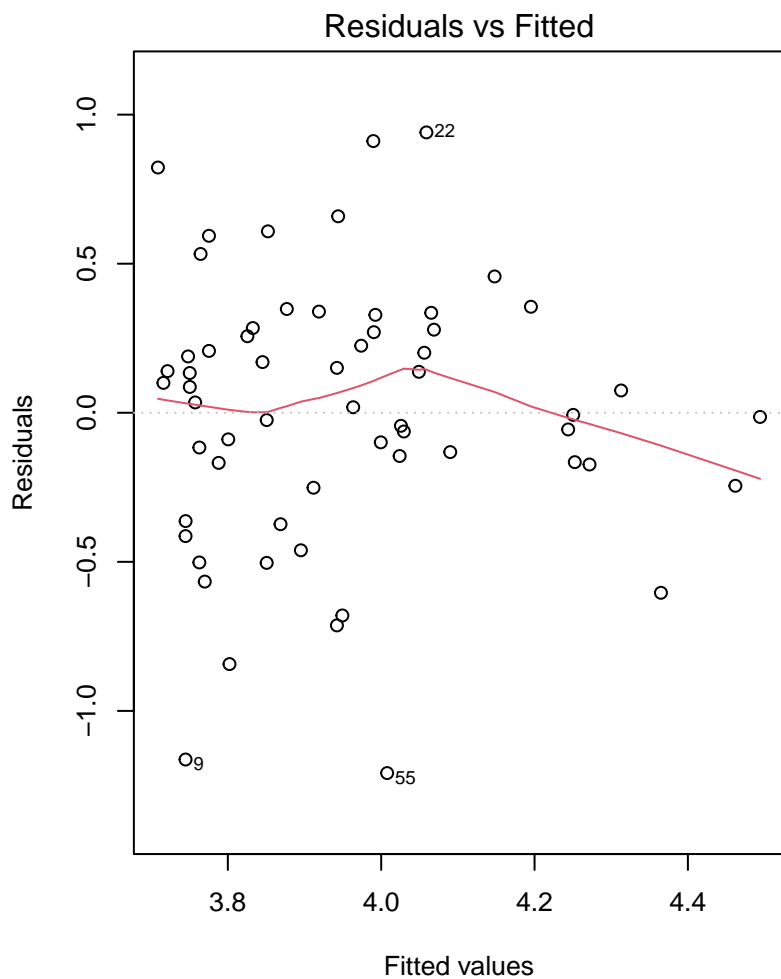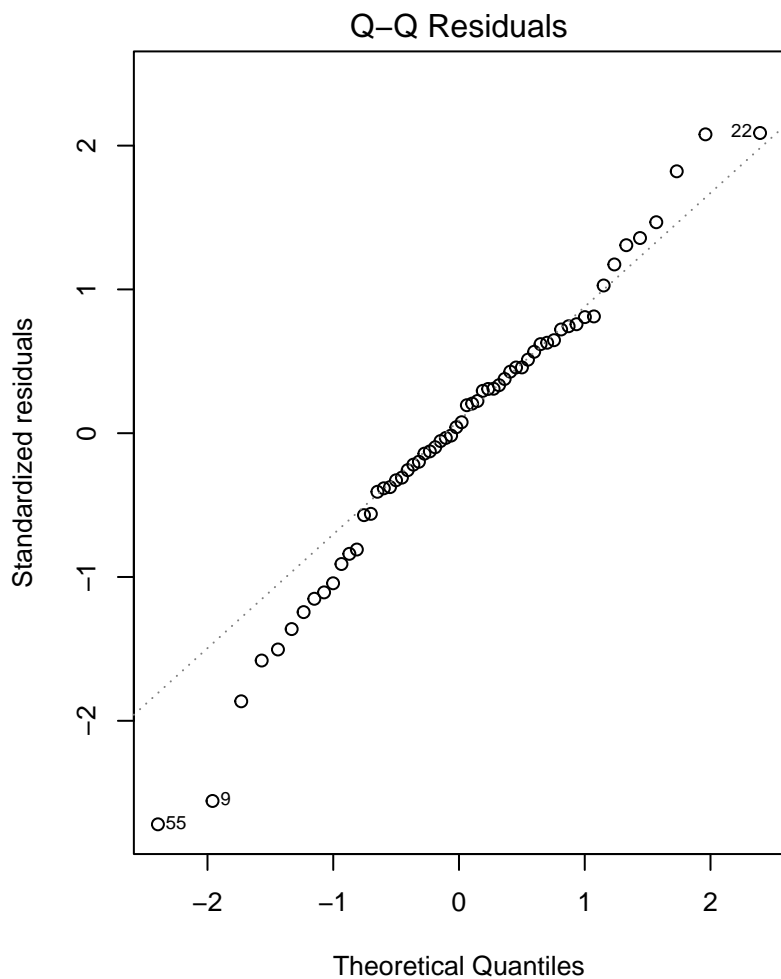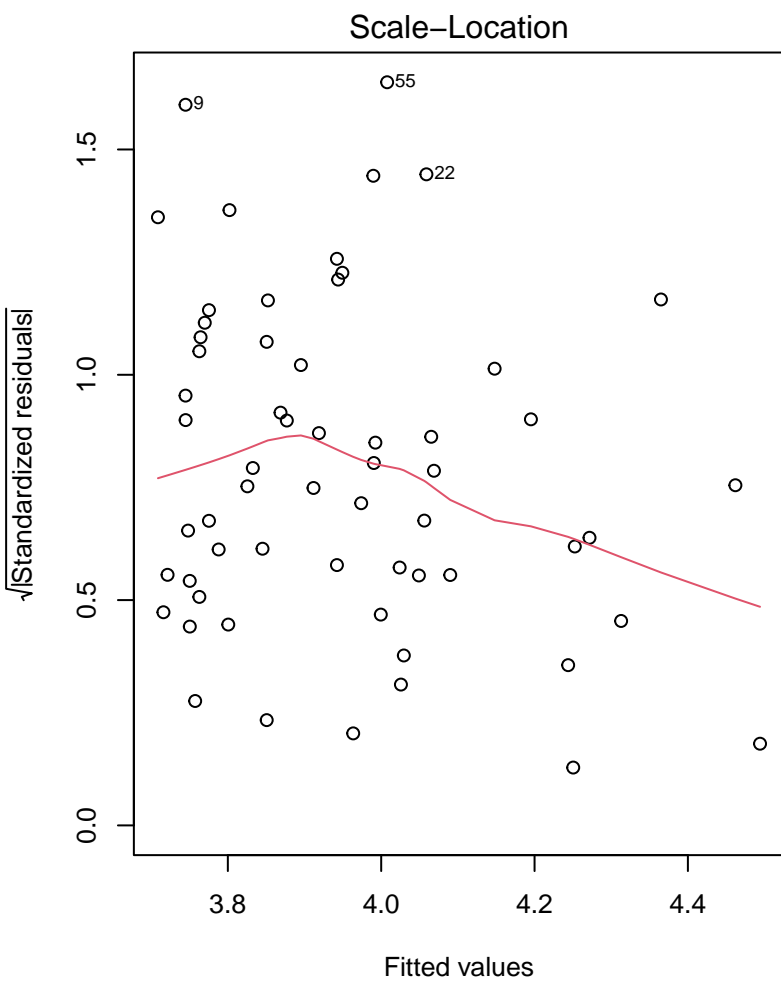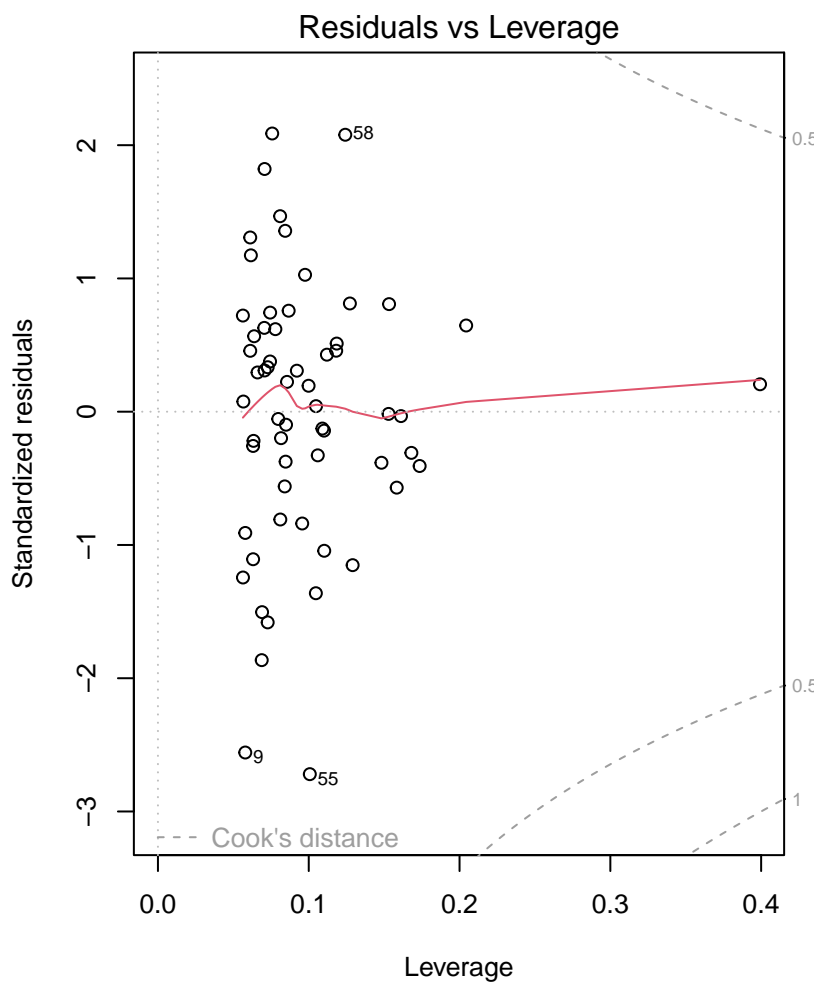

Supplement: Supplementary file 1 [file animals-16-00692-s001.zip › S1_ADP_m2_Group_Sex_Age_Weight.pdf]

# Im(makDiagnose(affOutcome(name))) GROUPS + Sex

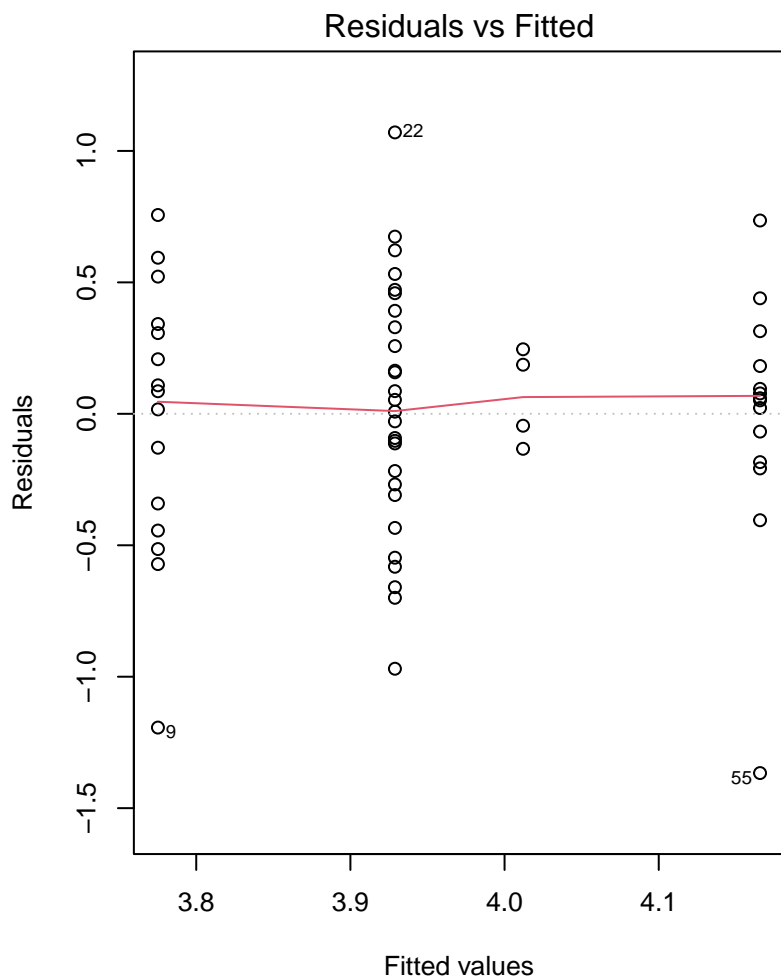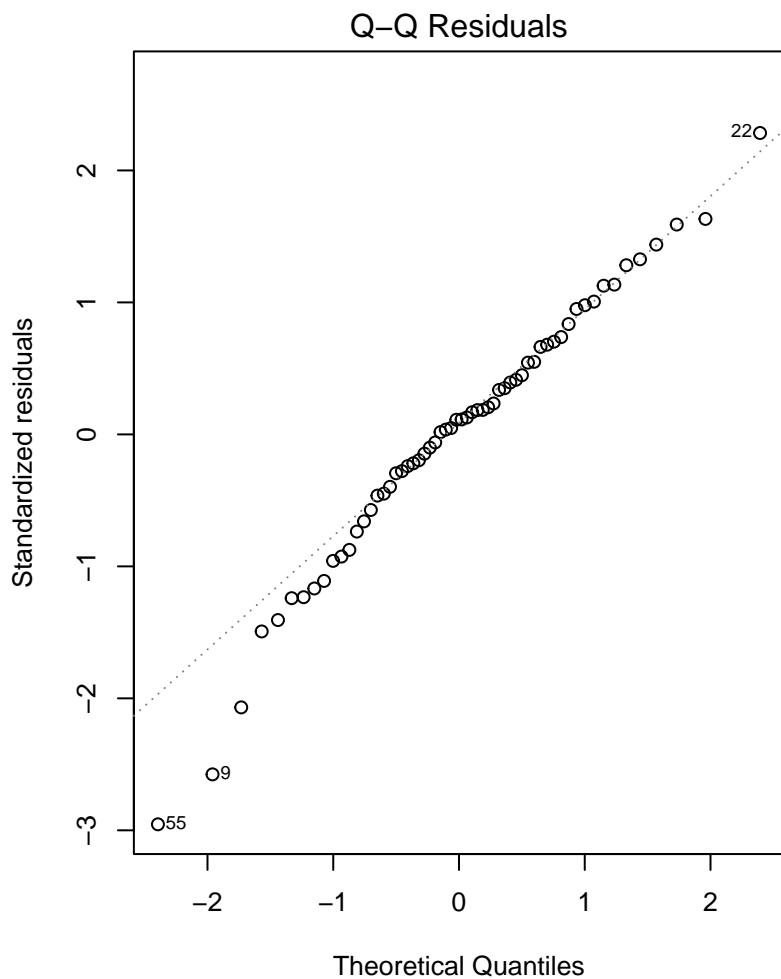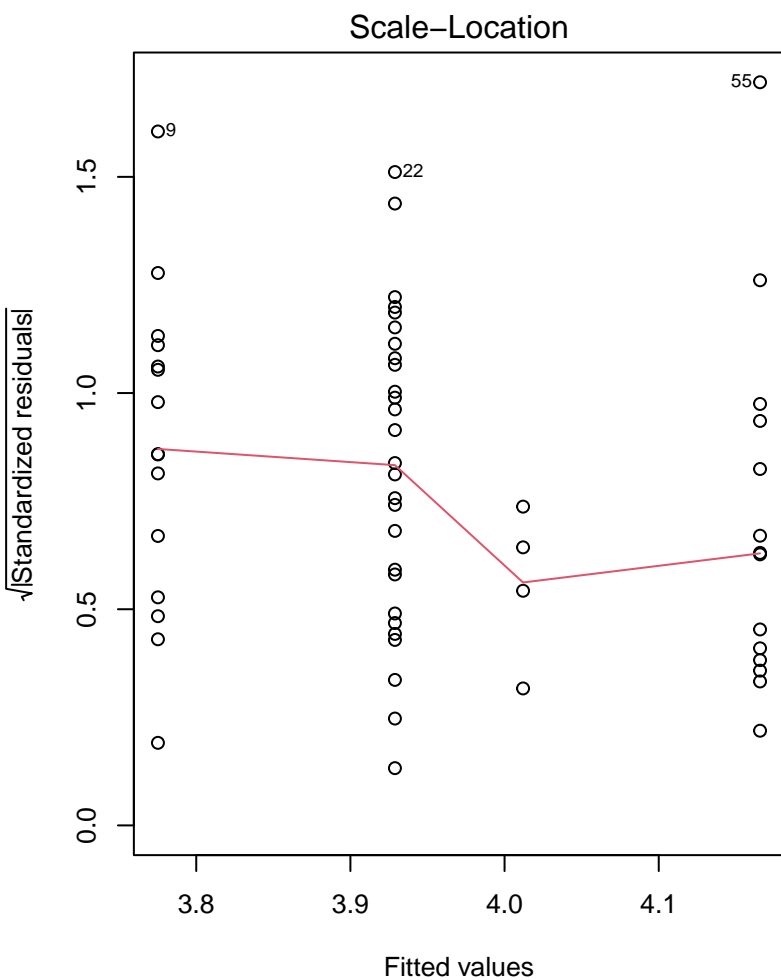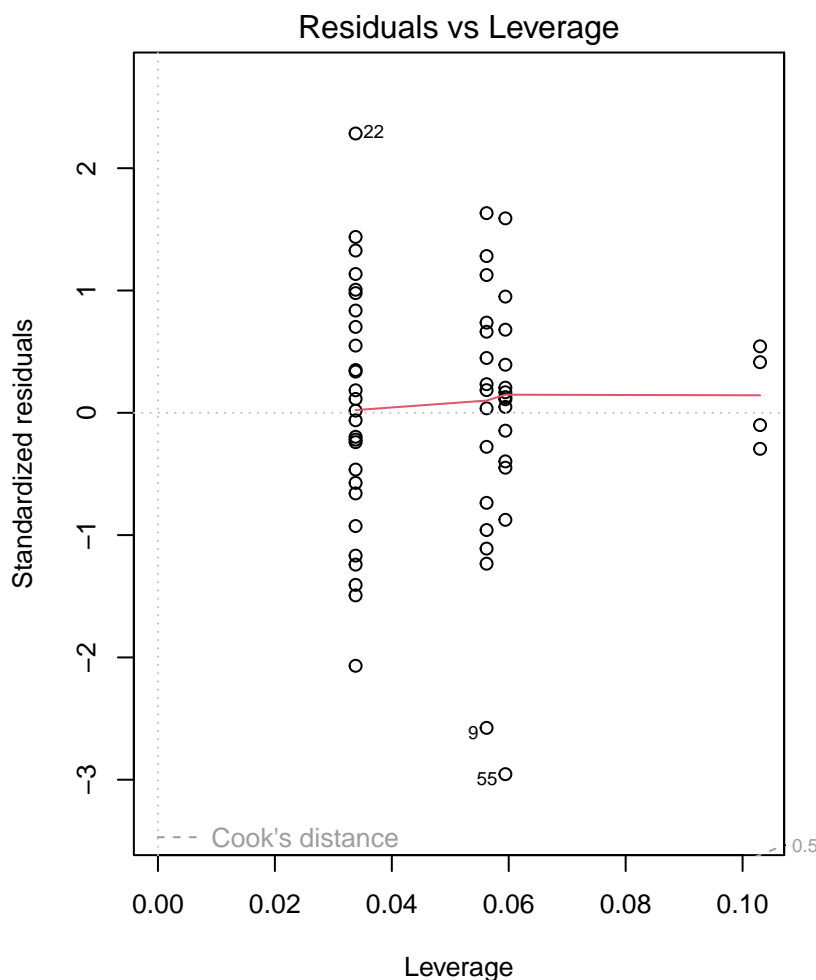

Supplement: Supplementary file 1 [file animals-16-00692-s001.zip › S1_ADP_m3_GROUPII_Sex.pdf]

lm(make\_response ~ All(outcome\_name) + Group + Sex)

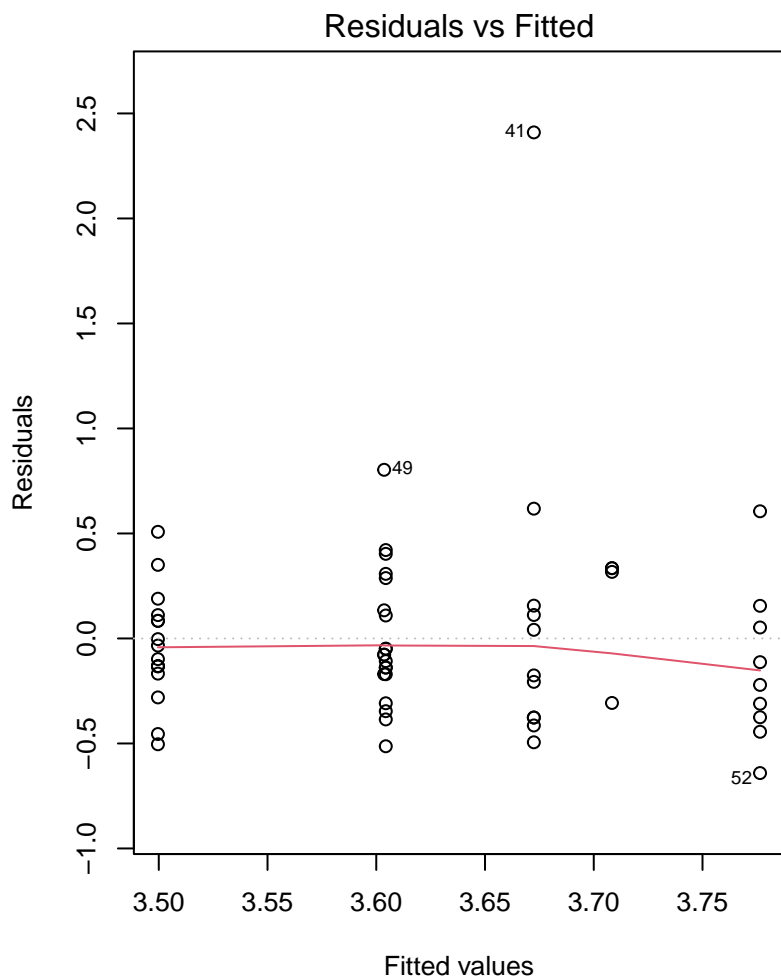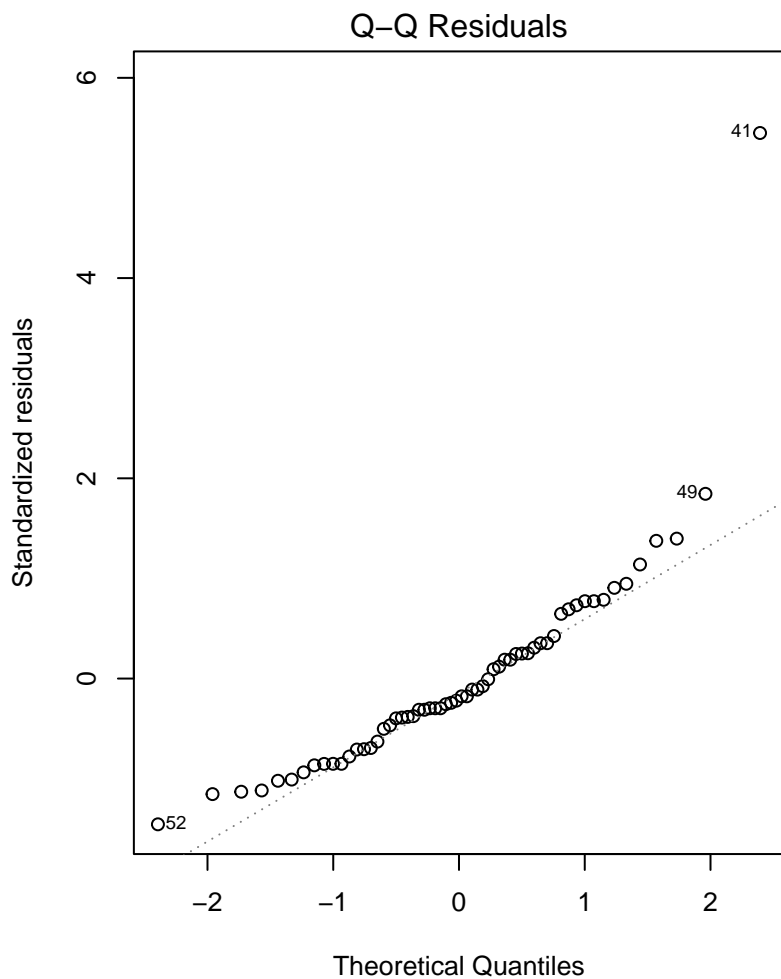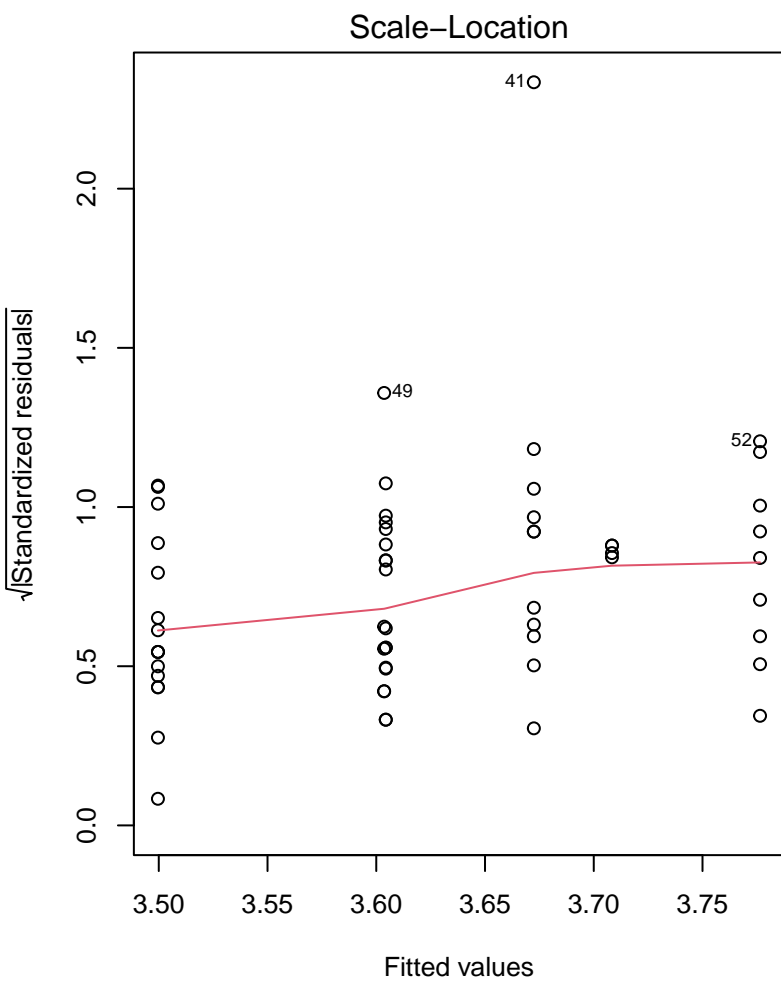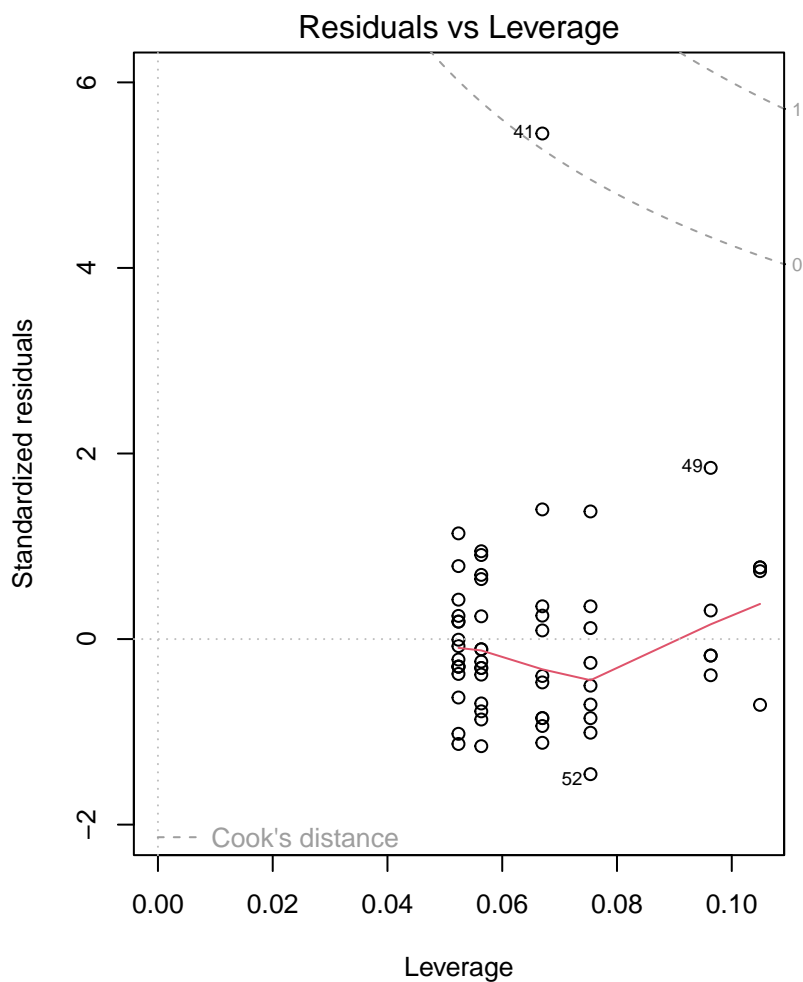

Supplement: Supplementary file 1 [file animals-16-00692-s001.zip › S1_ALT_m1_Group_Sex.pdf]

lm(make\_response ~ 42[Outcome] + Group + Sex + Age + Weight)

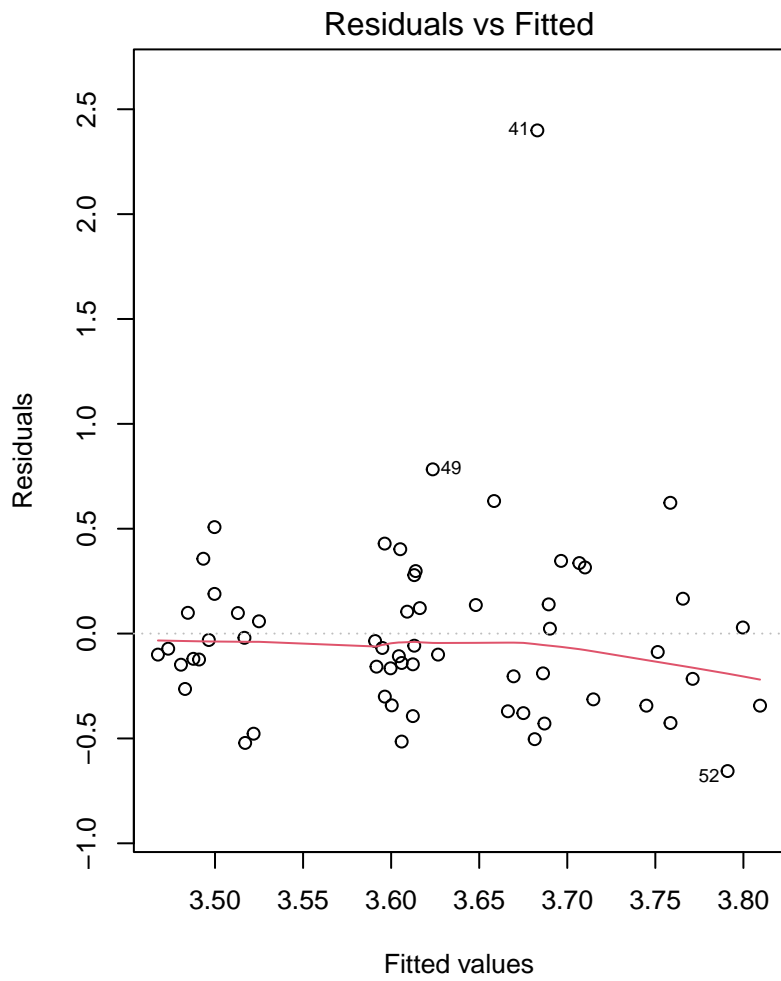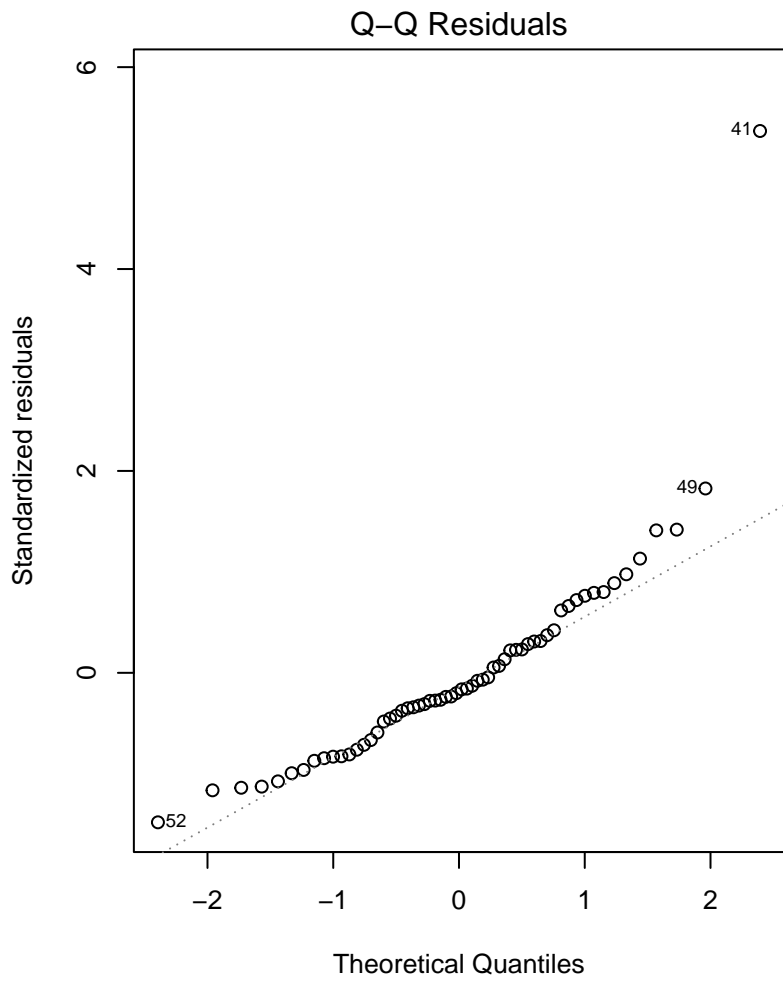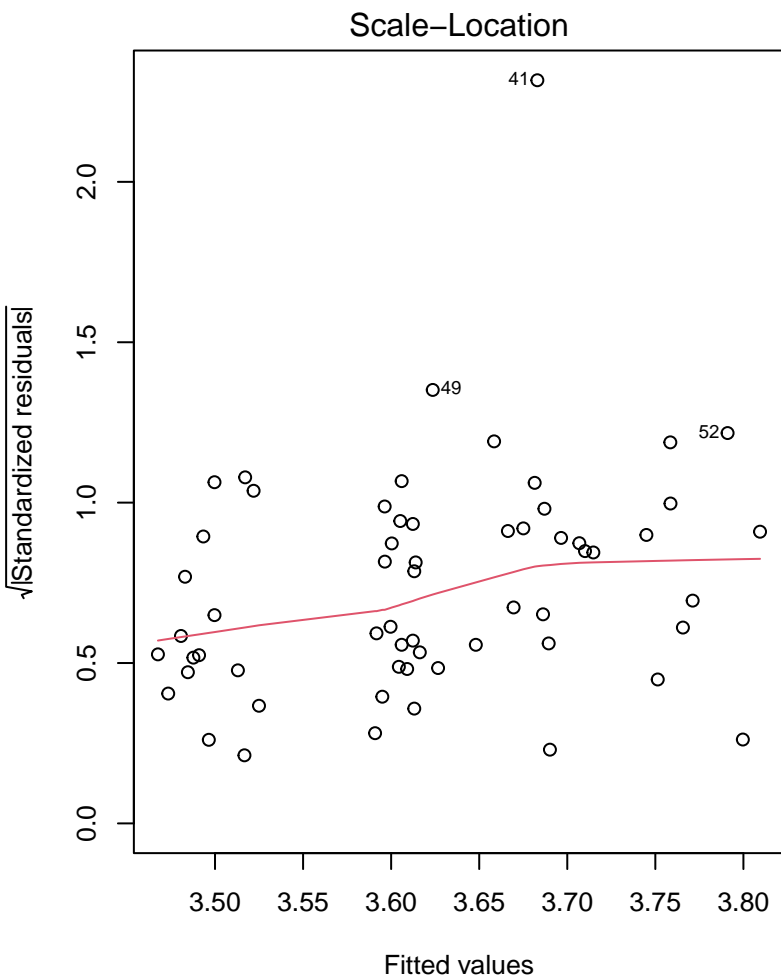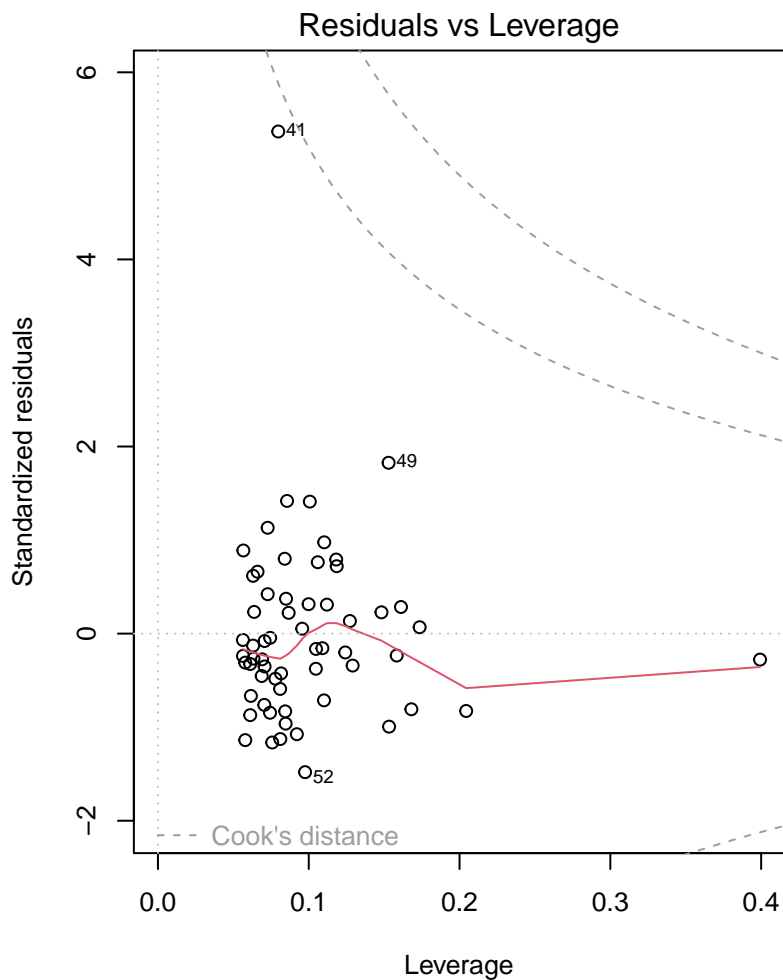

Supplement: Supplementary file 1 [file animals-16-00692-s001.zip › S1_ALT_m2_Group_Sex_Age_Weight.pdf]

lm(make\_response ~ AB[OutcomeName] + GROUBexl + Sex)

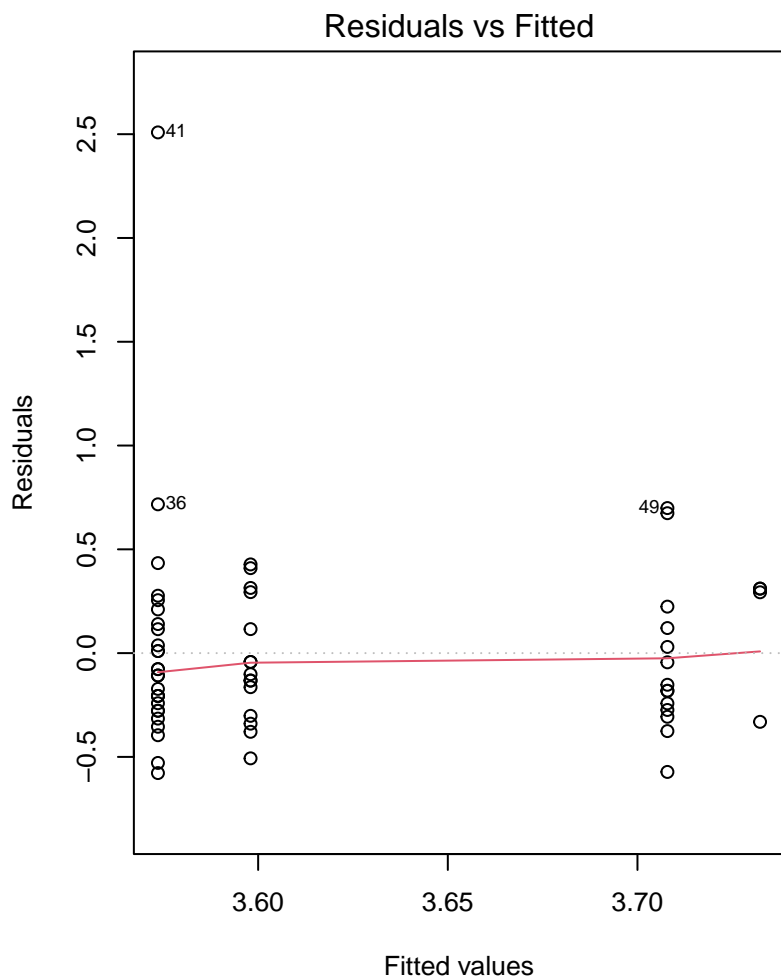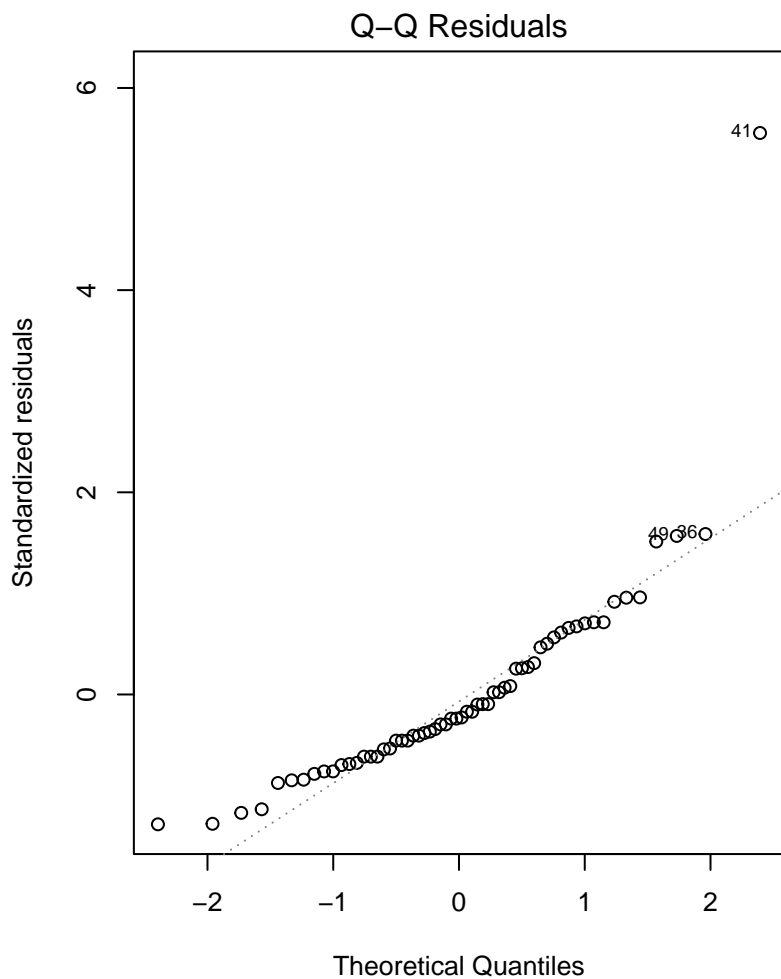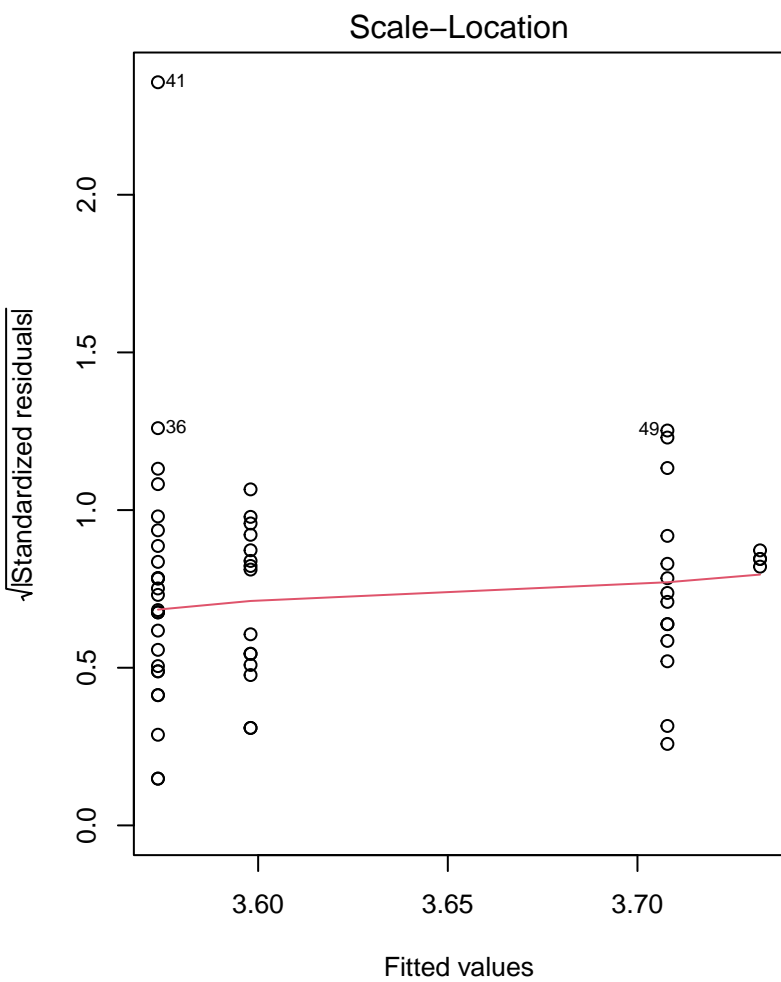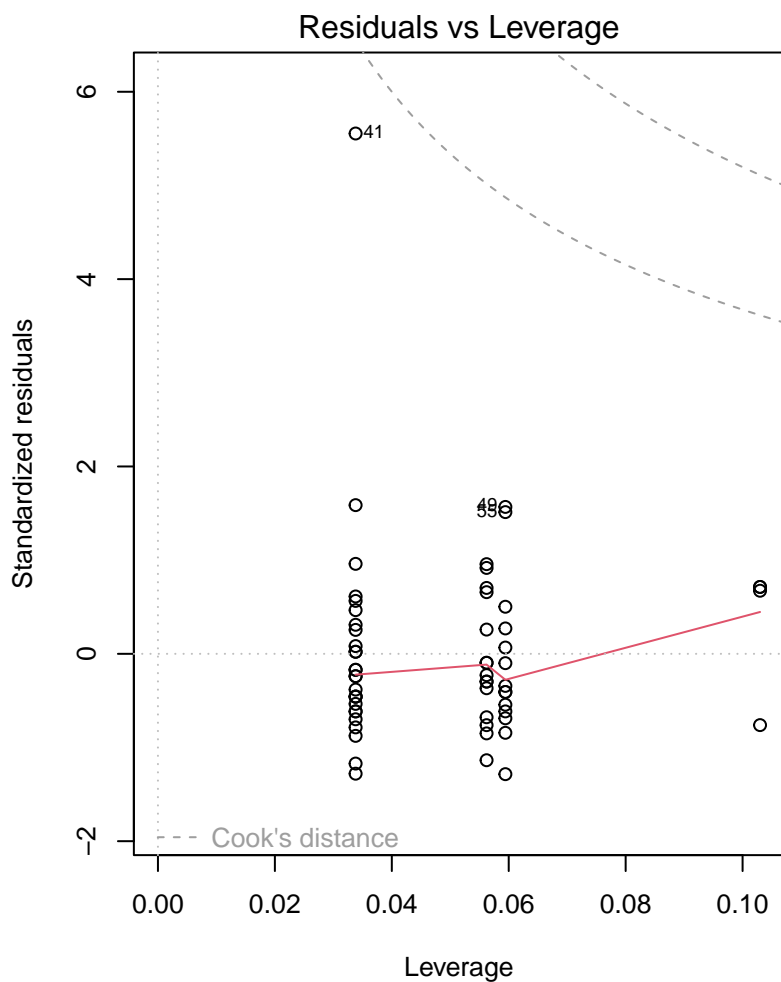

Supplement: Supplementary file 1 [file animals-16-00692-s001.zip › S1_ALT_m3_GROUPII_Sex.pdf]

lm(make\_response ~ A1[[putting\_name]] + Group + Sex)

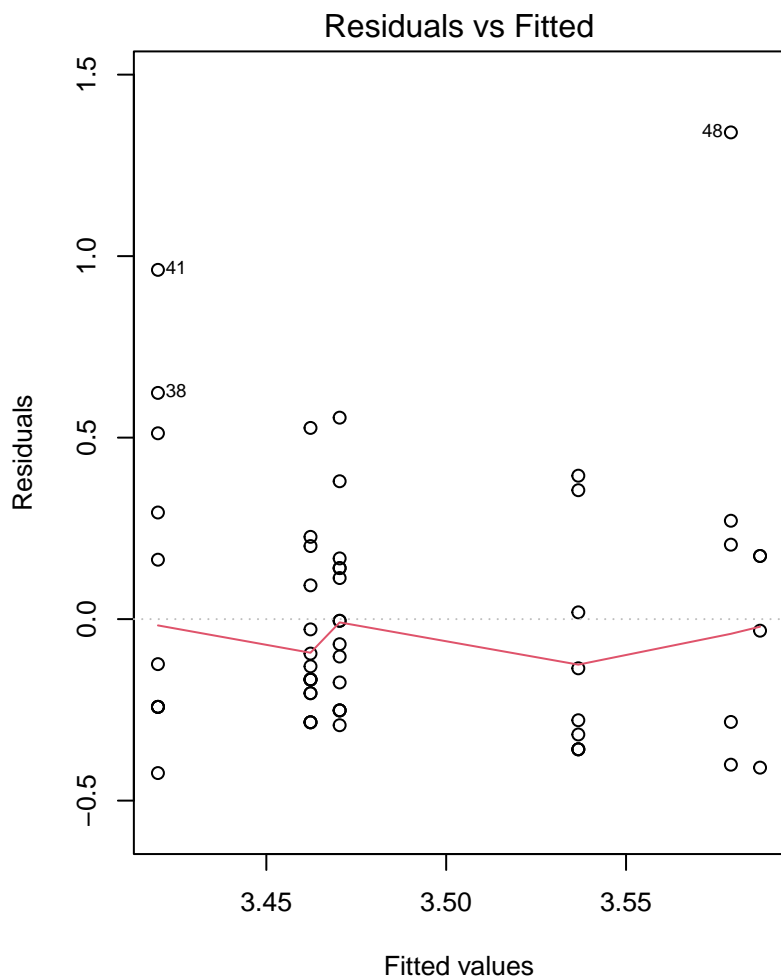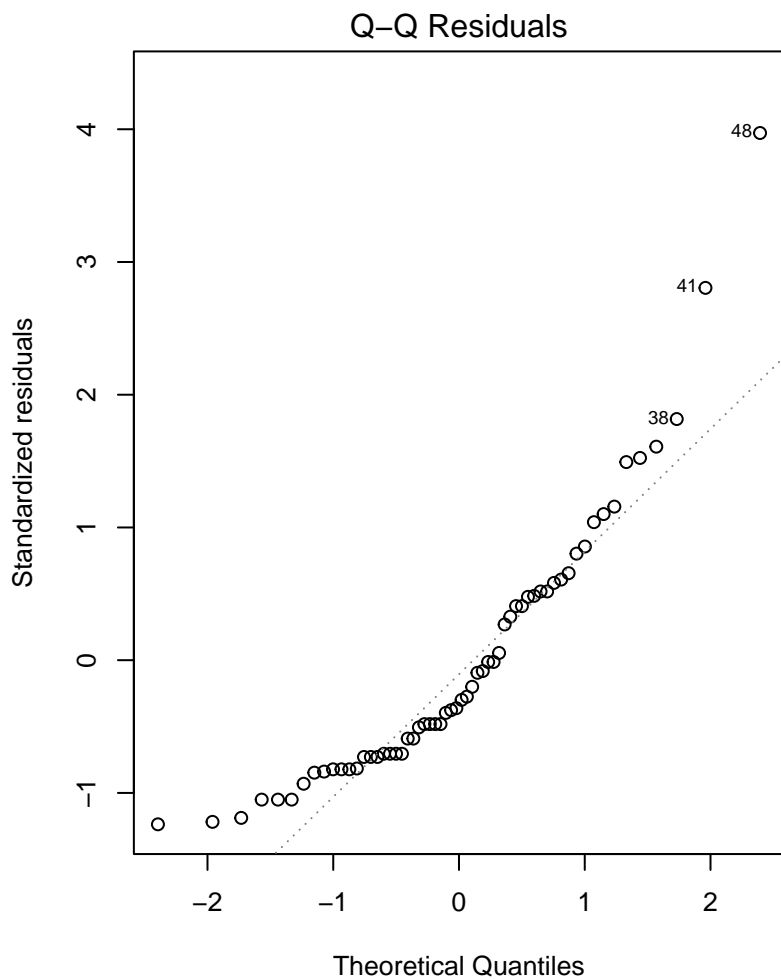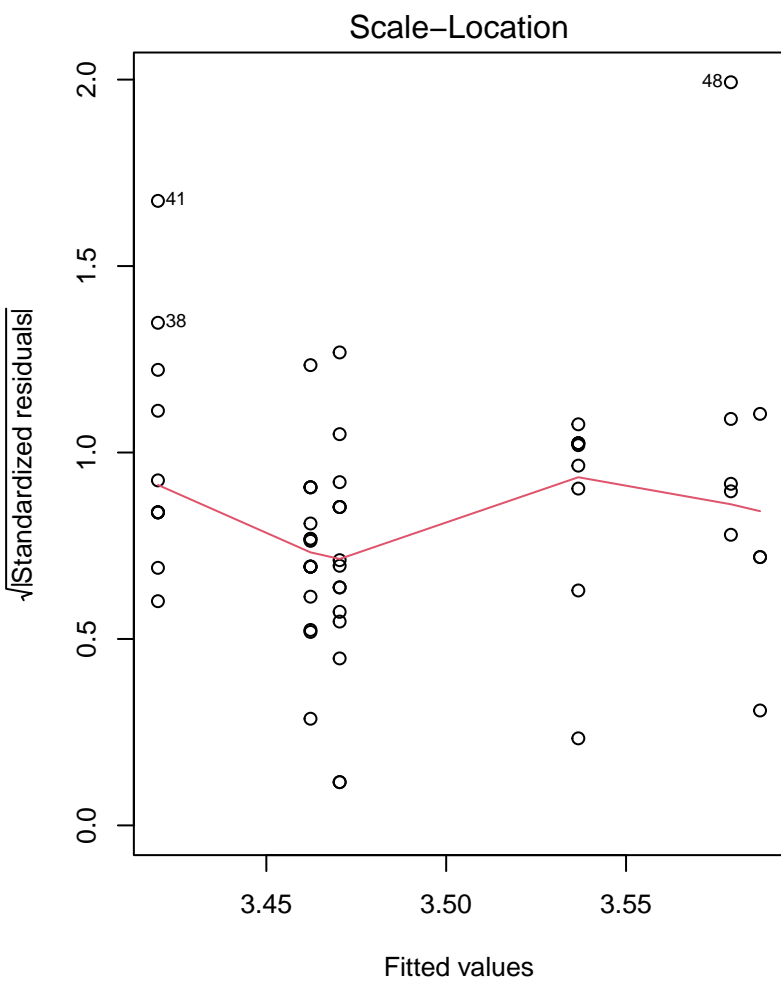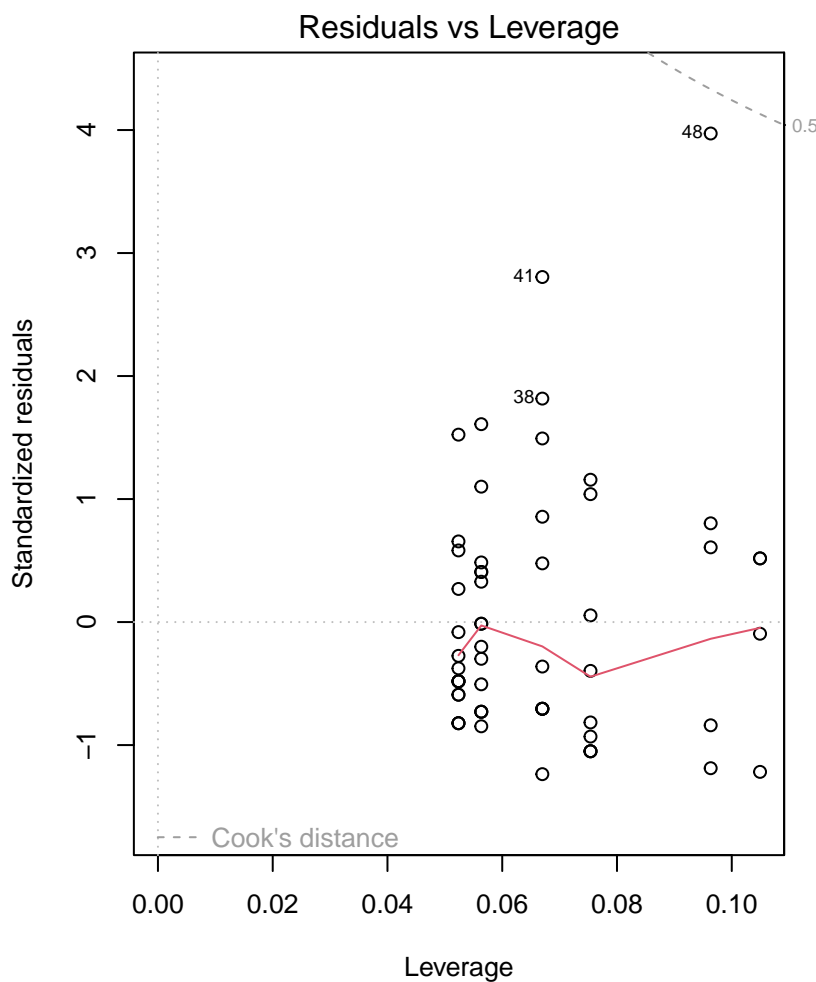

Supplement: Supplementary file 1 [file animals-16-00692-s001.zip › S1_AST_m1_Group_Sex.pdf]

lm(make\_response ~ (1 | outcome\_name) + Group + Sex + Age + Weight)

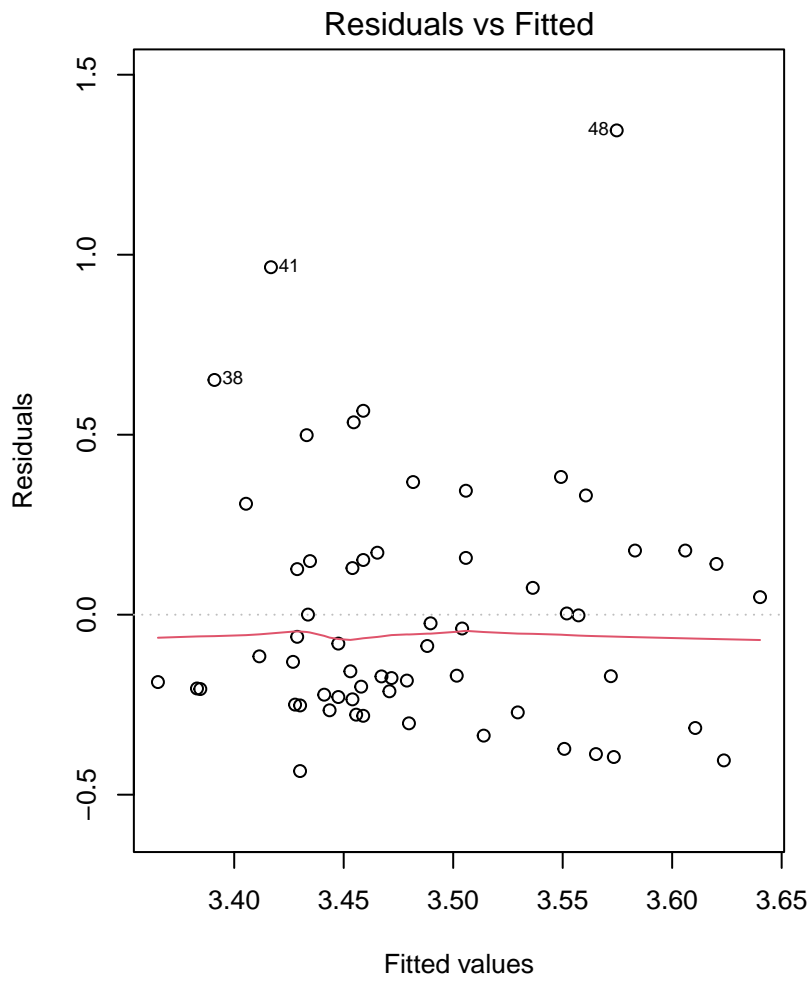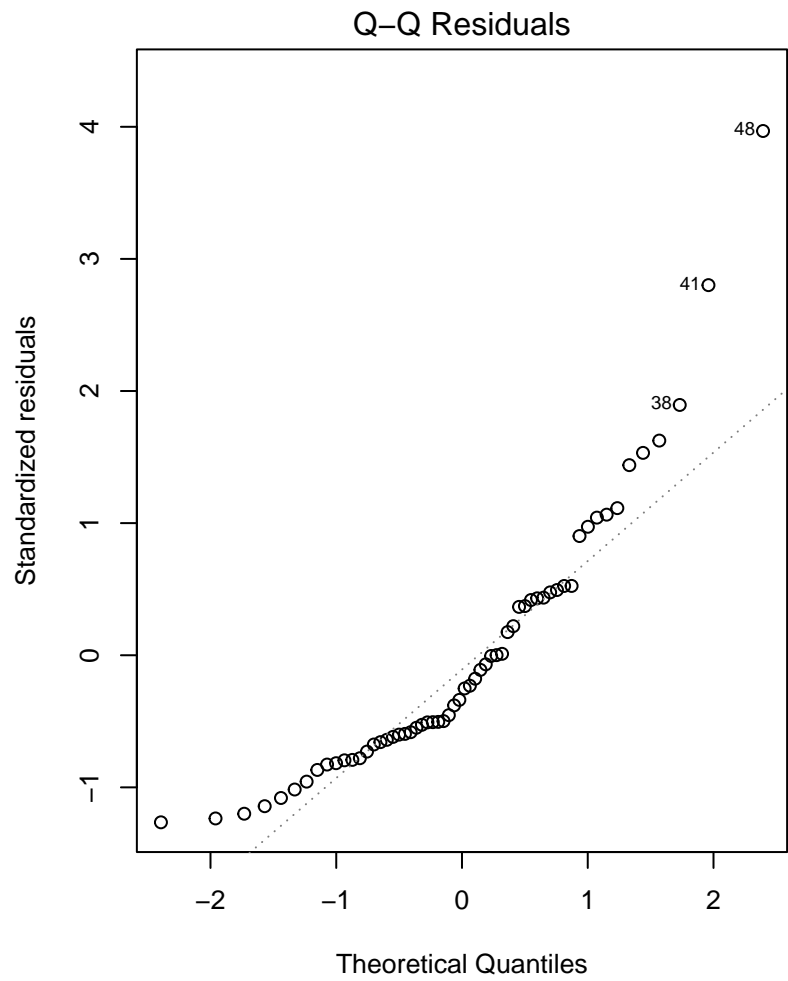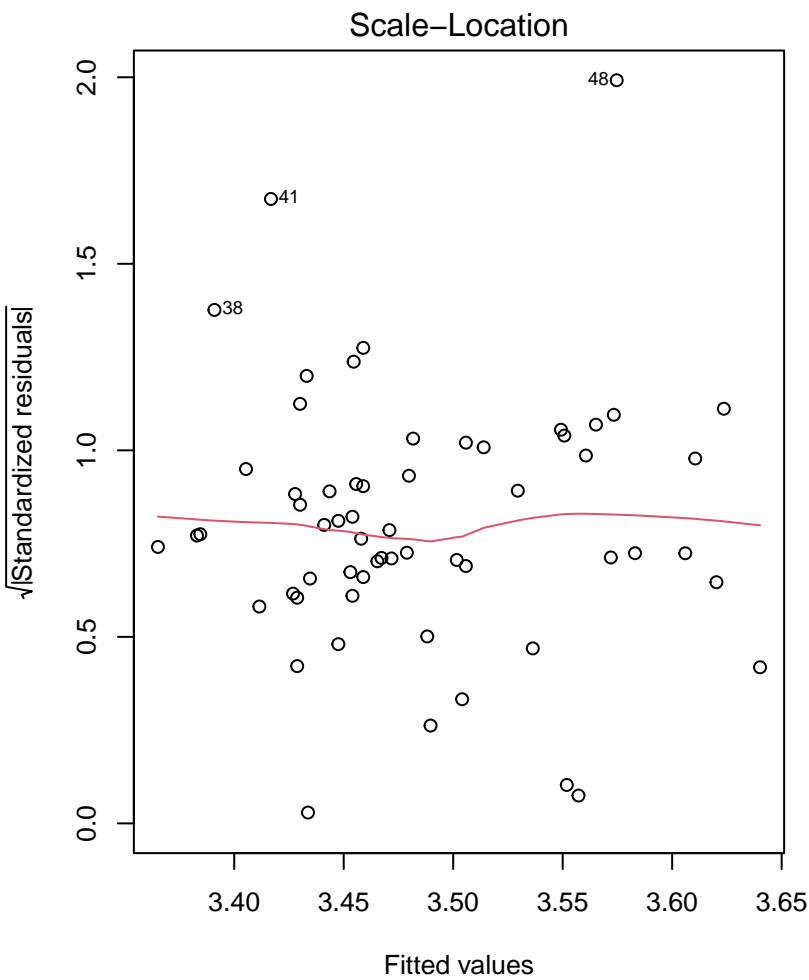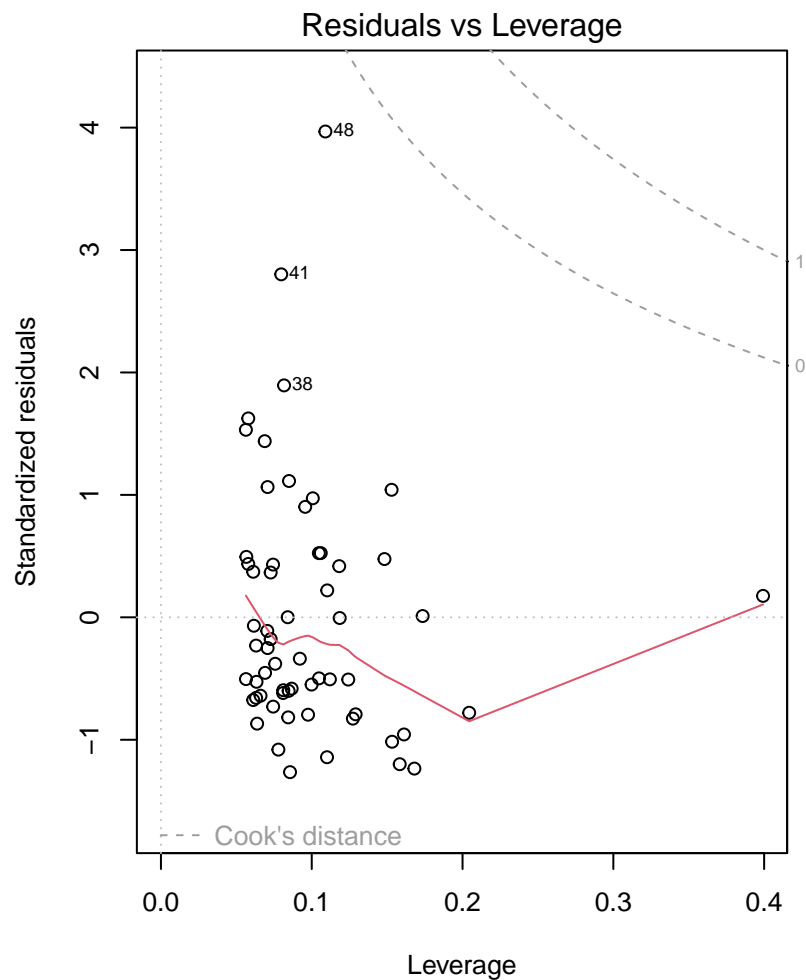

Supplement: Supplementary file 1 [file animals-16-00692-s001.zip › S1_AST_m2_Group_Sex_Age_Weight.pdf]

lm(make\_response(AS[OutcomeName]) ~ GROUP + Sex)

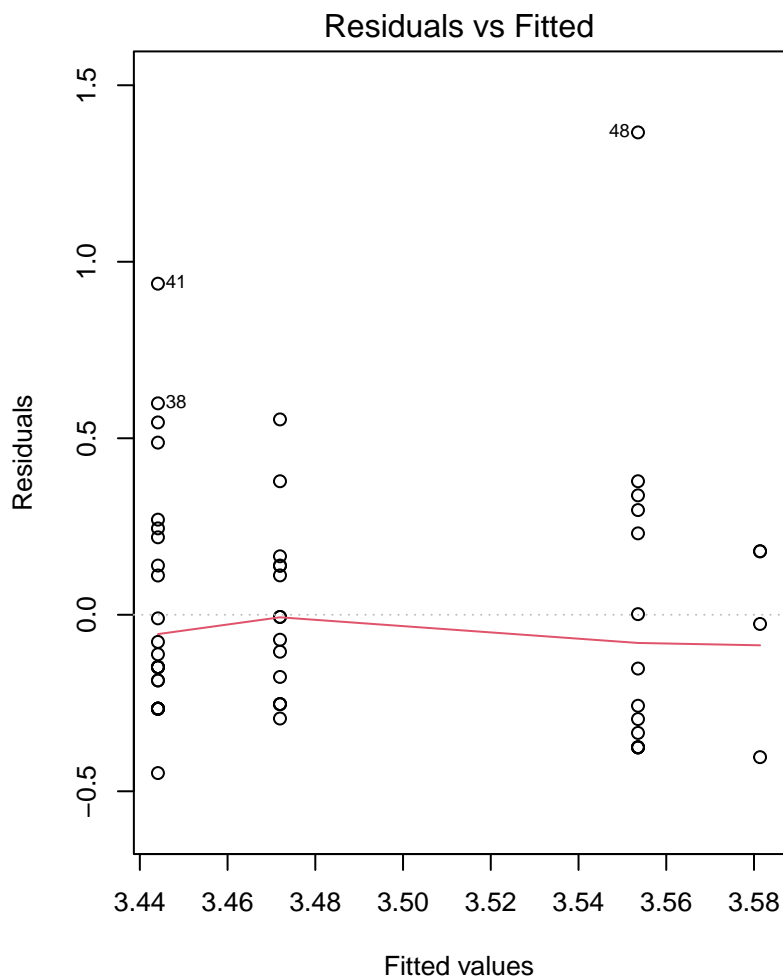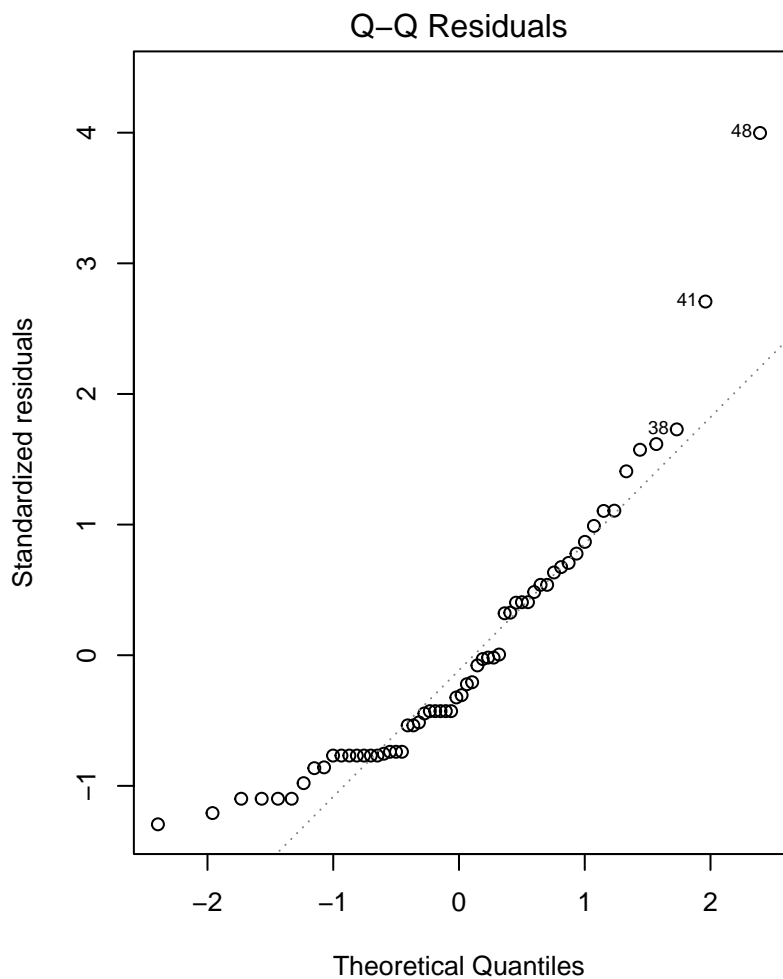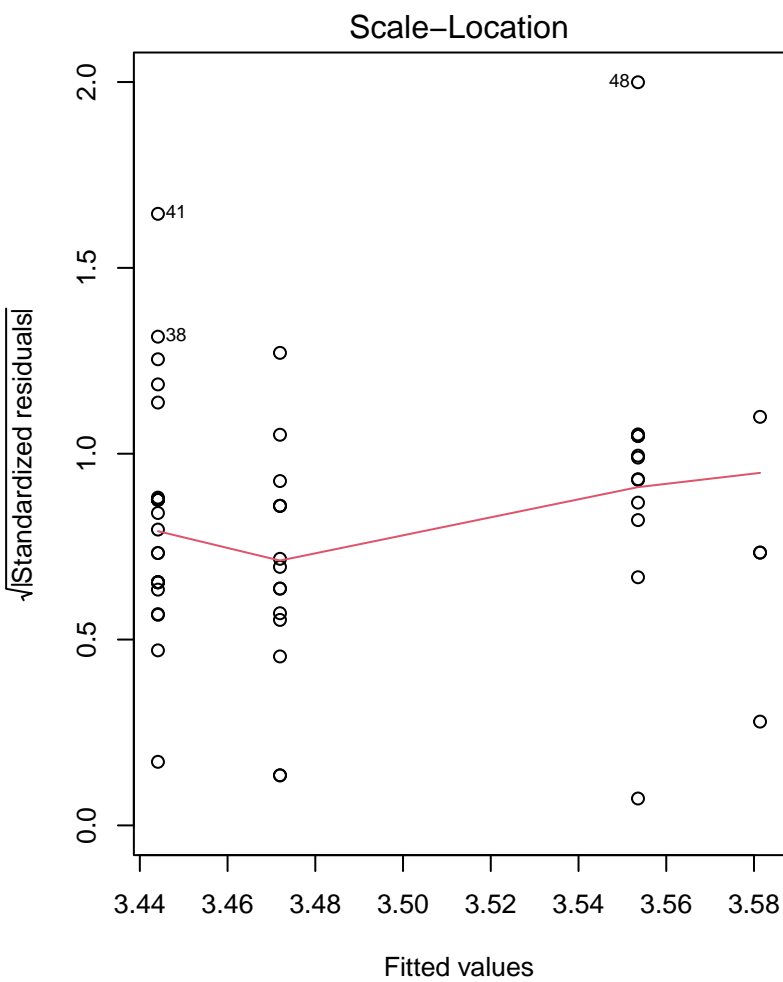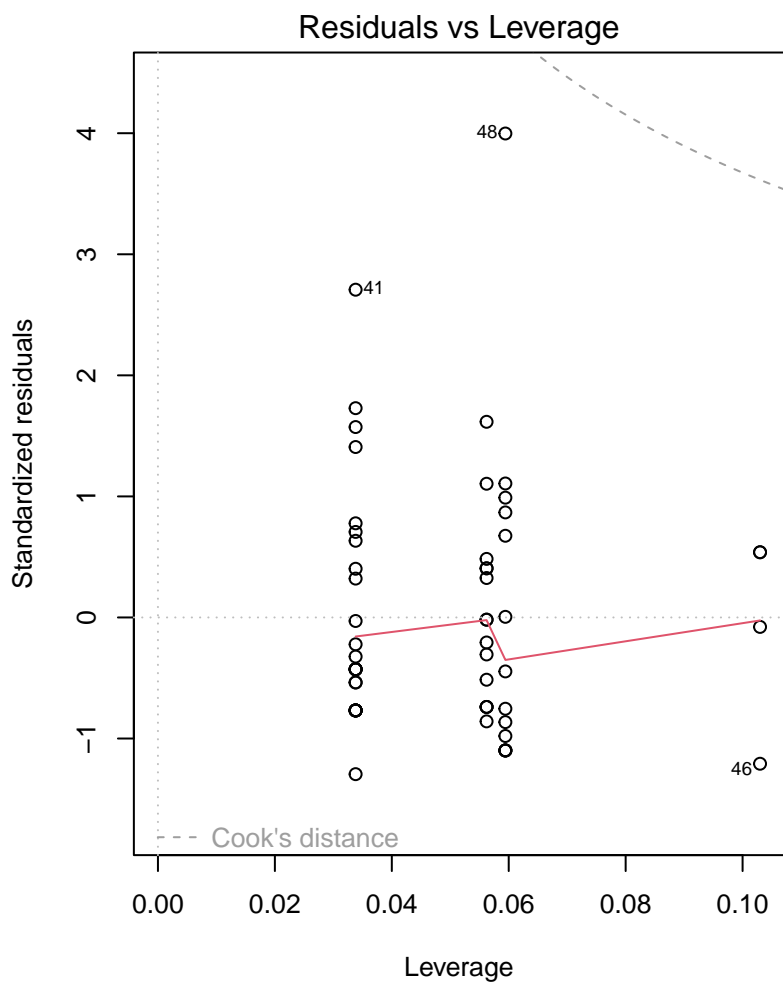

Supplement: Supplementary file 1 [file animals-16-00692-s001.zip › S1_AST_m3_GROUPII_Sex.pdf]

lm(Damage ~ Species + (1 | Group Sex))

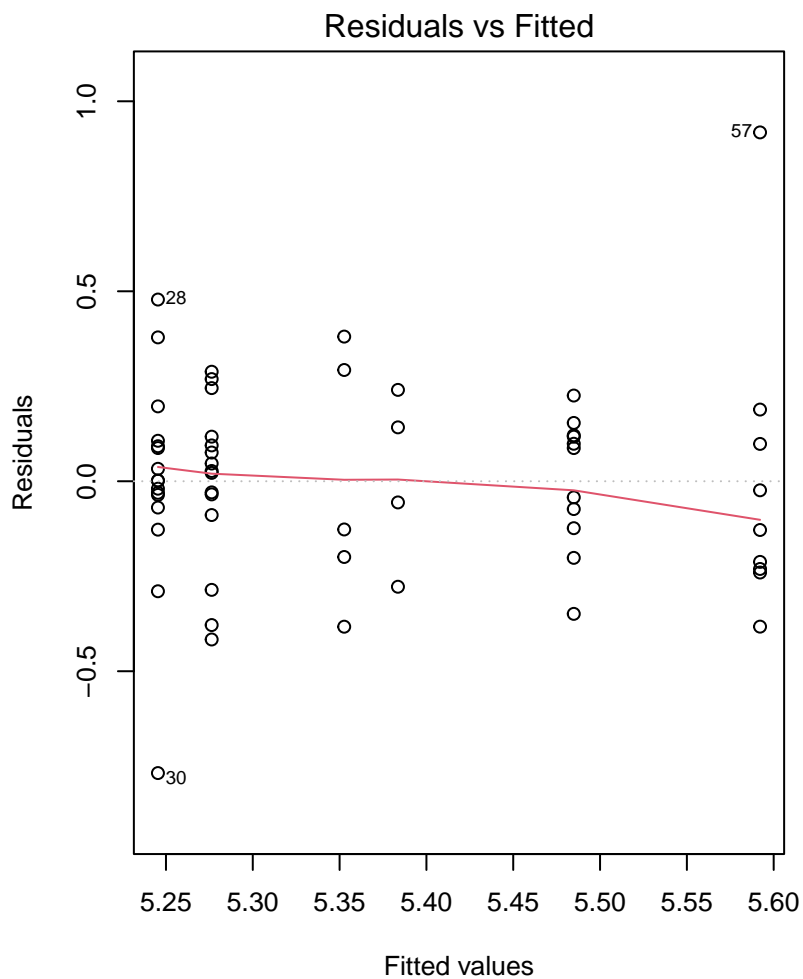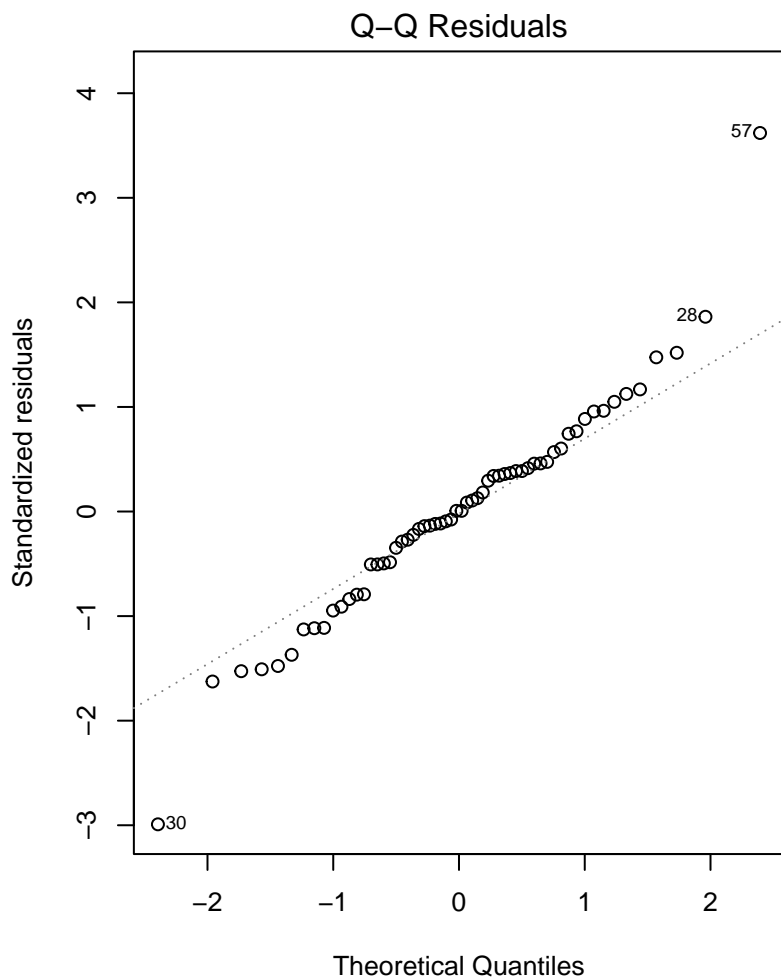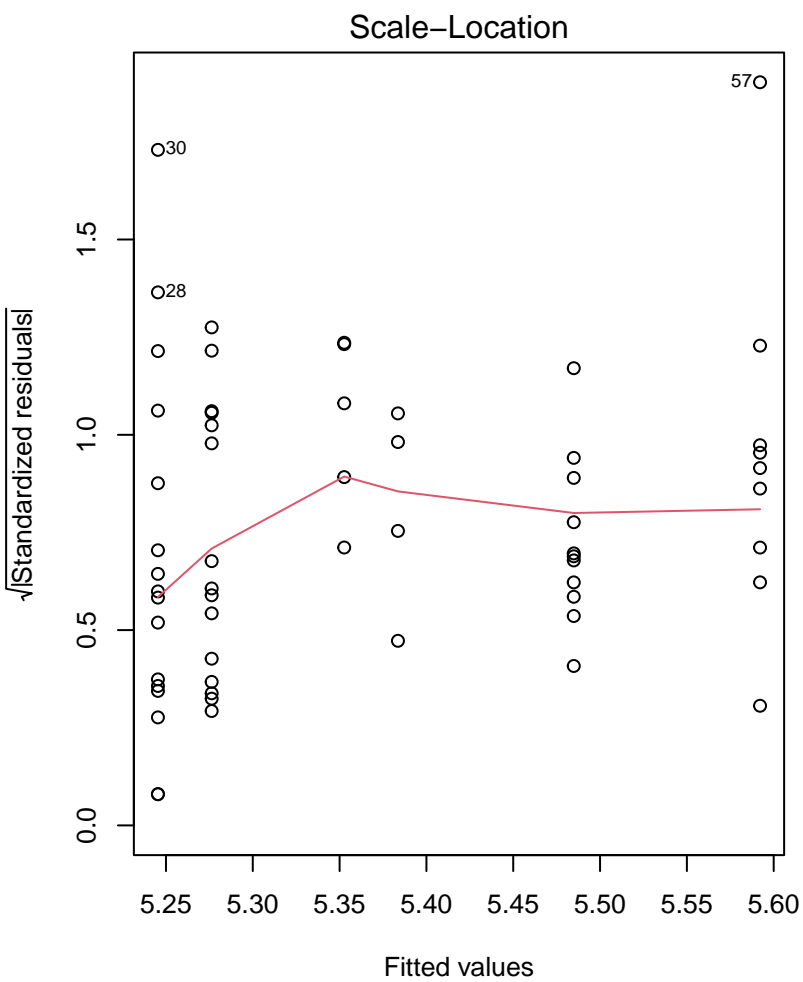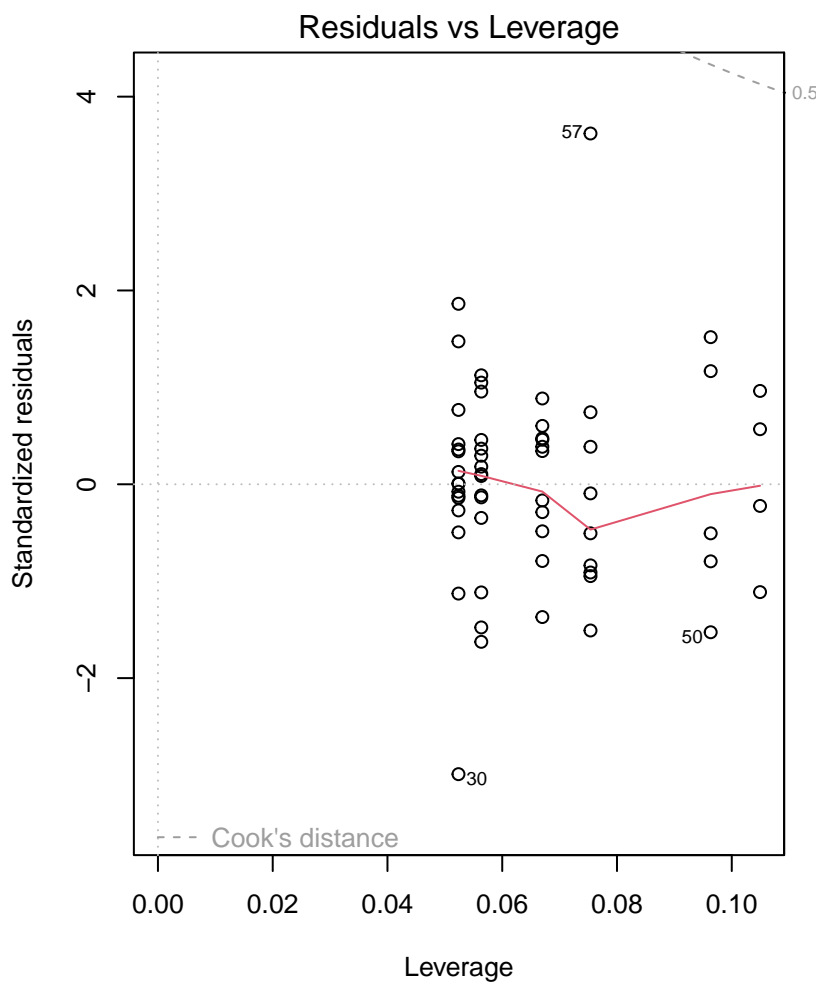

Supplement: Supplementary file 1 [file animals-16-00692-s001.zip › S1_Cholesterol_m1_Group_Sex.pdf]

lm(make\_response\_12[outcome\_log(me)] ~ Group + Sex + Age + Weight)

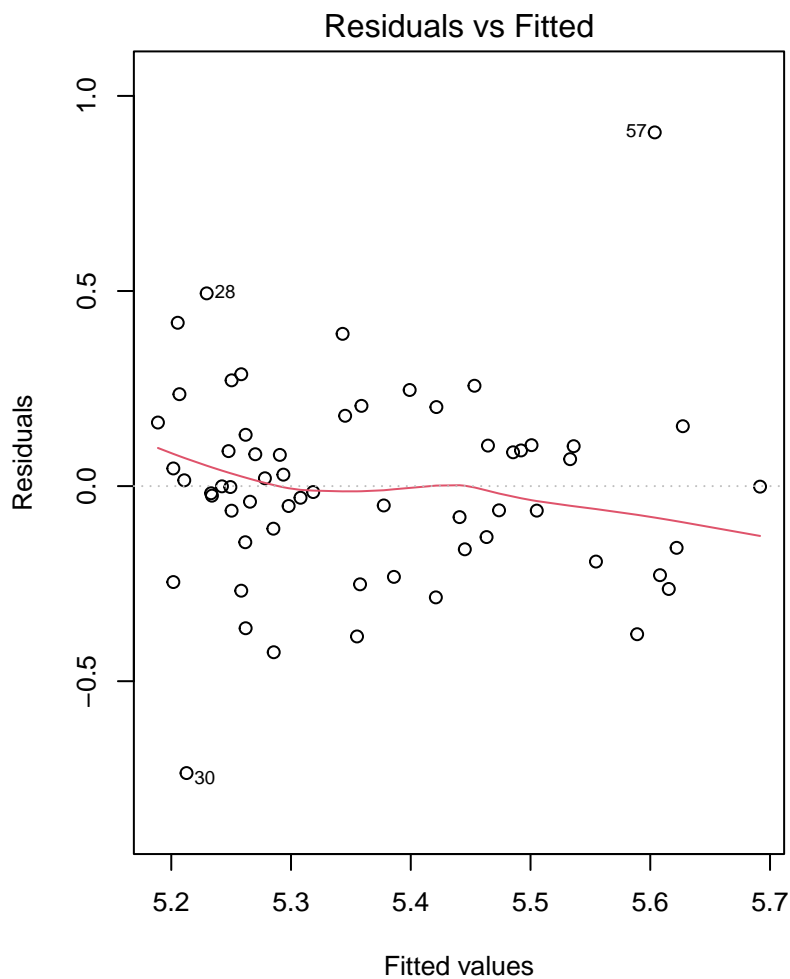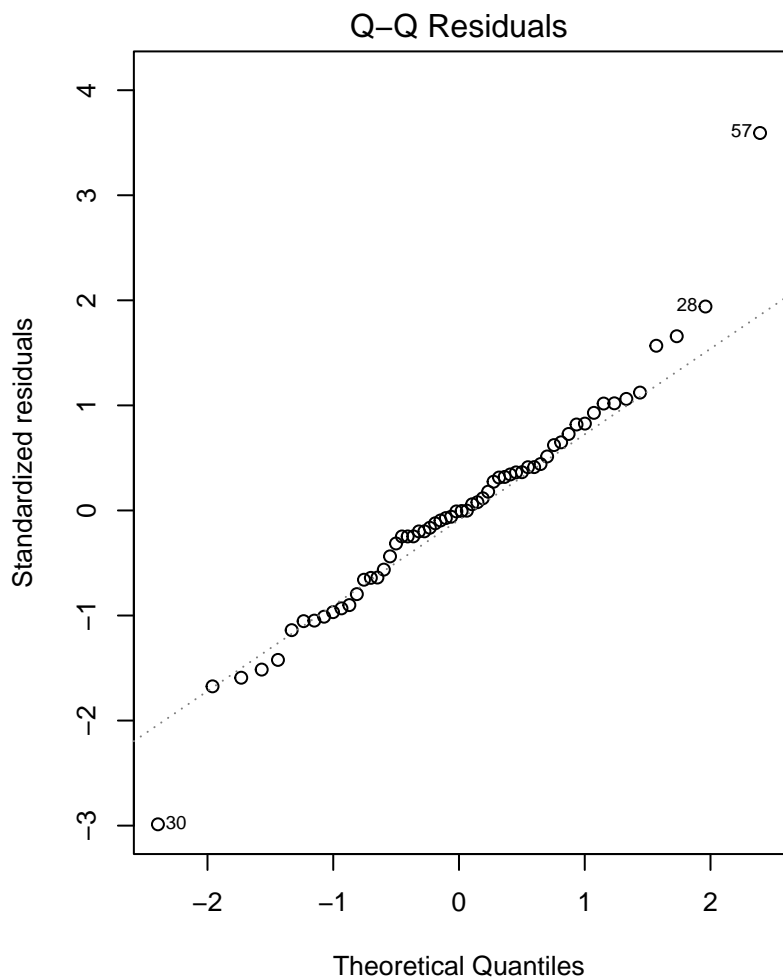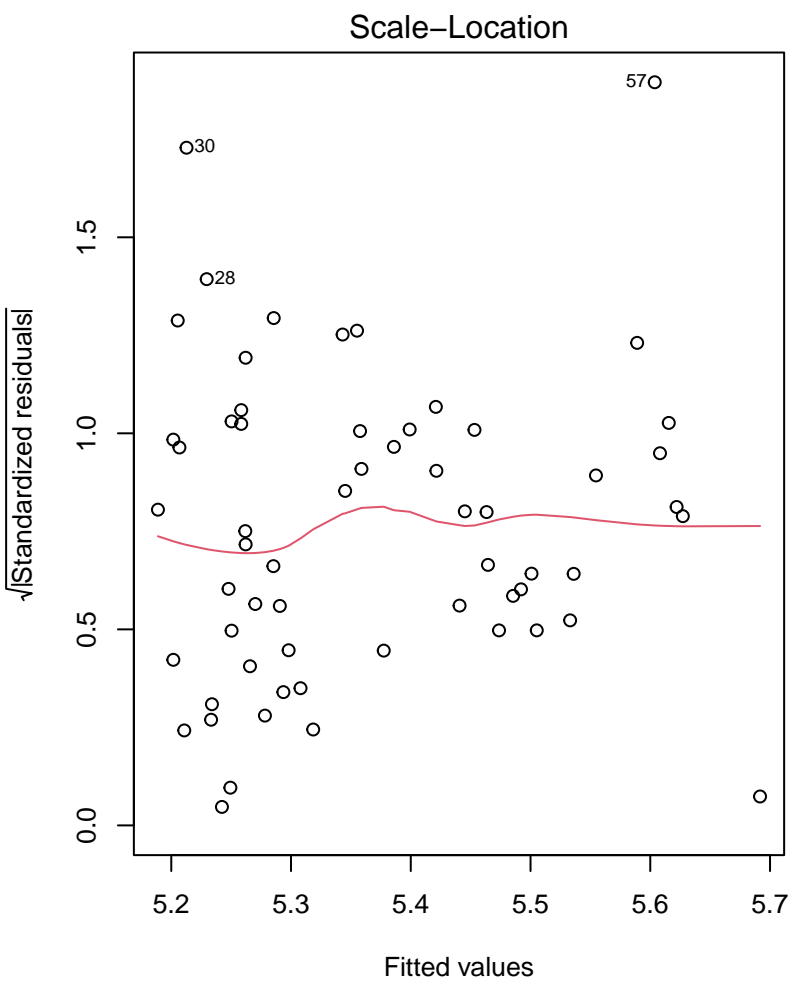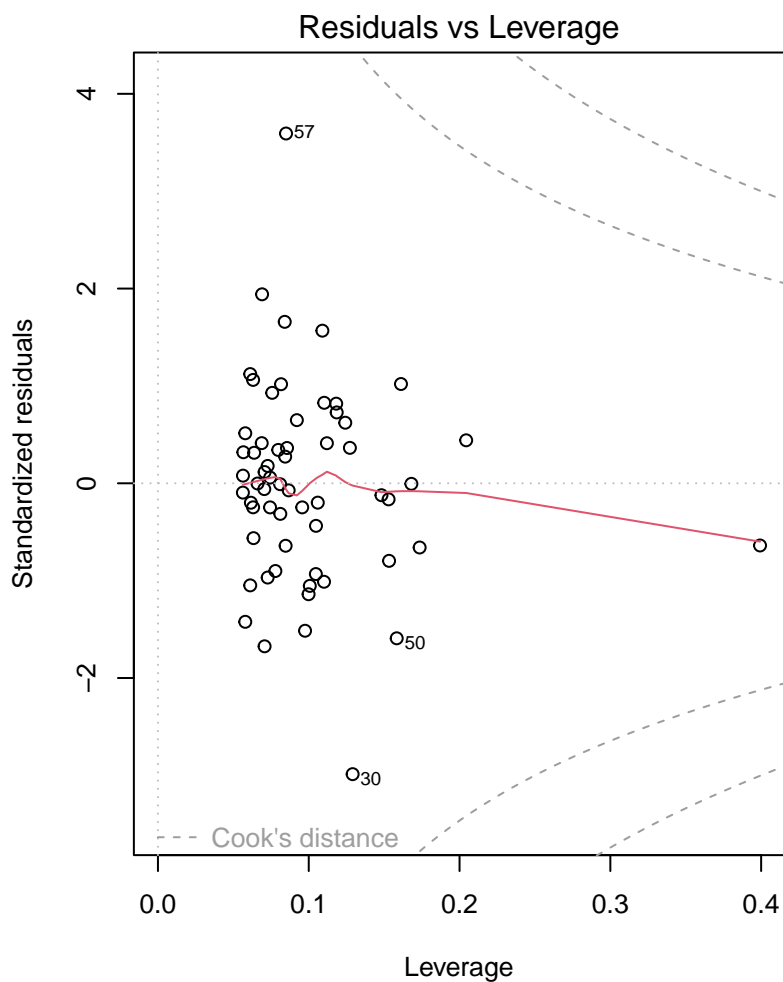

Supplement: Supplementary file 1 [file animals-16-00692-s001.zip › S1_Cholesterol_m2_Group_Sex_Age_Weight.pdf]

lm(DamageScore[1/2][outcome\_log(mel)] ~ GROUP + Sex)

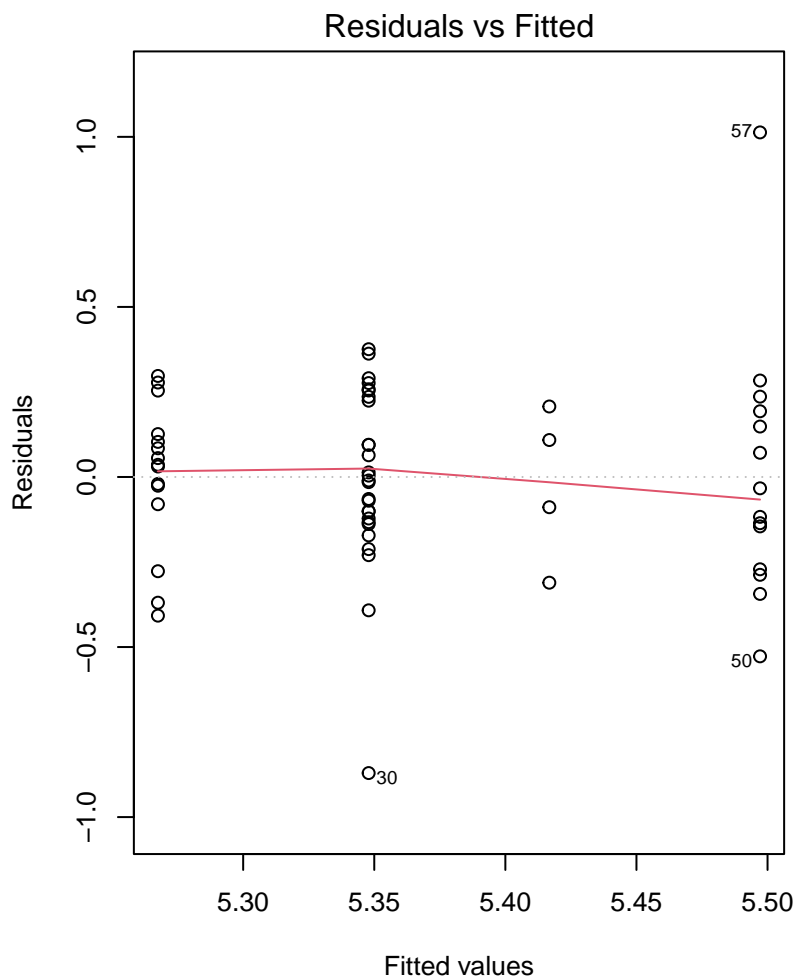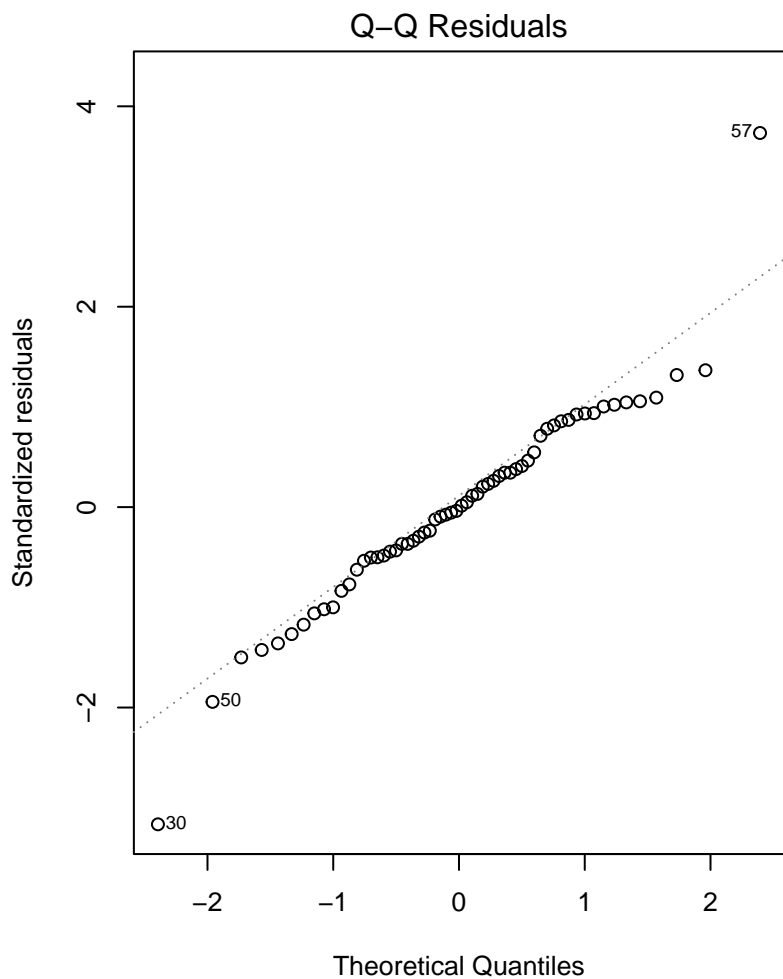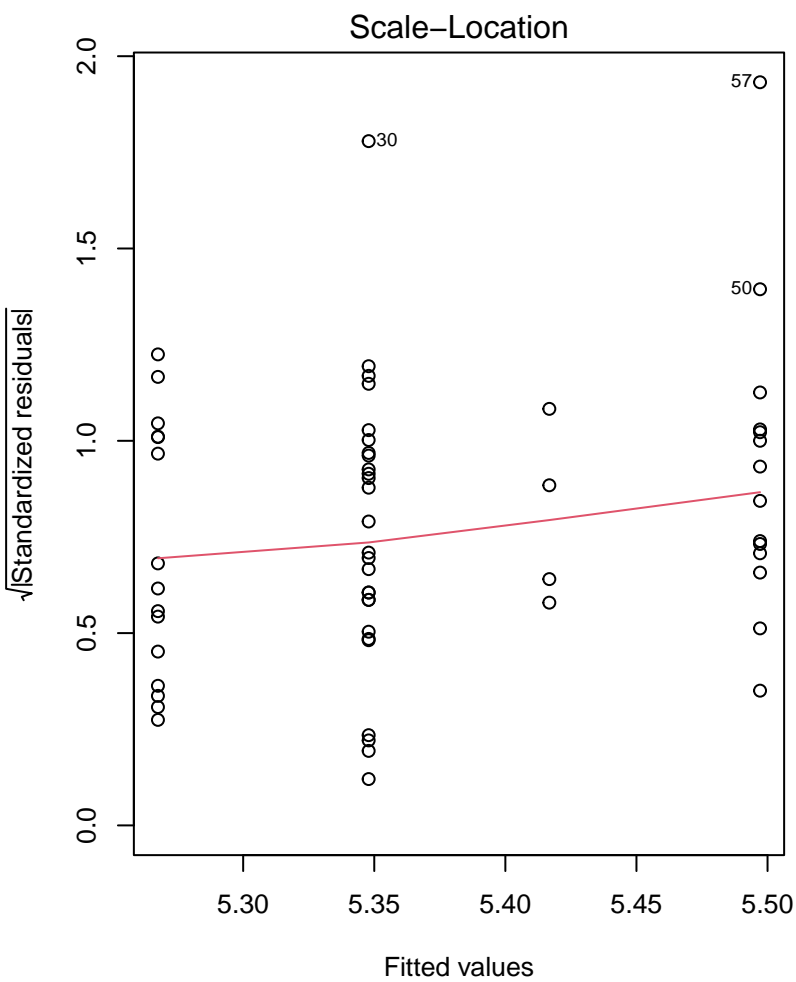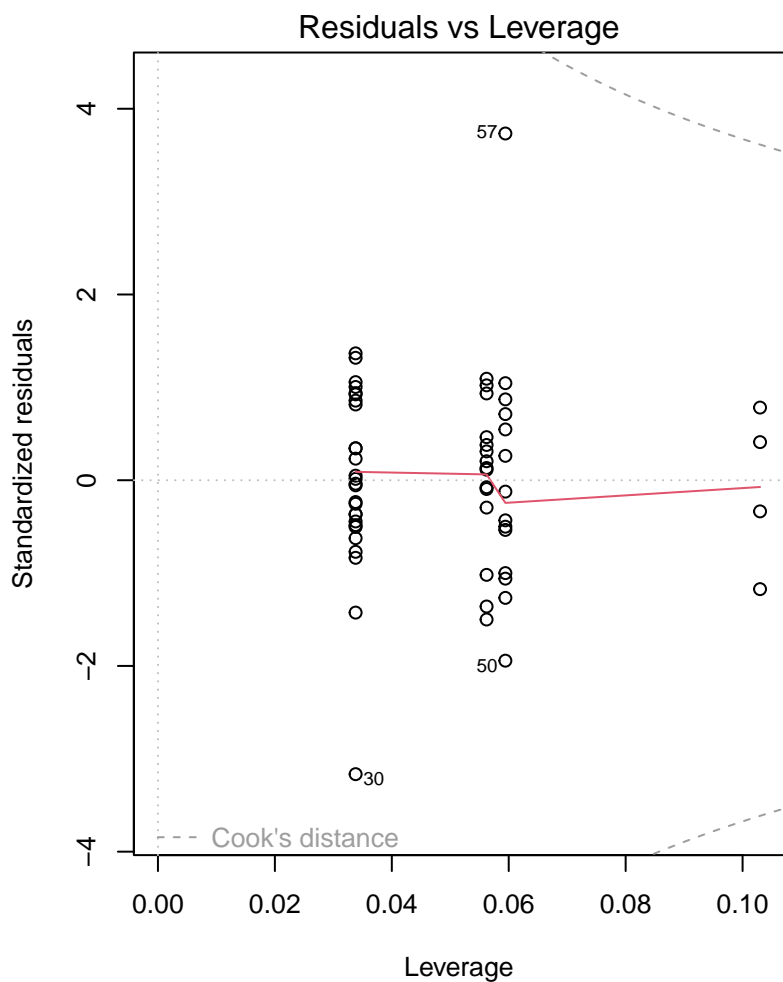

Supplement: Supplementary file 1 [file animals-16-00692-s001.zip › S1_Cholesterol_m3_GROUPII_Sex.pdf]

lm(nake\_response ~ all(outcome\_log(m)) Group Sex Sex)

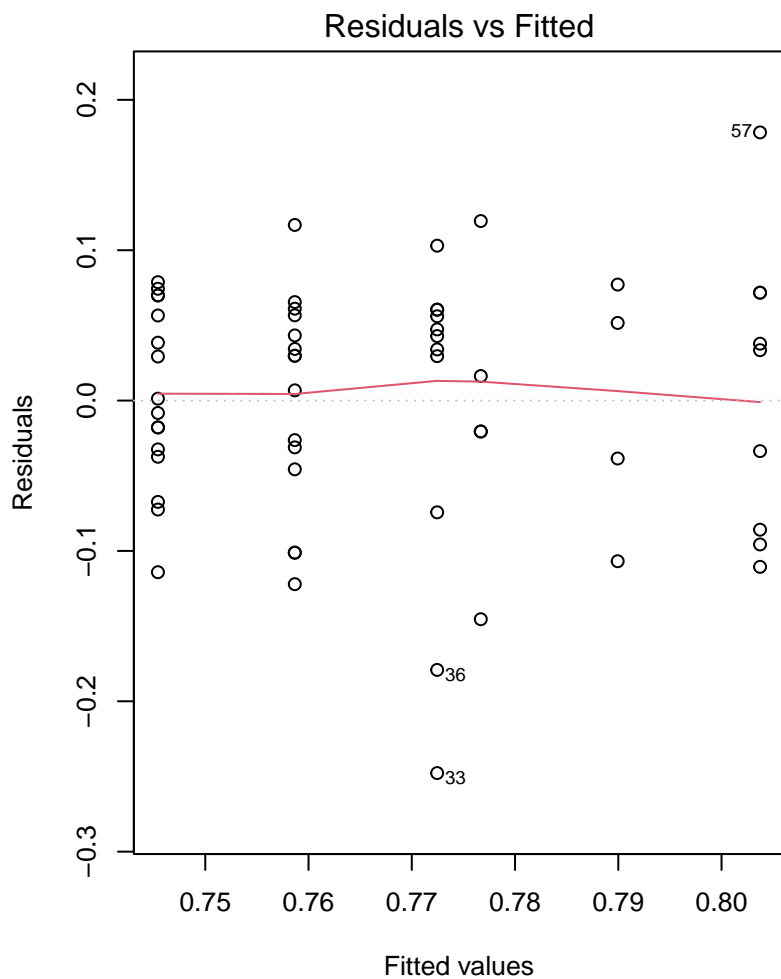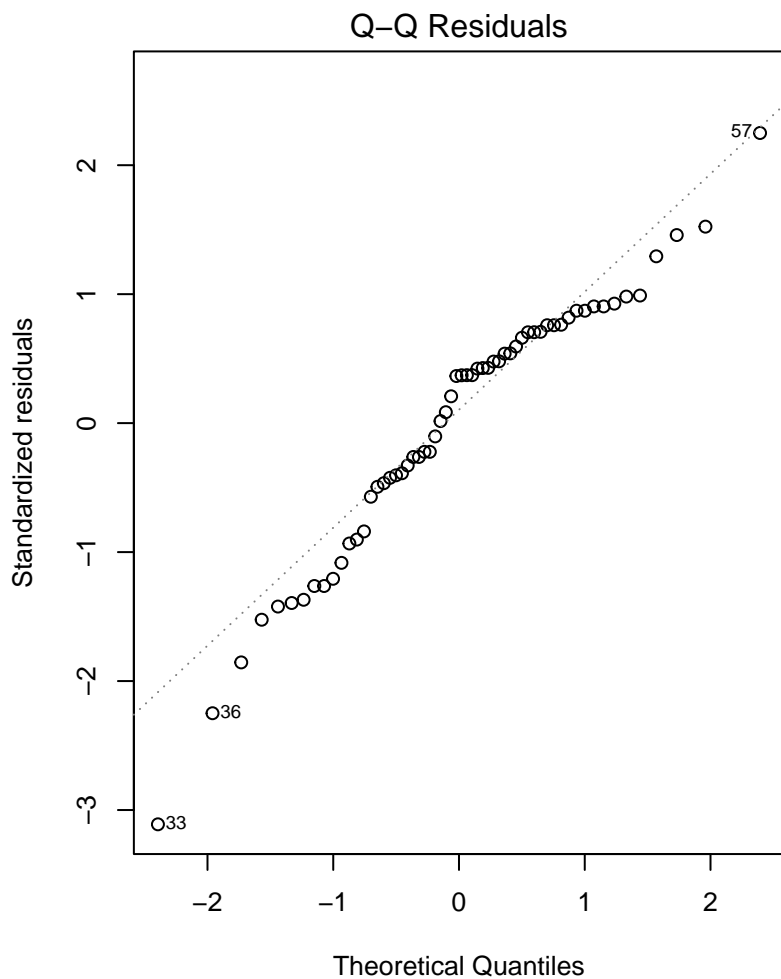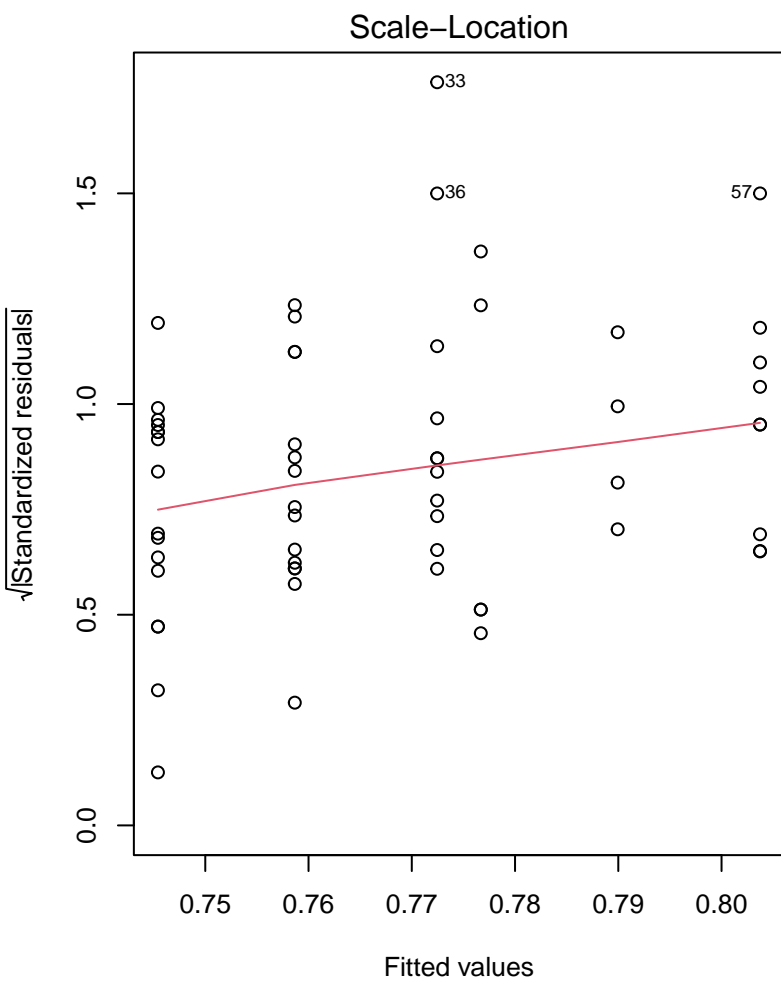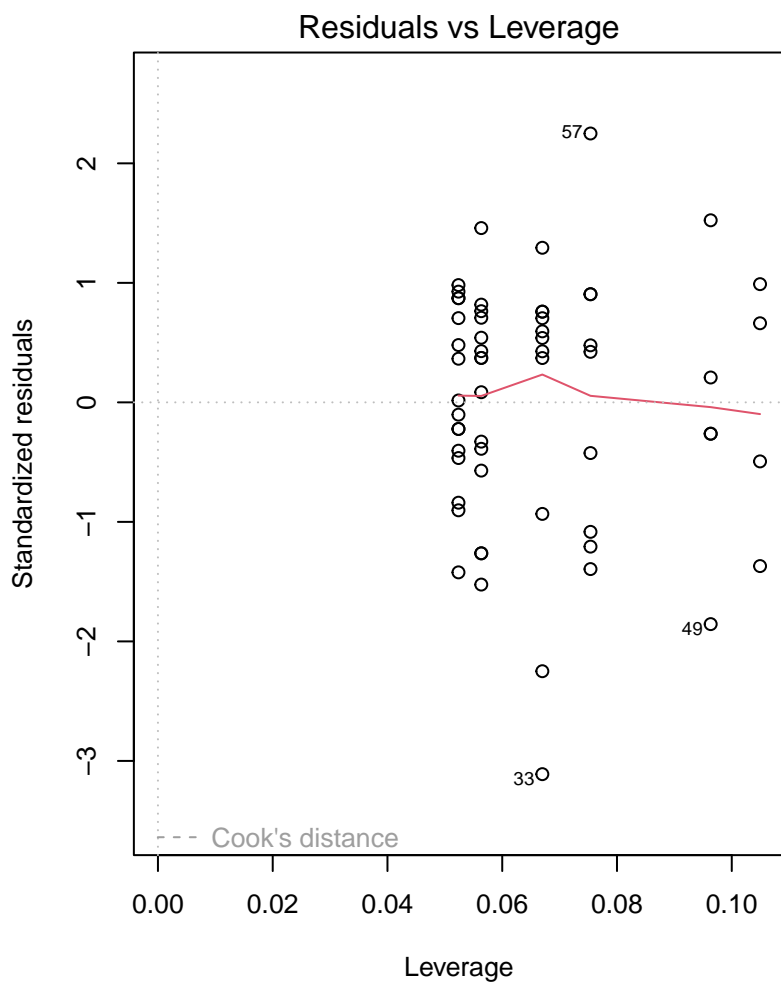

Supplement: Supplementary file 1 [file animals-16-00692-s001.zip › S1_Creatinine_m1_Group_Sex.pdf]

lm(male\_response ~ (12|household\_income)) - Group 3: Sex, Age, Weight

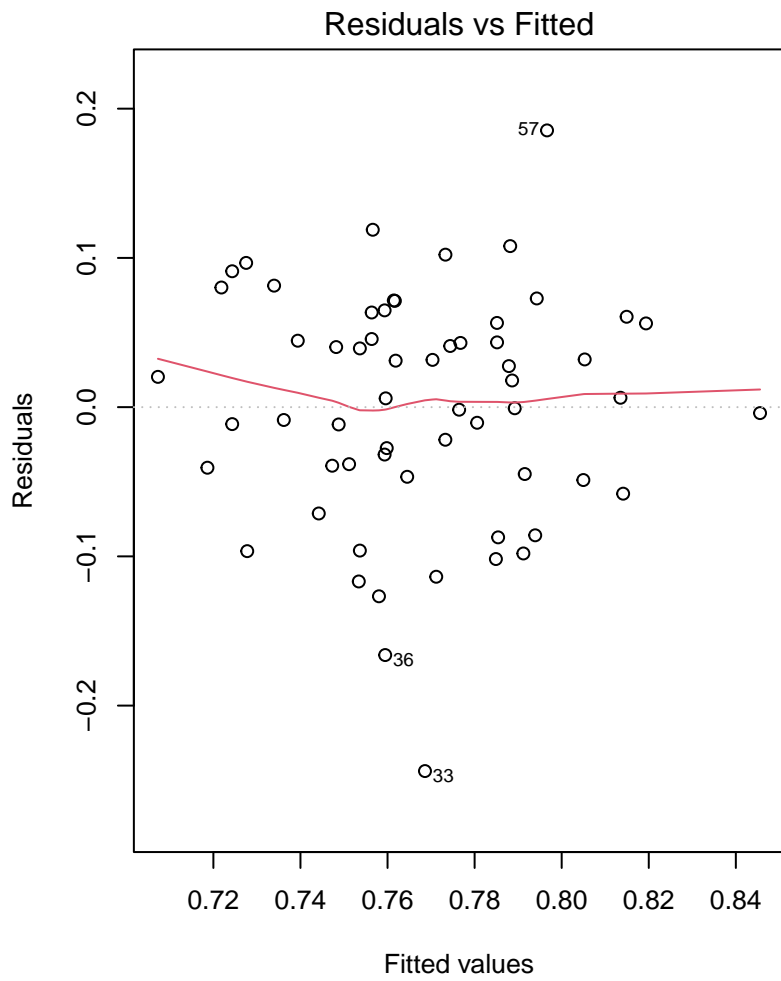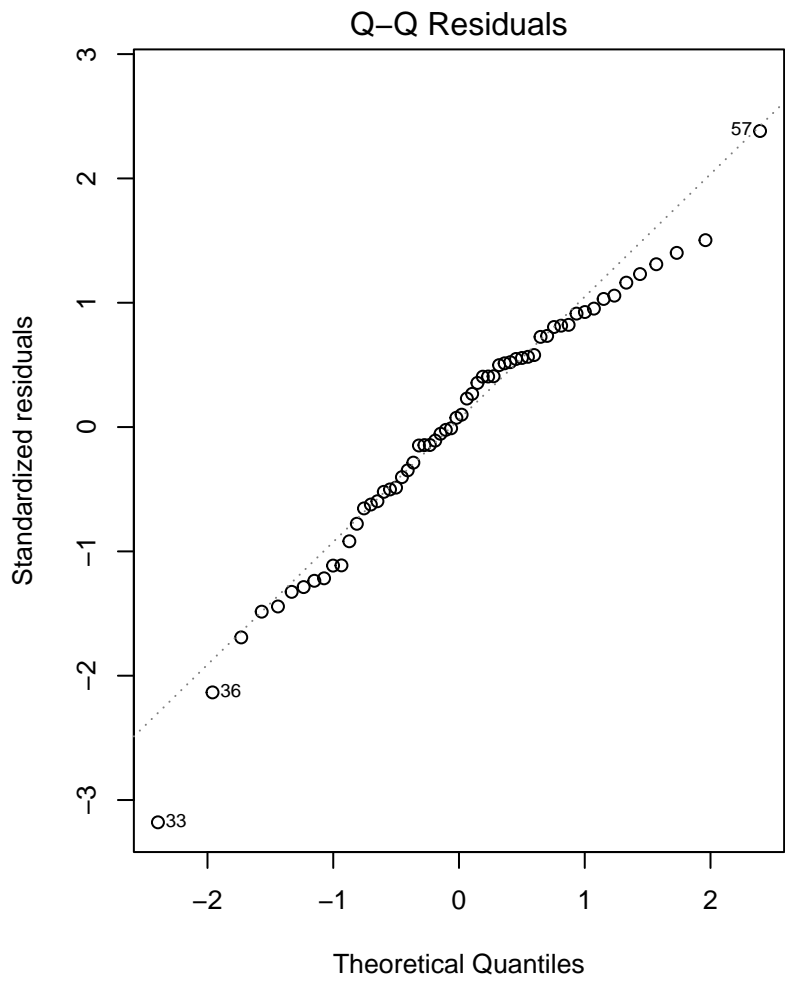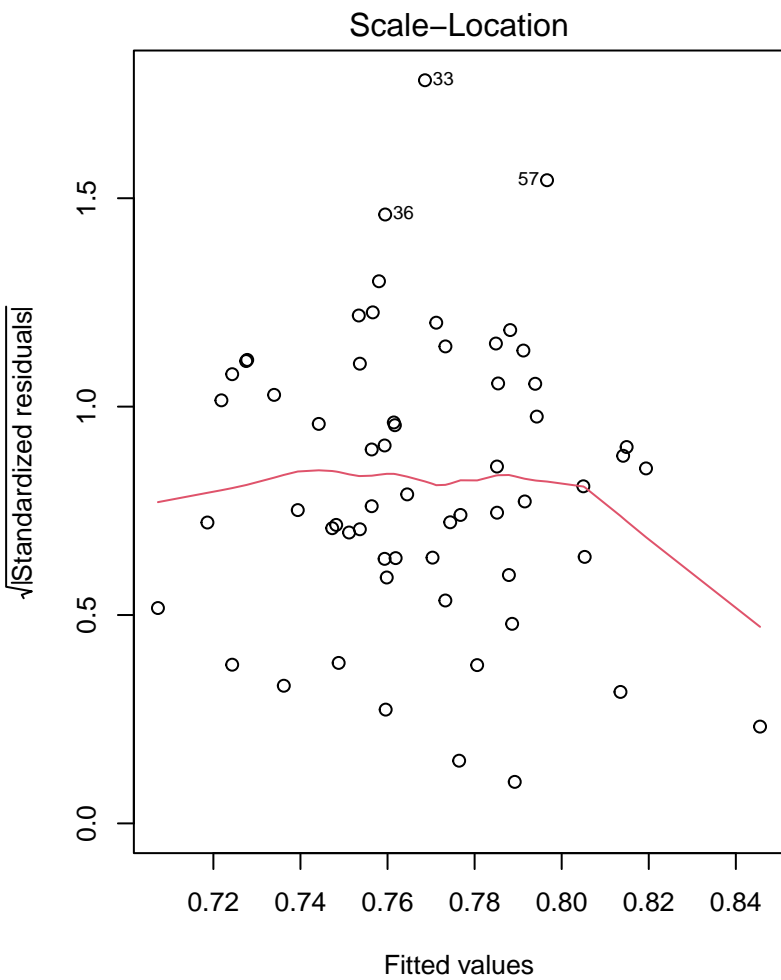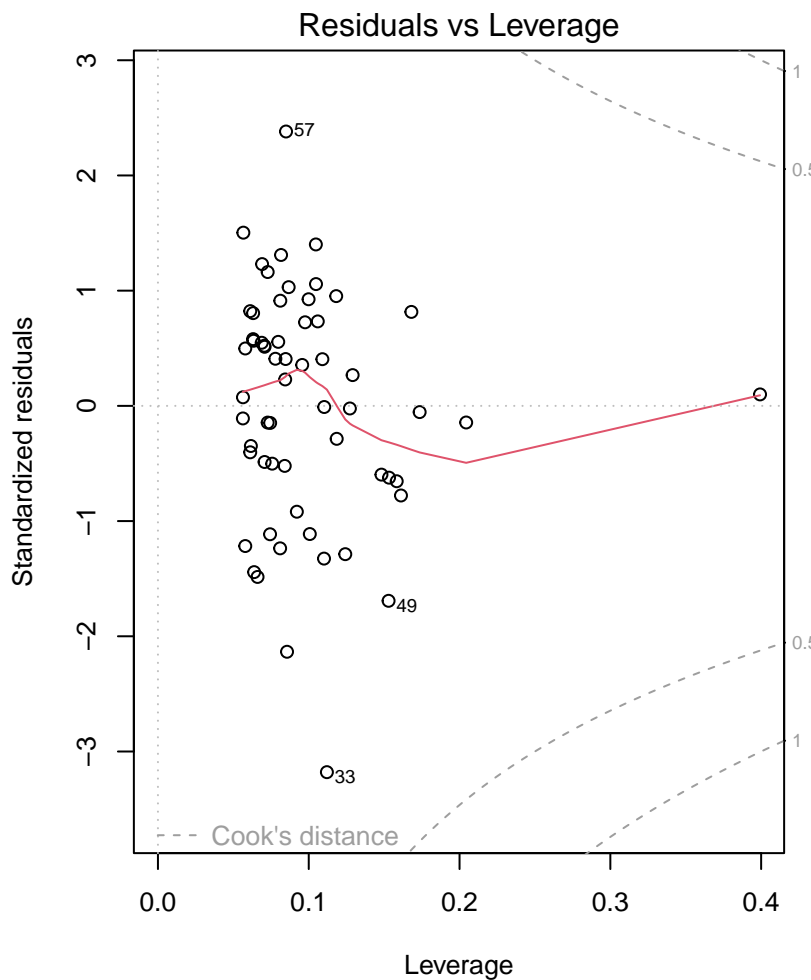

Supplement: Supplementary file 1 [file animals-16-00692-s001.zip › S1_Creatinine_m2_Group_Sex_Age_Weight.pdf]

lm(malignrespnSeed8[[outcome\_logme]] ~ GROUP + Sex, data = data)

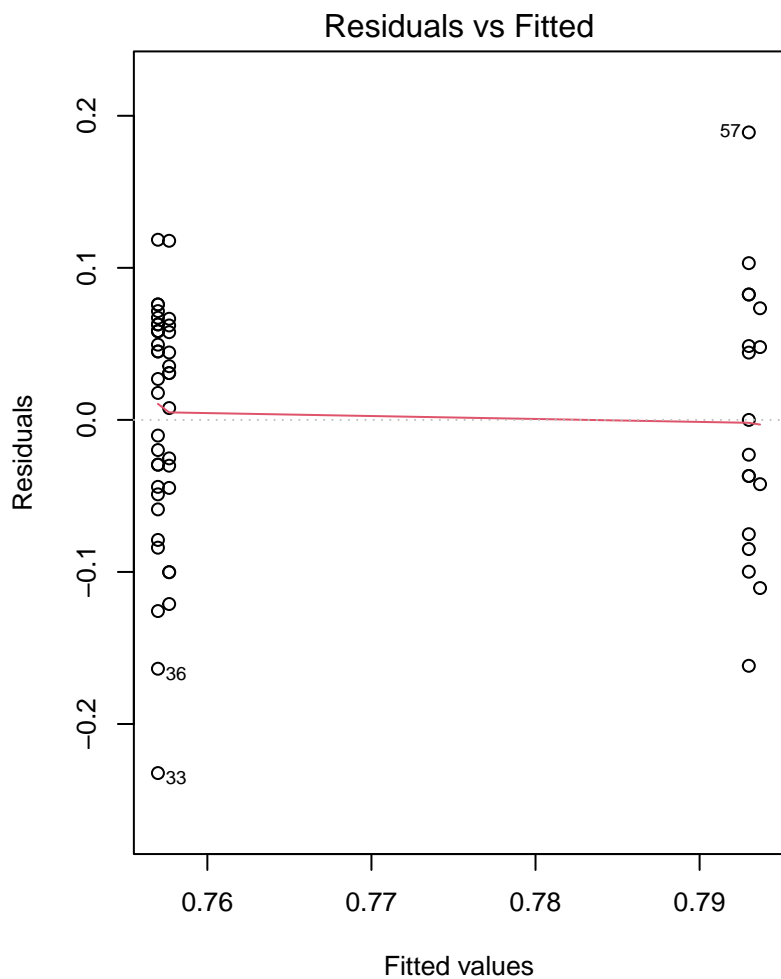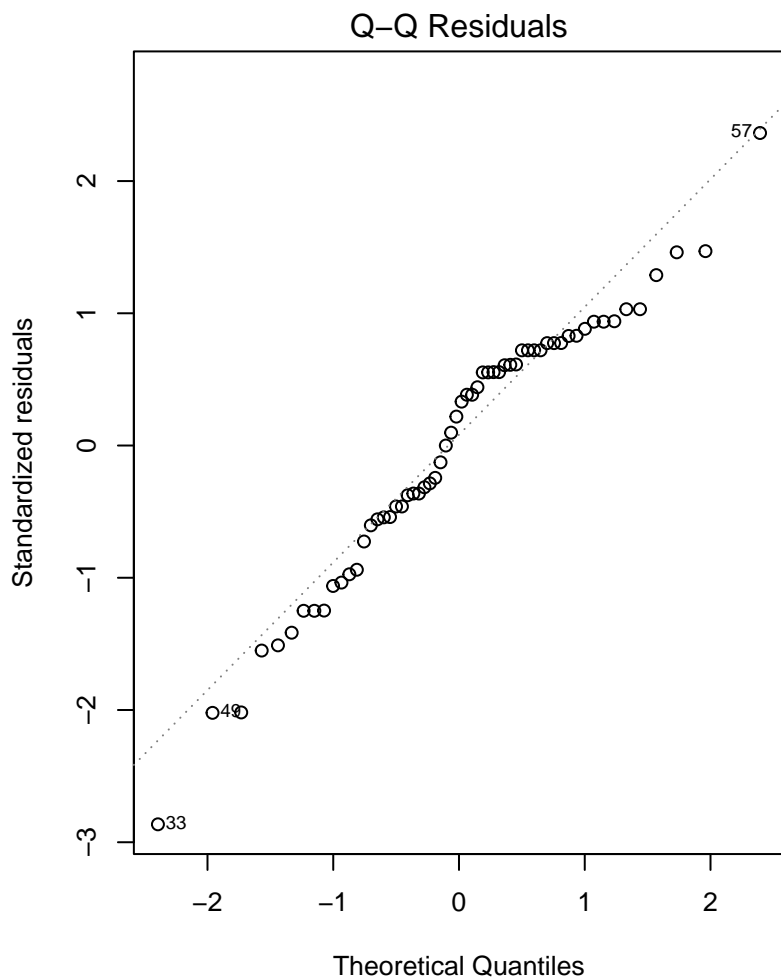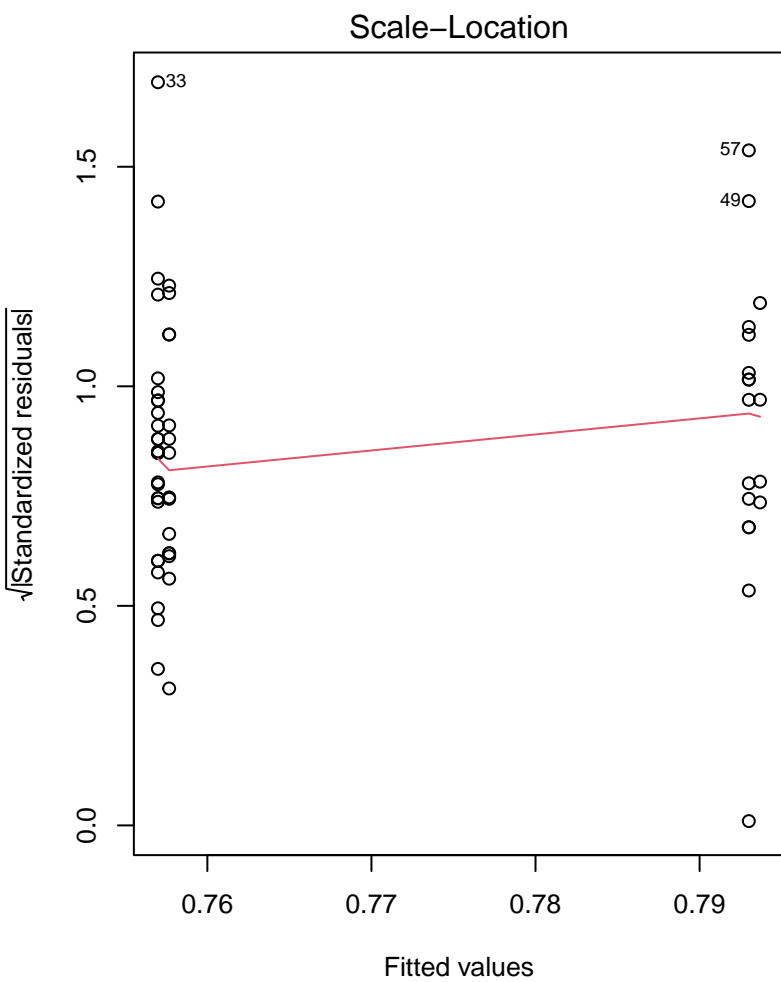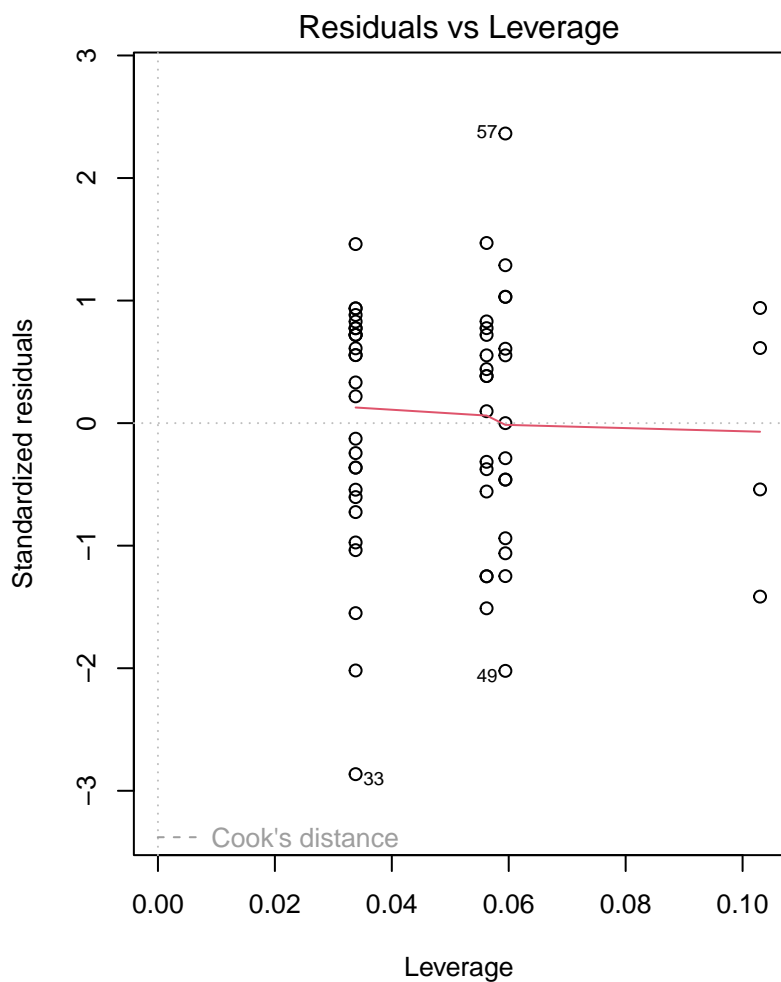

Supplement: Supplementary file 1 [file animals-16-00692-s001.zip › S1_Creatinine_m3_GROUPII_Sex.pdf]

lm(male\_response ~ M\_income + Group + Sex, data = male\_income)

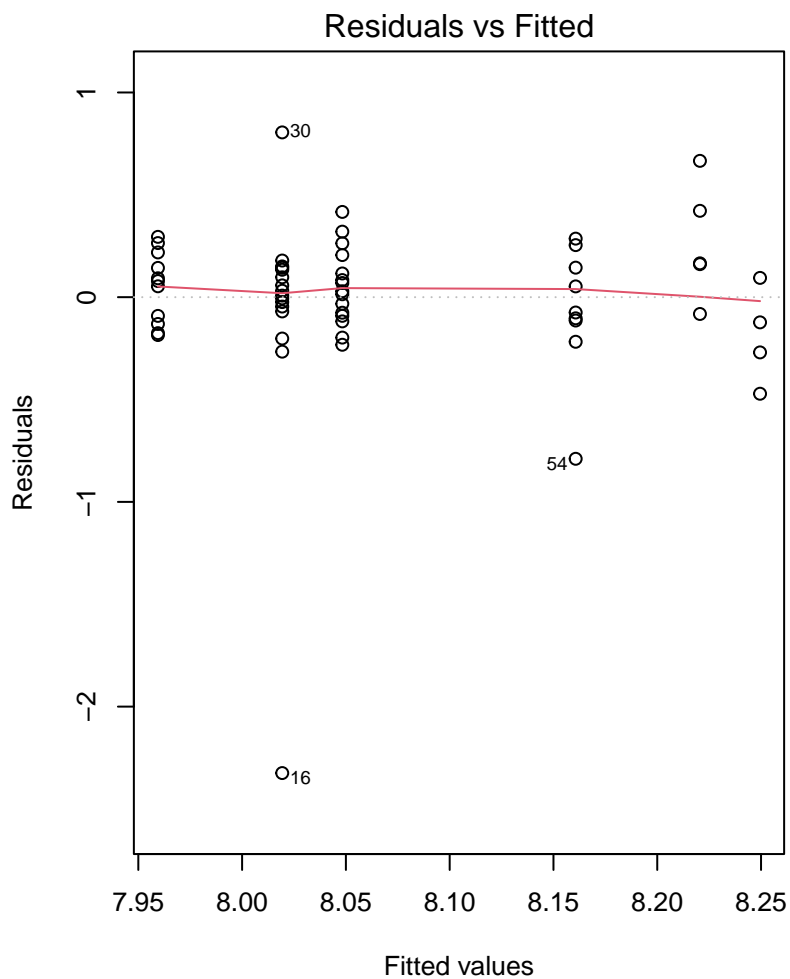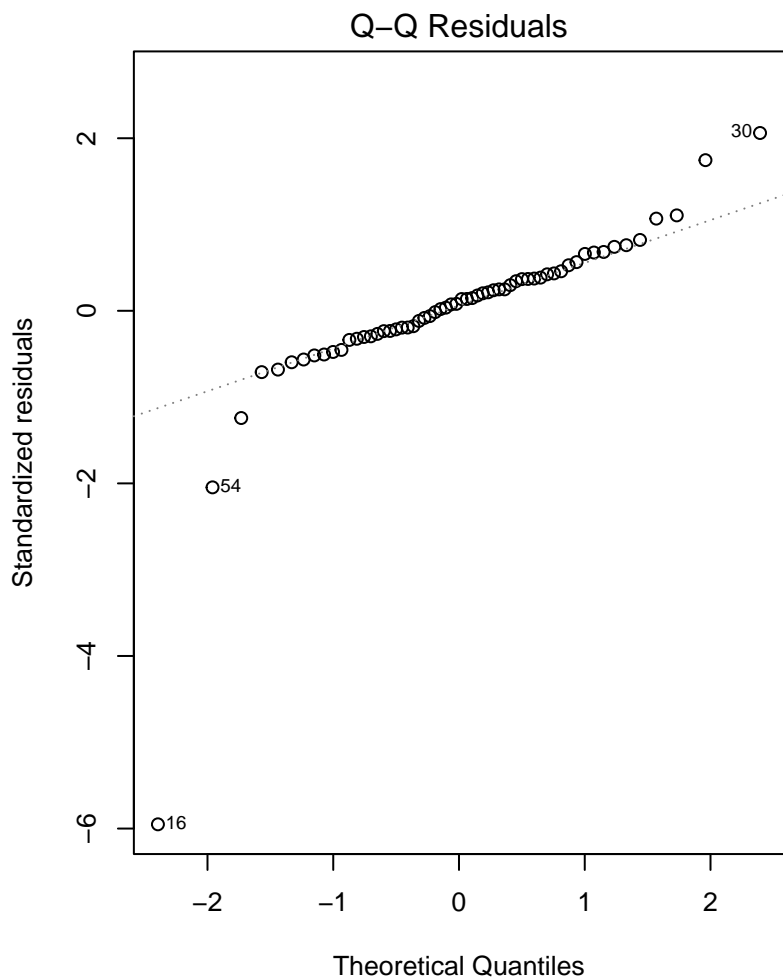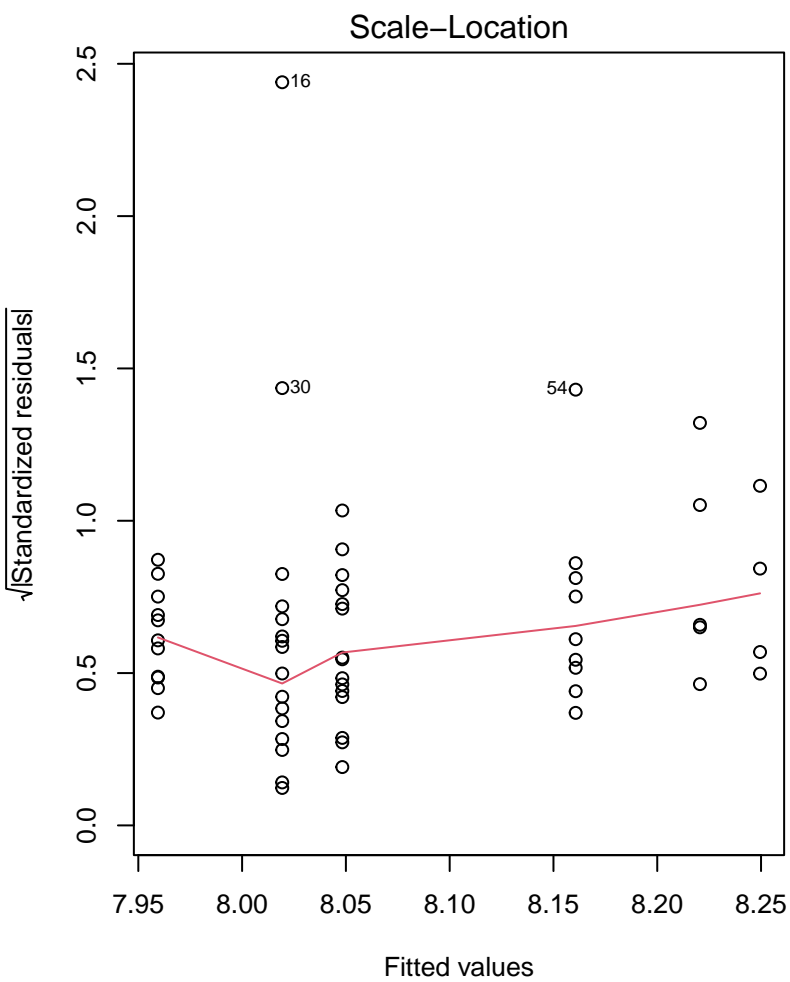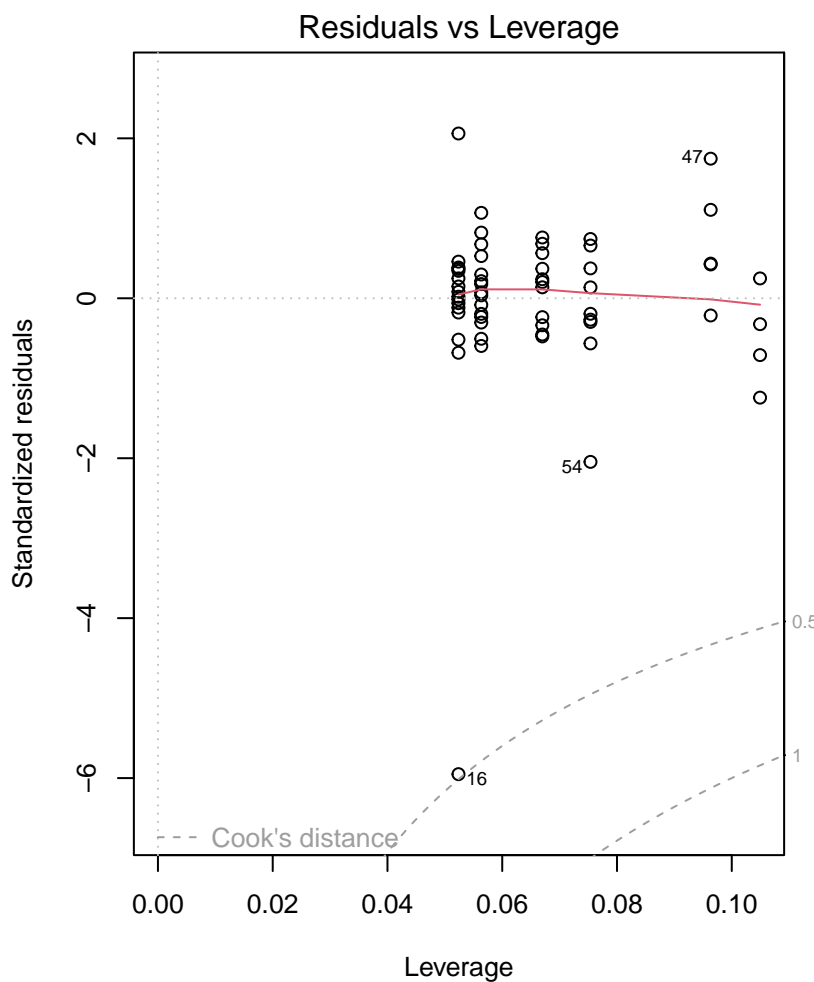

Supplement: Supplementary file 1 [file animals-16-00692-s001.zip › S1_CTX-II_m1_Group_Sex.pdf]

lm(male\_response ~ 0.2 \* (outcome - log(name)) \* Group \* Sex + Age + Weight)

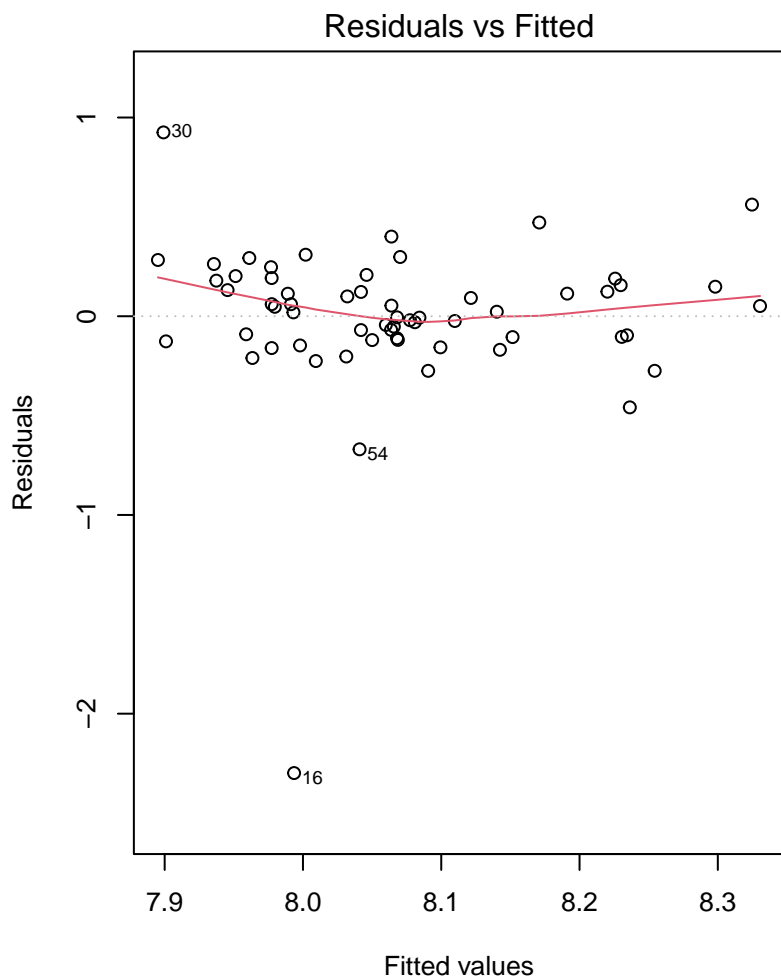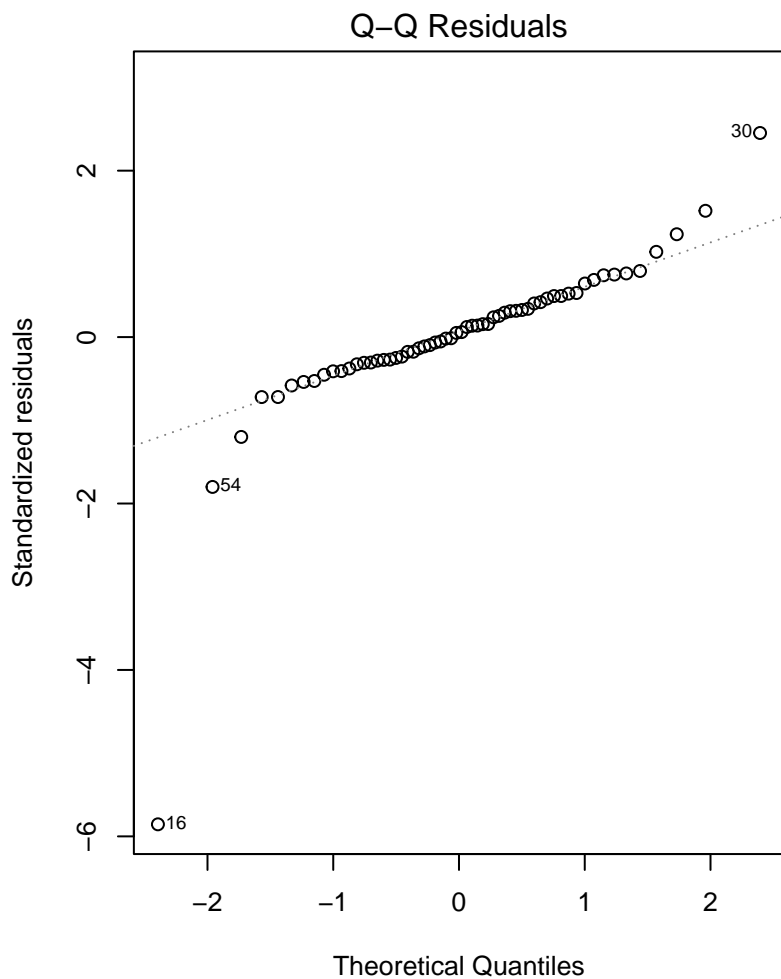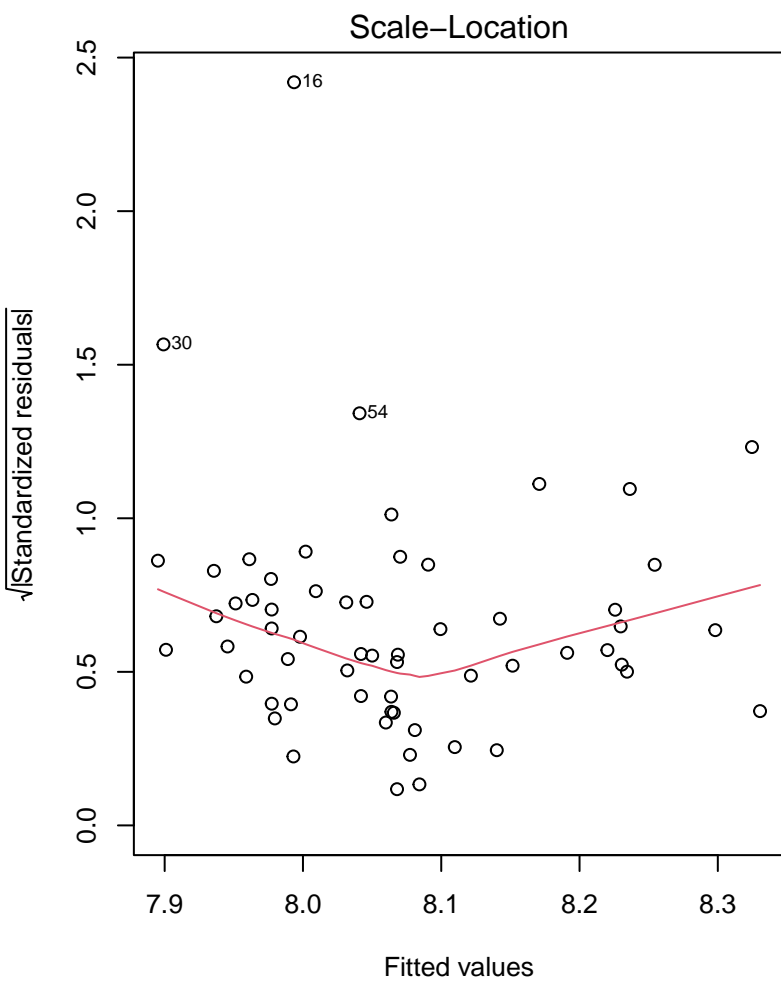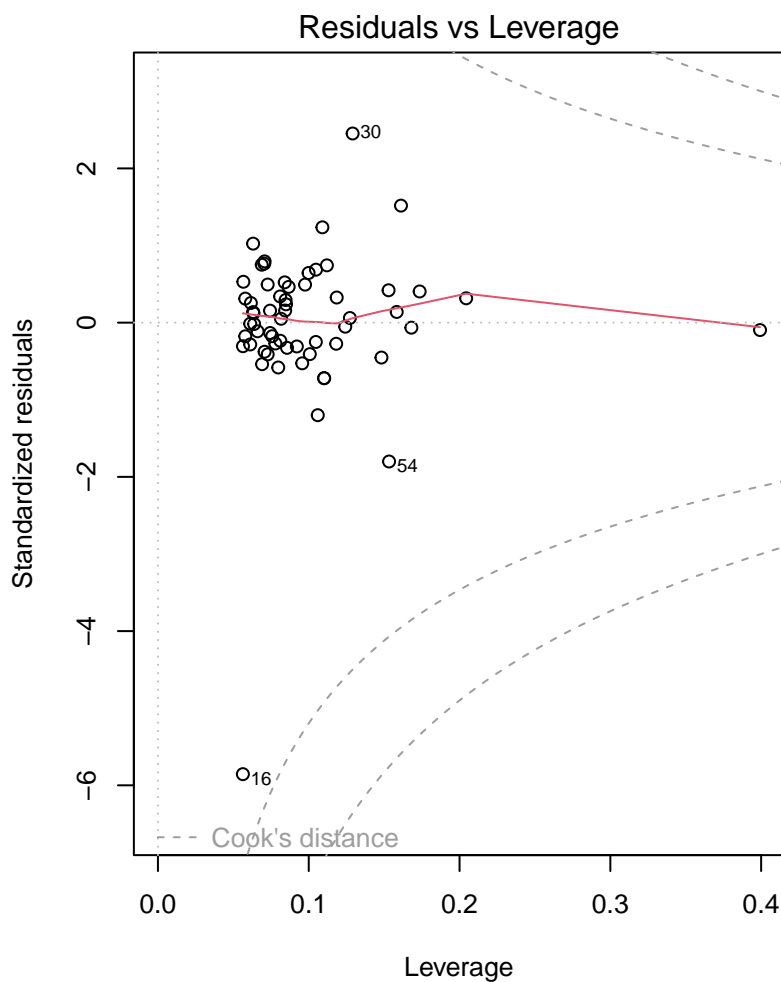

Supplement: Supplementary file 1 [file animals-16-00692-s001.zip › S1_CTX-II_m2_Group_Sex_Age_Weight.pdf]

lm(male\_gross\_pay[0:1][outcome\_name]) ~ GROUP Sex+ Sex)

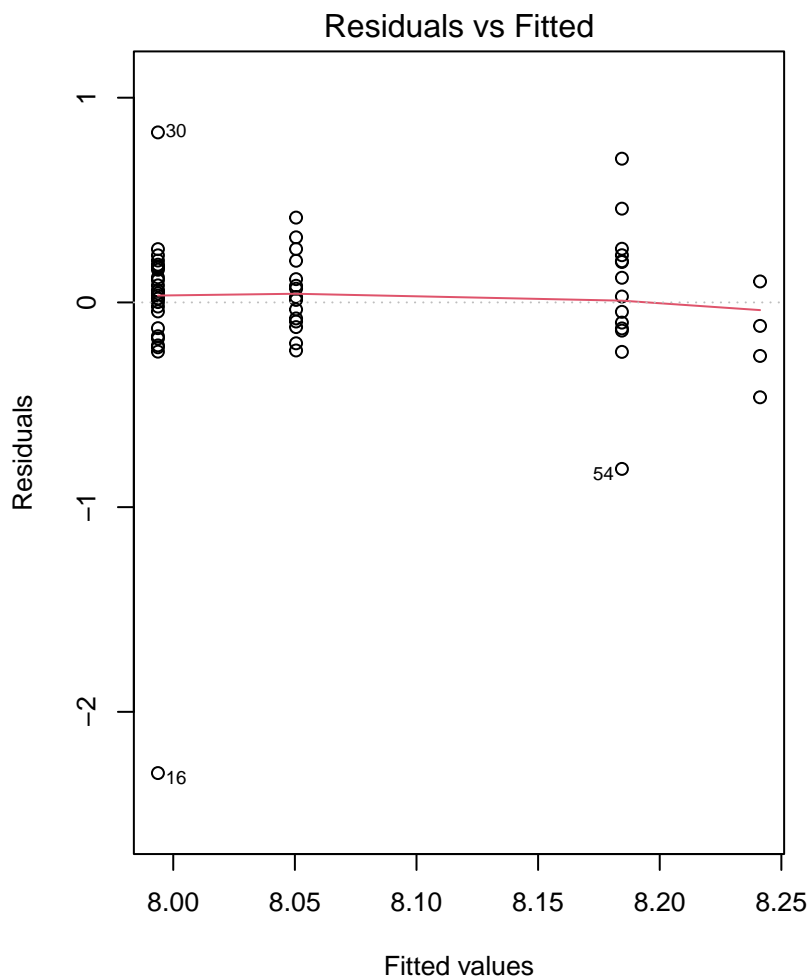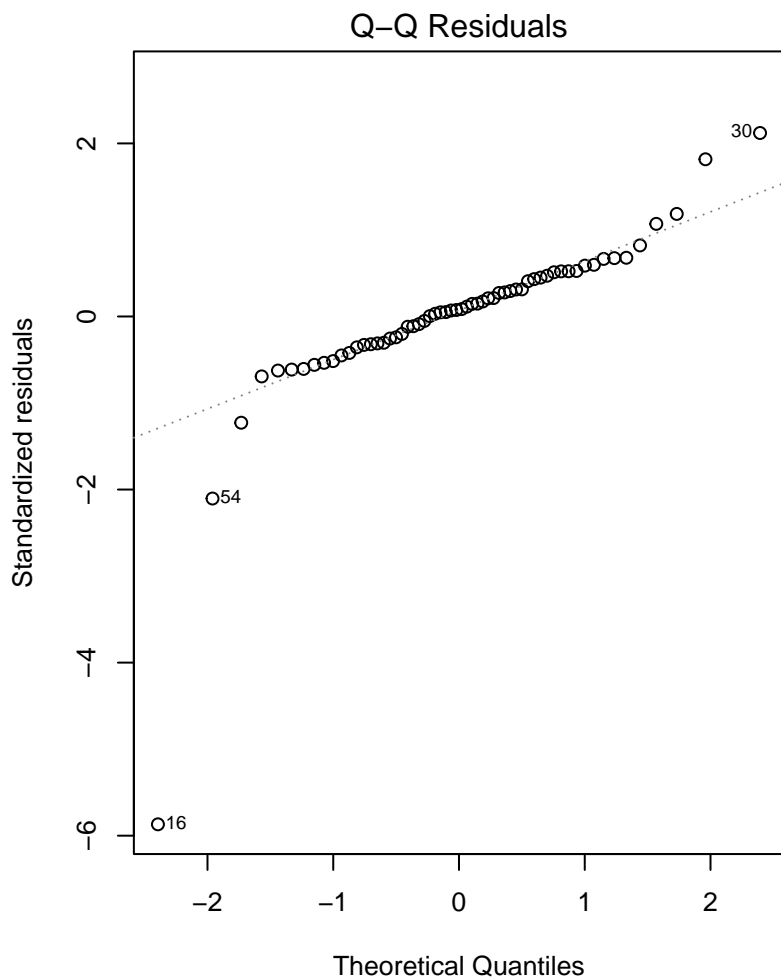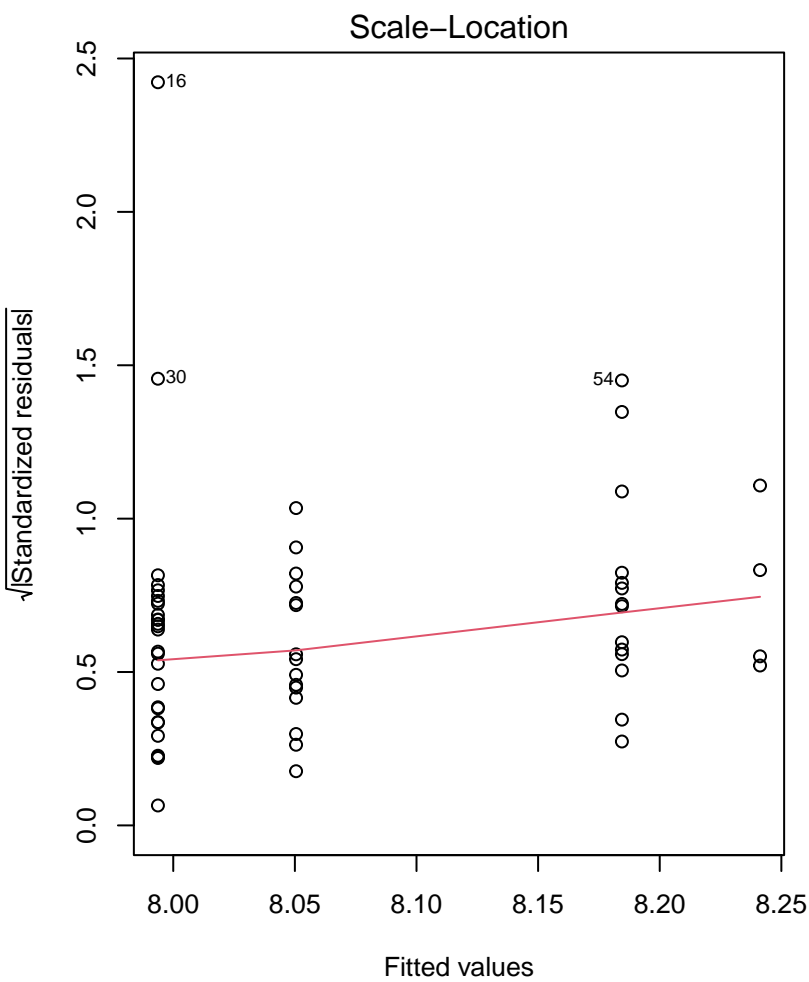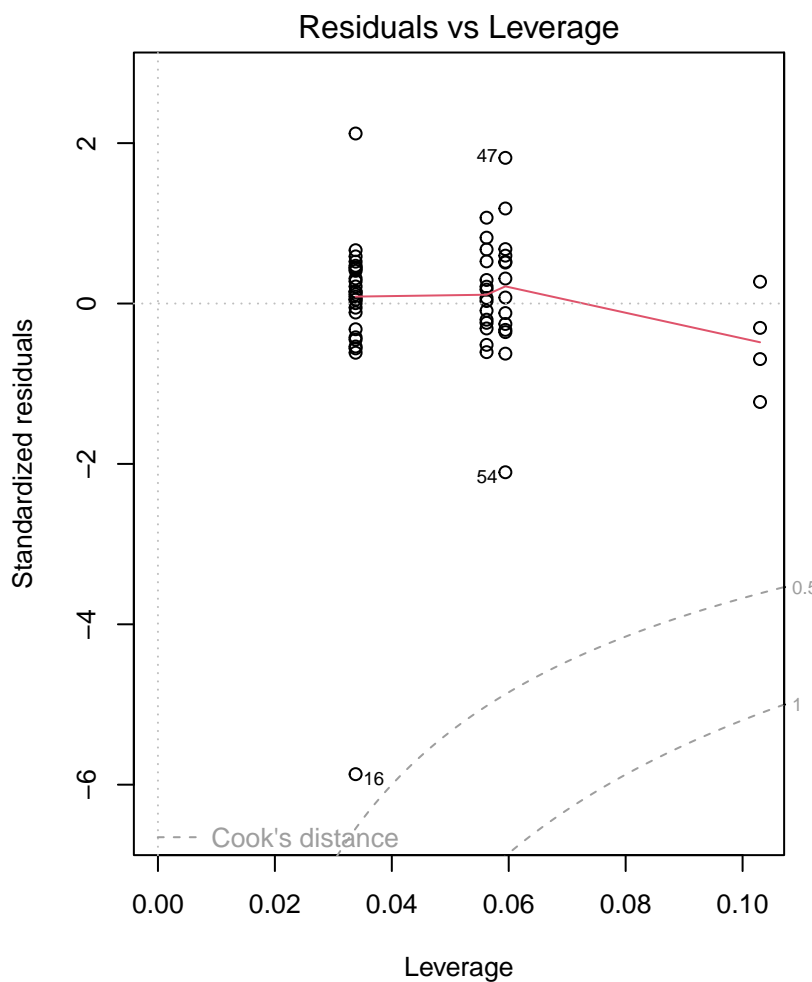

Supplement: Supplementary file 1 [file animals-16-00692-s001.zip › S1_CTX-II_m3_GROUPII_Sex.pdf]

lm(make\_response ~ G1 + (outcome\_name) + G2 + Sex)

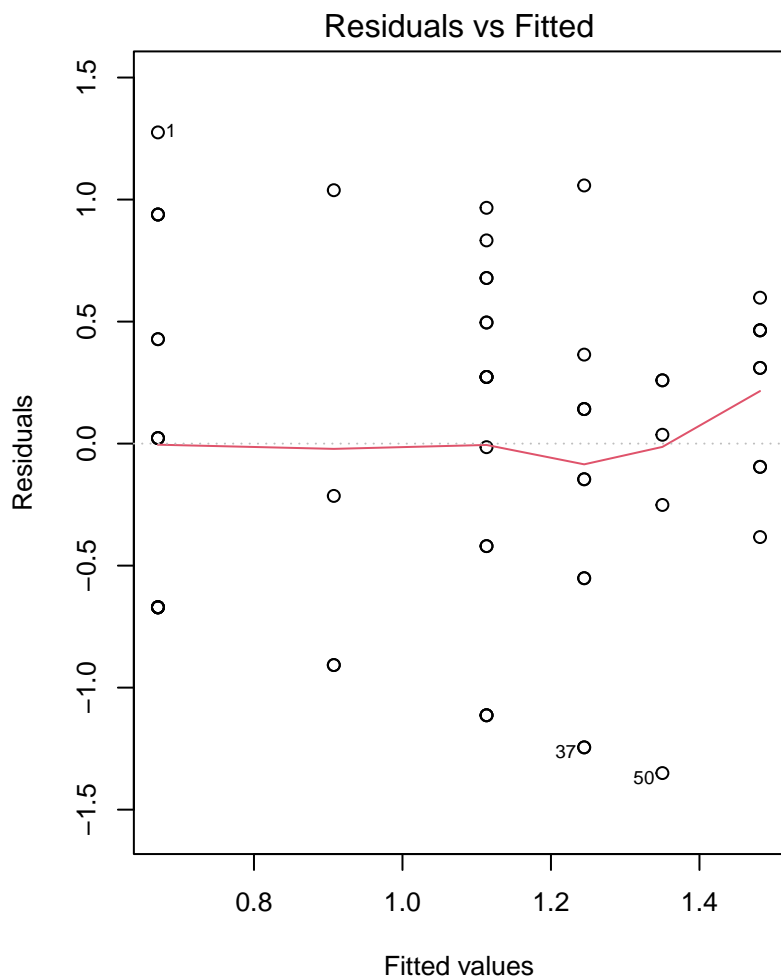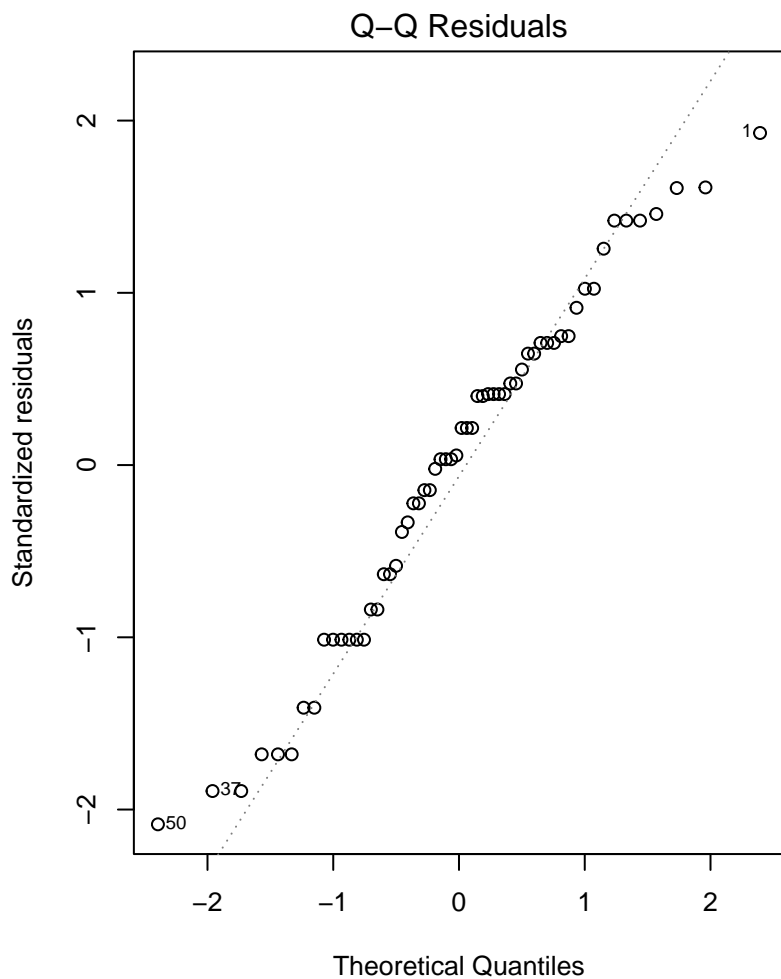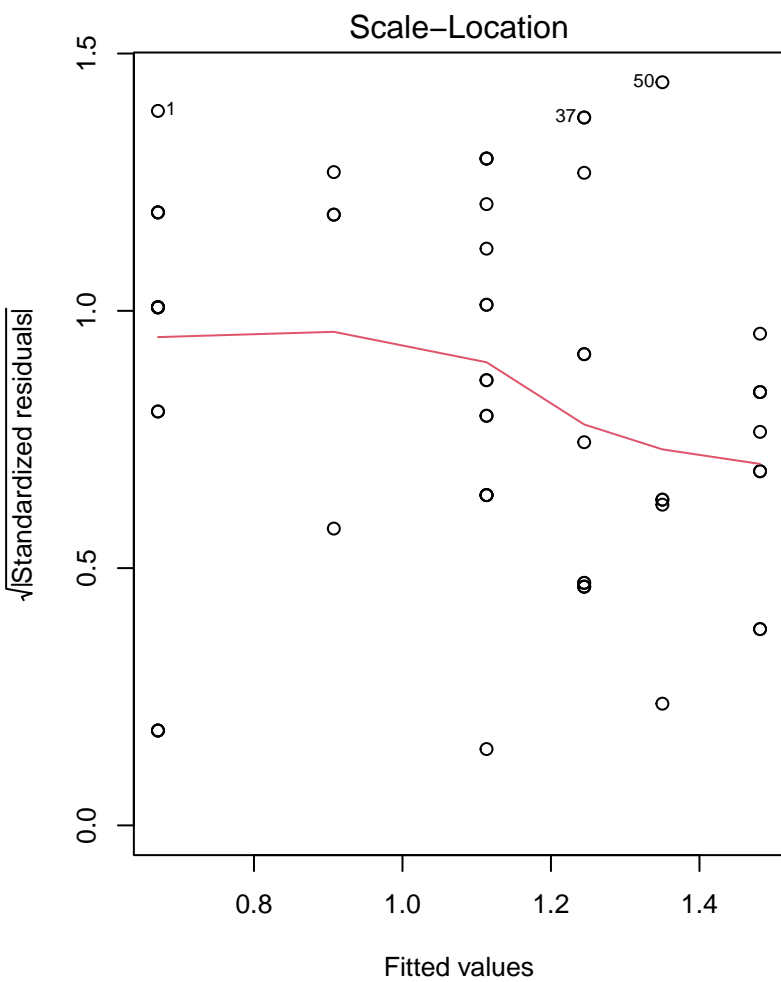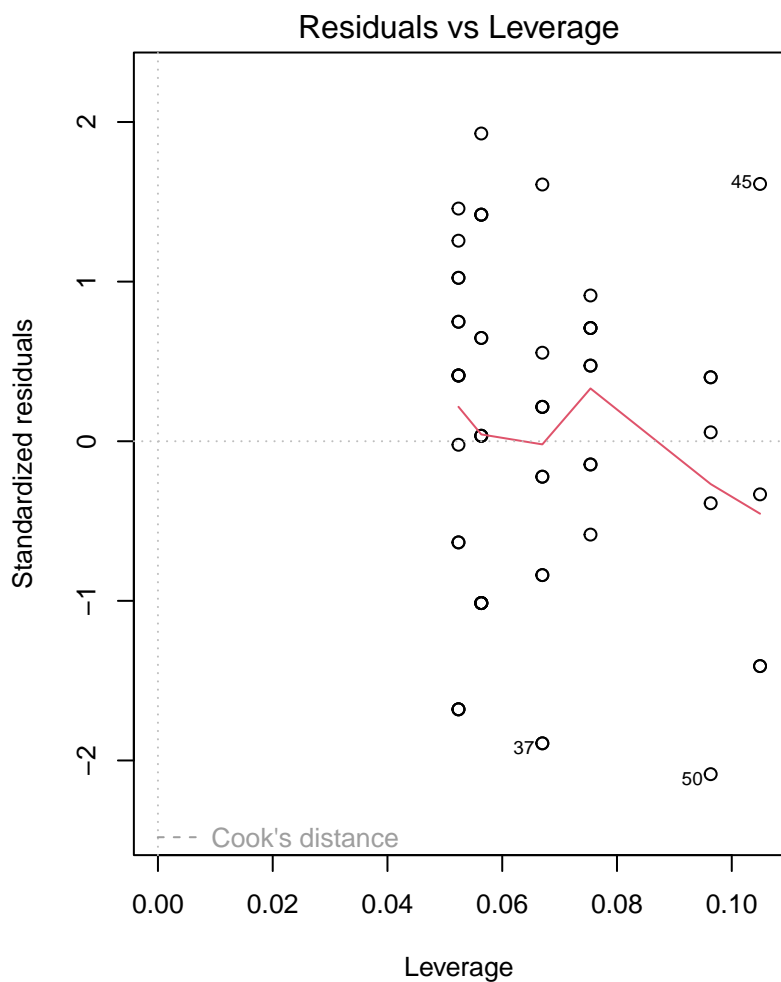

Supplement: Supplementary file 1 [file animals-16-00692-s001.zip › S1_GGT_m1_Group_Sex.pdf]

lm(make\_response(G2[output == "name"]) ~ Group + Sex + Age + Weight)

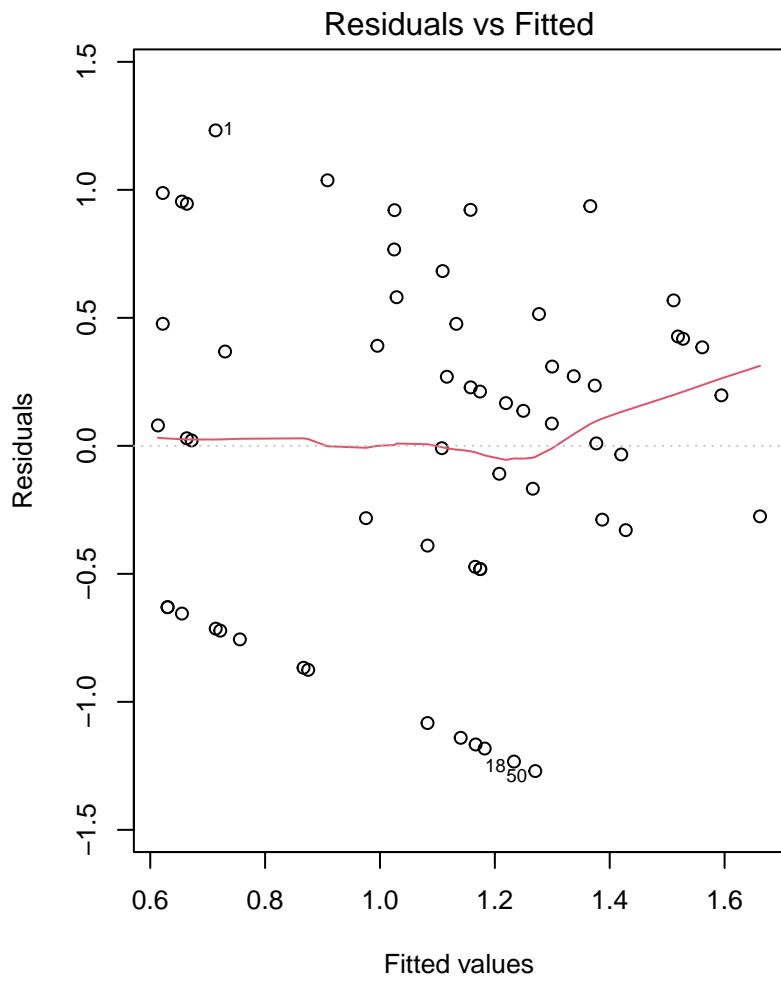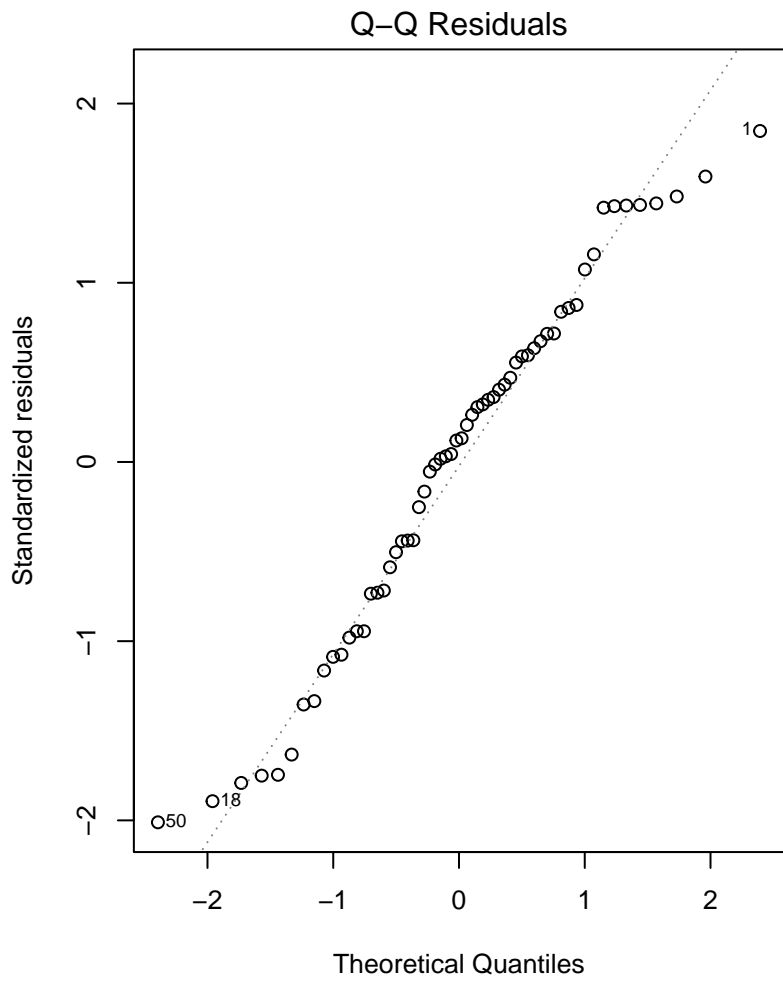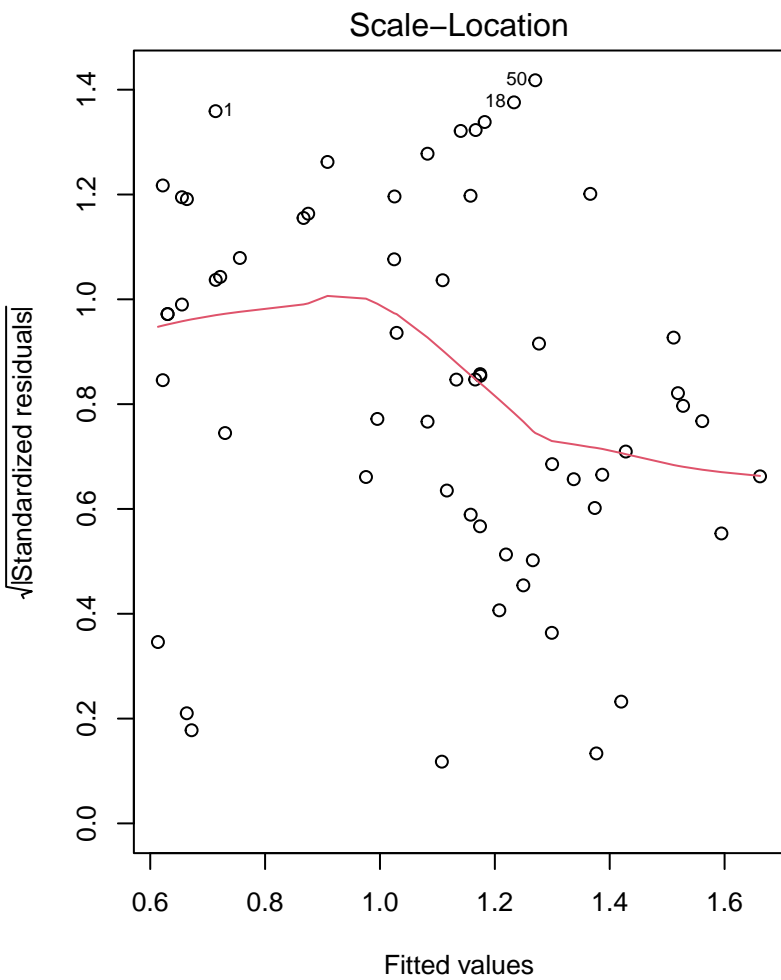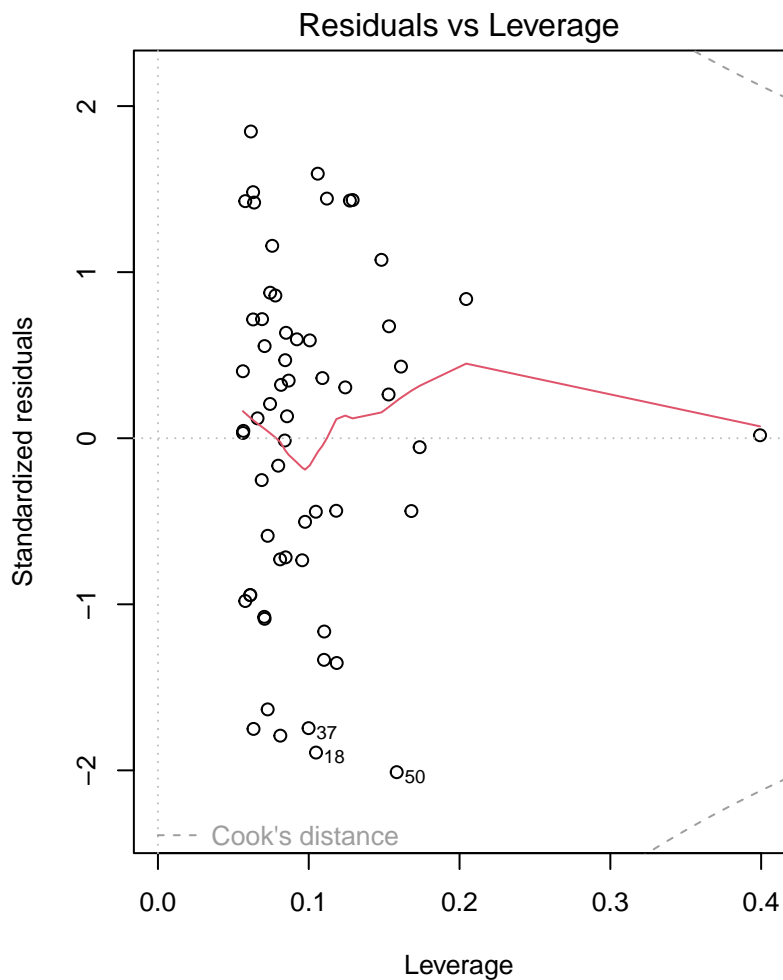

Supplement: Supplementary file 1 [file animals-16-00692-s001.zip › S1_GGT_m2_Group_Sex_Age_Weight.pdf]

lm(makDiagnose(d3[[outcome\_name]]\$GROUPS\_Sex))

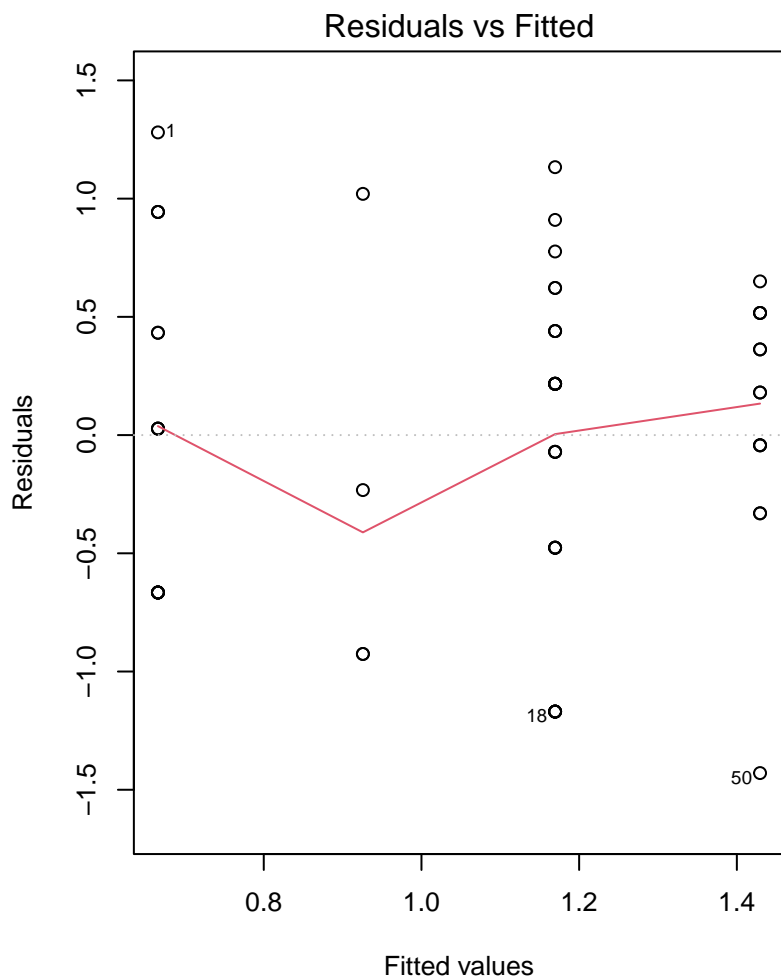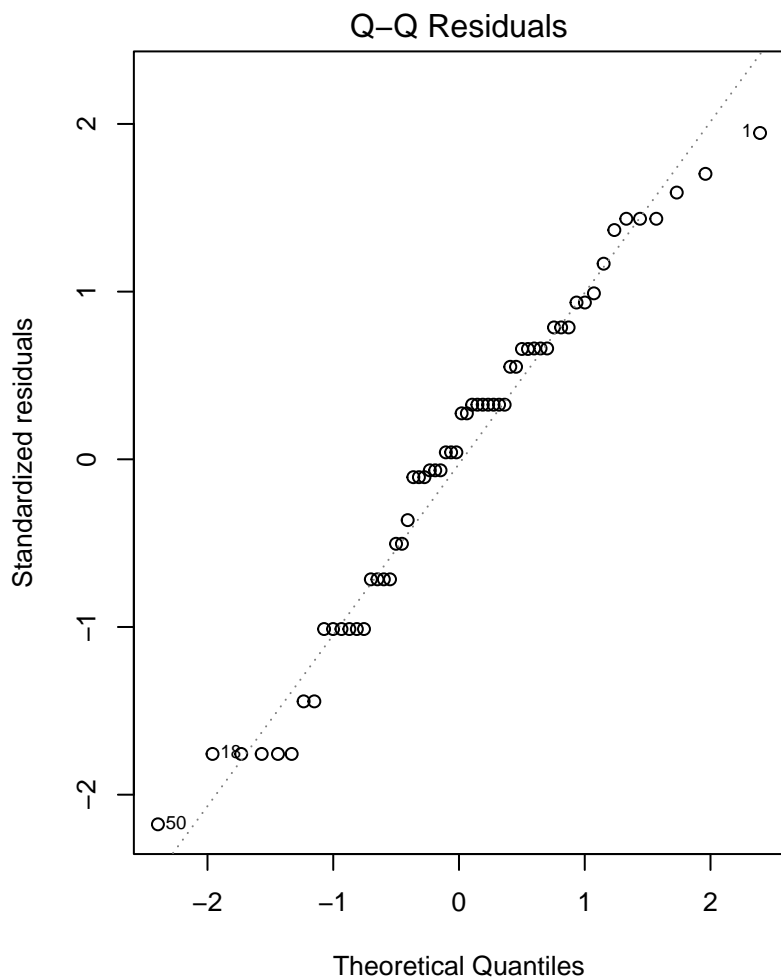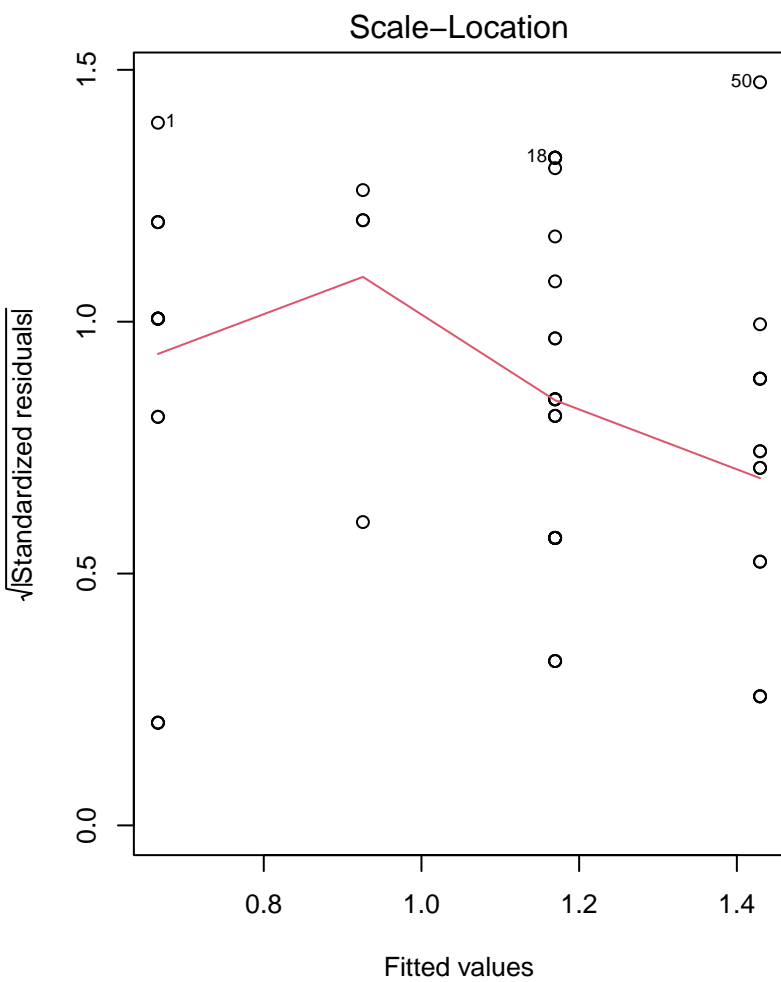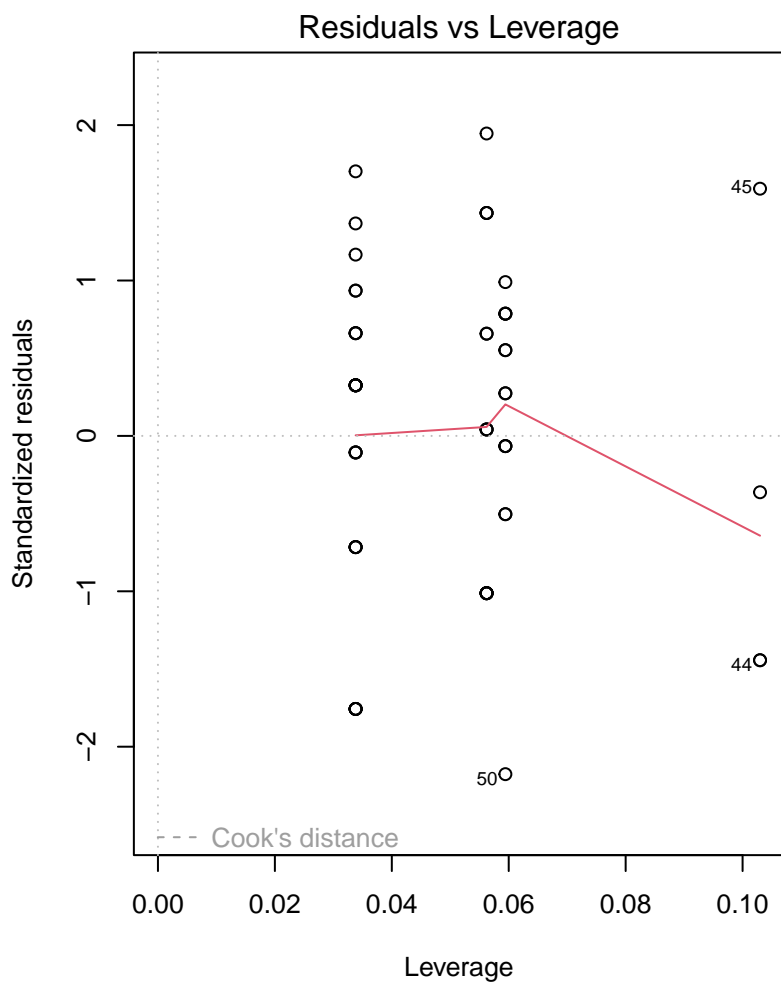

Supplement: Supplementary file 1 [file animals-16-00692-s001.zip › S1_GGT_m3_GROUPII_Sex.pdf]

lm(male response ~ (1 + sex) \* log(age)) Group Sex

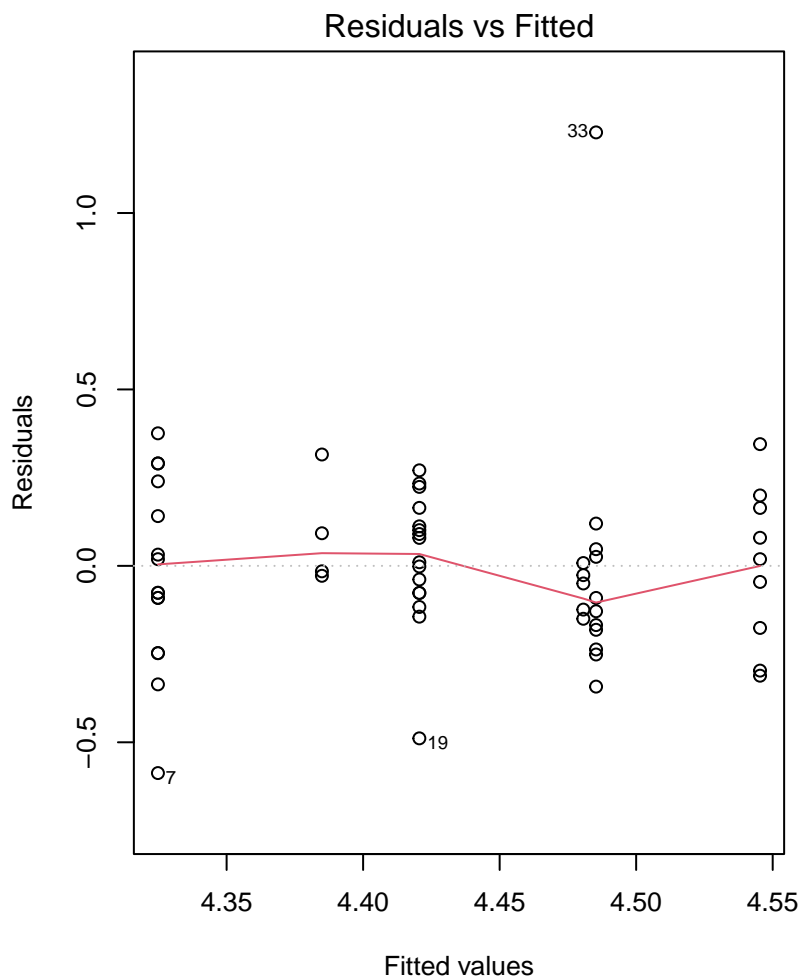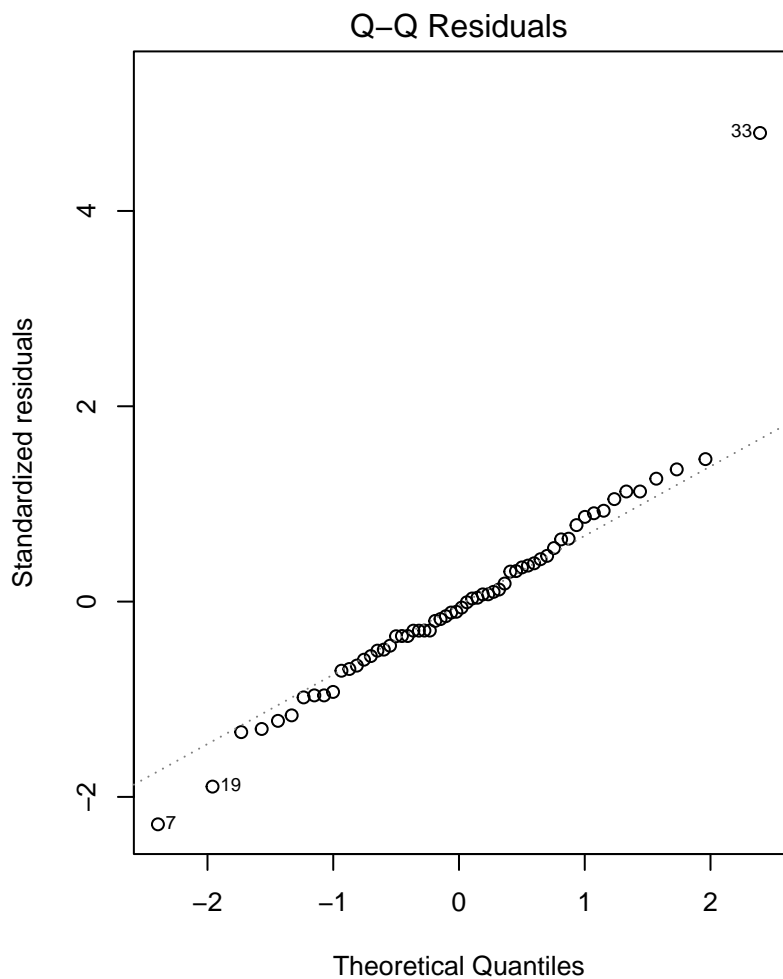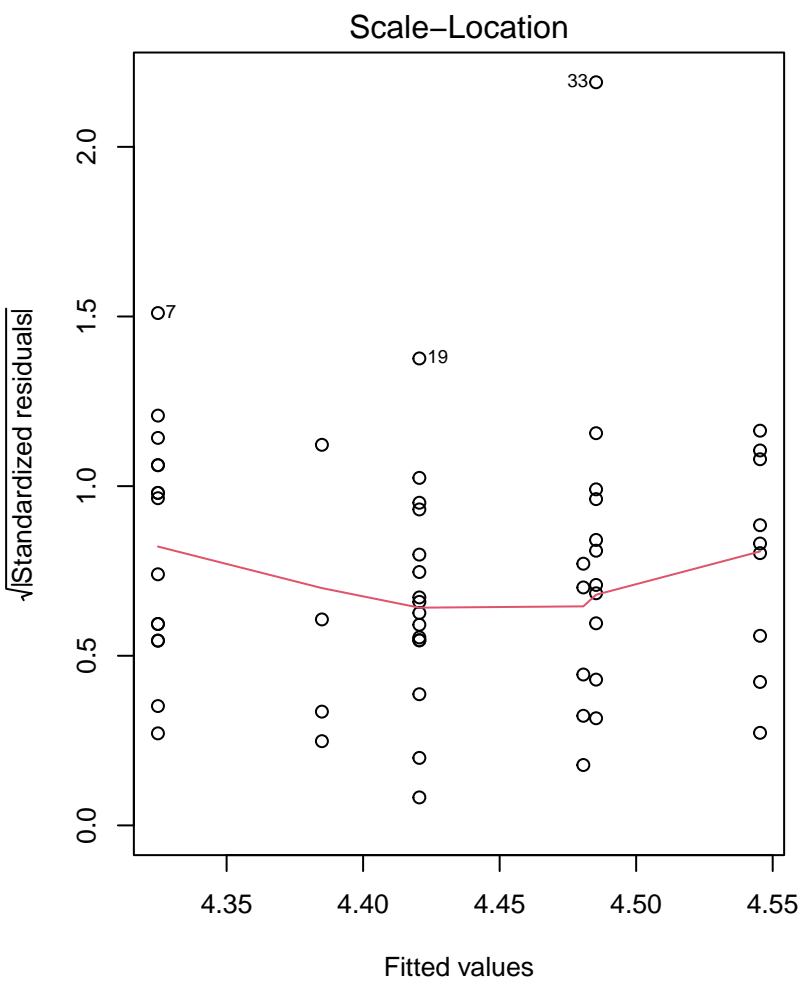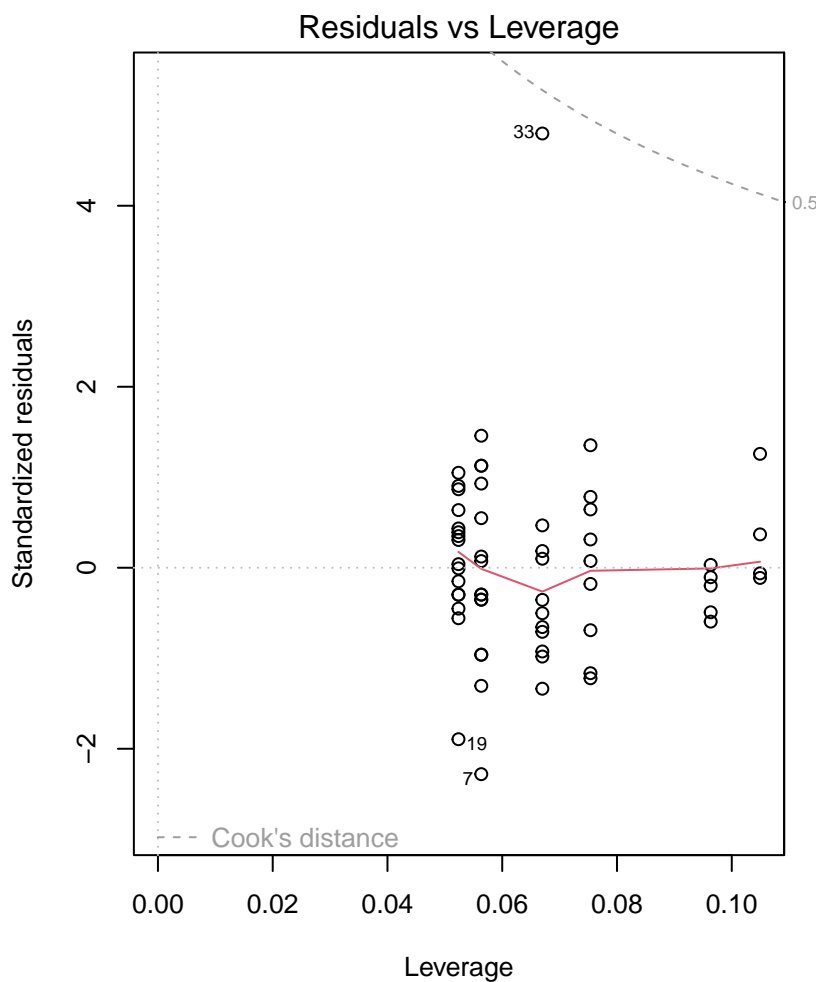

Supplement: Supplementary file 1 [file animals-16-00692-s001.zip › S1_Glucose_m1_Group_Sex.pdf]

lm(malresponse ~ log2[setmlog(arm)], Group ~ Sex, Sex ~ Age, Age ~ Weight)

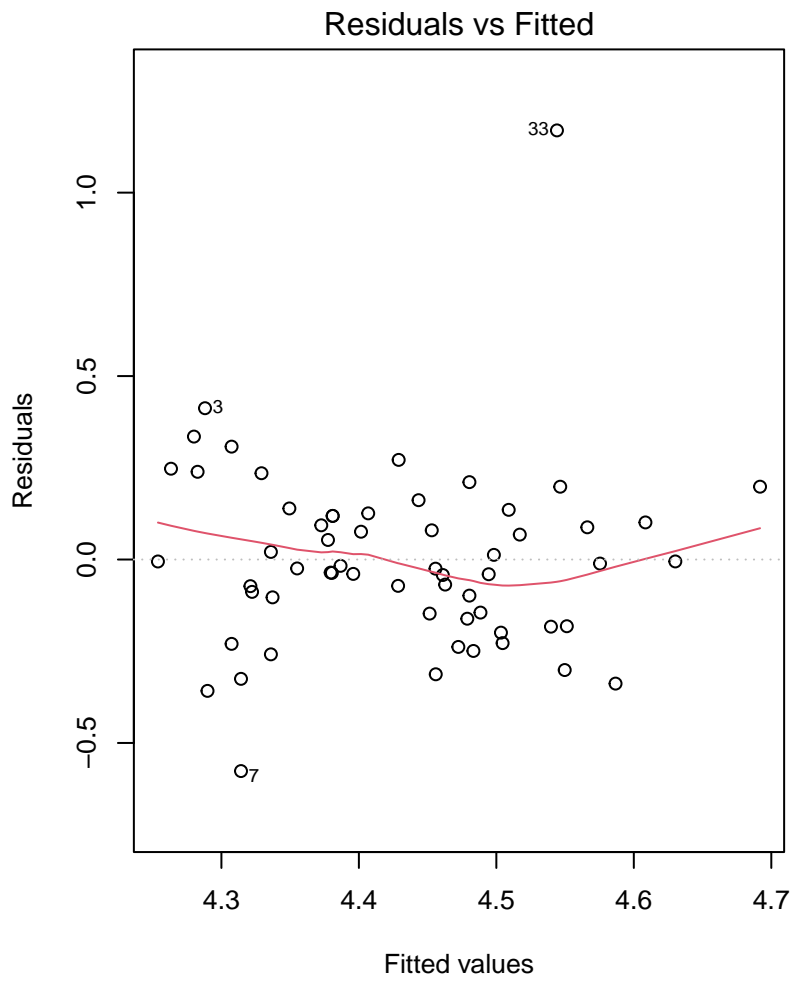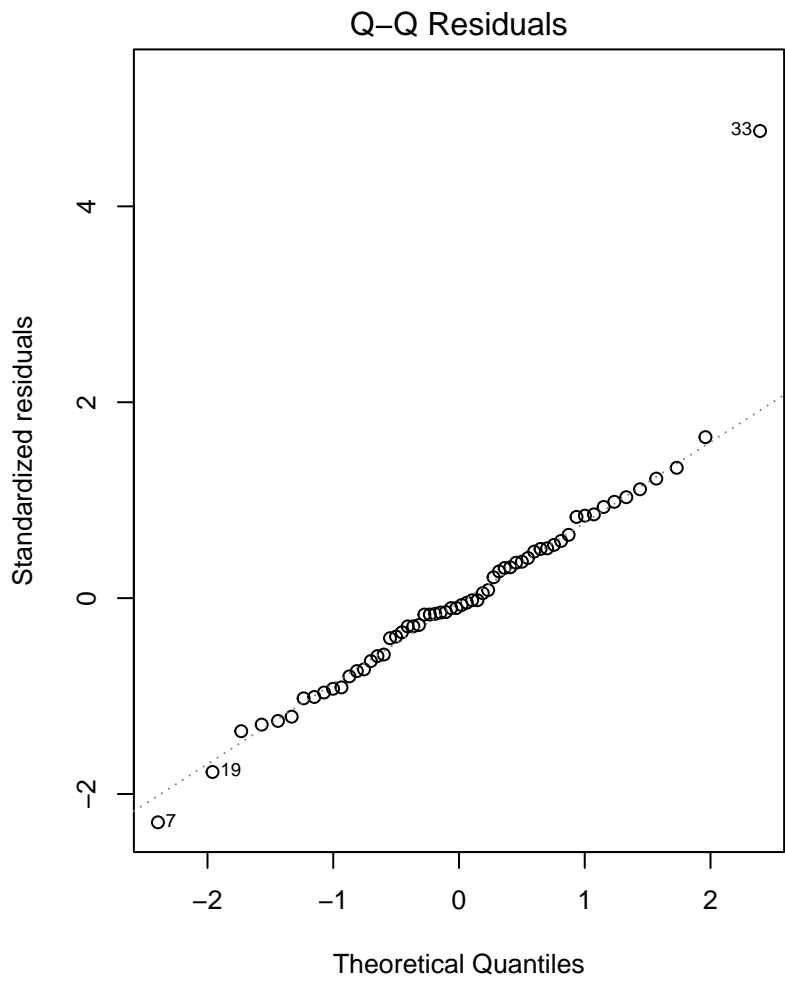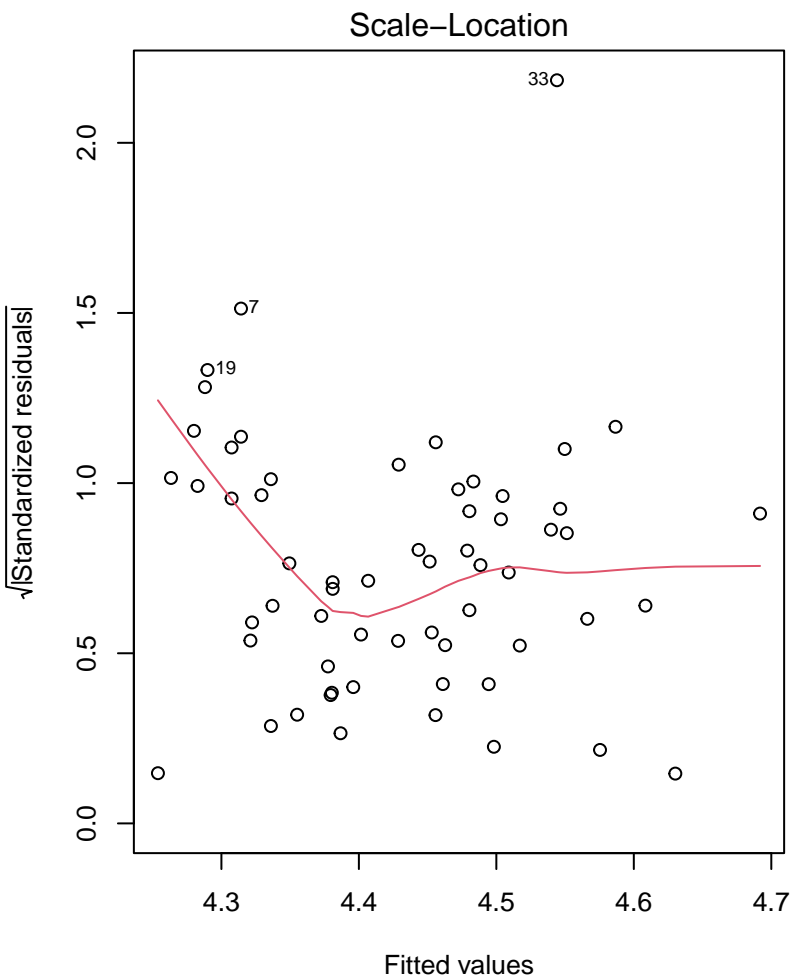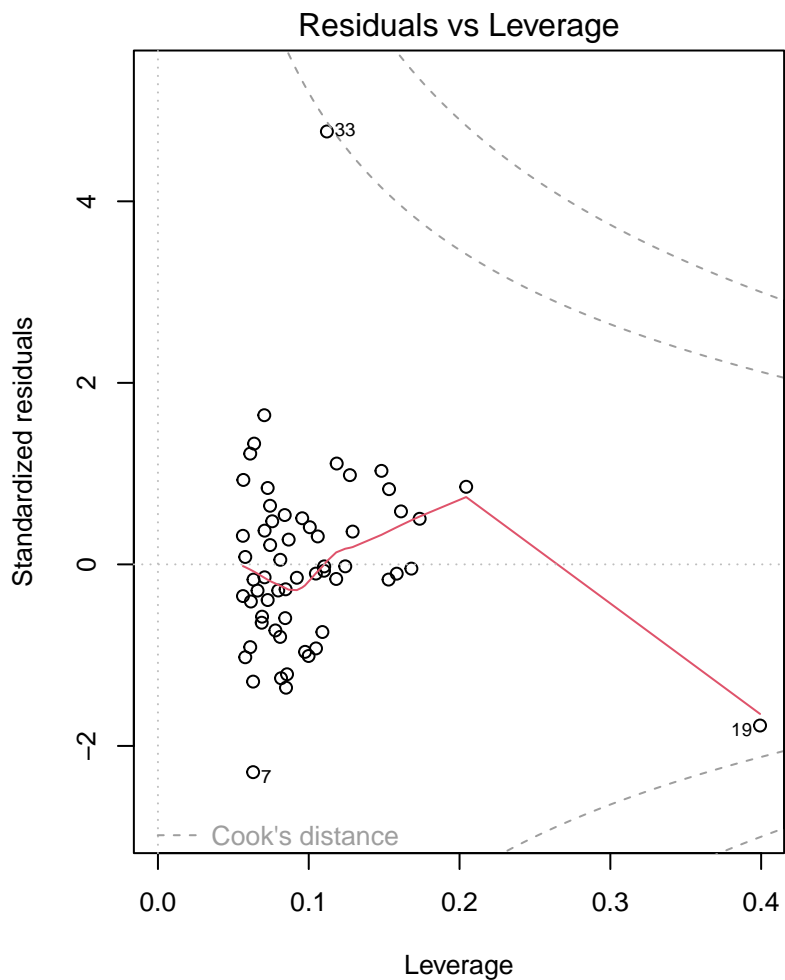

Supplement: Supplementary file 1 [file animals-16-00692-s001.zip › S1_Glucose_m2_Group_Sex_Age_Weight.pdf]

lm(malegresponse(d35[subset.comb(1:10)]) ~ GROUPE + Sex)

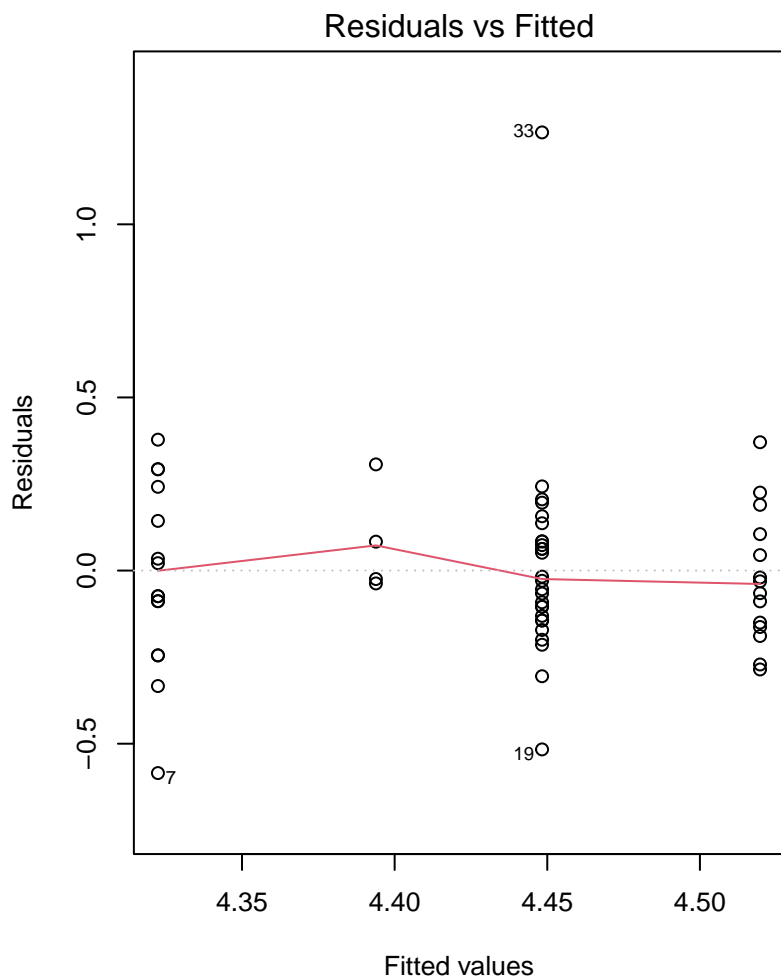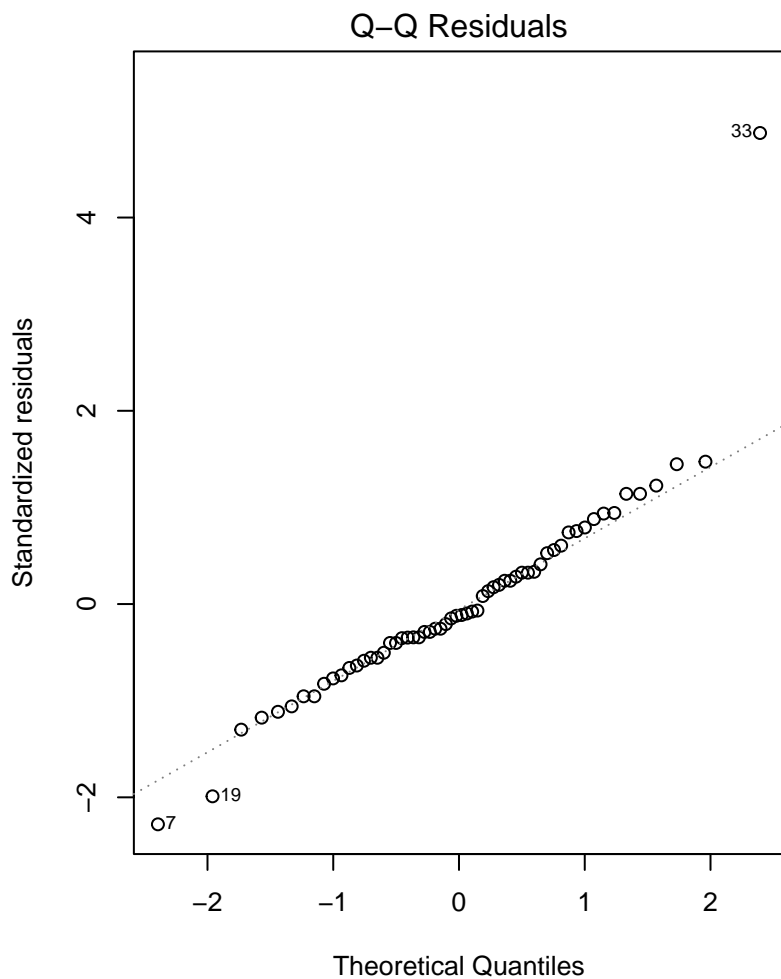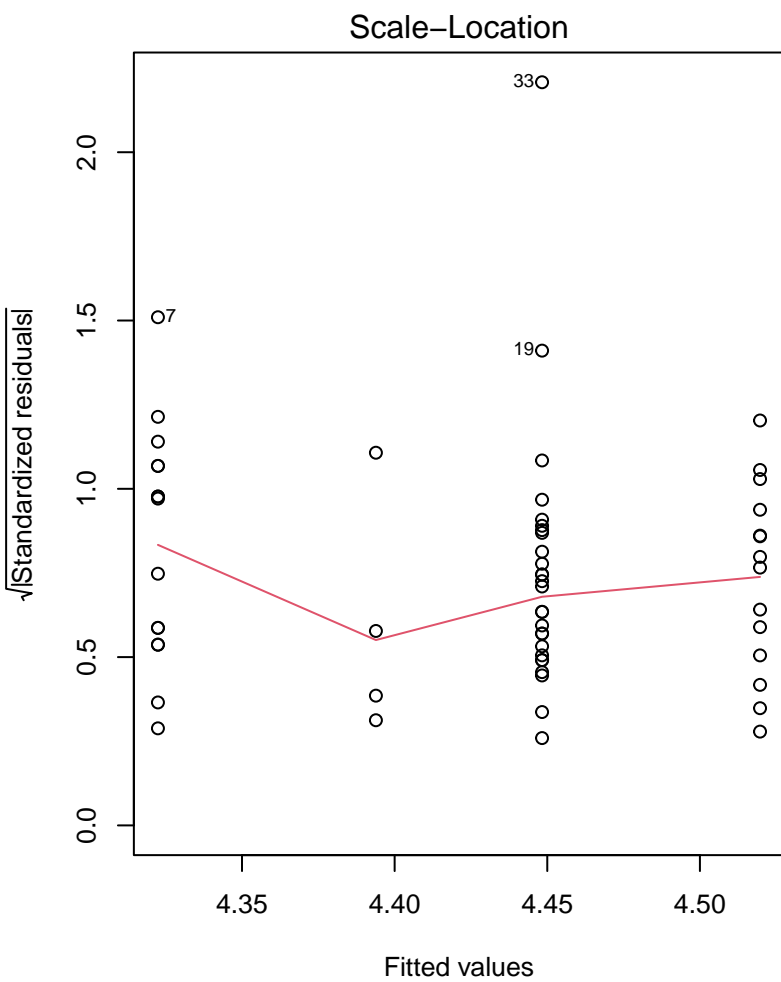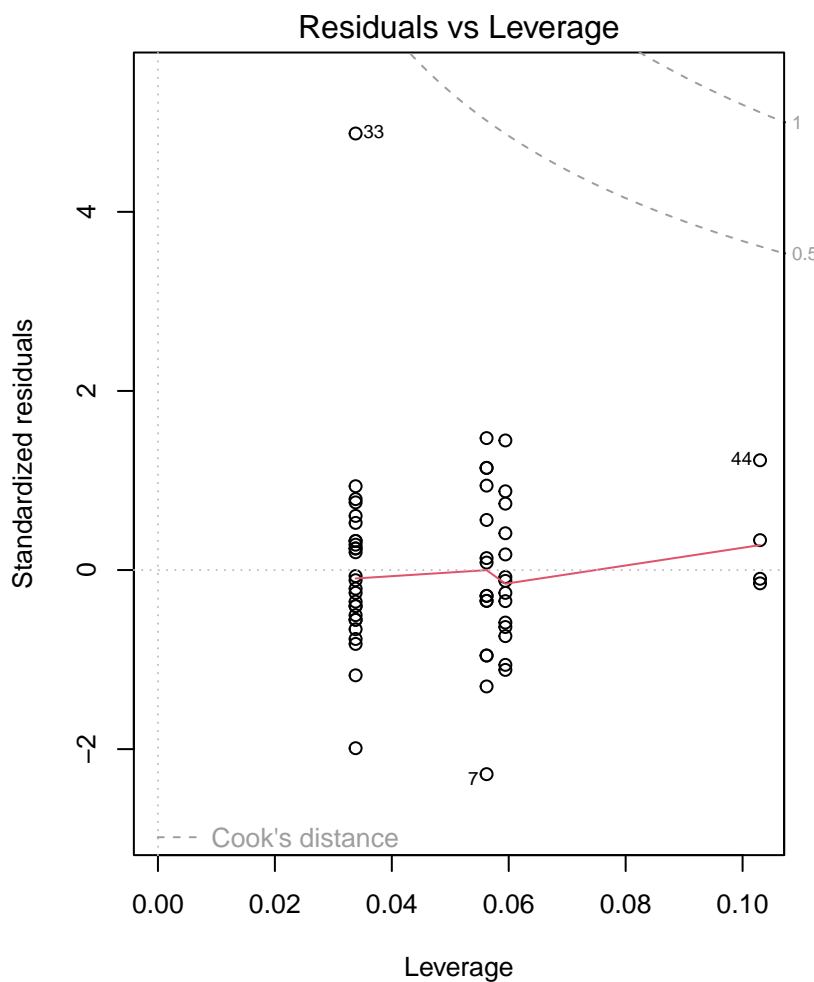

Supplement: Supplementary file 1 [file animals-16-00692-s001.zip › S1_Glucose_m3_GROUPII_Sex.pdf]

lm(make\_response ~ d1[[putting\_name]] + Group + Sex)

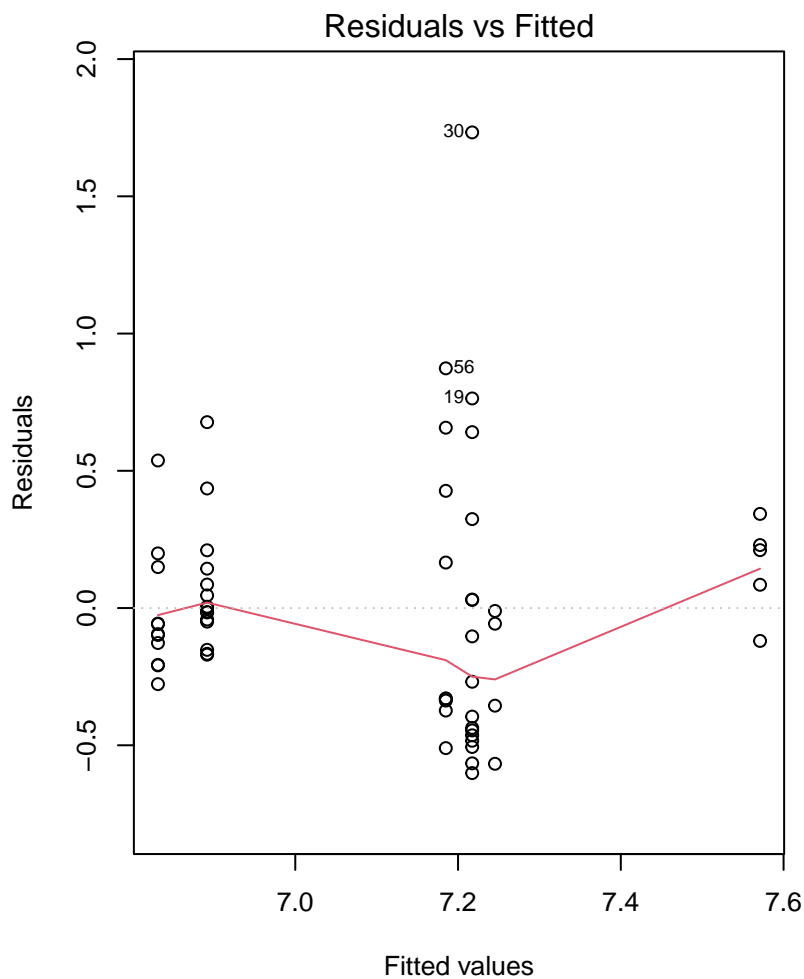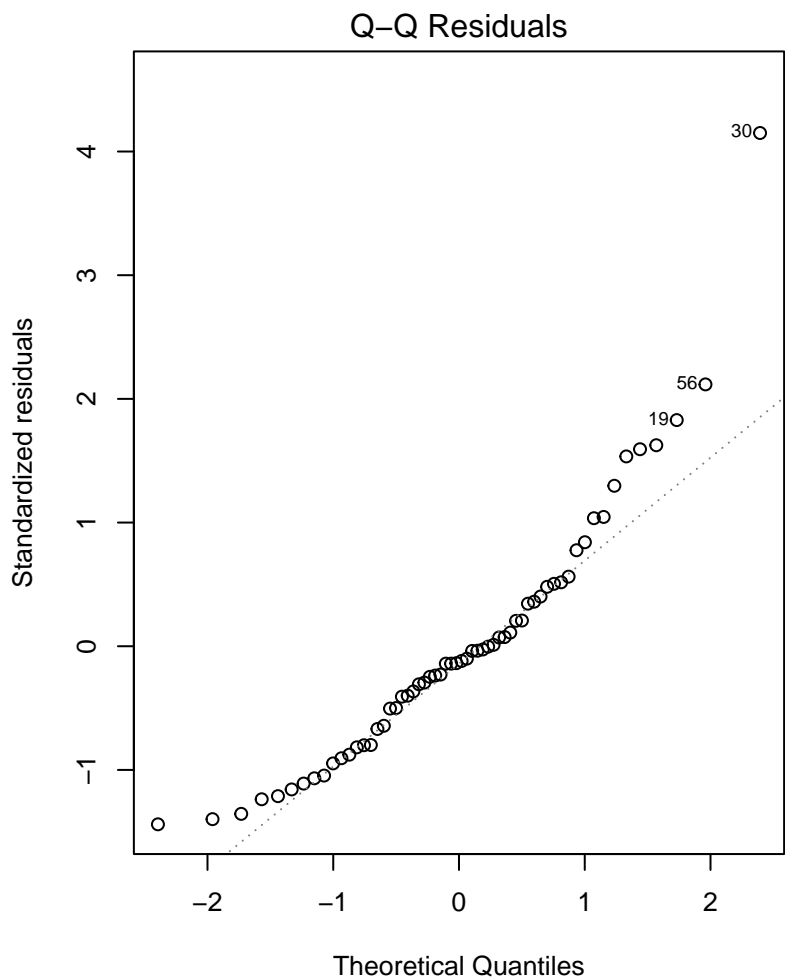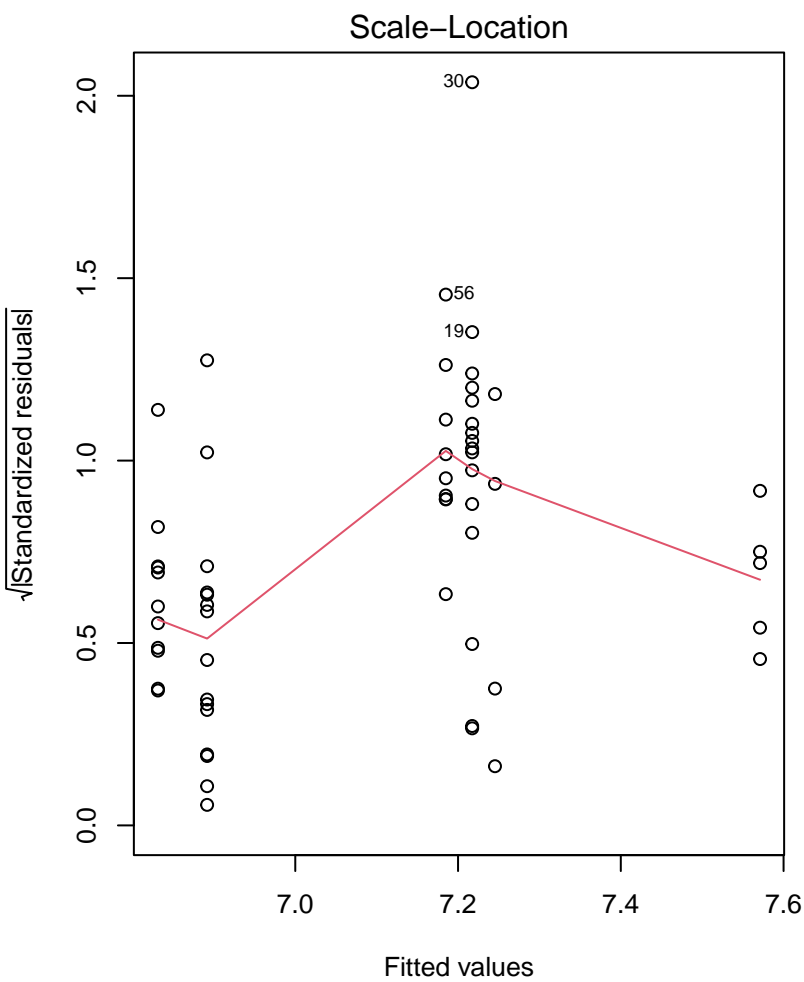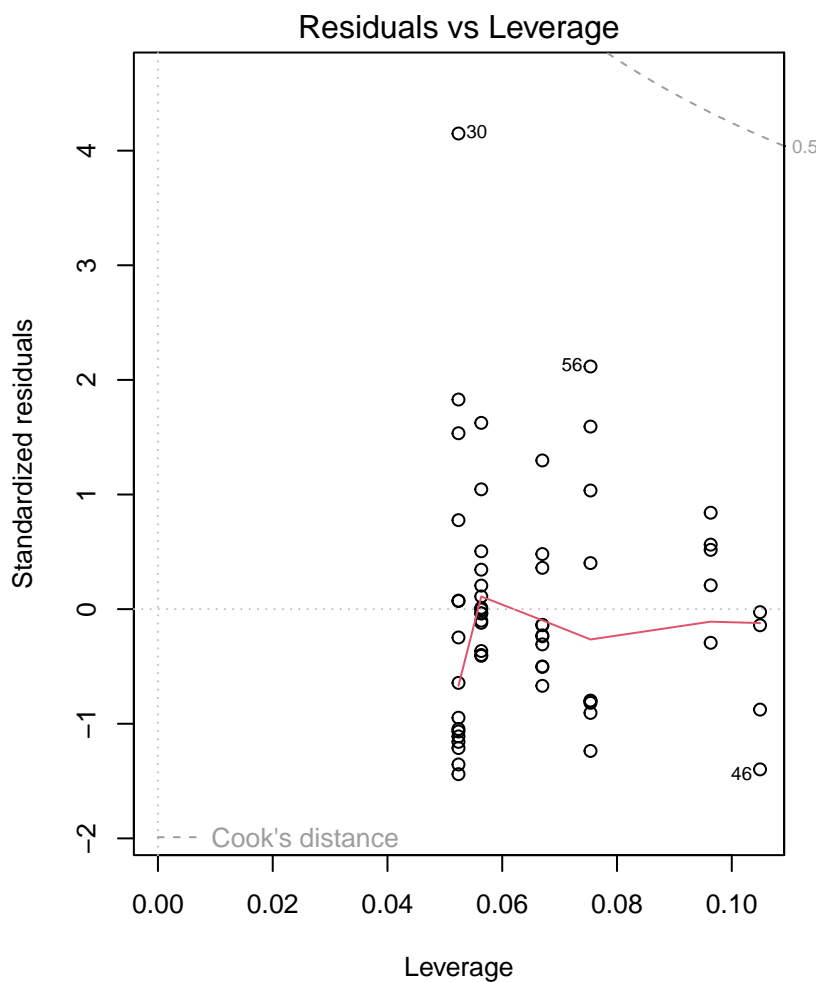

Supplement: Supplementary file 1 [file animals-16-00692-s001.zip › S1_IL-1_m1_Group_Sex.pdf]

lm(make\_response ~ 12[1:12] + outcome(Year), Group = Sex + Age + Weight)

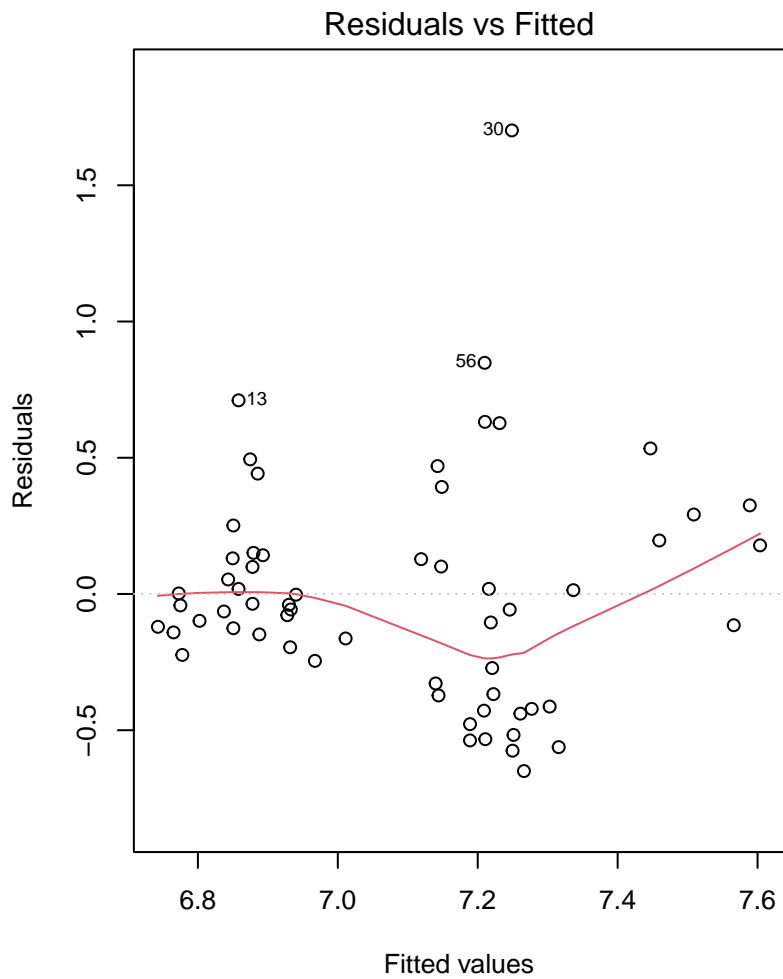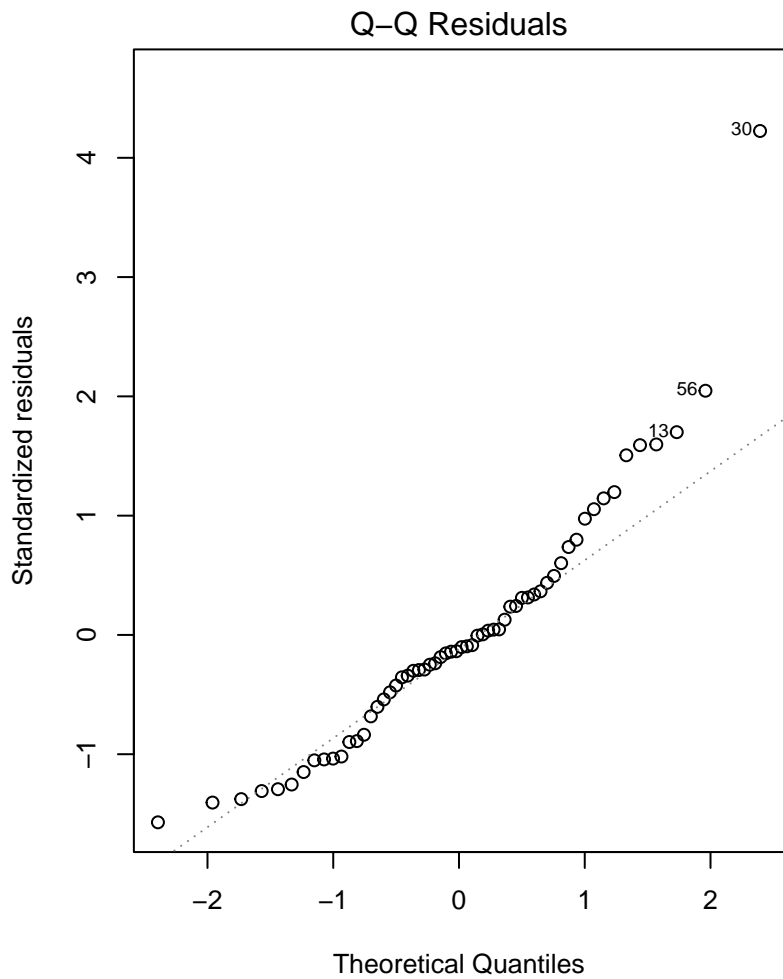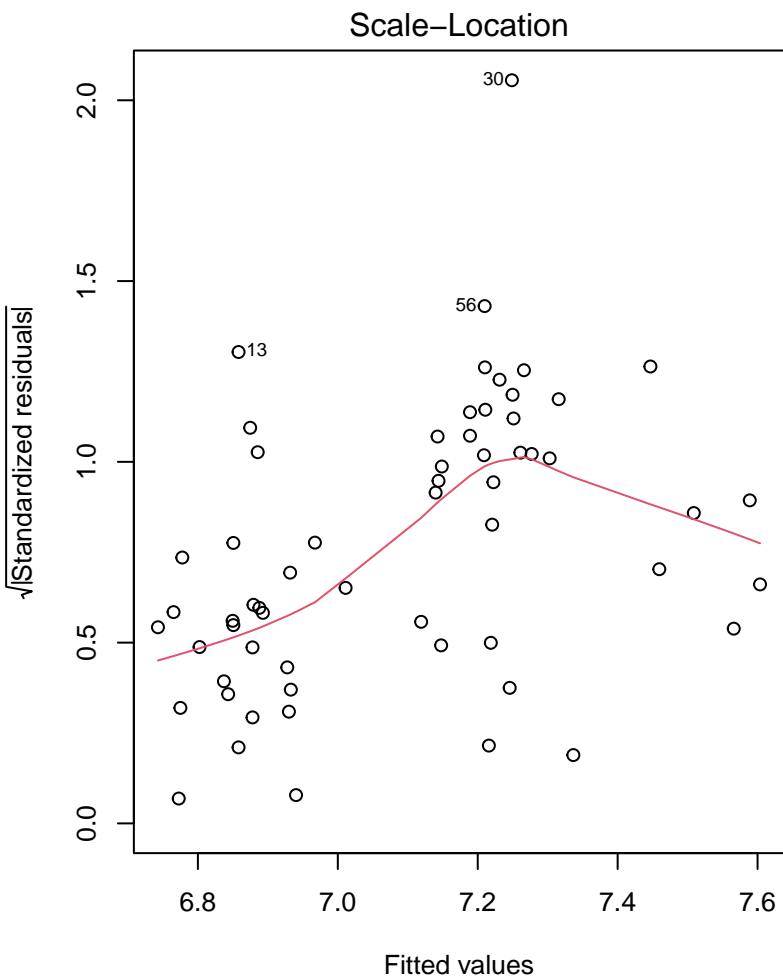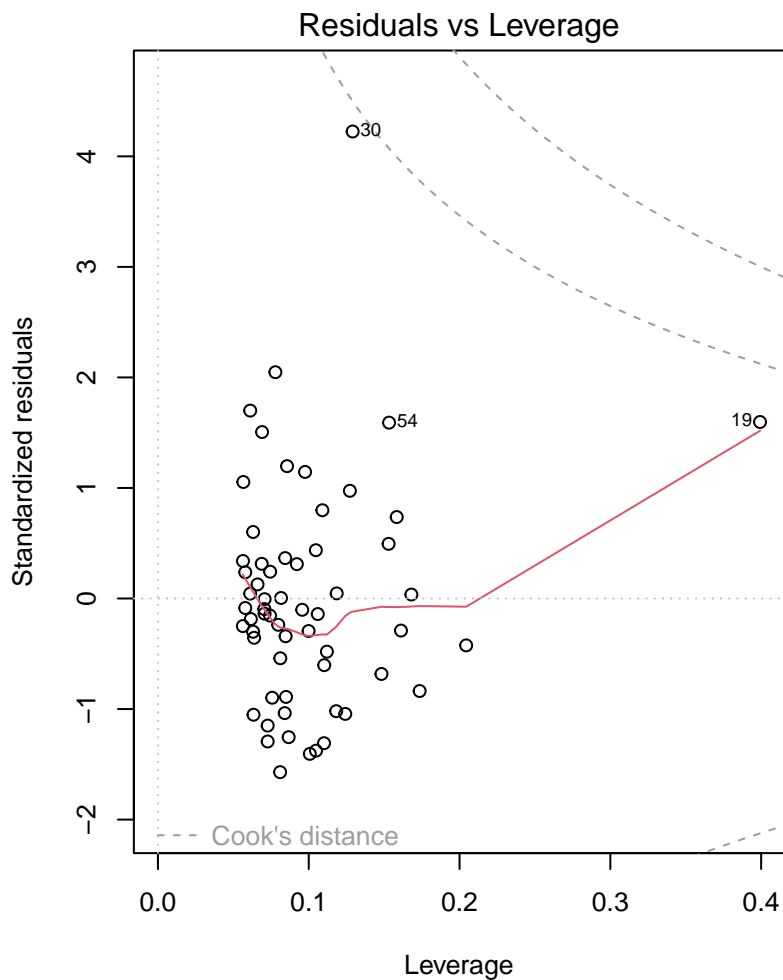

Supplement: Supplementary file 1 [file animals-16-00692-s001.zip › S1_IL-1_m2_Group_Sex_Age_Weight.pdf]

# Im(response(l3[OutcomeName]), GROUP\_Bel + Sex)

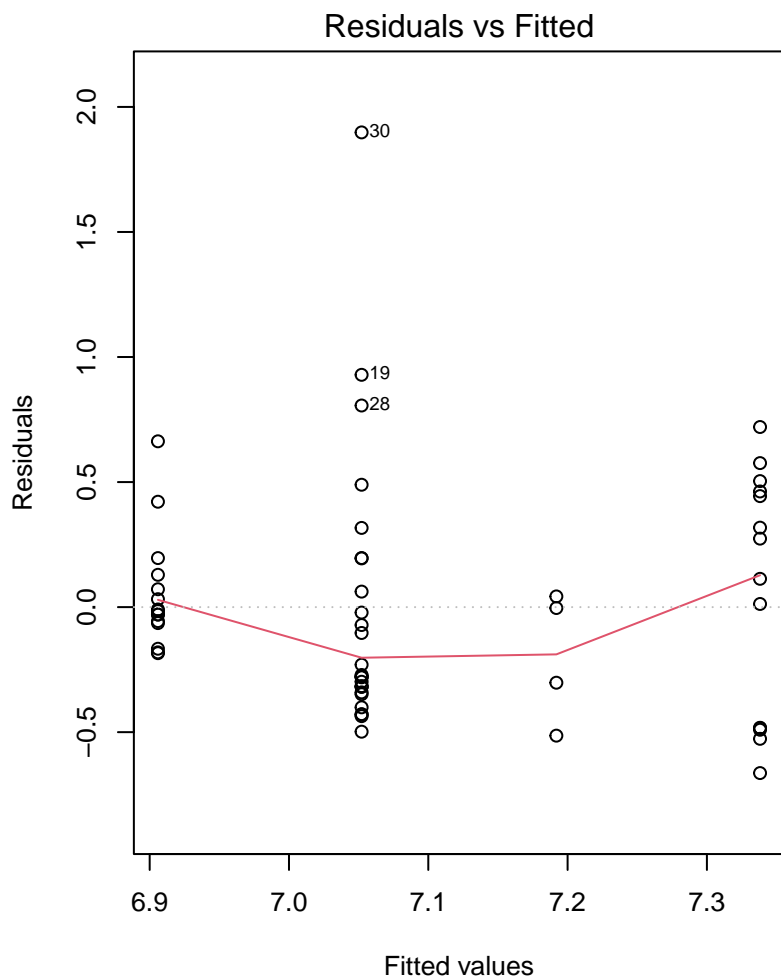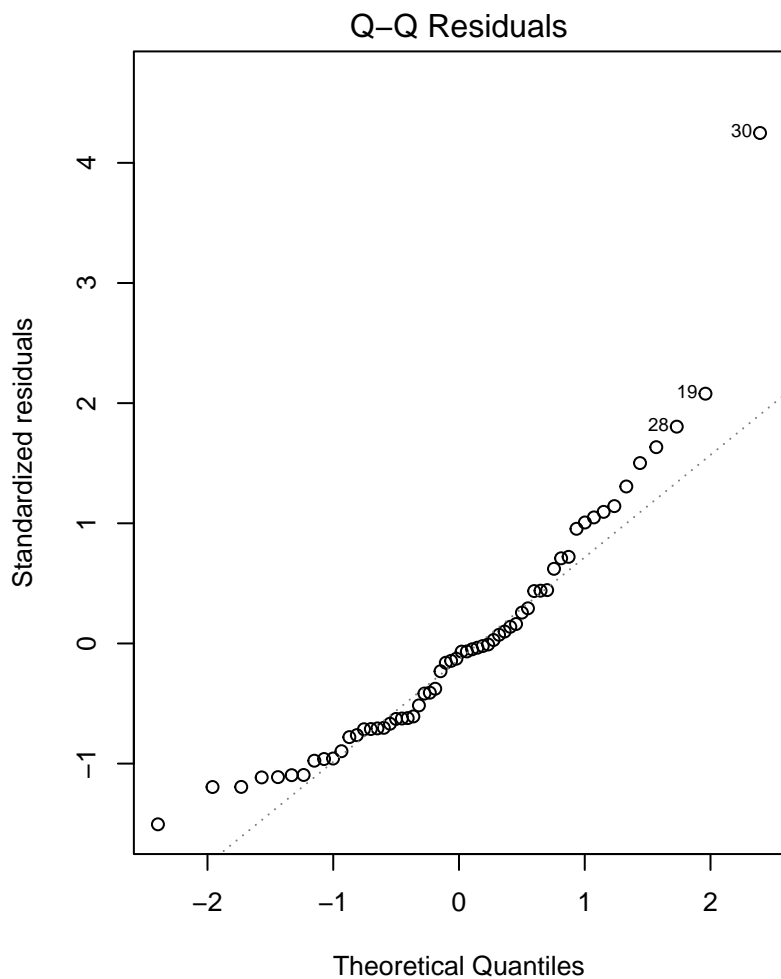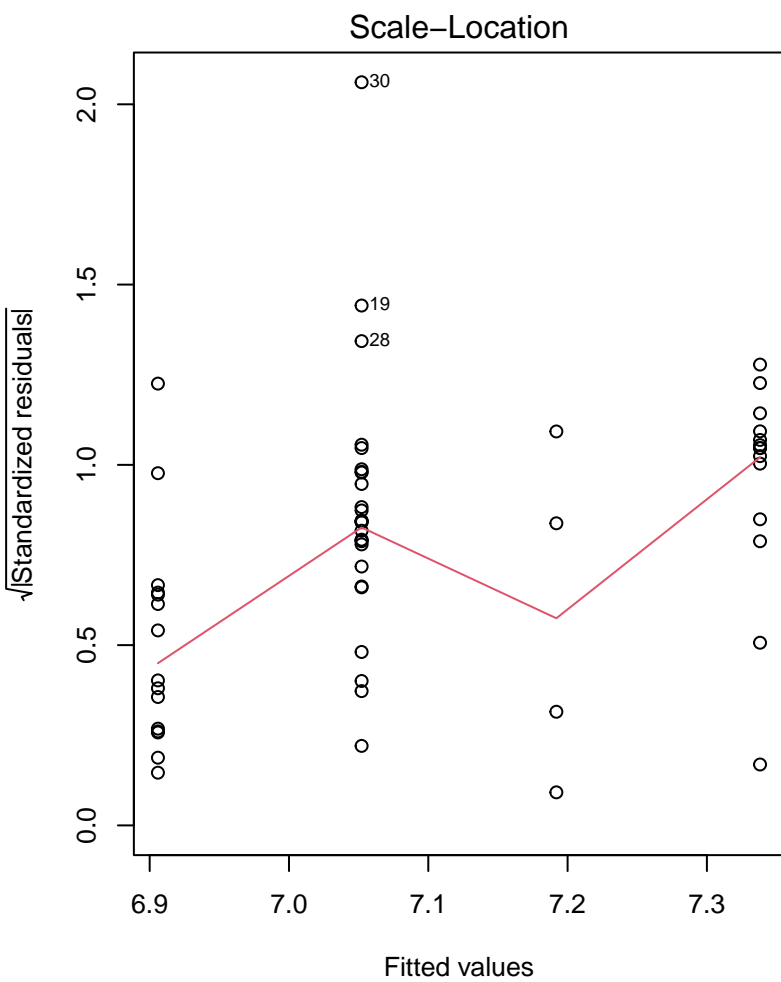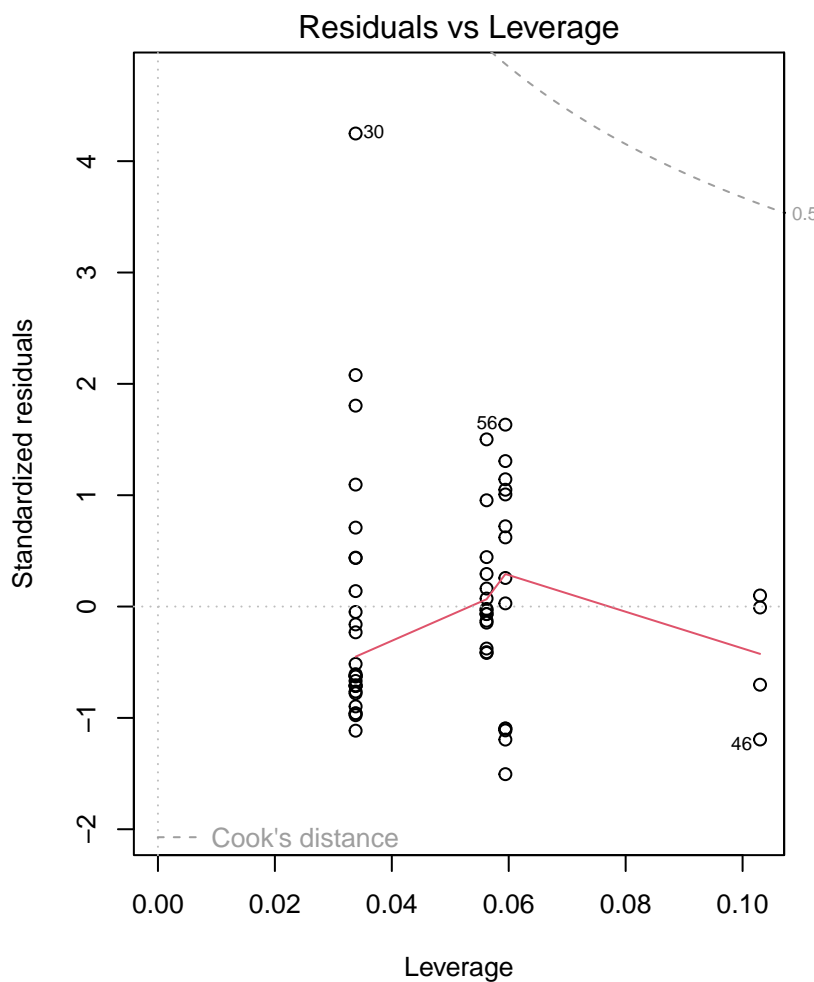

Supplement: Supplementary file 1 [file animals-16-00692-s001.zip › S1_IL-1_m3_GROUPII_Sex.pdf]

lm(make\_response ~ d14[putting name] + Group + Sex)

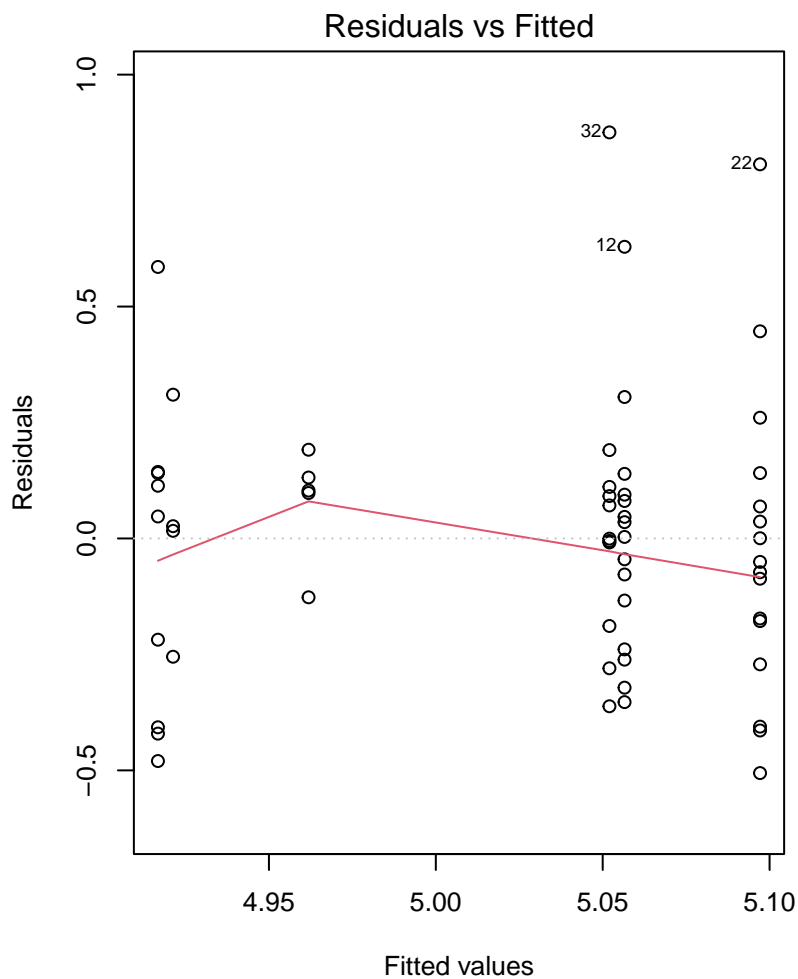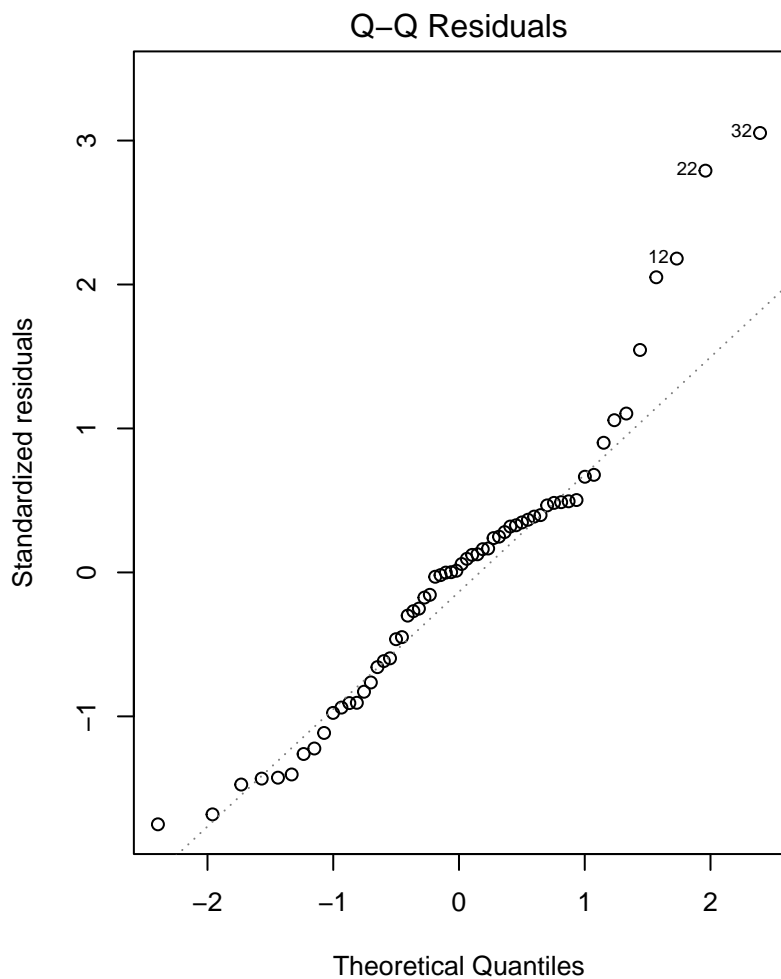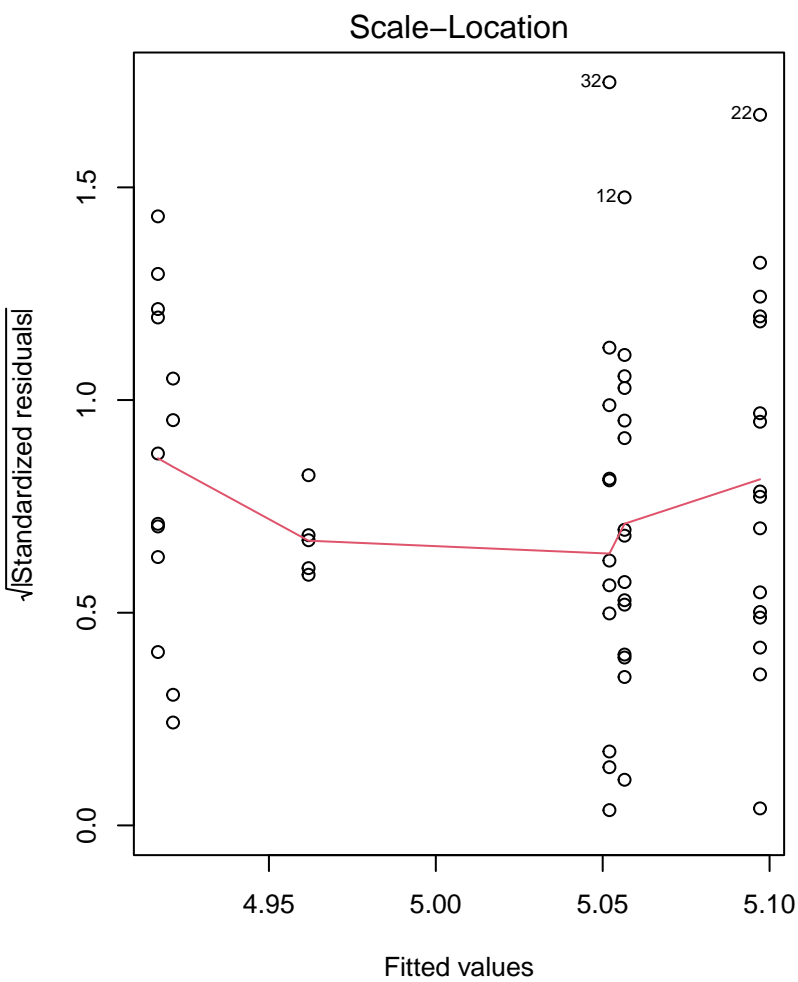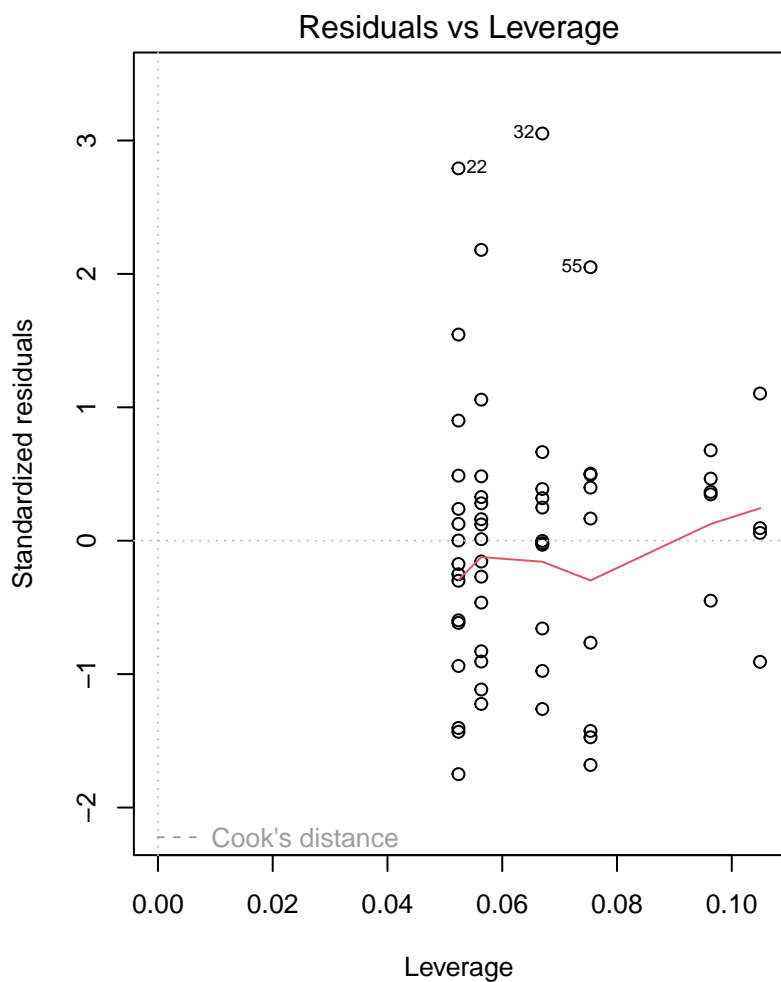

Supplement: Supplementary file 1 [file animals-16-00692-s001.zip › S1_IL-4_m1_Group_Sex.pdf]

lm(make\_response ~ 12[4] + outcome (name) Group + Sex + Age + Weight)

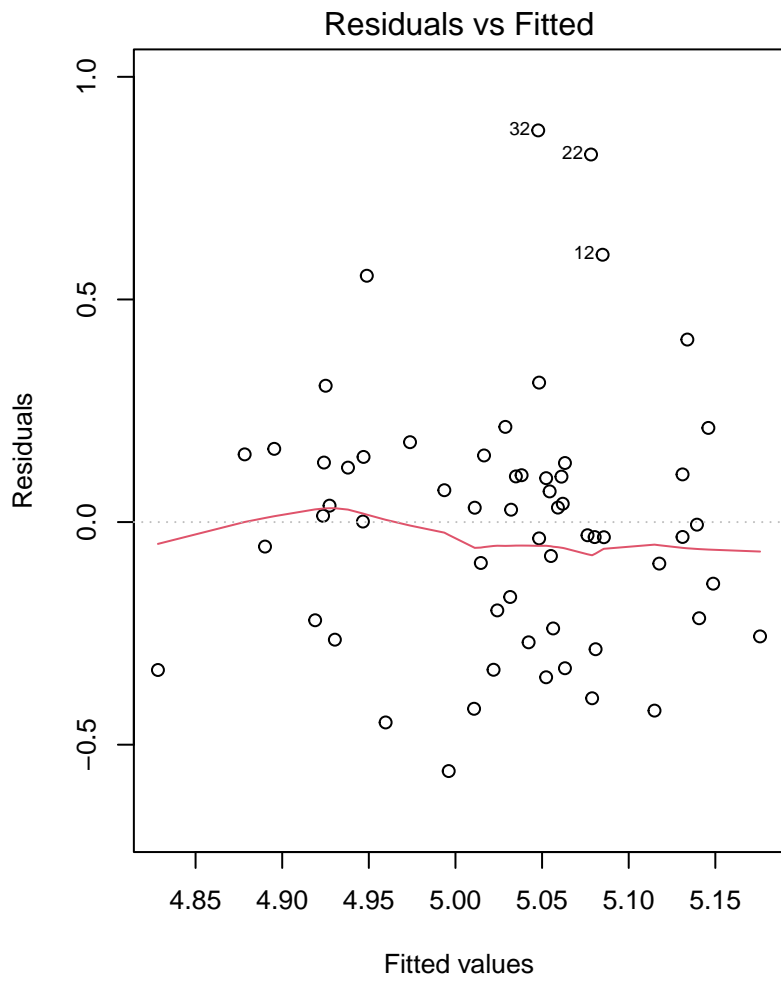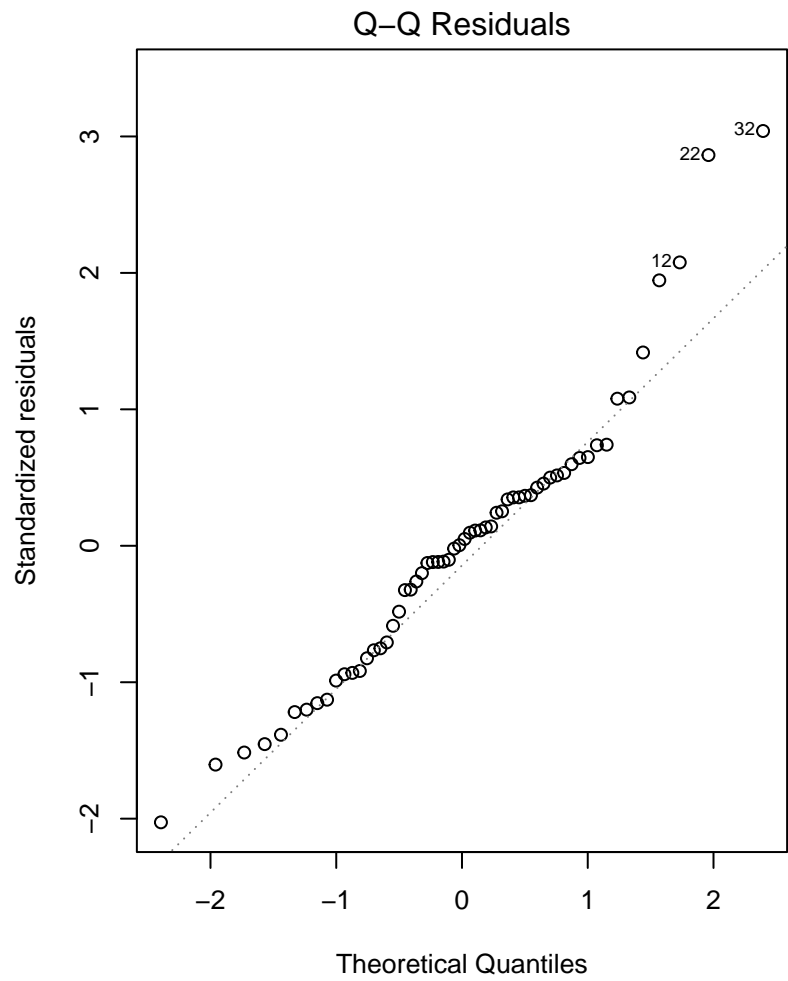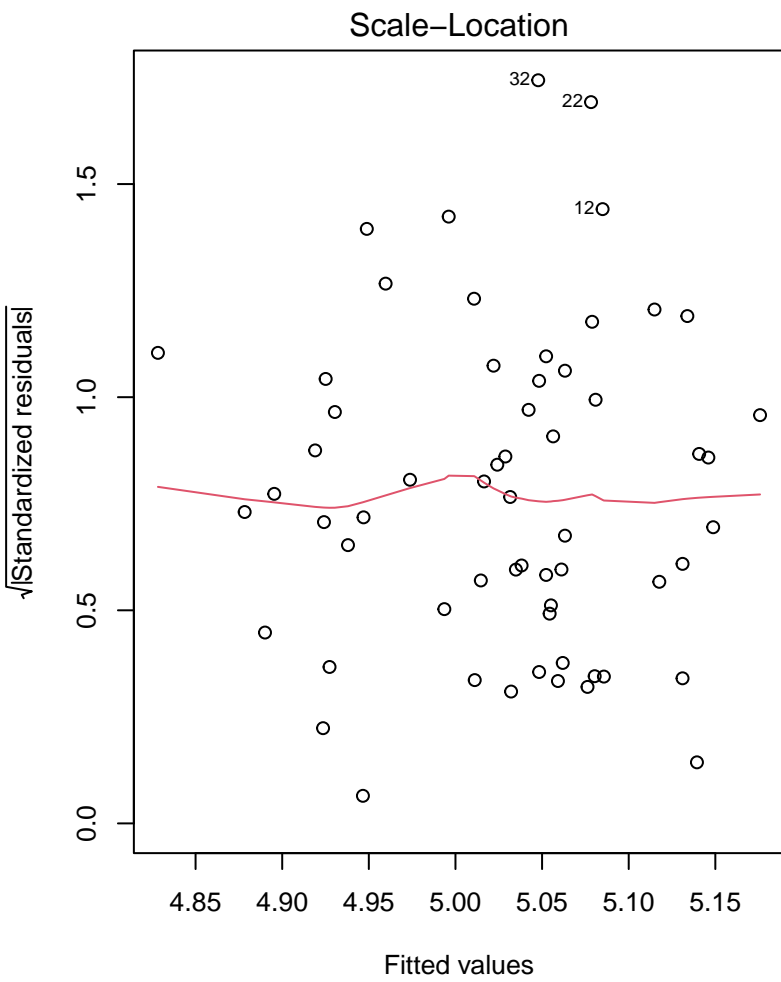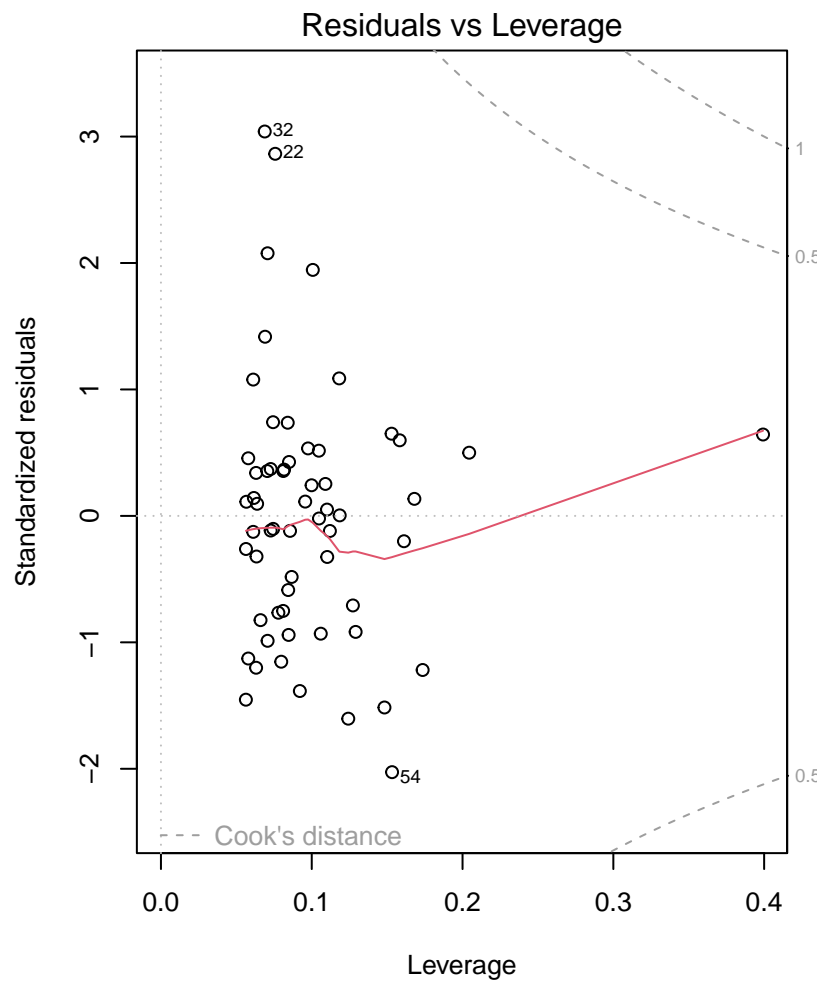

Supplement: Supplementary file 1 [file animals-16-00692-s001.zip › S1_IL-4_m2_Group_Sex_Age_Weight.pdf]

lm(make\_response ~ (1344) + income (name) + GROUP + Bel + Sex)

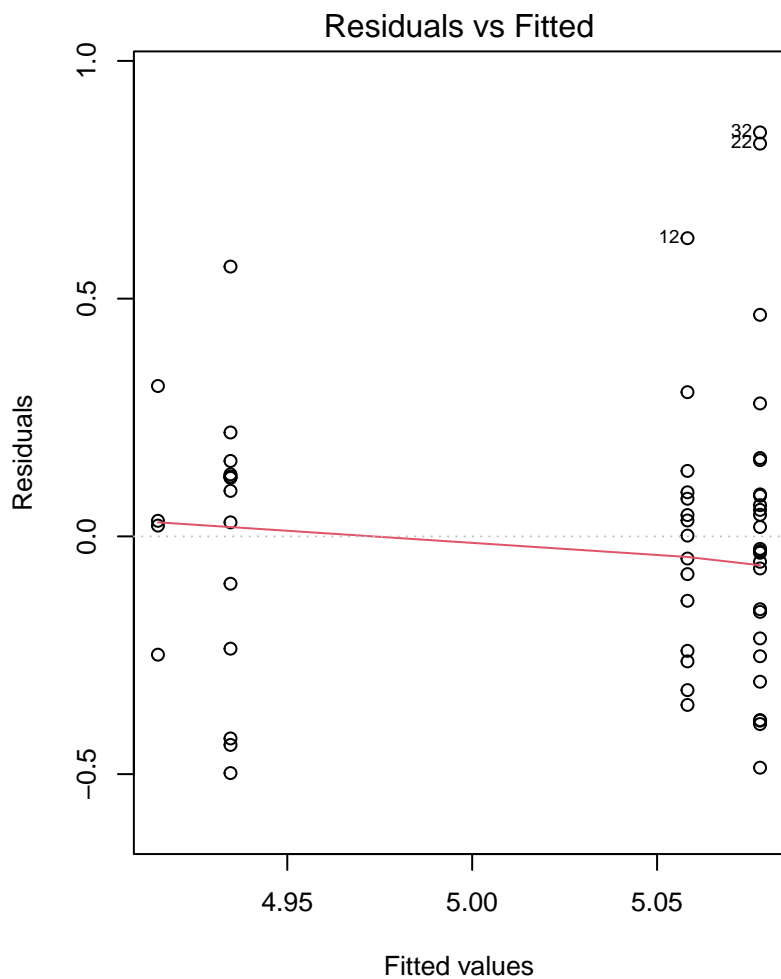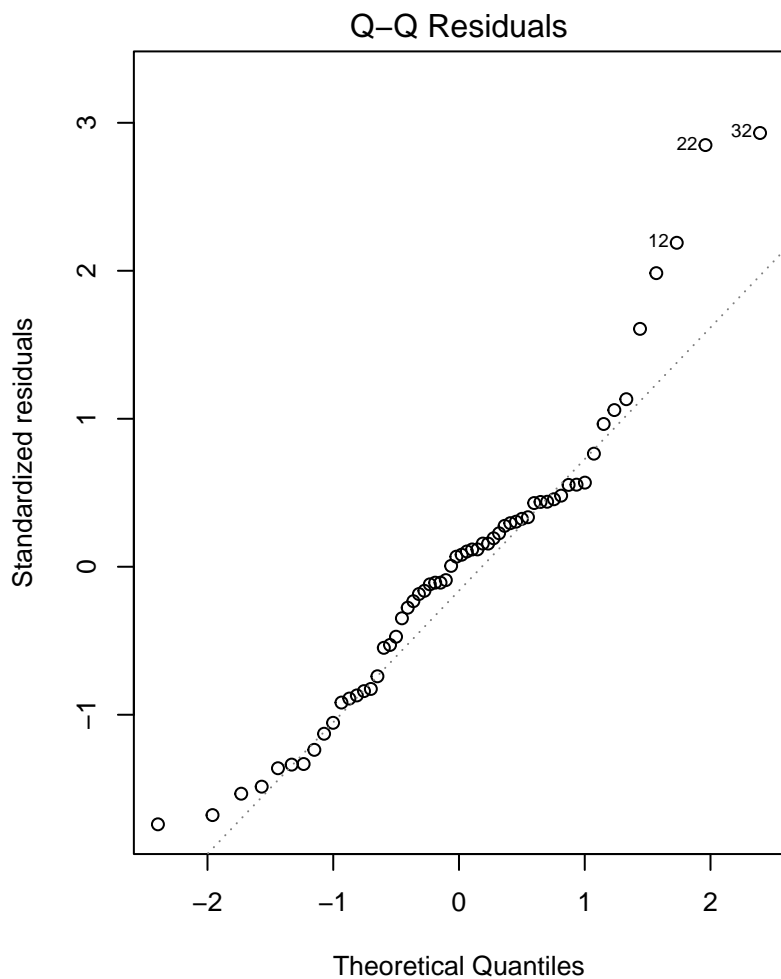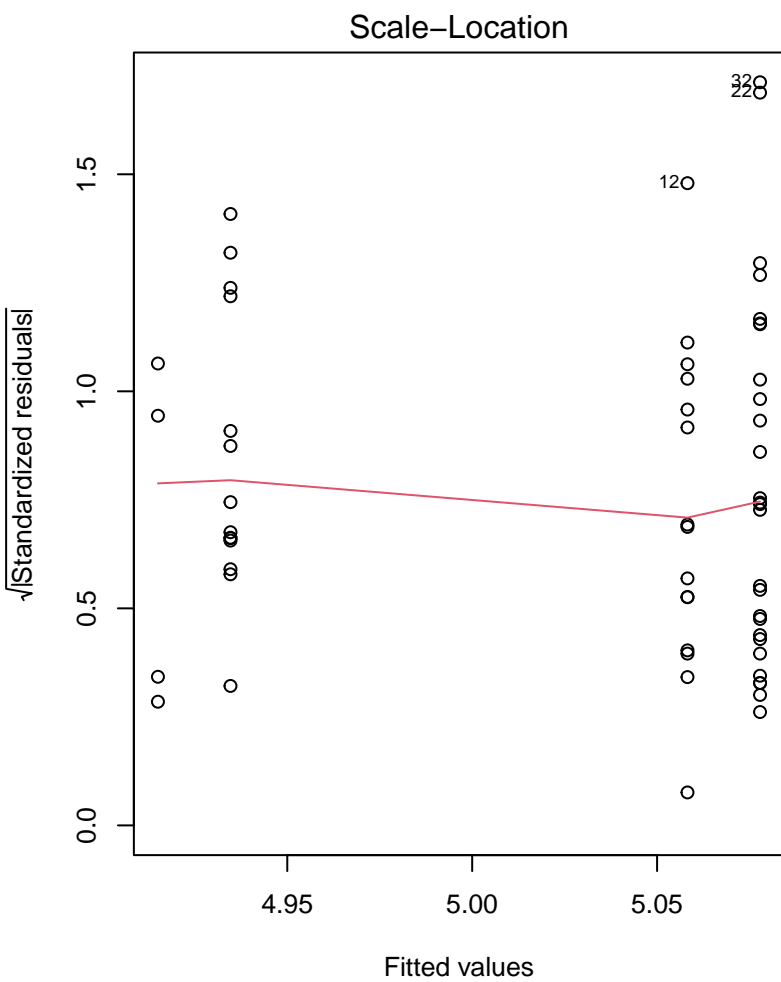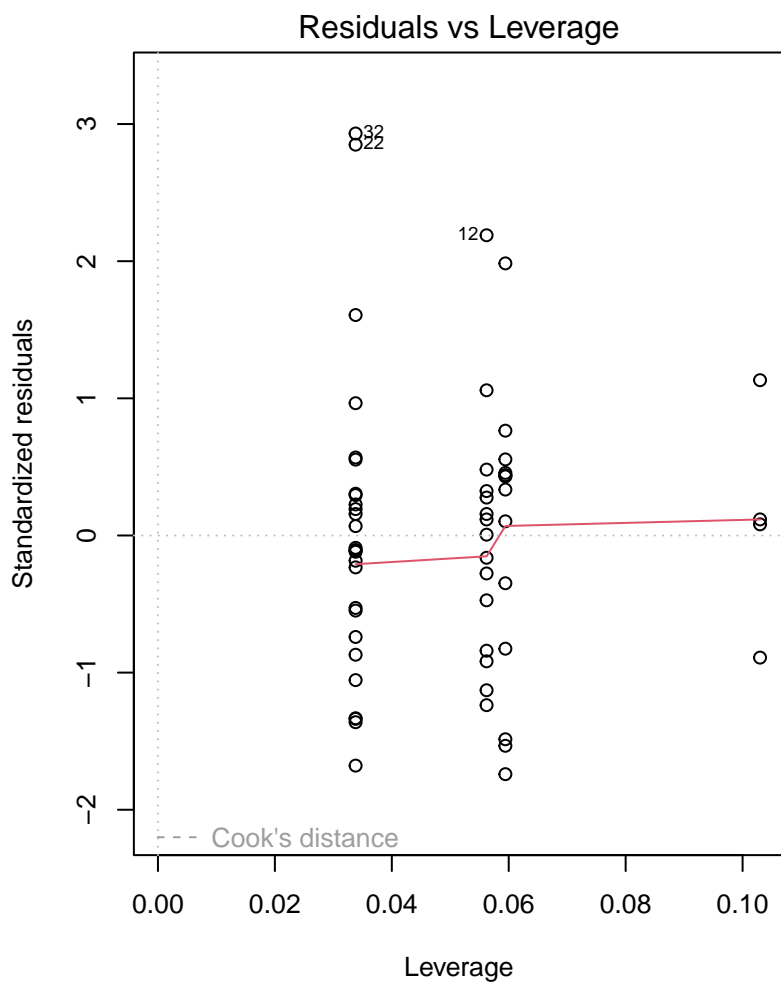

Supplement: Supplementary file 1 [file animals-16-00692-s001.zip › S1_IL-4_m3_GROUPII_Sex.pdf]

lm(male\_response ~ 1 | Outcome, data = Group + Sex)

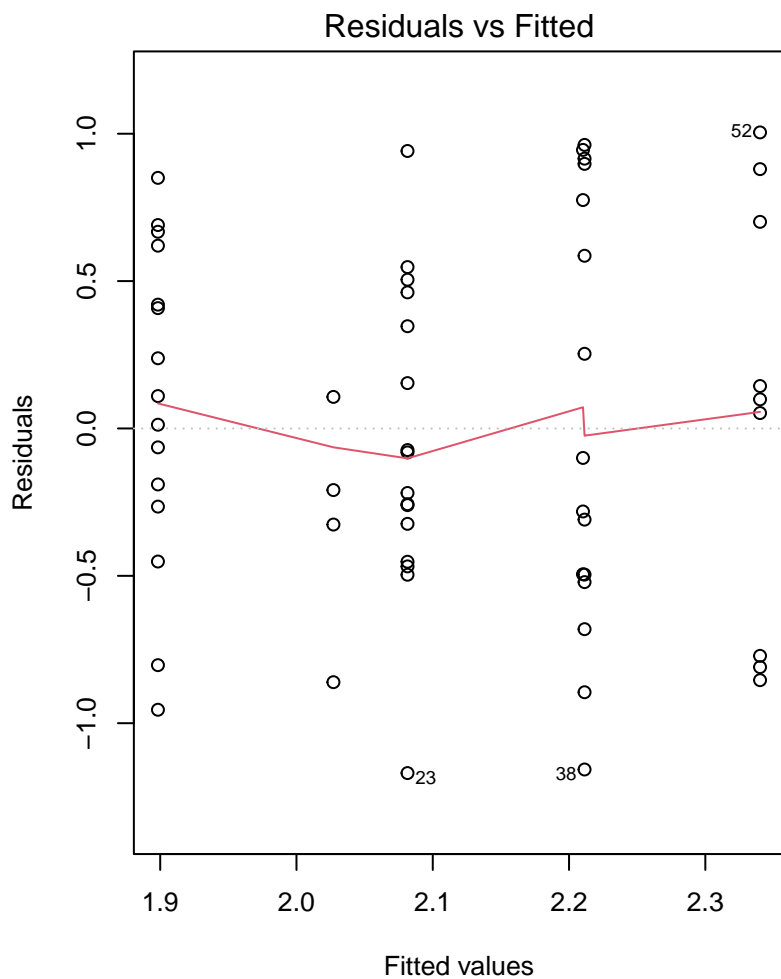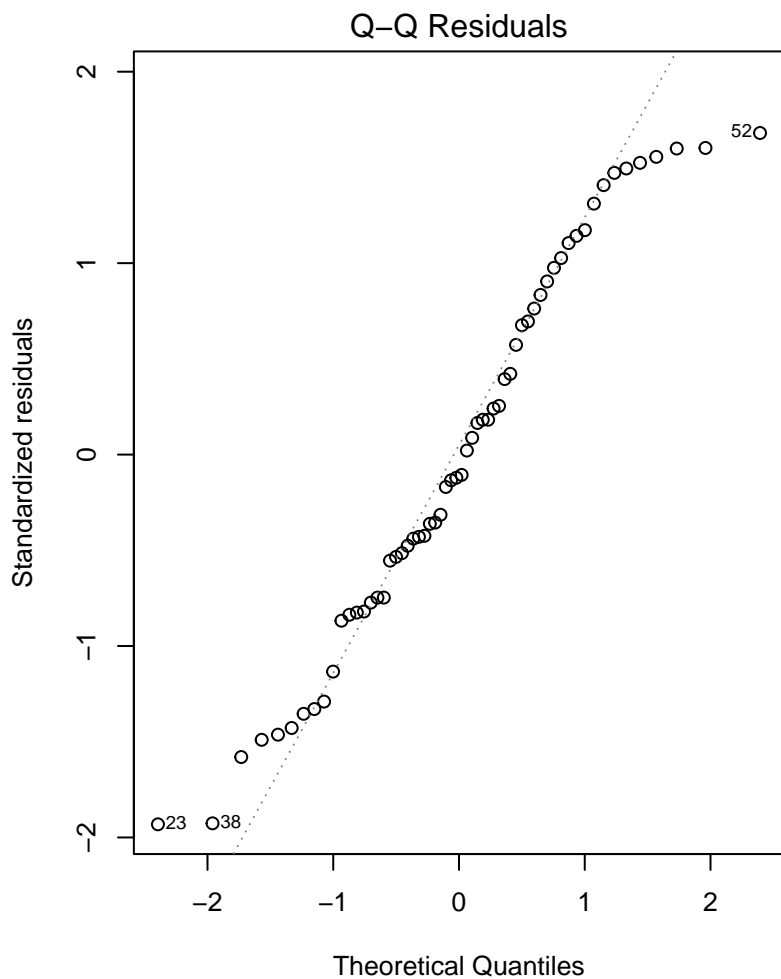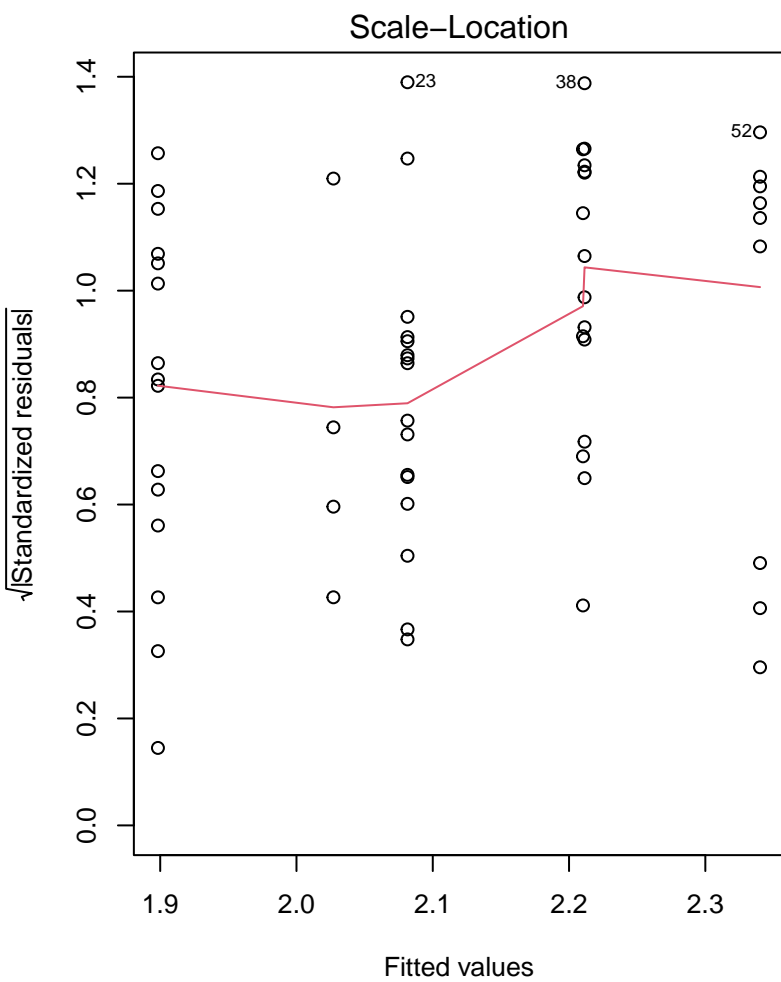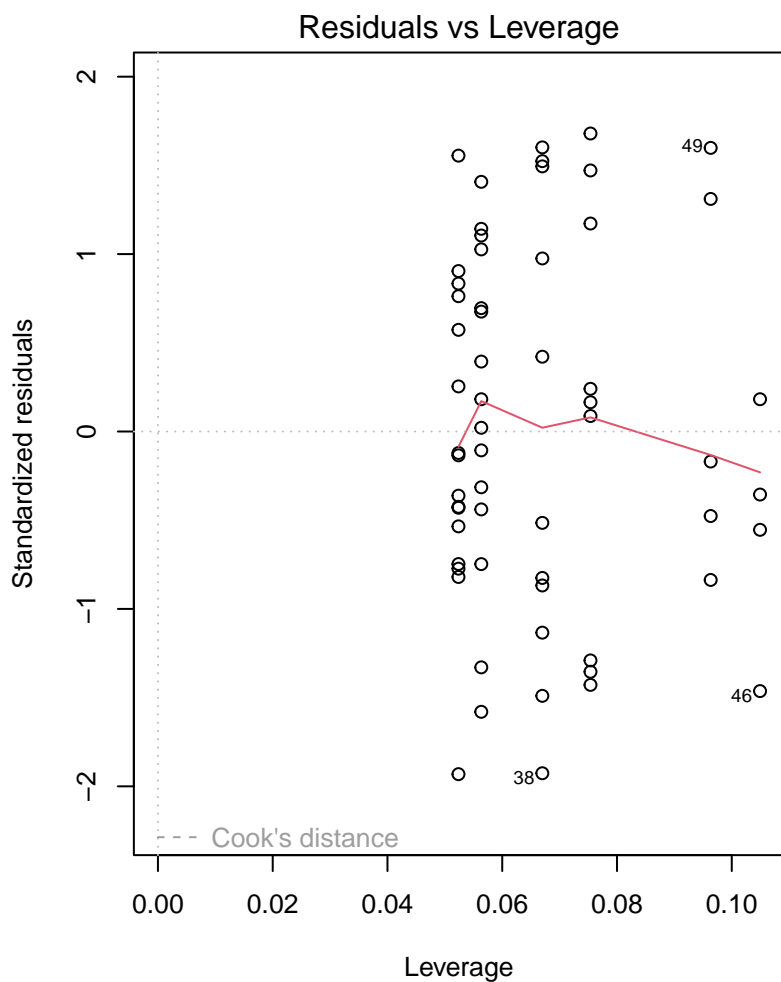

Supplement: Supplementary file 1 [file animals-16-00692-s001.zip › S1_IL-10_m1_Group_Sex.pdf]

lm(make\_response(d2[[Outcome]], Group + Sex + Age + Weight))

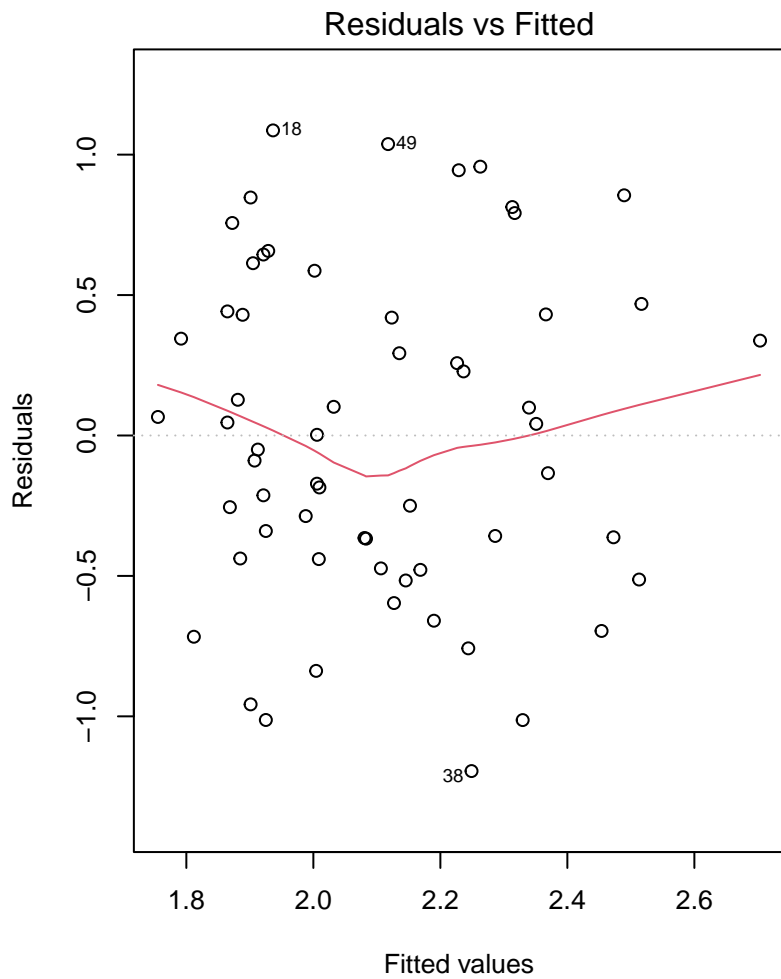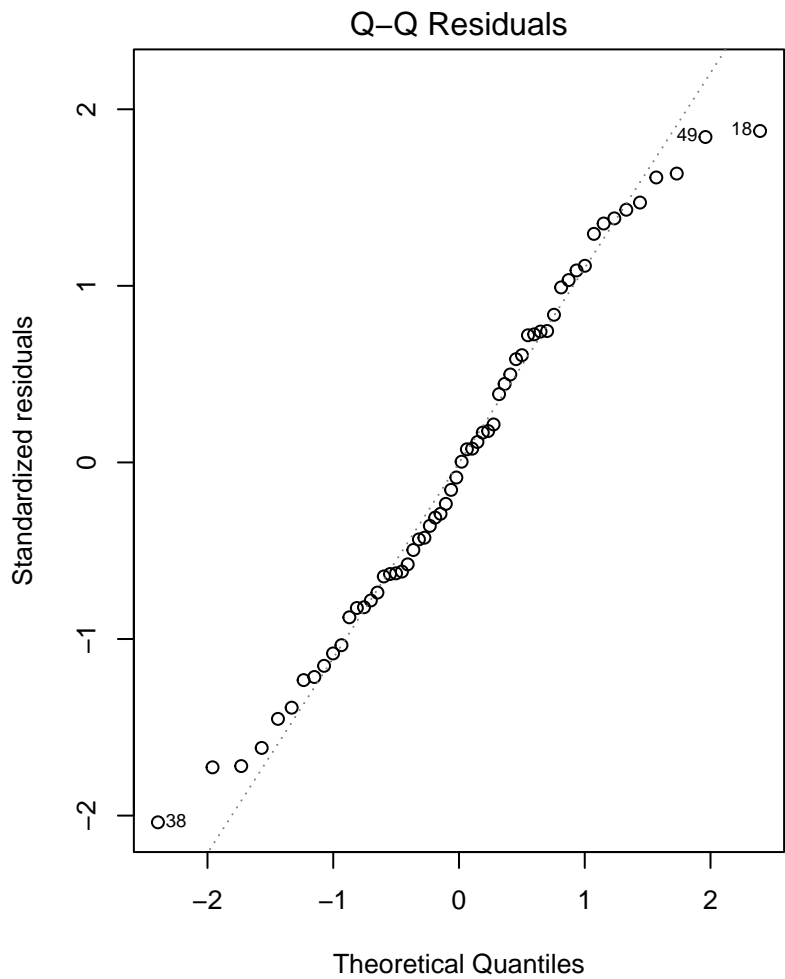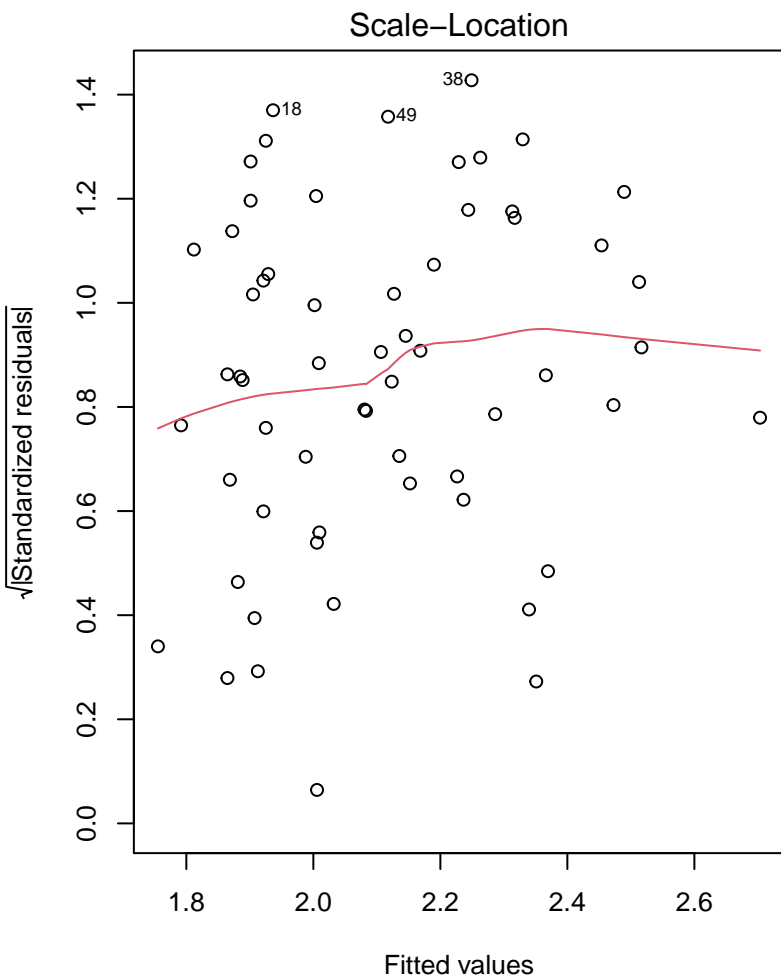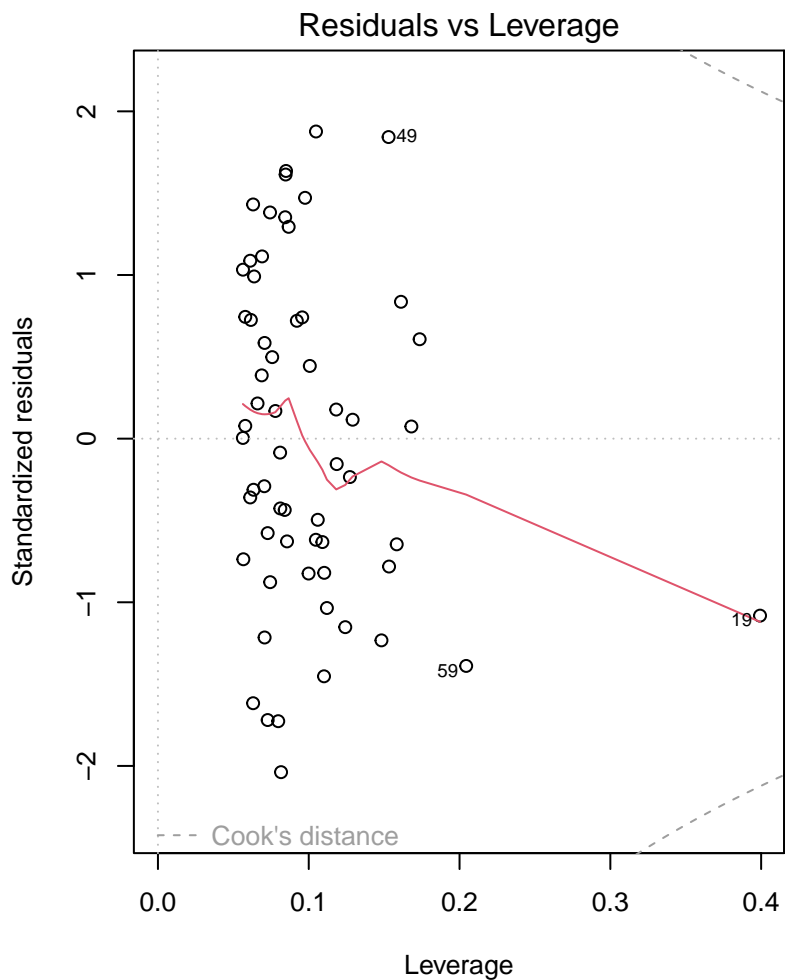

Supplement: Supplementary file 1 [file animals-16-00692-s001.zip › S1_IL-10_m2_Group_Sex_Age_Weight.pdf]

lm(make\_response ~ l3[purchase] + GROUPS[Sex + Sex])

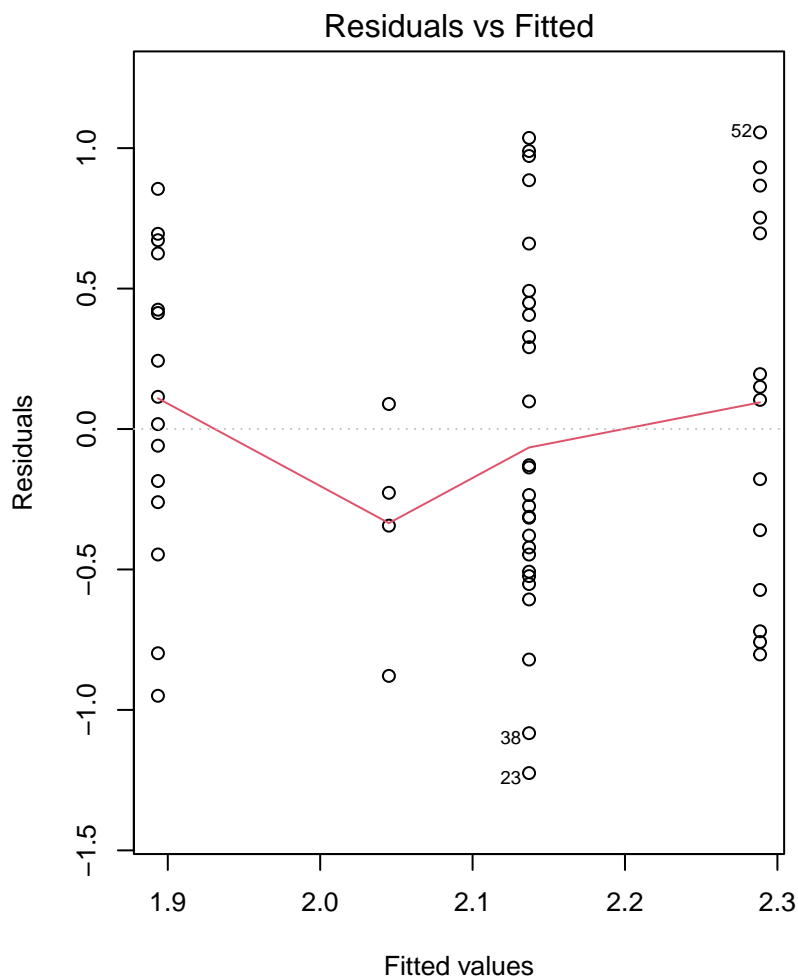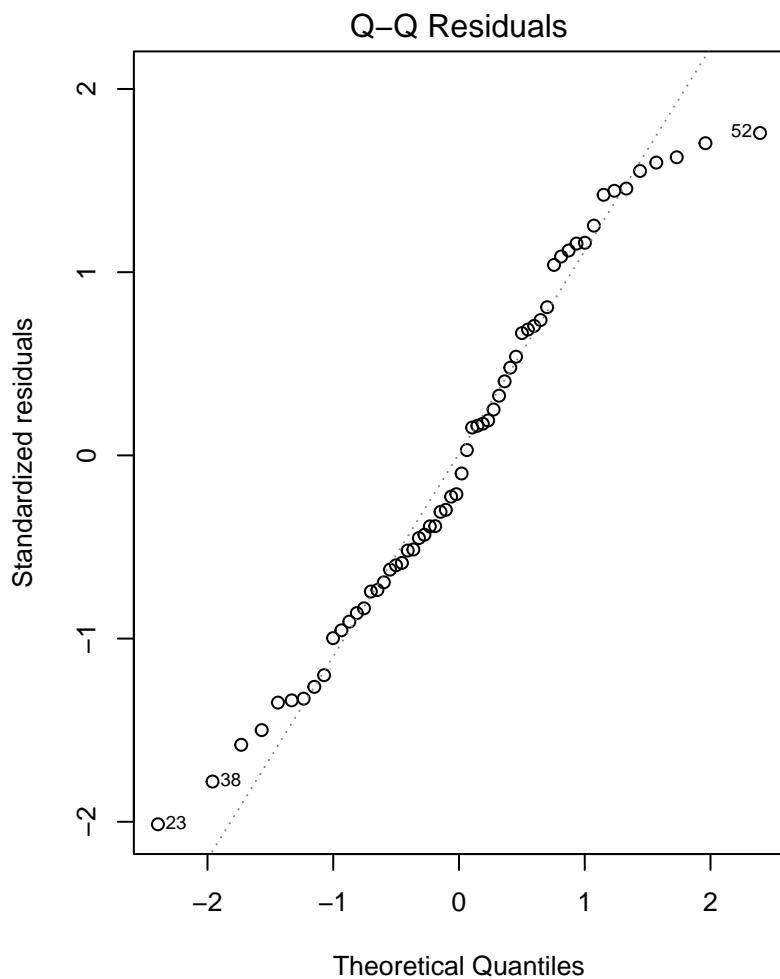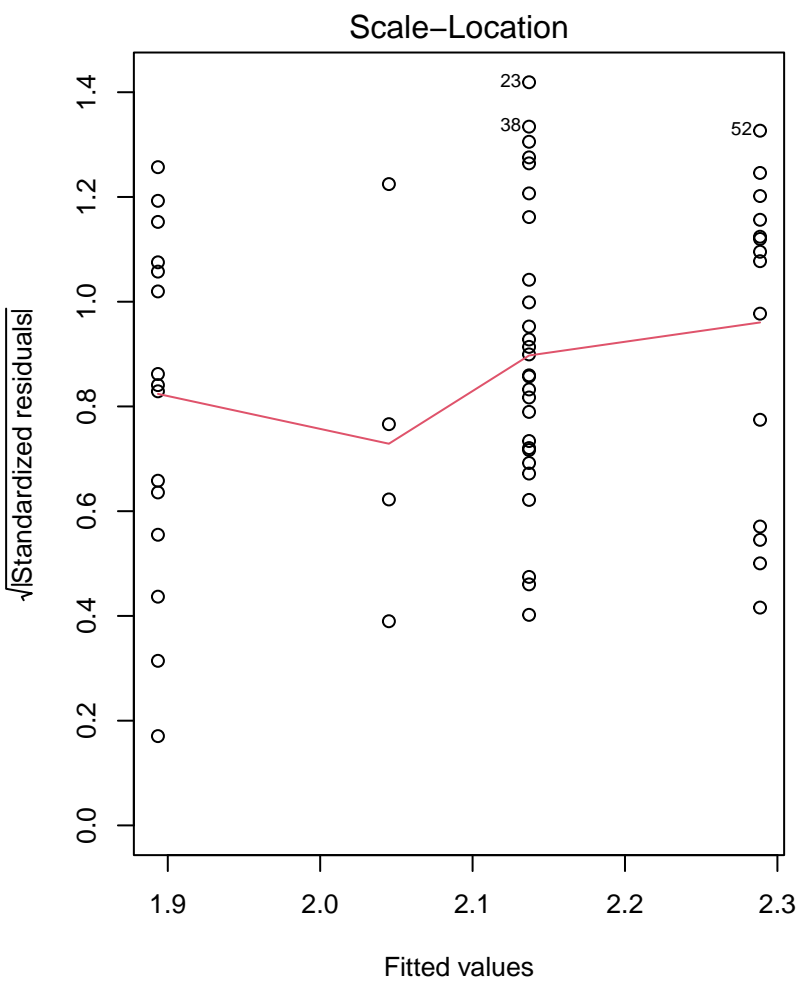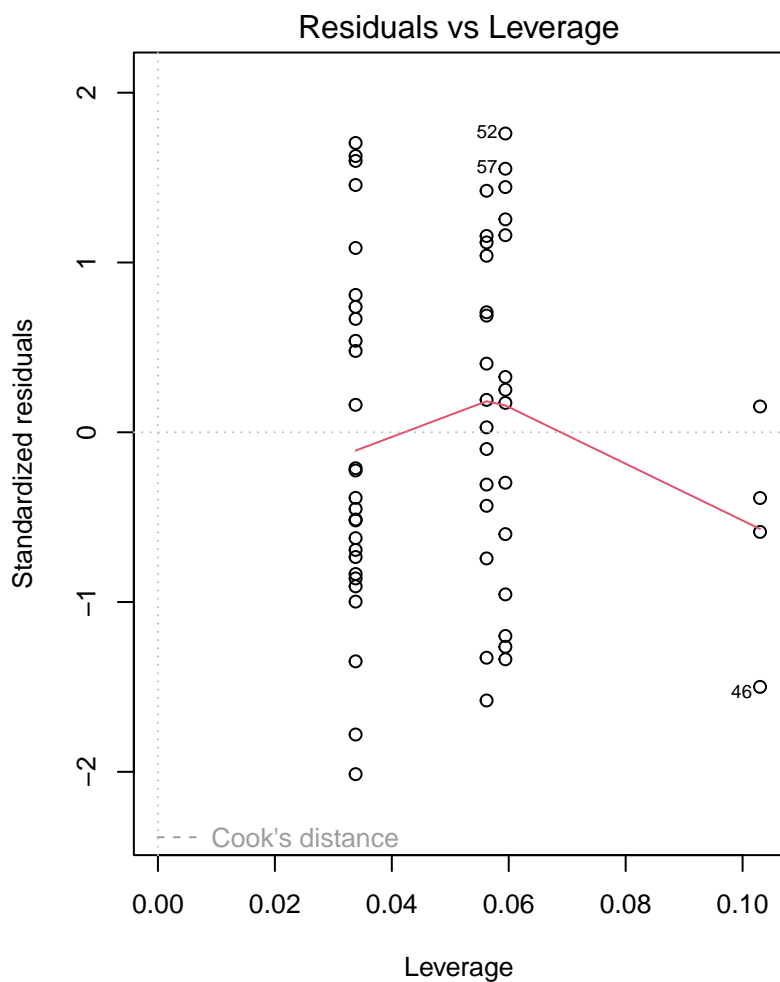

Supplement: Supplementary file 1 [file animals-16-00692-s001.zip › S1_IL-10_m3_GROUPII_Sex.pdf]

lm(make\_response ~ age + outcome + named\_group + sex + sex:age)

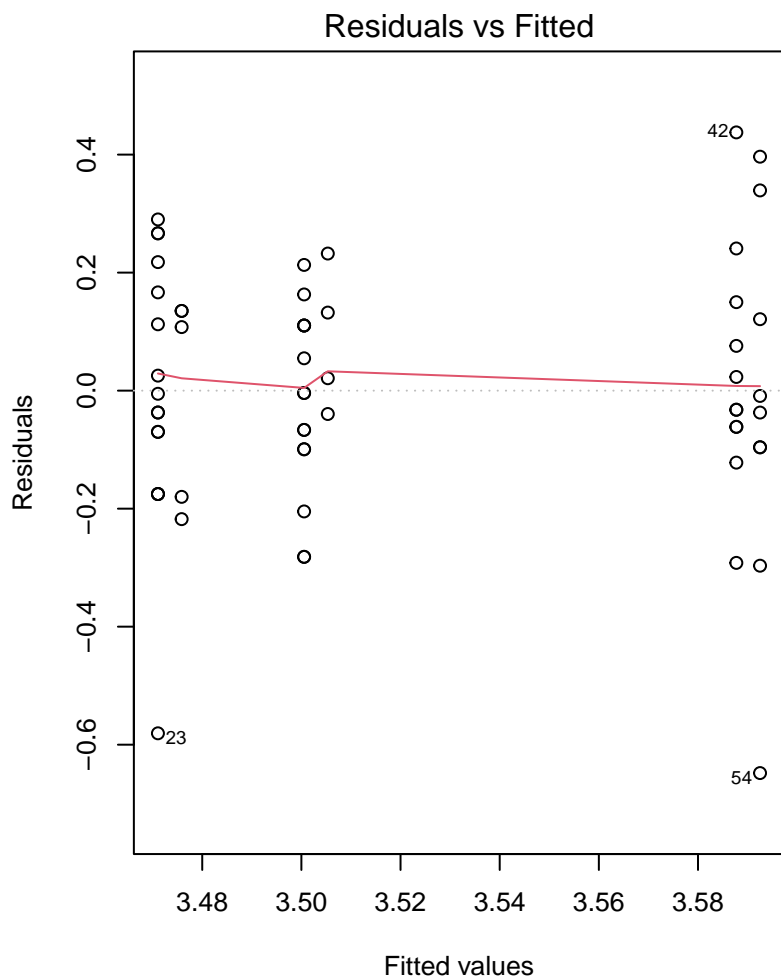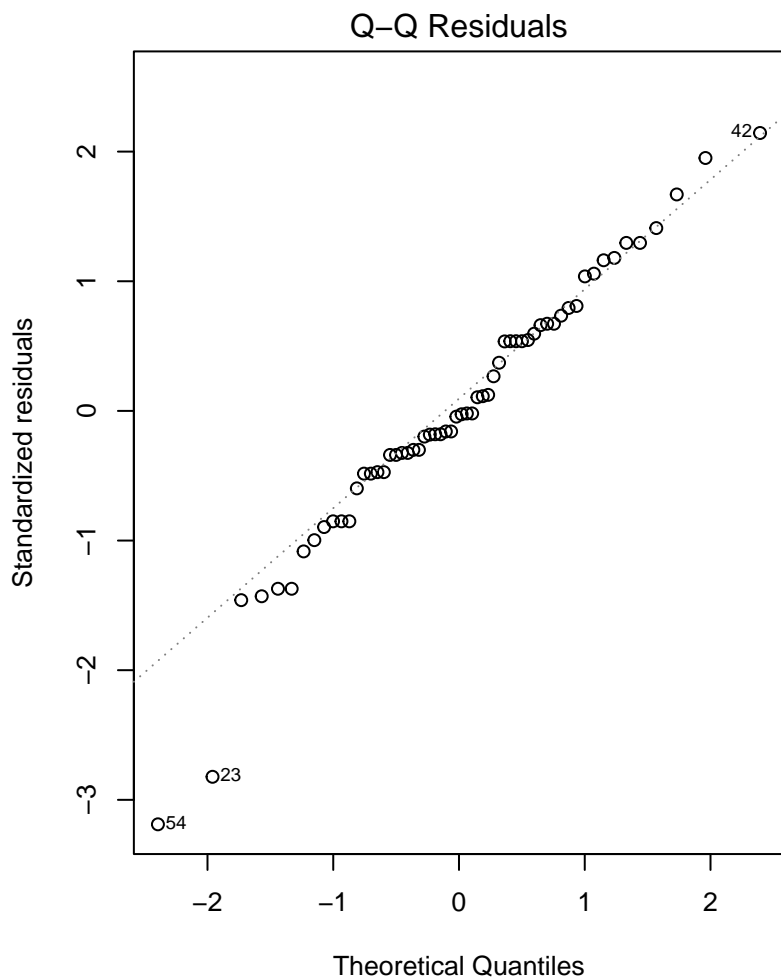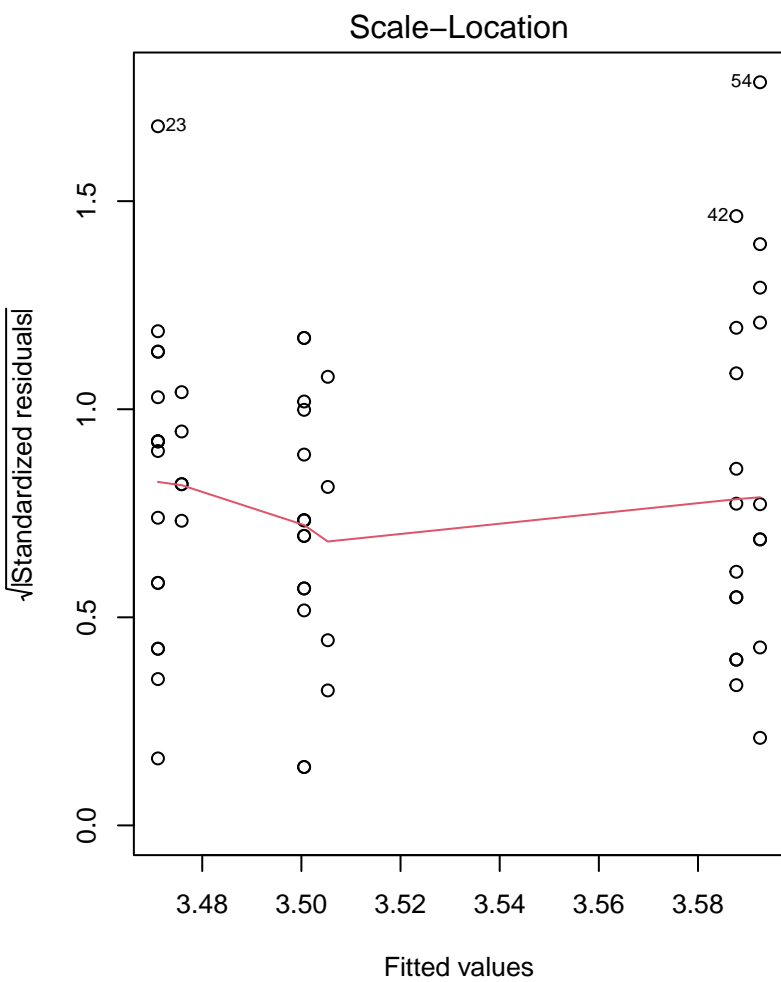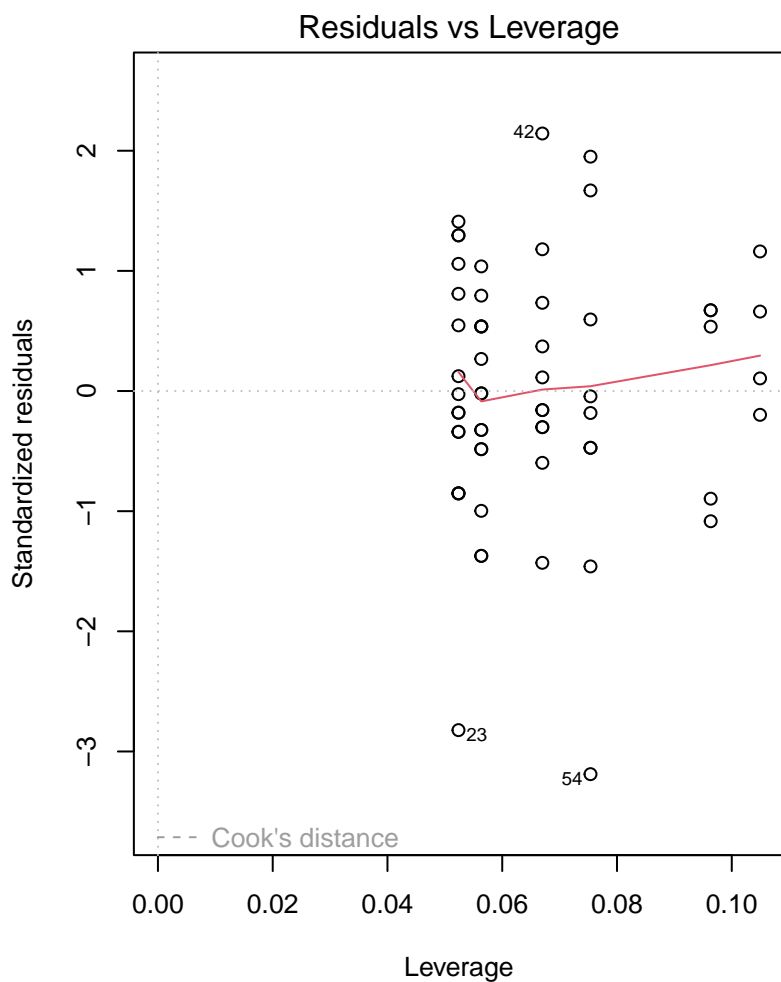

Supplement: Supplementary file 1 [file animals-16-00692-s001.zip › S1_Urea_m1_Group_Sex.pdf]

lm(make\_response ~ diff\_outcome + group + sex + age + weight)

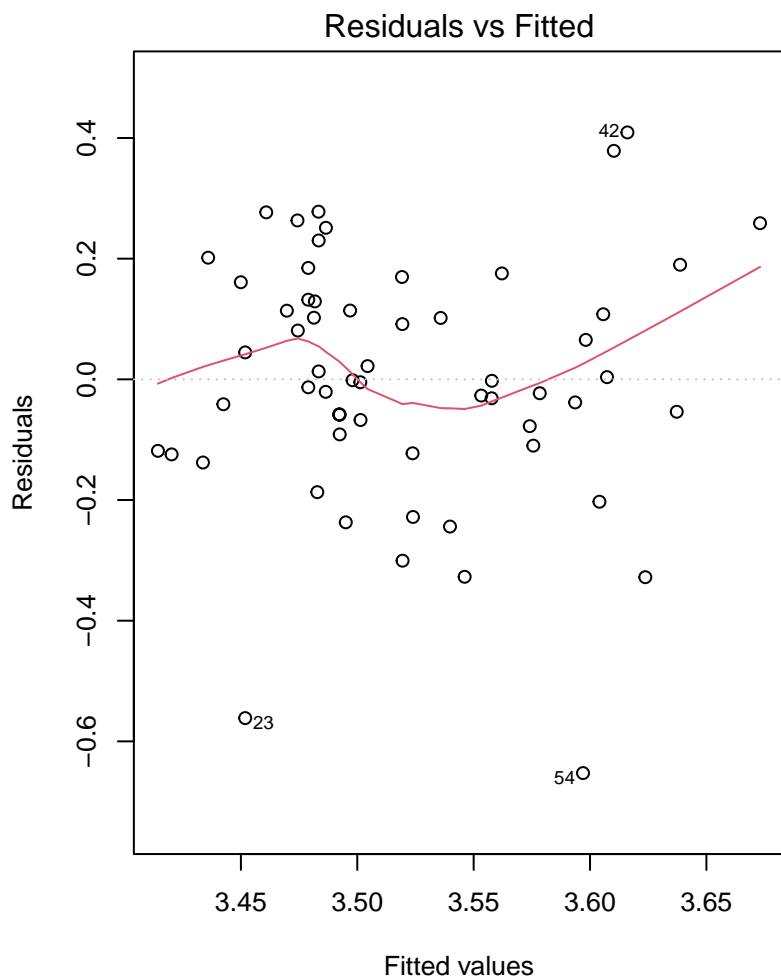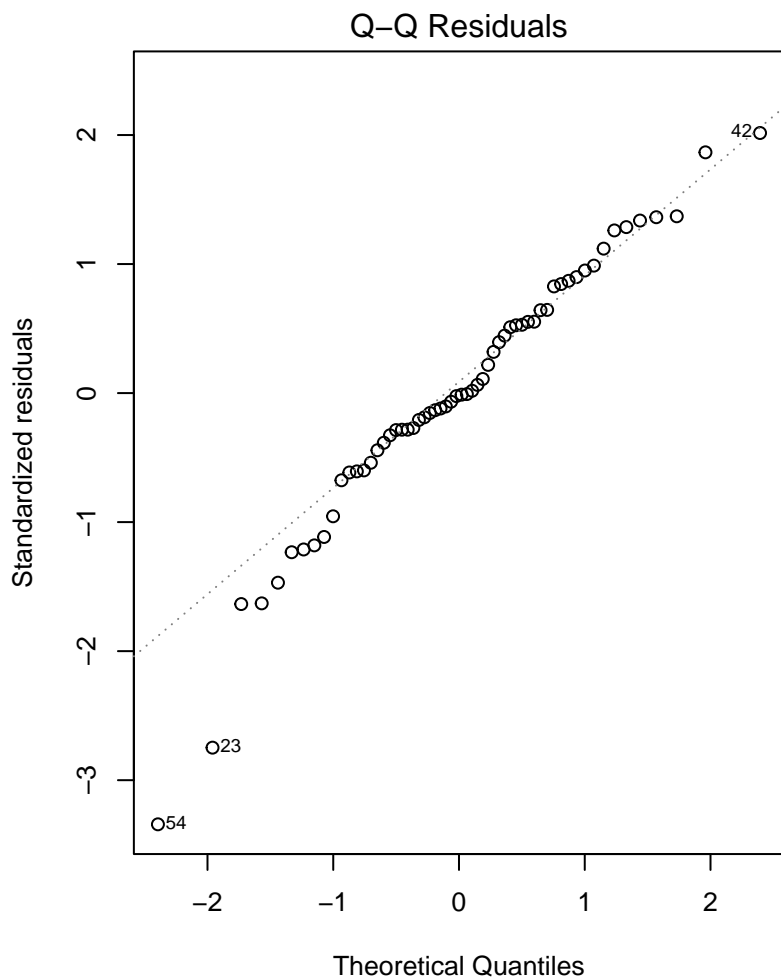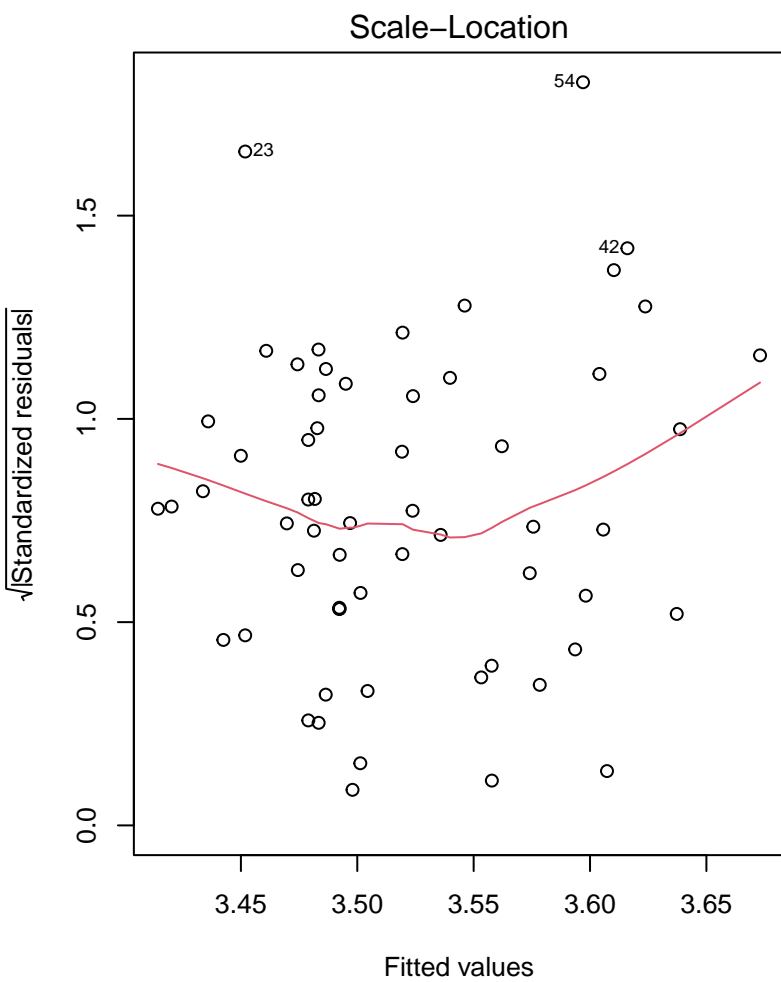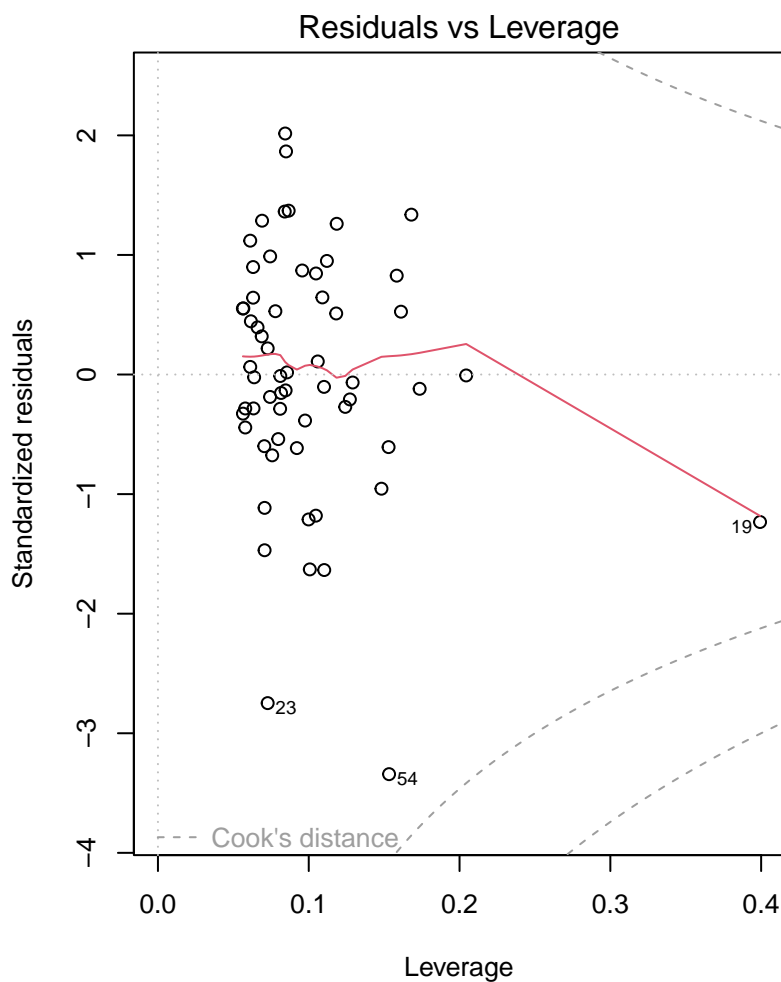

Supplement: Supplementary file 1 [file animals-16-00692-s001.zip › S1_Urea_m2_Group_Sex_Age_Weight.pdf]

lm(make\_response ~ log\_income + sex, data = log\_income\_data) >> plot

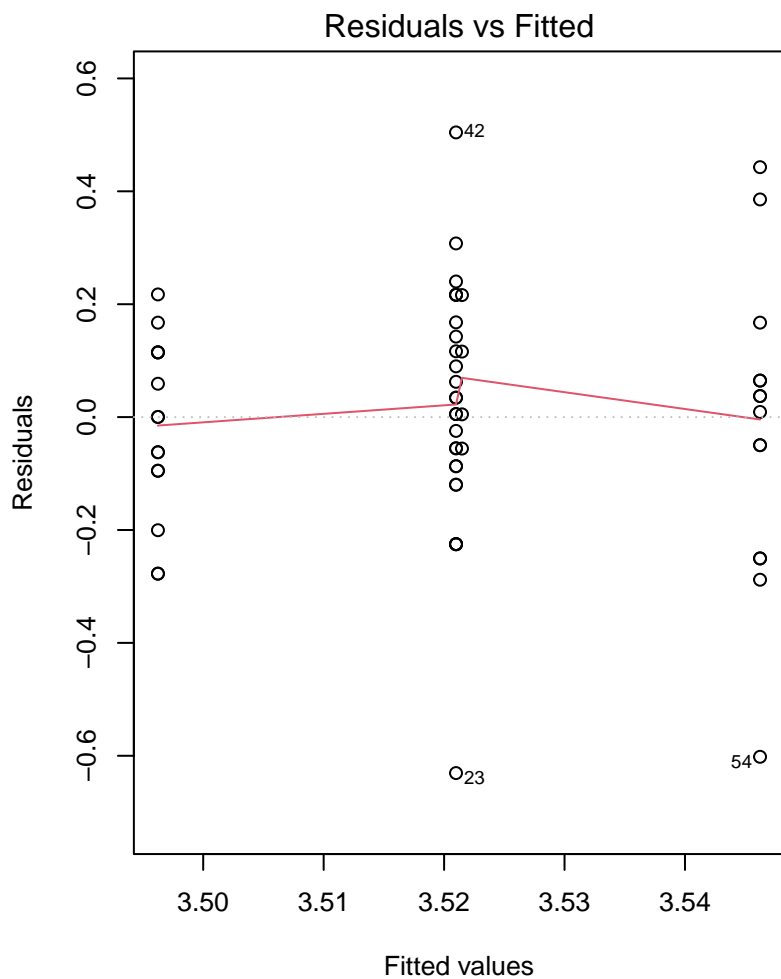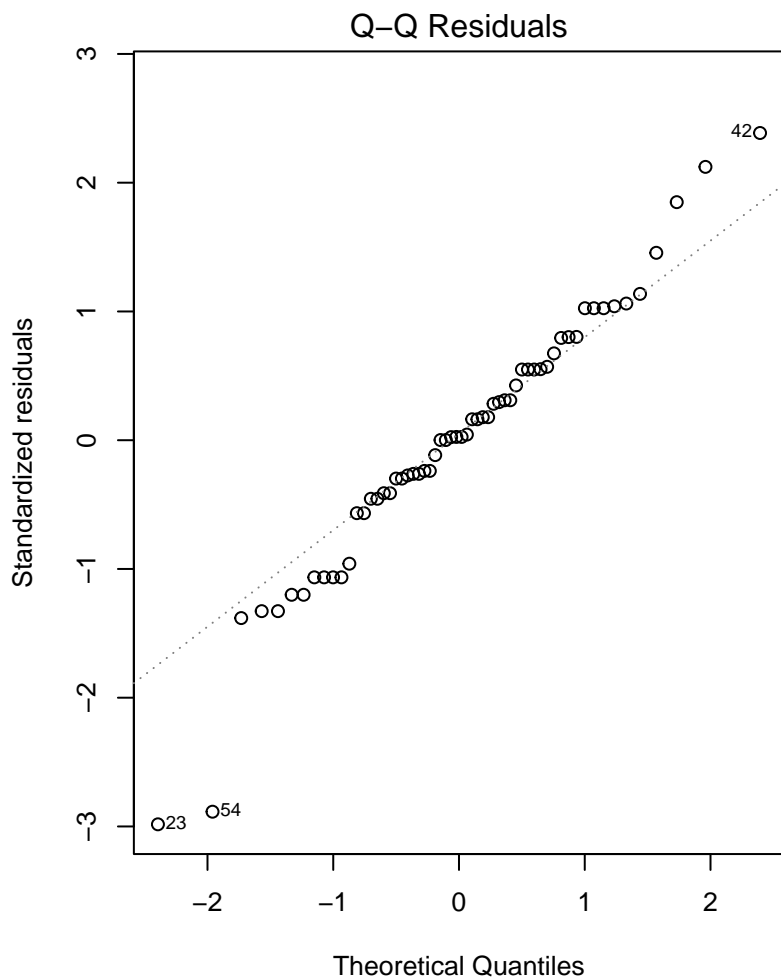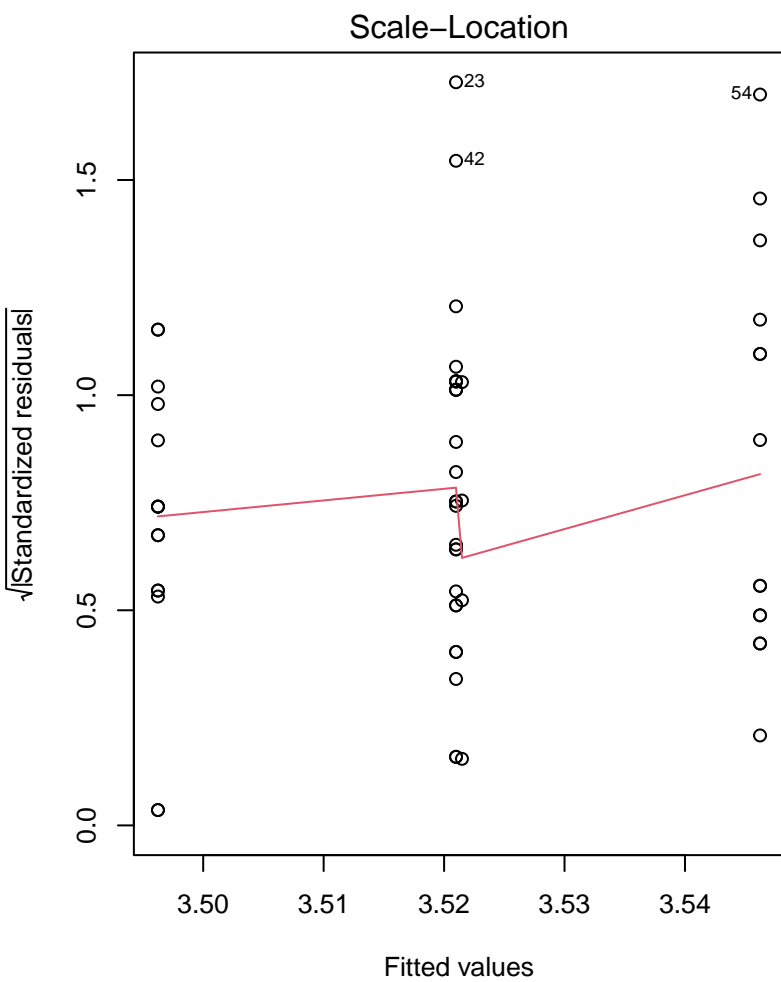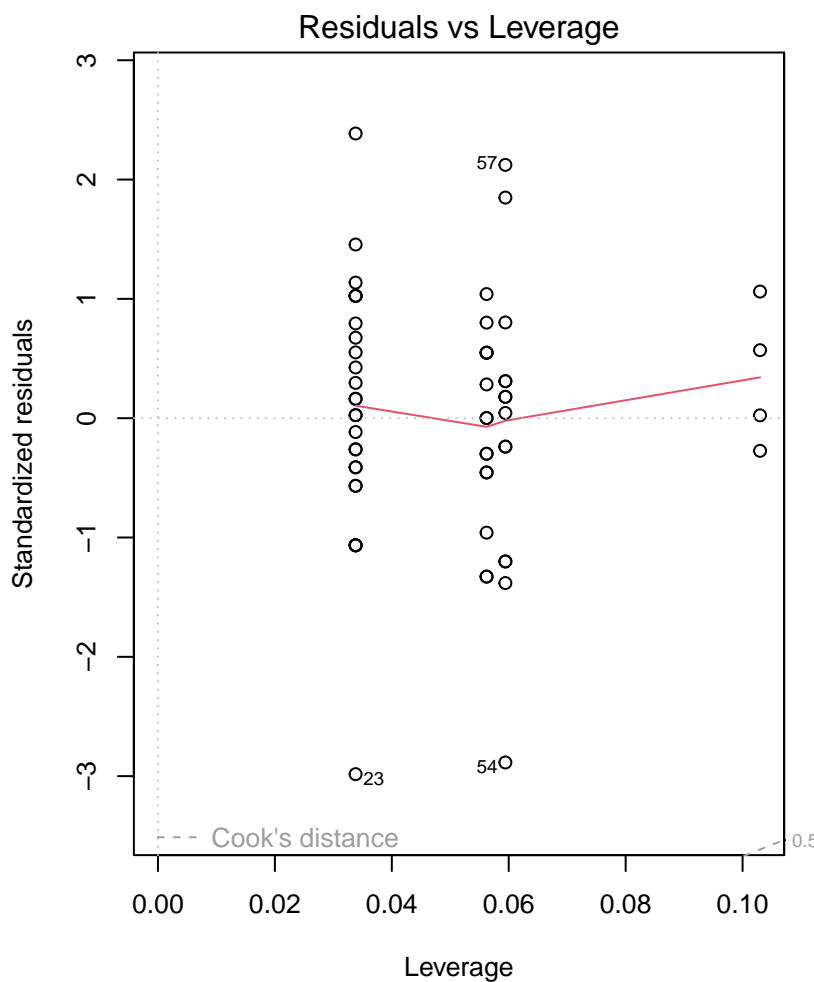

Supplement: Supplementary file 1 [file animals-16-00692-s001.zip › S1_Urea_m3_GROUPII_Sex.pdf]
